# Coal‐Tar Dye‐based Coordination Cages and Helicates

**DOI:** 10.1002/anie.202015246

**Published:** 2021-01-15

**Authors:** Irene Regeni, Bin Chen, Marina Frank, Ananya Baksi, Julian J. Holstein, Guido H. Clever

**Affiliations:** ^1^ Faculty of Chemistry and Chemical Biology TU Dortmund University Otto-Hahn-Strasse 6 44227 Dortmund Germany; ^2^ Current Address: State Key Laboratory of Radiation Medicine and Protection School for Radiological and Interdisciplinary Sciences (RAD-X) Soochow University Suzhou 215123 China

**Keywords:** chirality transfer, coordination cages, dyes, self-assembly, supramolecular chemistry

## Abstract

A strategy to implement four members of the classic coal‐tar dye family, Michler's ketone, methylene blue, rhodamine B, and crystal violet, into [Pd_2_L_4_] self‐assemblies is introduced. Chromophores were incorporated into bis‐monodentate ligands using piperazine linkers that allow to retain the auxochromic dialkyl amine functionalities required for intense colors deep in the visible spectrum. Upon palladium coordination, ligands with pyridine donors form lantern‐shaped dinuclear cages while quinoline donors lead to strongly twisted [Pd_2_L_4_] helicates in solution. In one case, single crystal X‐ray diffraction revealed rearrangement to a [Pd_3_L_6_] ring structure in the solid state. For nine examined derivatives, showing colors from yellow to deep violet, CD spectroscopy discloses different degrees of chiral induction by an enantiomerically pure guest. Ion mobility mass spectrometry allows to distinguish two binding modes. Self‐assemblies based on this new ligand class promise application in chiroptical recognition, photo‐redox catalysis and optical materials.

## Graphical abstract

A family of classic organic dyes, known since the advent of modern chemistry, was integrated into 3‐dimensional, self‐assembled nano‐objects via a novel piperazine‐based linker strategy that conserves the auxochromic substituents. The dynamic nature of the lantern‐shaped cages and helicates allows to recognize enantiopure guests via chirality transfer to the host, detected by circular dichroism response far in the visible region.

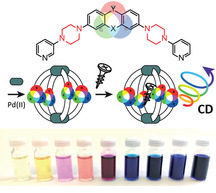

Organic chromophores have been integrated into supramolecular architectures to equip them with various functions. For example, artificial mimics for solar energy conversion often use porphyrin arrays as light harvesting units, capable of energy transfer and charge separation.^[1]^ Organic materials based on the non‐covalent arrangement of compounds with specific photophysical properties have been proposed as active components for displays, light sources, and optoelectronic circuits.^[2]^ Numerous examples for receptors with optical read‐out, photoswitchable self‐assemblies and light‐controlled molecular machines have been reported.^[3]^ Recently, photo‐redox chemistry started to take advantage of the supramolecular organization of organic or metal‐based chromophores into functional systems, for example, for catalytic oxidation of alcohols^[4]^ or cycloadditions to anthracenes.^[5]^ Dye‐based assemblies have further been studied for bio‐imaging^[6]^ and medicinal applications such as photodynamic therapy.^[7]^

With respect to discrete coordination cages with an accessible cavity (such as the popular [Pd_2_L_4_] systems),^[8]^ radical reactions have been realized in hosts where side‐walls can be brought to excited states by irradiation with light.^[9]^ Cages have also been used as photosensitizers for singlet O_2_ generation,^[10]^ and as reagents for photochemical hydrogen formation.^[11]^ Most reported systems, however, are based on chromophores that absorb in the UV to short‐wavelength visible region, giving rise to colorless or yellowish compounds. Exceptions include assemblies where highly colored charge‐transfer complexes are part of metal nodes,^[12]^ or metallo‐ligands^[13]^ and architectures based on photochromic diarylethenes (convertible between faint yellow and deep blue photoisomers)^[14]^ perylenes,^[15]^ porphyrins,^[16]^ triarylamines,^[17]^ and BODIPY.^[18]^

The so‐called “coal‐tar dyes” (such as di‐ or triarylmethanes, xanthenes, and phenothiazine derivatives) belong to the first block‐buster products of the emerging chemical industry in the 19th century. Until today, they are produced on large scales and find widespread application as redox and pH indicators,^[19]^ photosensitizers,^[20]^ dye‐based laser materials,^[21]^ for tissue staining,^[22]^ and imaging purposes,^[23]^ aside from their industrial application for coloring, for example, fabrics, polymers, paper, hair, and food. In the context of self‐assembled systems, however, they turn out to be underrepresented as integral structural elements and have been merely used as additives, for example, as photosensitizers for ^1^O_2_ generation.^[24]^ Non‐covalently encapsulated in metal‐organic hosts, such dyes were for example employed for proton reduction in combination with cobalt nodes in the architecture.^[25]^

Full integration of these chromophores into bridging positions of metallo‐supramolecular structures, however, requires functionalization with at least two distinct coordinating moieties. When examining the general structure of the herein studied dyes (box in Figure 1), it becomes apparent that the attachment of typical linkers such as alkynes or arenes is not feasible. In particular, removing the auxochromic dialkyl amines would not be tolerated, as they are fundamental parts of the delocalized π‐system, giving rise to intense colors in the visible region.

**Figure 1:**
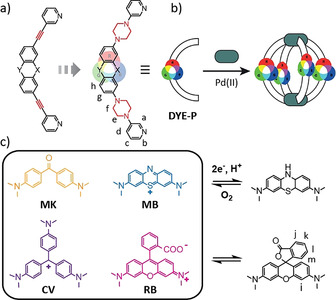
a) Design of dye‐based, bis‐monodentate ligands (**DYE‐P**) as opposed to ligands with alkyne bridges; b) formation of [Pd_2_L_4_] cages; c) organic chromophores chosen for this study: Michler's ketone **MK**, crystal violet **CV**, methylene blue **MB** (in blue/oxidized/cationic and colorless/reduced/uncharged states) and rhodamine B **RB** (in pink/zwitterionic and colorless/lactone forms).

Here we show how the implementation of piperazine linkers between coal‐tar dye‐based backbones (Michler's ketone **MK**, crystal violet **CV**, methylene blue **MB**, rhodamine B **RB**) and metal‐coordinating donors allows to retain the typical chromophore characteristics (Figure 1 a).^[26]^ Furthermore, as aliphatic linkers, piperazines should electronically decouple backbones from donors and at the same time not interfere with coordination to the metal centers, despite their ethylene diamine‐like substructure. Two choices of donor groups (3‐pyridyl **P** and 8‐isoquinolinyl **Q**) were examined, giving rise to eight (plus one derivative) ligands that were all found to form intensely colored Pd^II^‐mediated cages or helicates (ligand names are composed of backbone and donor label; see Figure S1 in the SI for all ligand structures). We further show how a chiral anionic guest translates its configurational information onto the assemblies and is able to discriminate between lantern‐shaped pyridyl cages and twisted isoquinolinyl helicates.

Ligand **RB‐P** was synthesized in only two steps by a Buchwald–Hartwig amination of literature‐described tosylated fluorescein with commercially available 3‐piperazyl‐pyridine. Following the same synthetic pattern, ligand **MK‐P** was obtained from 4,4′‐dihydroxybenzophenone. Ligand **CV‐P** was synthesized through a Grignard reaction of 4‐MgBr‐*N*,*N*‐dimethylaniline on ligand **MK‐P**. The synthesis of **MB‐P** followed a procedure already reported^[27]^ for substituted **MB** derivatives. Both **CV‐P** and **MB‐P** carry one positive charge and, as a final step of the synthesis, their halogen counter anions were exchanged for non‐coordinating anions (BF_4_^−^ or NO_3_^−^) by metathesis reactions with the corresponding silver salts.

Quantitative formation of cages [Pd_2_(**RB‐P**)_4_], [Pd_2_(**MK‐P**)_4_], [Pd_2_(**MB‐P**)_4_], and [Pd_2_(**CV‐P**)_4_] was achieved by mixing the respective ligands **RB‐P**, **MK‐P, MB‐P**, and **CV‐P** in a 2:1 ratio with the palladium(II) salts [Pd(CH_3_CN)_4_](BF_4_)_2_ or Pd(NO_3_)_2_ in [D_6_]DMSO at 70 °C for 15 min. In full accordance with our previously reported Pd^II^‐based cages,^[28]^ coordination of ligands to the metal centers was indicated by downfield shifting of the pyridine ^1^H NMR signals (in particular Ha and Hb, SI and Figure 2 a for [Pd_2_(**RB‐P**)_4_]). ^1^H DOSY NMR and high‐resolution ESI mass spectrometry further support the formation of the desired products (SI and Figure 2 b). Slow diffusion of toluene in the DMSO solution of [Pd_2_(**MK‐P**)_4_] (Figure 2 c), EtOAc in the DMSO solution of [Pd_2_(**RB‐P**)_4_] (Figure 2 d), and MTBE in the DMF solution of [Pd_2_(**CV‐P**)_4_] (Figure 2 e) afforded single crystals suitable for X‐ray diffraction. In the structure of [Pd_2_(**MK‐P**)_4_], all piperazines show a disorder concerning their chair‐type ring flip (occupancies approx. 50:50), revealing a certain flexibility of the linkers. The crystal structure of [Pd_2_(**RB‐P**)_4_] reveals two possible orientations for the spirolactone group in each ligand, giving rise to four possible isomers (Figure S46), which is consistent with the broadened ^1^H NMR spectrum observed for this cage.

**Figure 2:**
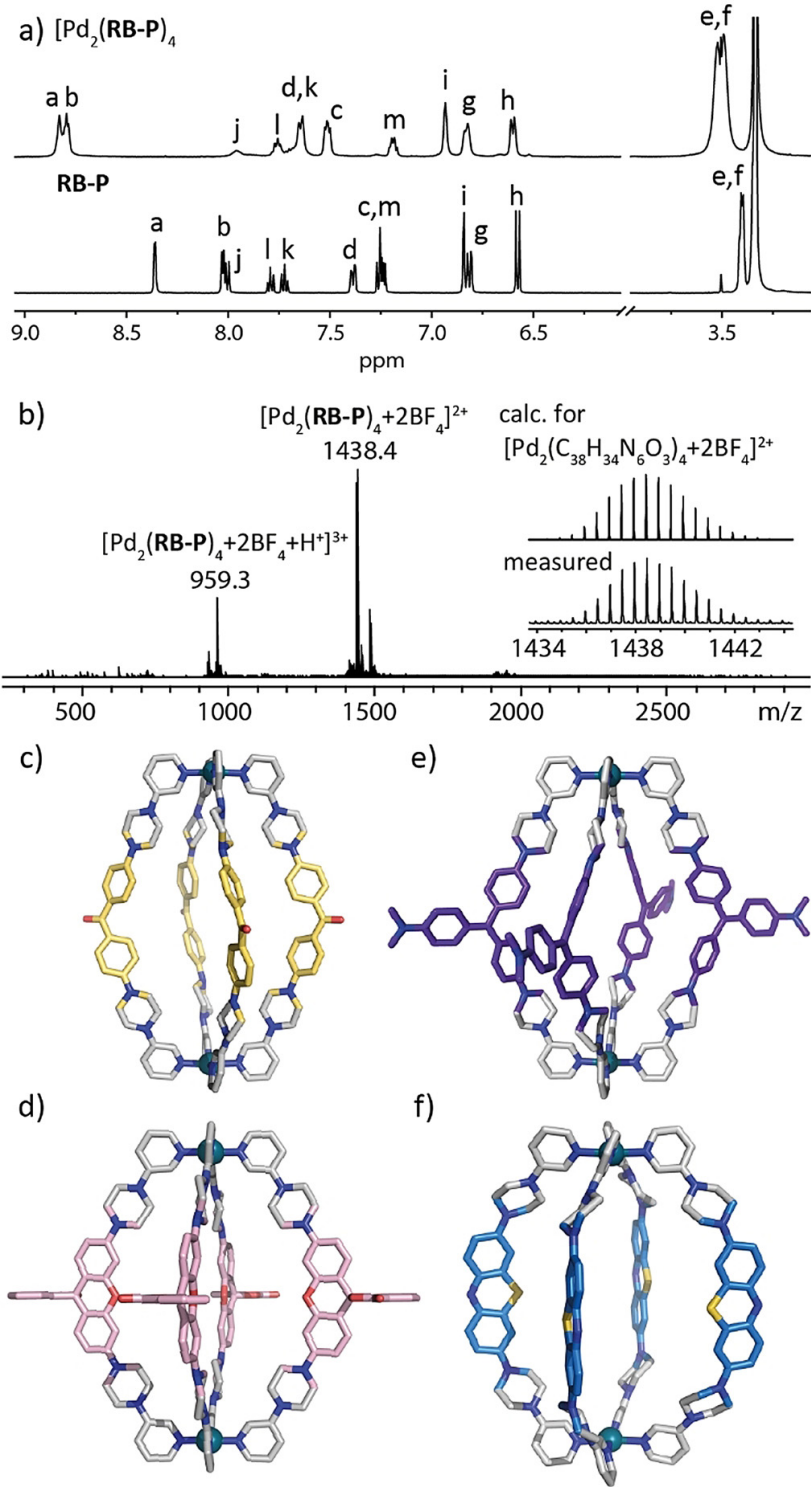
a) ^1^H NMR spectra in [D_6_]DMSO of ligand **RB‐P** and cage [Pd_2_(**RB‐P**)_4_]; b) ESI‐MS spectrum of cage [Pd_2_(**RB‐P**)_4_]. Side view of X‐ray crystal structures^[33]^ of c) [Pd_2_(**MK‐P**)_4_], d) [Pd_2_(**RB‐P**)_4_] (only one occupancy of disordered backbone shown), and e) [Pd_2_(**CV‐P**)_4_]; f) DFT (B3LYP/def2‐SV(P)) model of [Pd_2_(**MB‐P**)_4_]. Counter anions and solvent molecules omitted.

**MB** is known to be easily reduced to its colorless leuco form, prompting us to investigate whether this behavior is retained in cage [Pd_2_(**MB‐P**)_4_]. Indeed, treating cage [Pd_2_(**MB‐P**)_4_] with Zn powder induced partial decoloration and revealed a statistical distribution of oxidized (**MB‐P**) and reduced (**MB^R^‐P**) ligands by ESI‐MS analysis, with observed signals described by the formula {[Pd_2_(**MB‐P**)_*x*_(**MB^R^‐P**)_4−*x*_](BF_4_)_4+*x*−*z*_}^*z*+^(*x=*1–3; *z=*2–4; Figure S63).

Next, we introduced isoquinoline donors in order to bestow the [Pd_2_L_4_] assemblies with a helical twist (Figure 3). According to the above‐mentioned procedures, ligands **MK‐Q**, **RB‐Q**, **MB‐Q**, **CV‐Q** were synthesized using 8‐(piperazin‐1‐yl)isoquinoline. Corresponding [Pd_2_(**DYE‐Q**)_4_] helicates were assembled as described for the pyridine derivatives, but differ from the latter by their characteristic dynamic behavior. As previously reported,^[29]^ [Pd_2_L_4_] coordination cages featuring inward‐pointing isoquinolin‐8‐yl donors adopt a helical structure with ligands twisting around the Pd_2_‐axis, resulting in *M*‐ and *P*‐configured chiral conformers. Figure 3 b depicts the ^1^H NMR spectrum of [Pd_2_(**MB‐Q**)_4_] at different temperatures in [D_6_]DMSO. At room temperature, the C*H*_2_ protons of the piperazine moieties split into eight broad signals, resulting from slow ring flip dynamics and their diastereotopic nature within the chiral environment. At 75 °C all eight signals coalesce into two broad peaks, as expected for a situation where both the piperazine ring flip as well as the overall cage twist are fast on the NMR time scale. We assumed these dynamics to be dependent on the solvent. Indeed, the ^1^H NMR spectrum of [Pd_2_(**MB‐Q**)_4_] in D_2_O at room temperature shows only two signals (Figure S99), indicating faster exchange dynamics in the aqueous medium.

**Figure 3:**
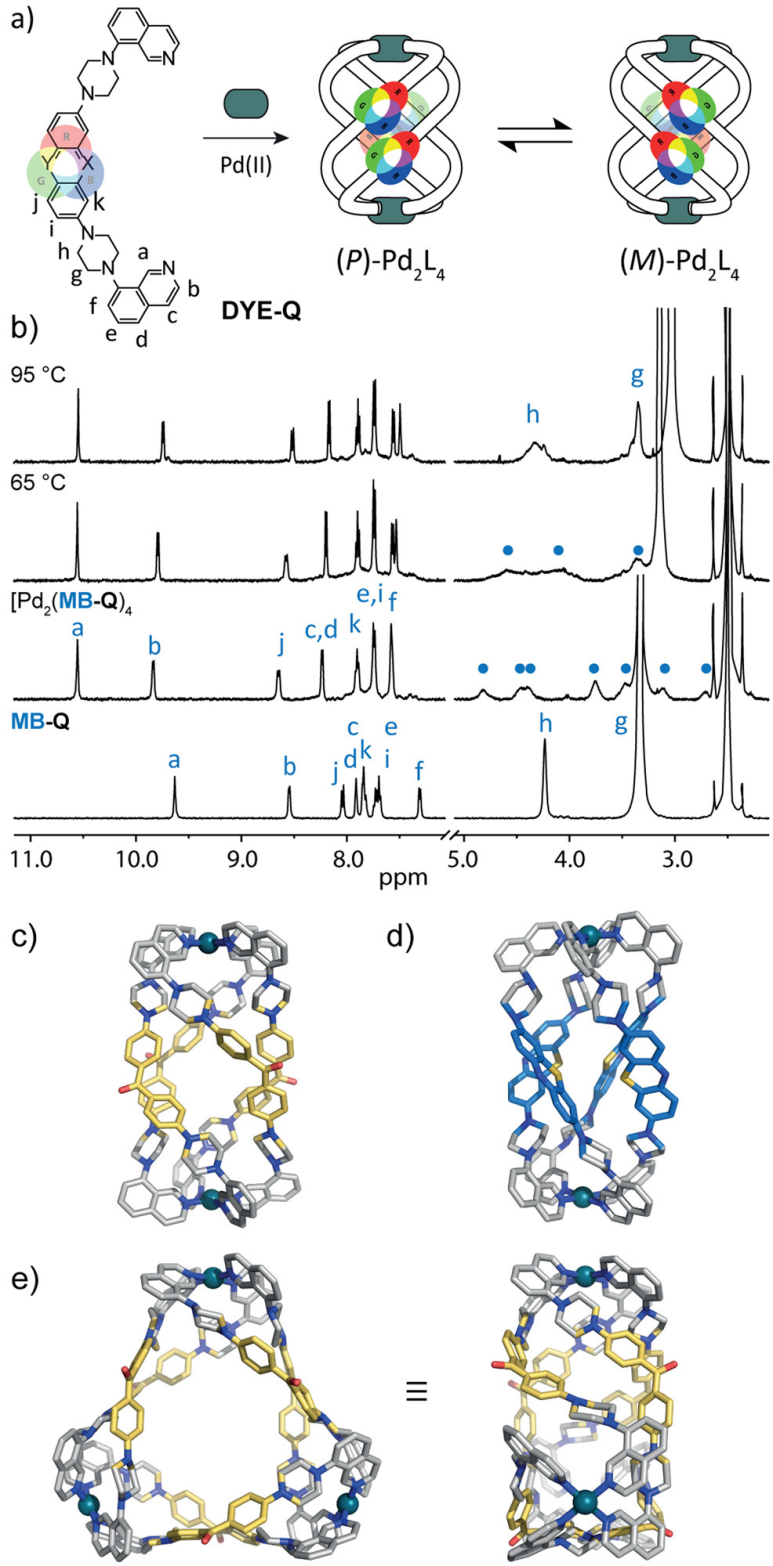
a) Generic bis‐isoquinolinyl ligand (**DYE‐Q**) forming a helical cage in two enantiomeric forms; b) ^1^H NMR spectra in [D_6_]DMSO of **MB‐Q** and [Pd_2_(**MB‐Q**)_4_] at indicated temperatures. DFT models (B3LYP/def2‐SV(P) of c) [Pd_2_(**MK‐Q**)_4_] and d) [Pd_2_(**MB‐Q**)_4_]; e) top and side views of the X‐ray crystal structure of [Pd_3_(**MK‐Q**)_6_].^[33]^ Counter anions and solvent molecules omitted.

For [Pd_2_(**MK‐Q**)_4_], a similar NMR behavior was observed (Figure S94). By slow vapor diffusion of toluene into the DMSO solution of [Pd_2_(**MK‐Q**)_4_], small yellow crystals suitable for synchrotron X‐ray diffraction were obtained. While ESI‐MS and DOSY NMR clearly support the dinuclear [Pd_2_(**MK‐Q**)_4_] stoichiometry in solution (Figures S90–S93; model Figure 3 c), the obtained solid‐state structure surprisingly revealed formation of a three‐membered ring [Pd_3_(**MK‐Q**)_6_] (Figure 3 e). We assume this structural rearrangement to occur in the course of the crystallization process, facilitated by the conformational flexibility of the **MK** backbone.

Next, we analyzed cage [Pd_2_(**RB‐Q**)_4_]. Its ^1^H NMR spectrum at room temperature is broadened due to signal coalescence. At higher temperatures, some signals sharpen, while at lower temperatures signals sharpen but show more complicated splitting patterns. Since its ESI‐MS analysis indicated the exclusive formation of [Pd_2_(**RB‐Q**)_4_], we assign the convoluted NMR behavior to the isomerism of the spirolactones (as discussed for [Pd_2_(**RB‐P**)_4_]), in combination with dynamic interconversion between two chiral conformers (Figure S97). A most interesting feature of **RB** is its solvent polarity‐dependent transformation between a colorless spirolactone form and an intensely pink zwitterionic open form (Figure 1). Indeed, exposing a colorless solution of [Pd_2_(**RB‐P**)_4_] in DMSO (Reichardt solvent polarity E_T_^N^=0.44)^[30]^ to increasing amounts of acetonitrile (E_T_^N^=0.46), methanol (E_T_^N^=0.76), or water (E_T_^N^=1.00) leads to a stepwise increase of the typical pink color (Figure 4 a). Since corresponding NMR spectra showed strong signal broadening, hampering quantification of the fraction of opened chromophores, we converted ligand **RB‐P** into ligand **RE‐P** in which the colored open form is secured by esterification of the carboxylic moiety. The corresponding cage [Pd_2_(**RE‐P**)_4_] could then be formed and unambiguously characterized as described for the other systems (see Section 3.2.5 in the SI).

**Figure 4:**
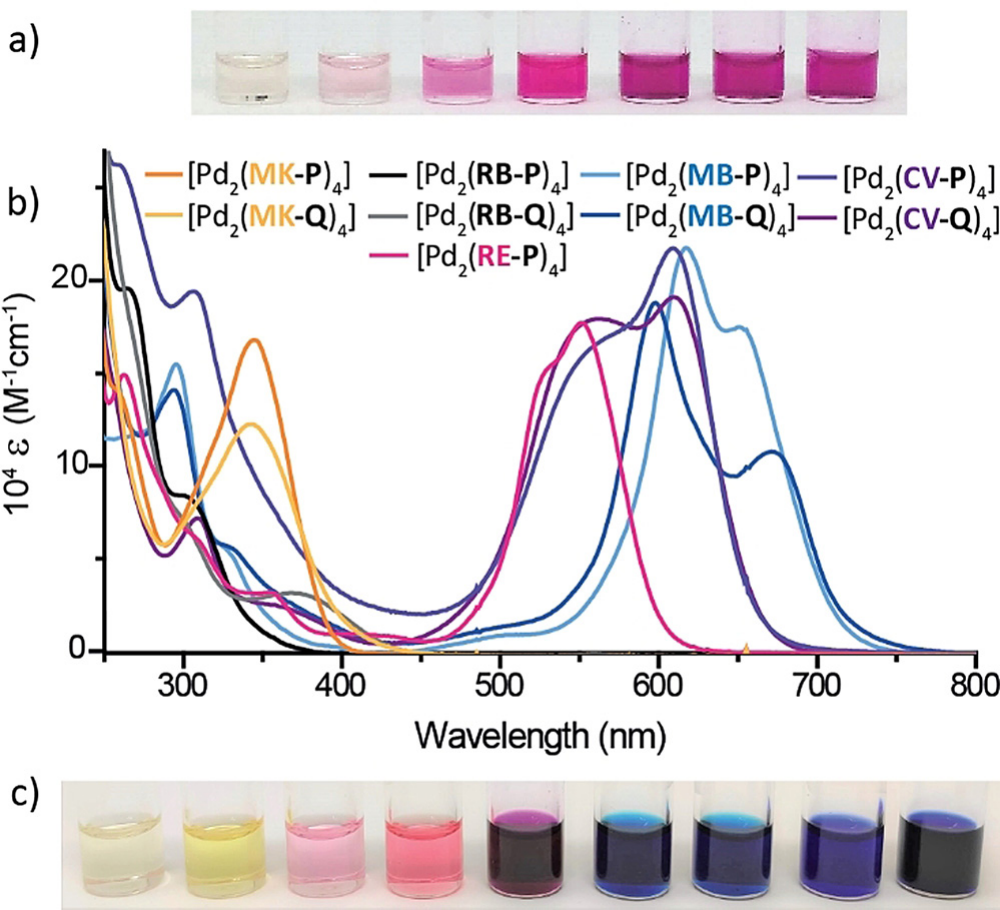
Photos of 0.7 mm solutions of a) [Pd_2_(**RB‐P**)_4_] from pure DMSO to DMSO/D_2_O 9:1 (left to right). b) UV/Vis absorption spectra of all nine colored assemblies in DMSO; c) 0.7 mm DMSO solutions (order as labels in (b)).

Next, we recorded ^1^H DOSY NMR spectra of the colored **DYE‐P** and **DYE‐Q** ligands and corresponding assemblies to compare their solution state dimensions. Interestingly, while ligands **DYE‐Q** are larger than **DYE‐P** in terms of their larger donor group, [Pd_2_(**DYE‐Q**)_4_] helicates are characterized by slightly smaller hydrodynamic radii as compared to their **DYE‐P** siblings (Table S2). This observation is in line with the X‐ray/DFT structure results, showing a more globular shape with larger cavity for the [Pd_2_(**DYE‐P**)_4_] cages and twisted structures with smaller and less accessible cavities for the [Pd_2_(**DYE‐Q**)_4_] helicates (Figure 2, 3, and S115).

Owing to the systems’ 3D chromophore arrangement and strong absorption in the visible spectrum (Figures 4 b,c), we examined their application in the chiral recognition of small molecules. First, encapsulation of (*R*)‐camphor sulfonate (**CSA**) in cages [Pd_2_(**RB‐P**)_4_] and [Pd_2_(**RE‐P**)_4_] in DMSO could be verified by ^1^H NMR spectroscopy (shift of inward‐pointing host protons) and ESI‐MS spectrometry. However, neither of these host–guest complexes showed a CD signature in the region 250–800 nm, which would be indicative of a significant guest‐to‐host chirality transfer. As similar observations were made for all other seven cages (see SI for ESI‐MS and CD spectra), we assume that the rather small, singly charged (*R*)‐**CSA** molecule is not able to induce preferred population of one diastereomeric host–guest complex over the other.^[31]^ We then tested the larger, double negatively charged guest (*R*)‐1,1′‐binaphthyl‐2,2′‐disulfonate (**BINSO_3_**). Equimolar solutions of each of all nine colored cages together with (*R*)‐**BINSO_3_** were prepared in DMSO and a selection of CD and UV/Vis absorption spectra is shown in Figure 5 a. In spectral regions were the cages (but not the guest) absorb, induced CD signals originate from guest‐to‐host chirality transfer (for further spectra see the SI).

**Figure 5:**
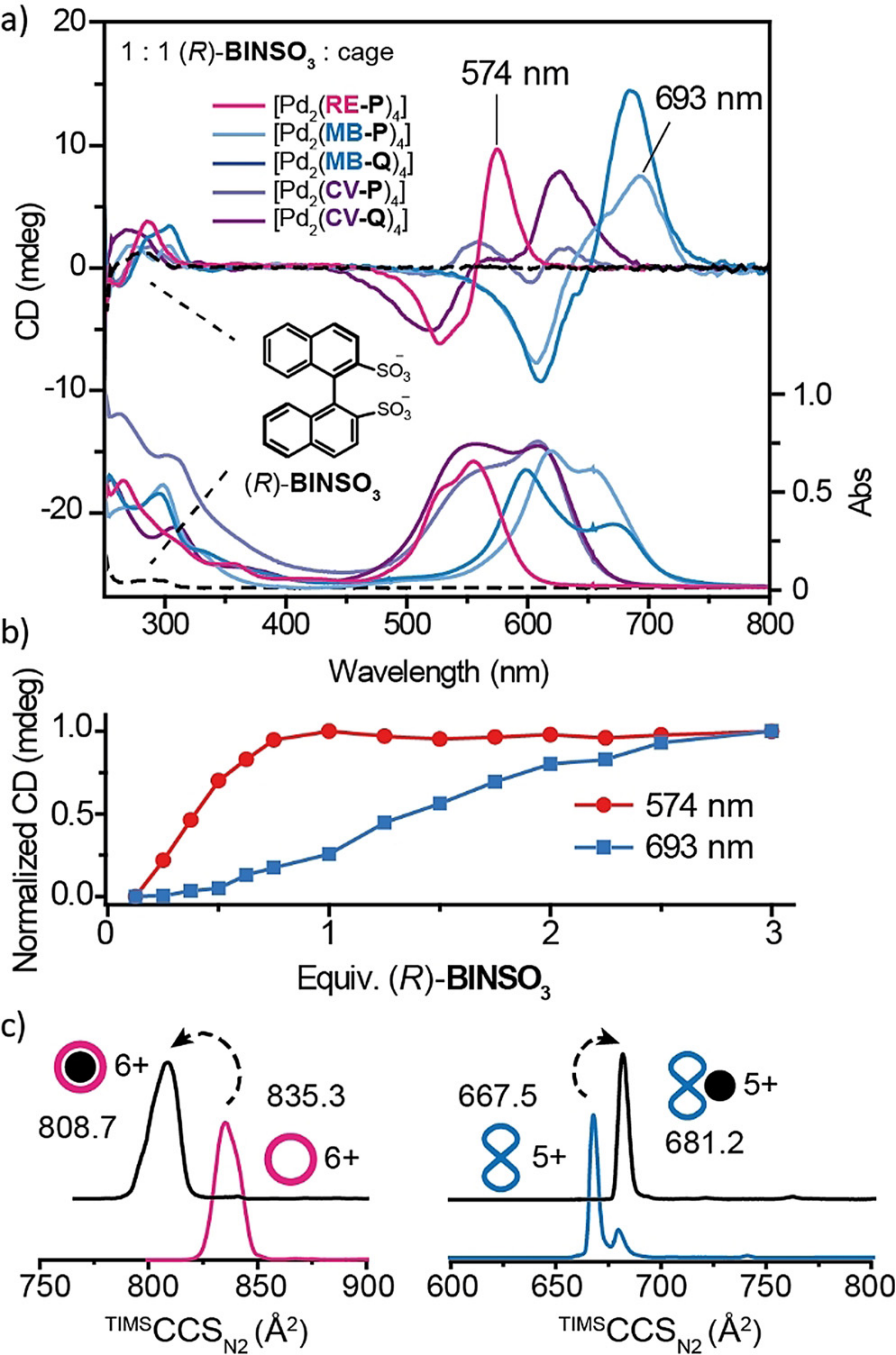
a) CD and UV/Vis absorption spectra of 18.75 μm cage (indicated in the legend) solution with 1 equiv of guest (*R*)‐**BINSO_3_** in DMSO at 25 °C. b) CD intensities at 574 and 693 nm of a 18.75 μm 1:1 mixed solution of cages [Pd_2_(**RE‐P**)_4_] and [Pd_2_(**MB‐Q**)_4_] at increasing amounts of (*R*)‐**BINSO_3_** in DMSO at 25 °C. c) Trapped ion mobility experiments showing guest encapsulation for cage [Pd_2_(**RE‐P**)_4_] vs. outside association for helicate [Pd_2_(**MB‐Q**)_4_].

Next, we examined differential guest binding preference by adding (*R*)‐**BINSO_3_** to a kinetically trapped, narcissistic 1:1 mixture of lantern‐shaped [Pd_2_(**RE‐P**)_4_] and helical‐shaped [Pd_2_(**MB‐Q**)_4_], showing that the guest has a stronger preference to interact with the pink globular cage rather than the blue helical one. The clearly distinguishable CD signatures of both cages allowed to follow this discrimination process in a titration experiment: while the induced CD band at 574 nm increases until one equivalent of guest is added, the one at 693 nm shows its steepest ascent after the 1:1 stoichiometry is reached (Figure 5 b). We ascribe this to the propensity of (*R*)‐**BINSO_3_** to bind inside the cage cavity of the more globular [Pd_2_(**DYE‐P**)_4_] systems (as confirmed by ^1^H NMR titrations), while for the quinolinyl‐based helicates, we propose rather weak outside binding. In fact, titration of (*R*)‐**BINSO_3_** into the [Pd_2_(**MB‐Q**)_4_] solution leads to a mere decrease of all NMR signals, indicative for an anion‐induced aggregation of the cationic assemblies. Moreover, while ESI‐MS spectra of (*R*)‐**BINSO_3_** with cages [Pd_2_(**DYE‐P**)_4_] show only peaks for the host–guest complexes, for helicates [Pd_2_(**DYE‐Q**)_4_], the respective peaks are either not found at all (for [Pd_2_(**MK‐Q**)_4_] and [Pd_2_(**RB‐Q**)_4_]) or are rather small and accompanied by multiple peaks of the free host. Here, trapped ion mobility mass spectrometry (TIMS) proved helpful to differentiate the proposed binding modes: While inside encapsulation led to contraction of the cage–guest complex, outside association caused a collisional cross section (CCS) increase instead (Figure 5 c and SI).

In conclusion, we report nine [Pd_2_L_4_] assemblies based on a new family of functional bis‐monodentate ligands, designed from coal‐tar dyes, bridged via auxochromic piperazines to two types of N‐donors. Pyridine‐based ligands afford lantern‐shaped cages with accessible cavities while isoquinoline‐based ligands lead to racemic mixtures of strongly twisted helicates. All assemblies were shown to maintain the electronic structure of the parental dyes, which was exploited in the chiral recognition of small anions. NMR and CD titration as well as ion mobility mass spectrometry indicate that the chiral anions interact via an encapsulation mode for the cages and outside‐association mode for the helicates. This new class of dye‐functionalized assemblies, spanning a color spectrum from yellow to blue/violet, based on chromophores with a well‐established application range, sets the stage for the development of photo‐redox catalysts with confined reaction chambers, light harvesting devices, as well as diagnostic tools for the chiroptical detection of biomolecules and ‐polymers.

## Conflict of interest

The authors declare no conflict of interest.

## Supporting information

As a service to our authors and readers, this journal provides supporting information supplied by the authors. Such materials are peer reviewed and may be re‐organized for online delivery, but are not copy‐edited or typeset. Technical support issues arising from supporting information (other than missing files) should be addressed to the authors.

Supplementary
