# Supplementary material for: Coal‐Tar Dye‐based Coordination Cages and Helicates
Source: Angew Chem Int Ed Engl. 2021 Jan 15;60(11):5673–8. doi: 10.1002/anie.202015246 (PMC7986857; doi:10.1002/anie.202015246)
Supplement: Supplementary file 1 — Supplementary [file ANIE-60-5673-s001.pdf]

Angewandte Chemie
Eine Zeitschrift der Gesellschaft Deutscher Chemiker

Supporting Information

# Coal-Tar Dye-based Coordination Cages and Helicates

*Irene Regeni, Bin Chen, Marina Frank, Ananya Baksi, Julian J. Holstein, and Guido H. Clever**

anie_202015246_sm_miscellaneous_information.pdf

1 Ligands used in this study ........ 2
2 General Methods ........ 3
2.1 NMR ........ 3
2.2 Mass spectrometry and ion mobility measurements ........ 3
2.3 UV-Vis ........ 3
2.4 CD ........ 3
2.5 Computational studies ........ 3
3 Experimental Procedures ........ 3
3.1 Synthesis of the ligands ........ 4
3.1.1 Synthesis of MK-P ........ 4
3.1.2 Synthesis of RB-P ........ 6
3.1.3 Synthesis of MB-$PBF_4$ and MB-$PNO_3$ ........ 8
3.1.4 Synthesis of CV-P ........ 12
3.1.5 Synthesis of RE-P ........ 16
3.1.6 Synthesis of 8-(piperazin-1-yl)isoquinoline (7) ........ 18
3.1.7 Synthesis of MK-Q ........ 20
3.1.8 Synthesis of RB-Q ........ 21
3.1.9 Synthesis of MB-Q ........ 23
3.1.10 Synthesis of CV-Q ........ 25
3.2 Assembly of the cages and helicates ........ 28
3.2.1 $[Pd_2(MK\text{-}P)_4](BF_4)_4$ ........ 28
3.2.2 $[Pd_2(RB\text{-}P)_4](BF_4)_4$ ........ 33
3.2.3 $[Pd_2(MB\text{-}P)_4](BF_4)_8$ and $[Pd_2(MB\text{-}P)_4](NO_3)_8$ ........ 39
3.2.4 $[Pd_2(CV\text{-}P)_4](NO_3)_8$ ........ 48
3.2.5 $[Pd_2(RE\text{-}P)_4](EtSO_4)_4(BF_4)_4$ ........ 51
3.2.6 $[Pd_2(MK\text{-}Q)_4](BF_4)_4$ ........ 55
3.2.7 $[Pd_2(RB\text{-}Q)_4](BF_4)_4$ ........ 61
3.2.8 $[Pd_2(MB\text{-}Q)_4](NO_3)_8$ ........ 63
3.2.9 $[Pd_2(CV\text{-}Q)_4](NO_3)_8$ ........ 67
4 $^1$H-DOSY spectroscopy ........ 72
5 X-ray Crystal Structures ........ 75
5.1 Crystal structure of $[Pd_2(MK\text{-}P)_4]$ ........ 76
5.2 Crystal structure of $[Pd_2(RB\text{-}P)_4]$ ........ 77
5.3 Crystal structure of $[Pd_2(CV\text{-}P)_4]$ ........ 78
5.4 Crystal structure of $[Pd_3(MK\text{-}Q)_6]$ ........ 79
6 UV-Vis absorption spectroscopy ........ 80
7 Host-Guest Chemistry ........ 80
7.1 (R)-camphor sulfonate (CSA) as tetrabutyl ammonium salt[3] ........ 80
7.1.1 UV-Vis and CD spectroscopy ........ 81
7.1.2 $^1$H NMR titration ........ 82
7.1.3 ESI-MS spectra ........ 84
7.2 Dipotassium (R)-1,1'-binaphthyl-2,2'-disulfonate ($BINSO_3$) ........ 88
7.2.1 UV-Vis and CD spectroscopy ........ 89
7.2.2 $^1$H NMR titration ........ 90
7.2.3 ESI-MS Spectrometry ........ 94
7.2.4 Competition experiments with $[Pd_2(MB\text{-}Q)_4]$ and $[Pd_2(RE\text{-}P)_4]$ on (*R*)-$BINSO_3$ ........ 98
7.2.5 Ion Mobility Measurements ........ 99

# 1 Ligands used in this study

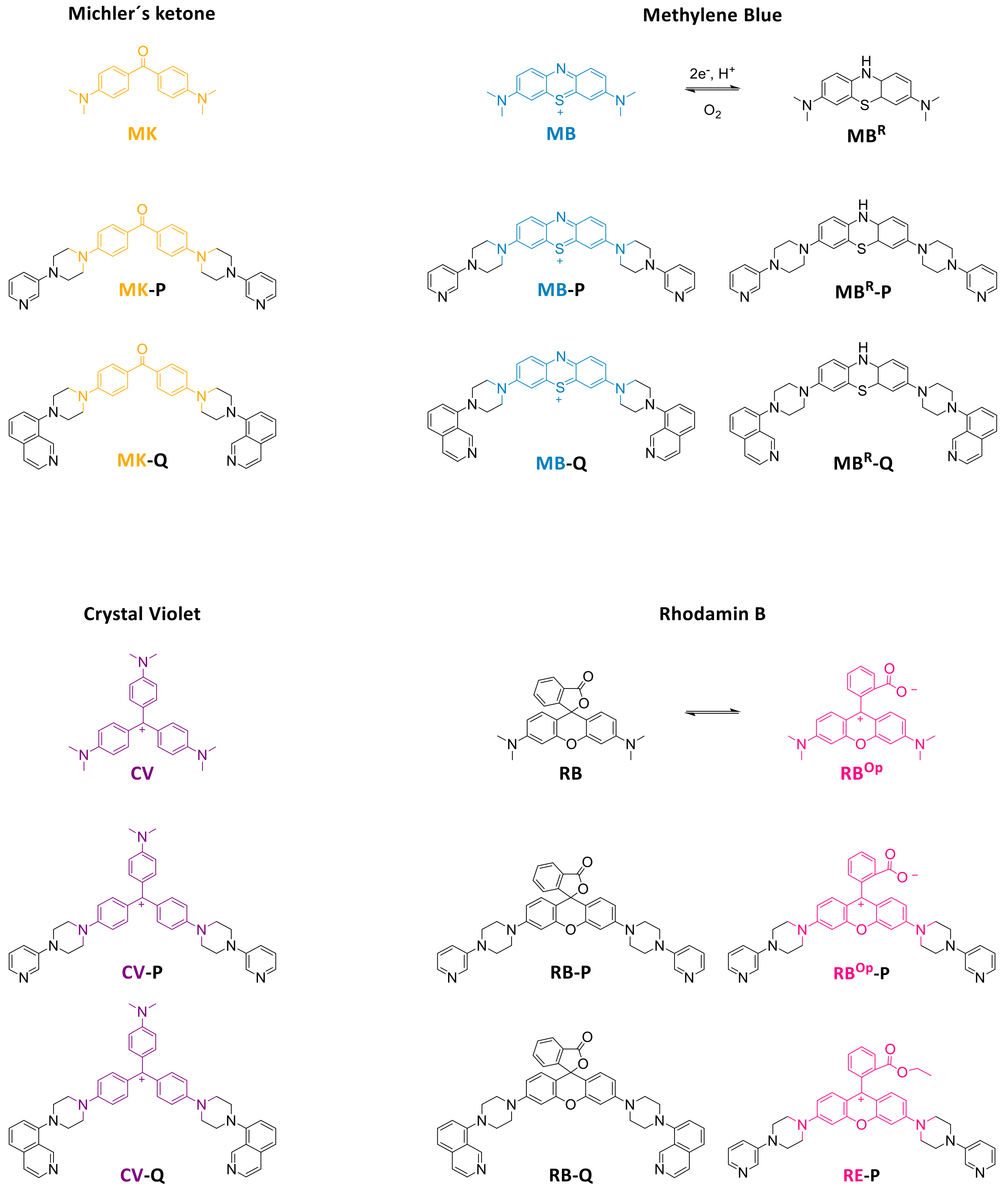


**Figure S1:** Ligands used in this study.

# 2 General Methods

## 2.1 NMR

NMR spectroscopic data was measured on the spectrometers Bruker AV 500 Avance NEO and AV 600 Avance III HD. For $^{1}H$ and $^{13}C$ NMR spectra, chemical shifts were calibrated to the solvent lock signal. Chemical shifts δ are given in ppm, coupling constants *J* in Hz. The following abbreviations are used to describe signal multiplicity for $^{1}H$ NMR spectra: s: singlet, d: doublet, t: triplet, dd: doublet of doublets; dt: doublet of triplets; m: multiplet, br: broad. All proton and carbon signals were assigned with the aid of 2D NMR spectra. All spectra were recorded in standard 5 mm NMR tubes at the indicated temperature. $^{1}H$ DOSY NMR spectra were recorded with a dstebpgp3s pulse sequence with diffusion delays D20 of 0.06-0.10 s and gradient powers P30 of 800 to 2000 µs. For further details on DOSY acquisition parameters and data elaboration see the dedicated paragraph **4**.

## 2.2 Mass spectrometry and ion mobility measurements

Mass spectrometry and trapped ion mobility data were measured on Bruker ESI-timsTOF (electrospray ionization-trapped ion mobility-time of flight) and Bruker compact high-resolution LC mass spectrometers (positive/negative mode). For calibration of the TIMS and TOF devices, Agilent ESI-Low Concentration Tuning Mix was used. For further details on experimental settings and processing of ion mobility data with CCS calculations, see the dedicated paragraph **7.2.6**.

## 2.3 UV-Vis

UV vis spectra were recorded on a DAD HP-8453 UV-Vis spectrometer. Cuvette path length 0.2 cm, unless otherwise stated; wavelength: 250 nm – 800 nm, step size: 1 nm.

## 2.4 CD

Circular dichroism spectra were recorded in DMSO with an Applied Photophysics Chirascan qCD Spectrometer with a temperature-controlled cuvette holder from the prepared NMR solutions with the proper dilution. The spectra were background-corrected and smoothed with a window size of 5. Cuvette path length 0.2 cm, wavelength: 250 nm – 800 nm, step size: 1 nm, band width: 0.5 nm.

## 2.5 Computational studies

Geometry optimized models of structures [$Pd_2$(**MB-P**)$_4$], [$Pd_2$(**MK-Q**)$_4$] and [$Pd_2$(**MB-Q**)$_4$] were constructed using Wavefunction SPARTAN'18[1] and first optimized on semiempirical PM6 level of theory without constraints. The resulting structures were then further refined by DFT optimization (B3LYP/def2-SV(P)) using GAUSSIAN 16.[2] Cage [$Pd_2$(**MB-P**)$_4$] and helicate [$Pd_2$(**MB-Q**)$_4$] were calculated with one $NO_3^-$ counterion in the center of the cavity in order to compensate the repulsive electrostatic interaction and, for [$Pd_2$(**MB-Q**)$_4$], avoid de-twisting of the ligands around the Pd-axis.

# 3 Experimental Procedures

Where necessary, experiments were performed under nitrogen atmosphere using standard Schlenk techniques. Chemicals and standard solvents were purchased from Sigma Aldrich, Acros Organics, Carl Roth, TCI Europe, VWR, ABCR and used as received, if not mentioned differently. Dry solvents were purchased or purified and dried over absorbent-filled columns on a GS-Systems solvent purification system (SPS). Reactions were monitored with thin layer chromatography (TLC) using silica coated aluminium plates (Merck, silica 60, fluorescence indicator F254, thickness 0.25 mm). For column chromatography, silica (Merck, silica 60, 0.02–0.063 mesh ASTM) was used as the stationary phase. The tetrabutyl ammonium salts of the guest (*R*)-CSA was prepared according to previously reported procedures.[3]

## 3.1 Synthesis of the ligands

### 3.1.1 Synthesis of MK-P

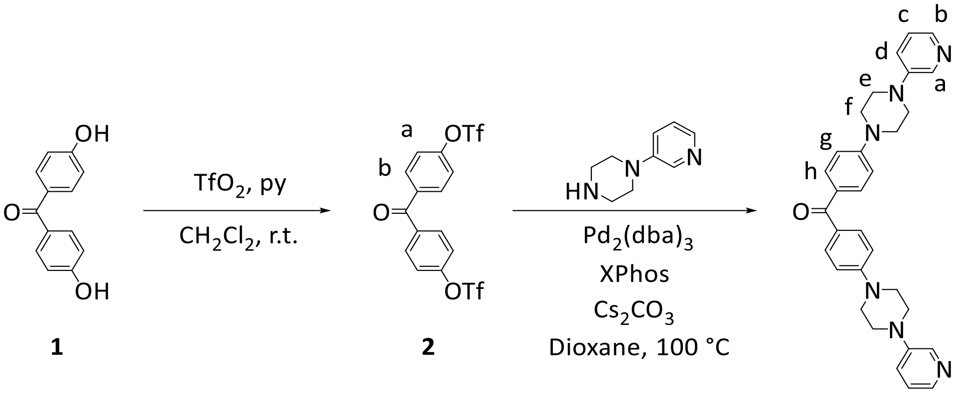


**Scheme S1:** Synthesis of **MK-P**.

#### 3.1.1.1 Synthesis of 4,4-di(trifluoromethanesulfonyl)benzophenone (**2**)

The synthesis of 4,4-di(trifluoromethanesulfonyl)benzophenone was carried out according to a procedure described by Plietzsch and co-workers.[4]

To a solution of 4,4-dihydroxybenzophenone (2.00 g, 9.34 mmol, 1 equiv.) in dry pyridine (25 mL), trifluoromethanesulfonic anhydride (3.4 mL, 5.79 g, 20.5 mmol, 2.2 equiv.) was added dropwise at 0 °C under $N_2$ atmosphere. The reaction mixture was stirred for 14 h and was allowed to warm to room temperature. The reaction was quenched with water (40 mL). The aqueous reaction mixture was extracted with $CH_2Cl_2$ (3 × 40 mL). The combined organic layers were washed with 10% HCl (40 mL) and 1M NaOH solution (40 mL), dried over $MgSO_4$ and the solvent was removed under reduced pressure. The crude product was purified by column chromatography (0-10 % EtOAc/*n*-Hexane solvent gradient). The product was isolated as white solid (3.96 g, 8.28 mmol, 88 %).

$^1$H NMR (500 MHz, 298 K, chloroform-*d*) δ 7.91 (d, *J* = 8.8 MHz 4H, Hb), 7.43 (d, *J* = 8.8 MHz 4H, Ha).

$^{13}$C NMR (126 MHz, 298 K, chloroform-*d*) δ 192.99 (C=O), 152.44 ($C^q$, **C**-Ca), 136.80 ($C^q$, **C**-Cb), 132.29 (Cb), 121.88 (Ca), 118.73 (**C**$F_3$, $J_{C-F}$=321.17 Hz).

HR ESI-MS: measured for $(C_{15}H_8F_6O_7S_2H)^+$: 478.9696

calculated: 478.9688

#### 3.1.1.2 Synthesis of **MK-P**

Compound **2** (100 mg, 210 µmol, 1.0 equiv.) was added together with $Pd_2(dba)_3$ (19.0 mg, 20.9 µmol, 0.1 equiv.), XPhos (30.0 mg, 62.7 µmol, 0.3 equiv.), $Cs_2CO_3$ (190 mg, 585 µmol, 2.8 equiv.) and 1-(pyridin-3-yl)piperazine (75.1 mg, 459.9 µmol, 2.2 equiv.) in dioxane into a Schlenk tube und the mixture was degassed via the *pump- and freeze-method*. The mixture was heated to 100 °C for 18 h. The solvent was evaporated and the reaction mixture was purified by column chromatography (0-5 % $CH_2Cl_2$/MeOH solvent gradient). The product was isolated as a yellow solid (87 mg, 172.4 µmol, 82 %).

$^{1}$H NMR (600 MHz, 298 K, chloroform-*d*) δ 8.31 (s, 1H, Ha), 8.10 (s, 1H, Hb), 7.75 – 7.70 (m, 2H, Hh), 7.20 – 7.12 (m, 2H, Hc, Hd), 6.93 – 6.87 (m, 2H, Hg), 3.49 – 3.45 (m, 4H, Hf), 3.35 – 3.31 (m, 4H, He).

$^{13}$C NMR (151 MHz, 298 K, chloroform-*d*) δ 194.08 (C=O), 153.41 ($C^q$, Cg-**C**), 146.85($C^q$, Cd-**C**-Ca), 141.37 (Cb), 138.92 (Ca), 132.22 (Ch), 129.15 (Ch-**C**), 123.77 (Cd), 122.87 (Cc), 114.00 (Cg), 48.59 (Ce), 47.77 (Cf).

HR ESI-MS: measured for ($C_{31}H_{32}N_6OH^+$): 505.2710

calculated: 505.2706

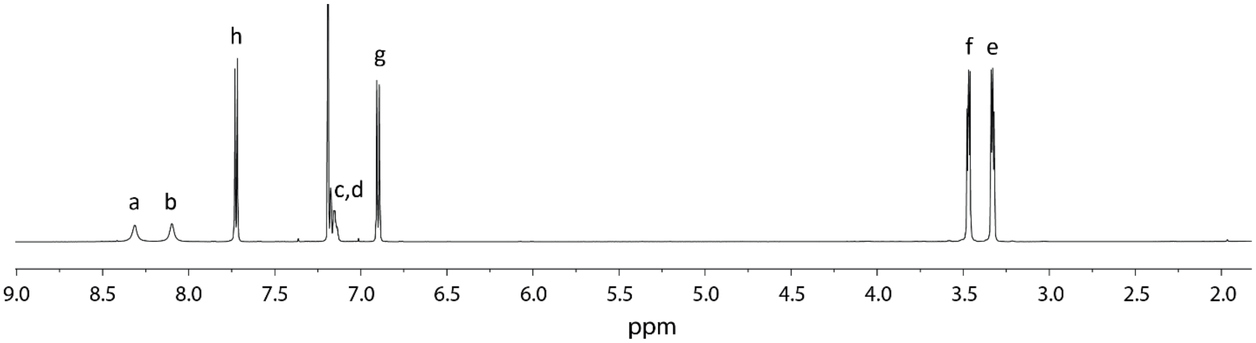


**Figure S2:** $^{1}$H NMR spectrum (600 MHz, 298K, chloroform-*d*) of ligand **MK-P**.

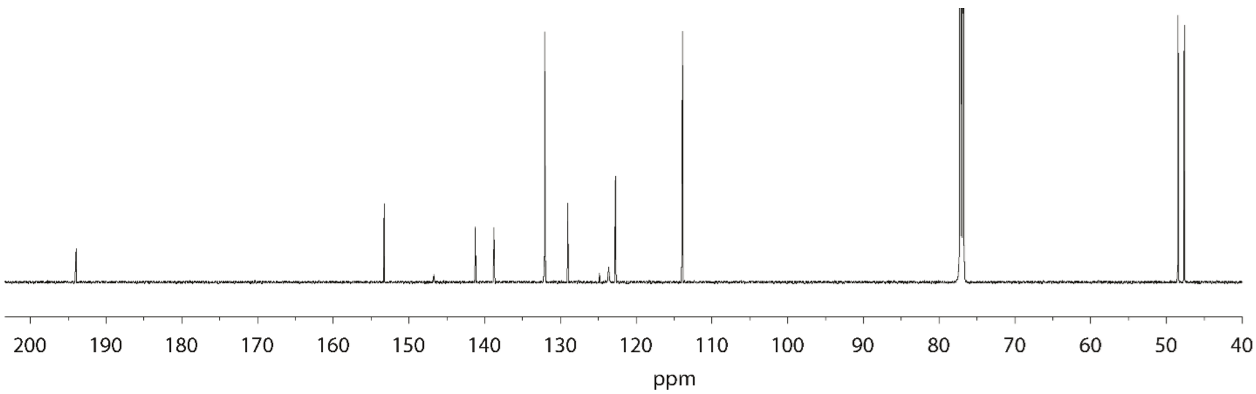


**Figure S3:** $^{13}$C NMR spectrum (151 MHz, 298K, chloroform-*d*) of ligand **MK-P**.

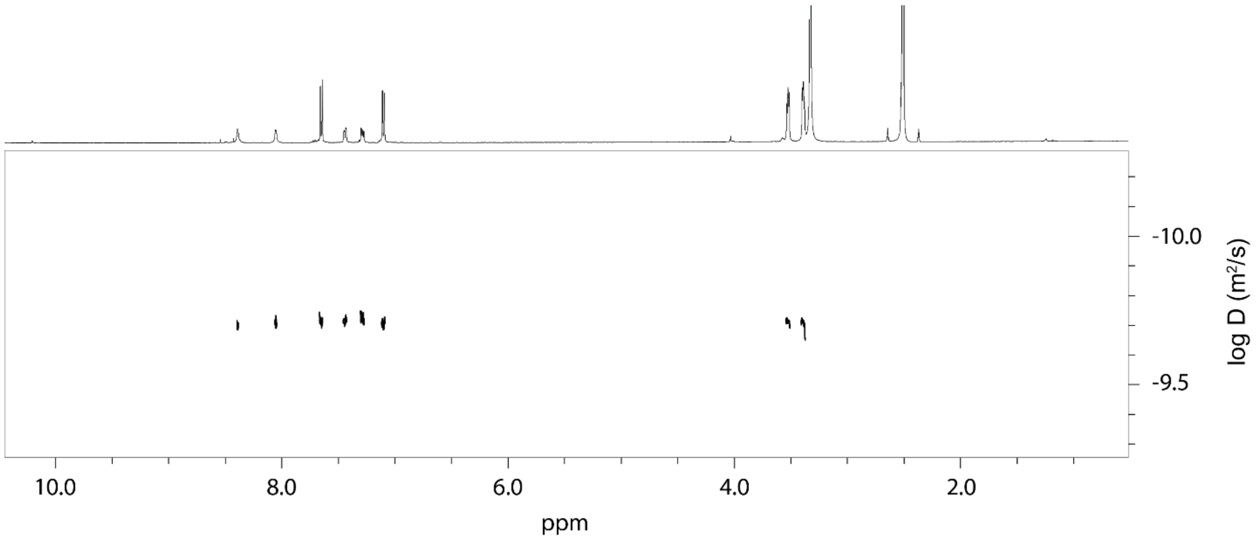


**Figure S4:** $^{1}H$ DOSY spectrum (500 MHz, 298K, DMSO-$d_6$) of **MK-P** (2.8 mM). Diffusion coefficient: $1.902\times10^{-10}$ $m^2s^{-1}$, log D=-9.721. Hydrodynamic radius= 5.77 Å.

### 3.1.2 Synthesis of RB-P

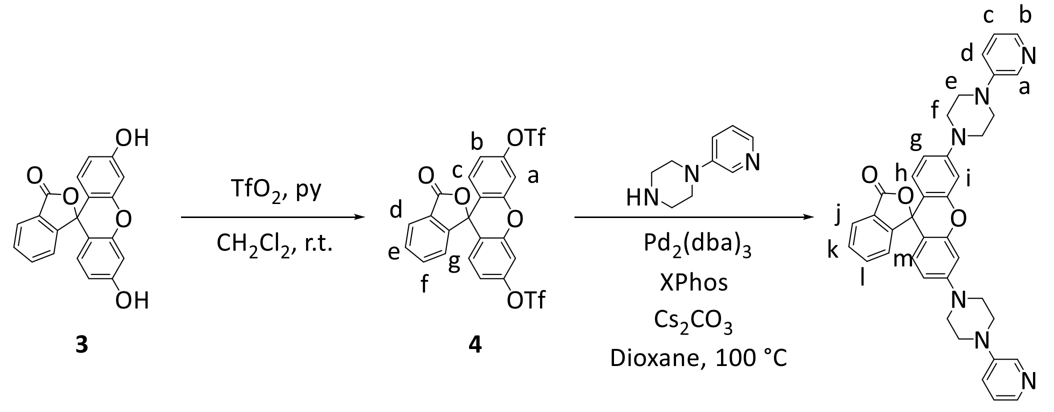


**Scheme S2:** Synthesis of **RB-P.**

3.1.2.1 Synthesis of 3-oxo-3H-spiro[isobenzofuran-1,9'-xanthene]-3',6'-diyl bis(trifluoromethanesulfonate) (**4**)
The synthesis was carried out according to a procedure described by Grimm and co-workers.[5]

Fluorescein (2.50 g, 7.52 mmol, 1.0 equiv.) was dissolved in 30 mL of $CH_2Cl_2$ and the solution was degassed via the *pump- and freeze-method*. The solution was cooled to 0 °C with an ice bath. Pyridine (4.76 g, 4.87 mL, 60.2 mmol, 8.0 equiv.) and trifluoromethanesulfonic anhydride (8.49 g, 5.06 mL, 30.1 mmol, 4.0 equiv.) were added. The ice bath was removed and the reaction mixture was stirred for 21 h at room temperature. The reaction mixture was mixed with water (30 mL) and the product was extracted with $CH_2Cl_2$ (2 x 30 mL). The organic extracts were collected, washed with NaCl (each 30 mL) and dried over $MgSO_4$. The solvent was removed and the crude product was purified by column chromatography (0-30 % EtOAc/*n*-Hexane solvent gradient). The product was isolated as yellow solid (3.73 g, 6.26 mmol, 83 %).

$^{1}$H NMR (300 MHz, 298 K, chloroform-*d*) δ = 8.11 – 8.04 (m, 1H, Hd), 7.78 – 7.65 (m, 2H, He, Hf), 7.30 (d, *J* = 2.4 Hz, 2H, Ha), 7.22 – 7.15 (m, 1H, Hg), 7.03 (dd, *J* = 8.8 Hz, 2.4 Hz, 2H, Hb), 6.96 (d, *J* = 8.8 Hz, 2H, Hc).

$^{13}$C NMR (75 MHz, 298 K, chloroform-*d*) *δ* 168.57(**C**=O), 152.32 ($C^q$, **C**-Cd) 151.48 ($C^q$, **C**-Ca), 150.38 ($C^q$, **C**-Cc), 135.94 (Ce), 130.83 (Cf), 130.11 (Cc), 125.87 (Cd), 125.78 ($C^q$, **C**-Cg), 123.89 (Cg), 119.51 ($C^q$, Ca-**C**-Cb), 118.71 (**C**$F_3$, $J_{C\text{-}F}$=119.52 Hz), 117.52(Cb), 110.84 (Ca), 80.22(spiro $C^q$).

HR ESI-MS: measured for ($C_{22}H_{10}F_6O_9S_2H^+$): 596.9742

calculated: 596.9743

### 3.1.2.2 Synthesis of **RB-P**

Compound **4** (280 mg, 470 µmol, 1.0 equiv.) was added together with $Pd_2(dba)_3$ (86 mg, 94 µmol, 0.2 equiv.), XPhos (67 mg, 141 µmol, 0.3 equiv.), $Cs_2CO_3$ (429 mg, 1.31 mmol, 2.8 equiv.) and 1-(pyridin-3-yl)piperazine (168.6 mg, 1.03 mmol, 2.2 equiv.) in dioxane into a Schlenk tube und the mixture was degassed via *the pump-and freeze-method*. The mixture was heated to 100 °C for 18 h. The solvent was evaporated and the reaction mixture was purified by column chromatography (0-10 % MeOH /$CH_2Cl_2$). The product was isolated as a light pink solid (230 mg, 369 µmol, 78 %).

$^{1}$H NMR (600 MHz, 298 K, acetonitrile–$d_3$) δ 8.34 (d, *J* = 3.0 Hz, 2H, Ha), 8.05 (dd, *J* = 4.6, 1.1 Hz, 2H, Hb), 7.98 (dt, *J* = 7.7, 1.0 Hz, 1H, Hm), 7.74 (td, *J* = 7.5, 1.2 Hz, 1H, Hk), 7.68 (td, *J* = 7.5, 1.0 Hz, 1H, Hl), 7.32 (ddd, *J* = 8.6, 3.1, 1.4 Hz, 2H, Hd), 7.24 – 7.15 (m, 3H, Hc, Hj), 6.80 (d, *J* = 2.5 Hz, 2H, Hi), 6.74 (dd, *J* = 8.9, 2.5 Hz, 2H, Hg), 6.66 (d, *J* = 8.9 Hz, 2H, Hh), 3.45-3.39 (m, 8H, Hf), 3.39-3-34 (m, 8H, He).

$^{13}$C NMR (151 MHz, 298 K, acetonitrile–$d_3$) δ 169.79 (C=O), 153.60 ($C^q$, Cg-**C**-Ci), 153.54 ($C^q$, **C**-Cm), 153.07 ($C^q$, Cg-**C**-Ci), 147.52($C^q$, Ca-**C**-Cd), 141.09 (Cb), 139.04 (Ca), 135.79 (Cl), 130.37 (Ck), 129.26 (Ch), 127.48 ($C^q$, **C**-Cj), 125.16 (Cj), 124.41 (Cm), 124.08 (Cc), 122.91 (Cd), 112.49 (Cg), 109.88 ($C^q$, C-Ch), 102.21 (Ci), 84.15 (spiro $C^q$), 48.55 (Ce), 48.24 (Cf).

HR ESI-MS: measured for ($C_{38}H_{34}N_6O_3H^+$): 623.2762

calculated: 623.2765

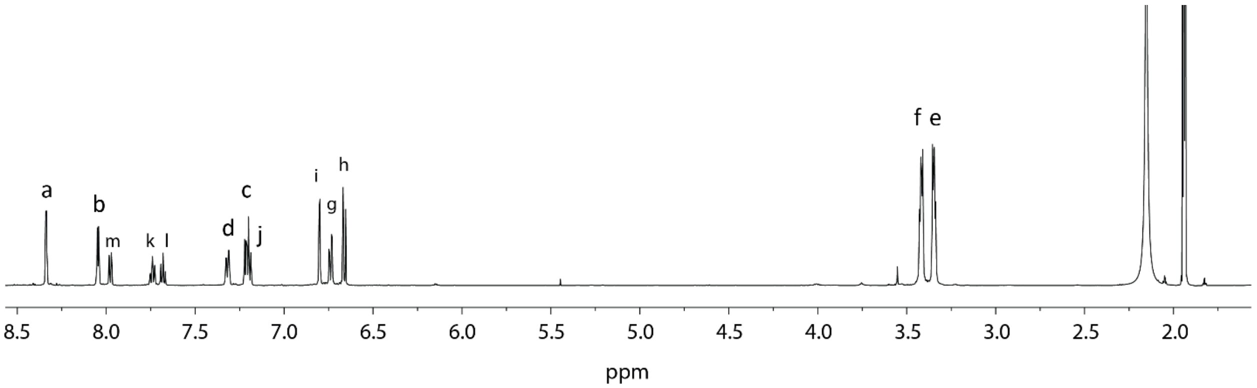


**Figure S5:** $^{1}$H NMR spectrum (600 MHz, 298K, $CD_3CN$) of ligand **RB-P.**

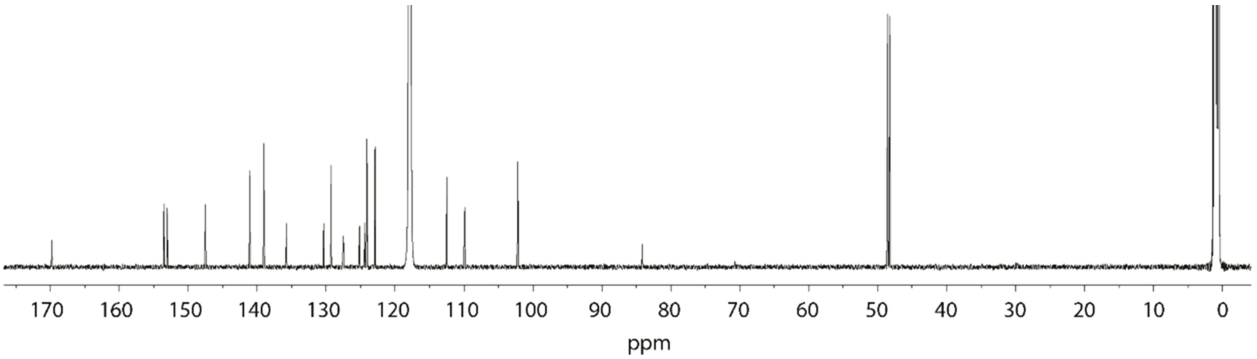


**Figure S6:** $^{13}C$ NMR spectrum (151 MHz, 298K, $CD_3CN$) of ligand **RB-P.**

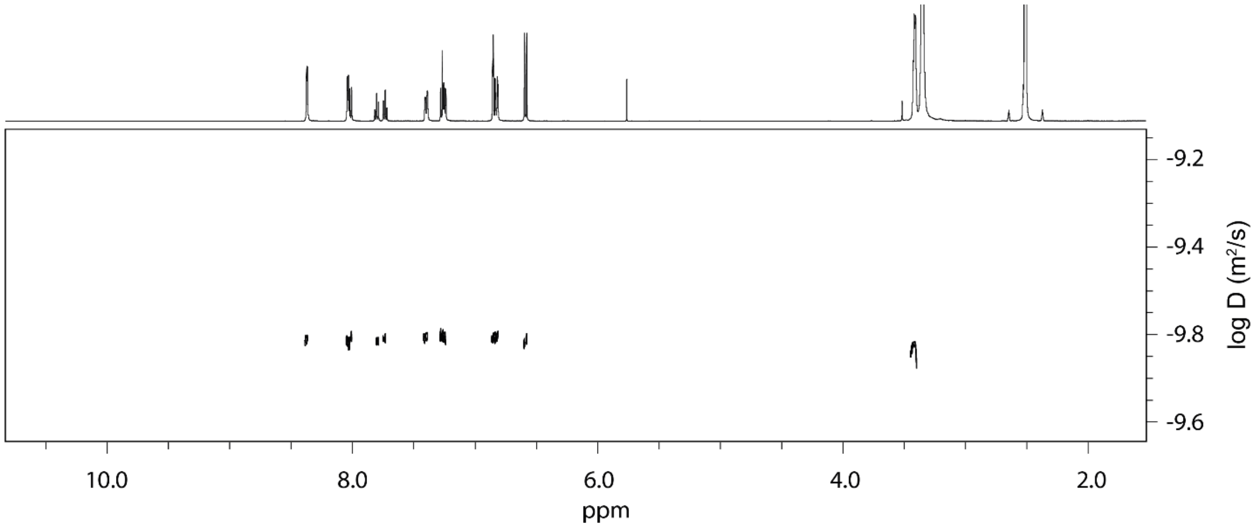


**Figure S7:** $^1H$ DOSY spectrum (500 MHz, 298K, DMSO-$d_6$) of **RB-P** (2.8 mM). Diffusion coefficient: $1.667\times10^{-10}$ $m^2s^{-1}$, log D=-9.778. Hydrodynamic radius= 6.59 Å.

### 3.1.3 Synthesis of MB-PBF$_4$ and MB-PNO$_3$

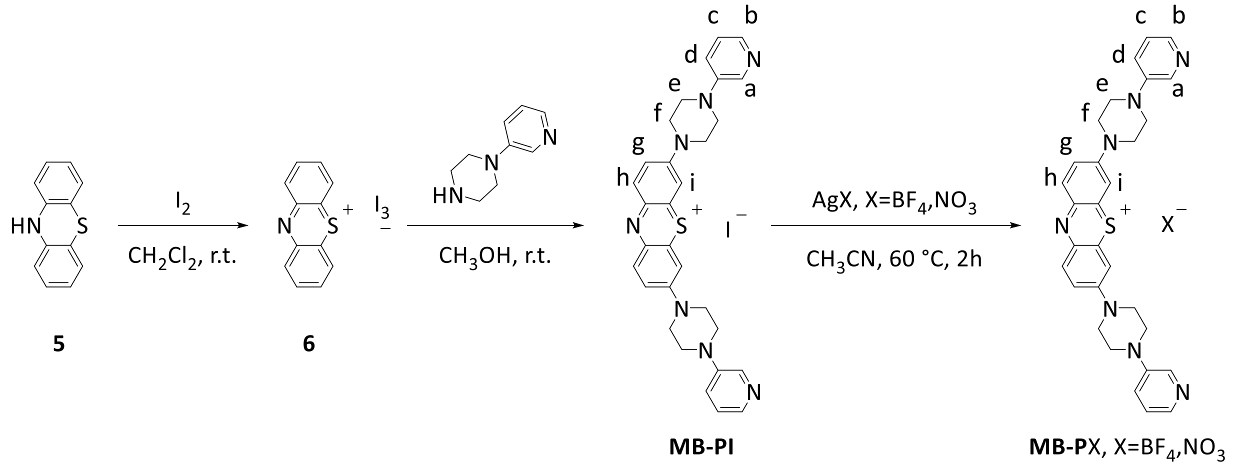


**Scheme S3:** Synthesis of ligands **MB-P**BF$_4$ and **MB-P**NO$_3$.

The synthesis of ligands **MB-P**BF$_4$ and **MB-P**NO$_3$ followed a procedure reported by us, recently.[6]

### 3.1.3.1 Oxidation of 10H-phenothiazine (**6**)

10H-Phenothiazine (2 g, 10 mmol) was dissolved in 50 mL of $CH_2Cl_2$ at room temperature. A solution of iodine (8 g, 30 mmol) in $CH_2Cl_2$ (150 mL) was added, and the whole mixture stirred for 10 h at room temperature. The resulting purple-black solid was filtered and washed free of iodine by using large amounts of $CH_2Cl_2$. Yield: 5.00 g. The compound was directly used in the next step.

### 3.1.3.2 Synthesis of **MB-P**I

To a solution of the above-mentioned black powder **6** (1 mmol, 0.706 g, 1 equiv.) in methanol (15 mL) a methanol (15 mL) solution of 1–(3–pyridinyl)piperazine (1.63 g, 10 mmol, 10 equiv.) was added drop-wise. The reaction was carried out under stirring at room temperature. When the reaction was complete (15 h, TLC control), the solution was evaporated to dryness under vacuum and purified by flash column chromatography (Rf=0.4, $CH_2Cl_2$ : MeOH 95 : 5). After collection and evaporation, the compound was purified by reprecipitation using $CH_3OH$/EtOAc and the desired product was obtained as a dark blue powder (180 mg).

$^{1}$H NMR (500 MHz, 298 K, acetonitrile–*d*$_3$) δ 8.31 (d, *J* = 2.5 Hz, 2H, Ha), 8.07 (d, *J* = 3.4 Hz,2H, Hb), 8.00 (d, *J*=9.6 Hz, 2H, Hh), 7.57 (dd, *J*=9.8, 2.5 Hz, 2H, Hg), 7.46 (d, *J*=2.4 Hz, 2H, Hi), 7.36 (d, *J*=7.6 Hz, 2H, Hd), 7.29 (dd, *J*=8.3, 4.6 Hz, 2H, Hc), 3.96–4.02 (m, 8H, Hf), 3.57–3.43 (m, 8H, He).

$^{13}$C NMR (126 MHz, 298 K, acetonitrile–*d*$_3$) δ 146.65 (C$^q$, Ca-**C**-Cd), 140.44 (Cb), 139.28 (Ch), 137.65 (C$^q$, **C**-Ch), 137.08 (Ca), 135.82 (C$^q$, **C**-Ci), 124.38 (Cc), 122.81 (Cd), 119.87 (Cg), 107.39 (Ci), 47.65 (Ce,Cf). C$^q$, Ci-**C**-Cg not found.

| | | |
|---|---|---|
| HR ESI-MS: | measured for ($C_{30}H_{30}N_7S^+$): | 520.2254 |
| | calculated: | 520.2278 |

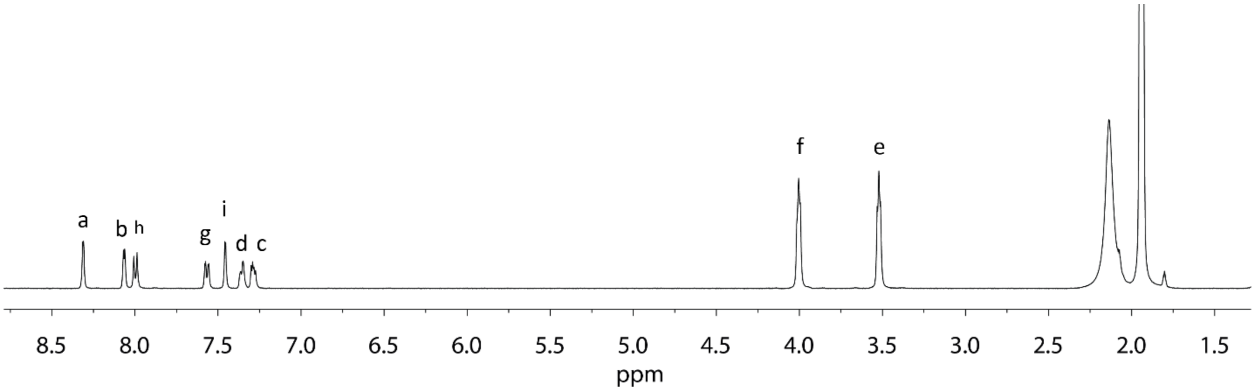


**Figure S8:** $^{1}$H NMR spectrum (500 MHz, 298K, $CD_3CN$) of **MB-P**I.

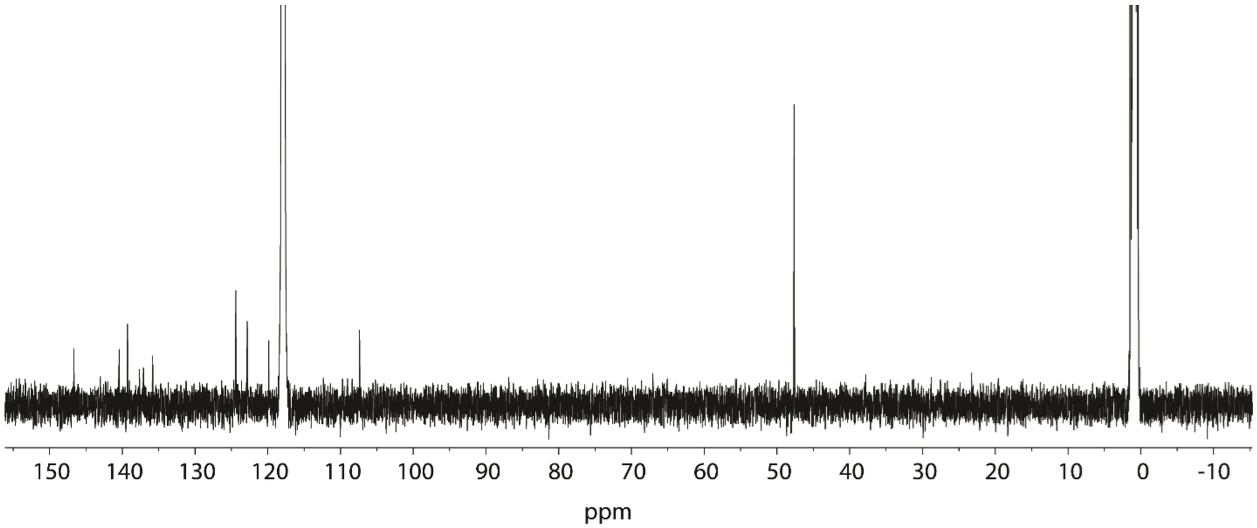


**Figure S9:** $^{13}C$ NMR spectrum (126 MHz, 298K, $CD_3CN$) of **MB-P**I.

3.1.3.3 Synthesis of **MB-P**$BF_4$ and **MB-P**$NO_3$.

To an acetonitrile (10 mL) solution of **MB-P**I (64.76 mg, 0.1 mmol, 1 equiv.) was added the silver salt of the desired counterion ($AgBF_4$ or $AgNO_3$) (0.11 mmol, 1.1 equiv.). The reaction was carried out under stirring at 60 °C for 2 h. After cooling down to room temperature, the resulting solution was filtered and washed by using acetonitrile to get the filtrate. The solvent was evaporated to obtain the product in a quantitative yield.

**MB-P**$BF_4$

$^1H$ NMR (600 MHz, 298 K, acetonitrile–$d_3$) δ 8.29 (d, $J$ = 2.8 Hz, 1H, Ha), 8.06 (dd, $J$ = 4.6,1.1 Hz, 1H, Hb), 8.00 (d, $J$ = 9.6 Hz, 1H, Hh), 7.56 (dd, $J$ = 9.7, 2.8 Hz, 1H, Hg), 7.44 (d, $J$ = 2.8 Hz, 1H, Hi),7.43–7.38 (m, 1H, Hd), 7.33 (dd, $J$ = 8.5, 4.7 Hz, 1H, Hc), 4.04–3.96 (m, 4H, Hf), 3.58–3.49 (m, 4H, He).

$^{13}C$ NMR (151 MHz, 298 K, acetonitrile–$d_3$) δ 154.53 ($C^q$, Ci-**C**-Cg), 147.19 ($C^q$, Ce-**C**-Cd), 139.66 (Cb), 139.62 (Ch), 137.44 ($C^q$, **C**-Ch), 136.70 (Ca), 136.18 ($C^q$, **C**-Ci), 125.14 (Cc), 123.91 (Cd), 120.21 (Cg), 107.70 (Ci), 47.89 (Ce), 47.74 (Cf).

| | | |
|---|---|---|
| HR ESI-MS: | measured for ($C_{30}H_{30}N_7S^+$): | 520.2256 |
| | calculated: | 520.2278 |

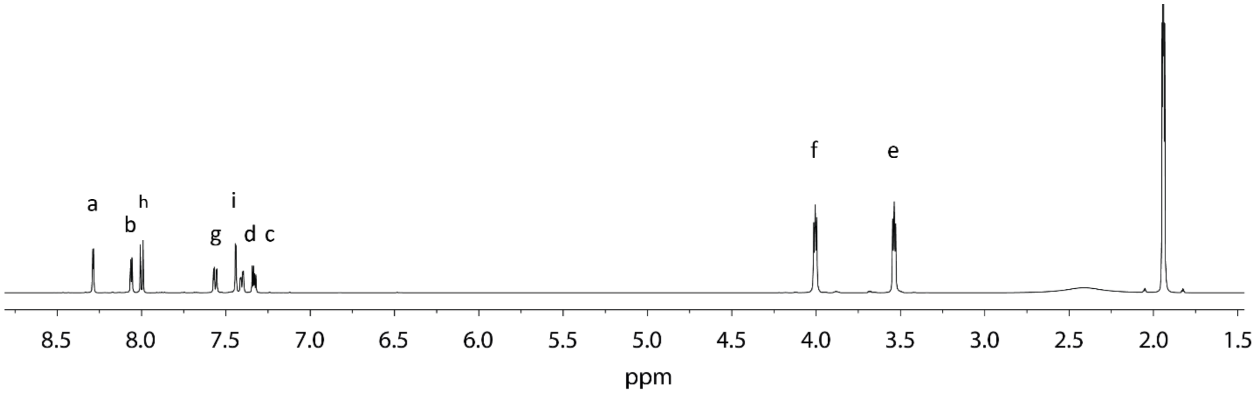


**Figure S10:** $^1H$ NMR spectrum (600 MHz, 298K, $CD_3CN$) of ligand **MB-P**$BF_4$.

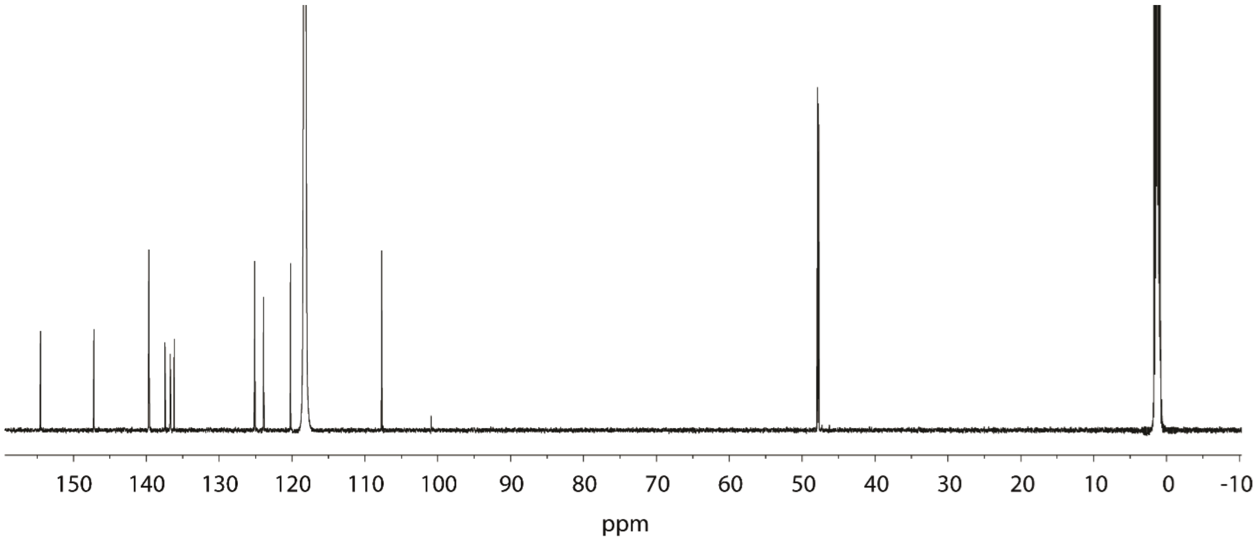


**Figure S11:** $^{13}C$ NMR spectrum (151 MHz, 298K, $CD_3CN$) of ligand **MB-P**$BF_4$.

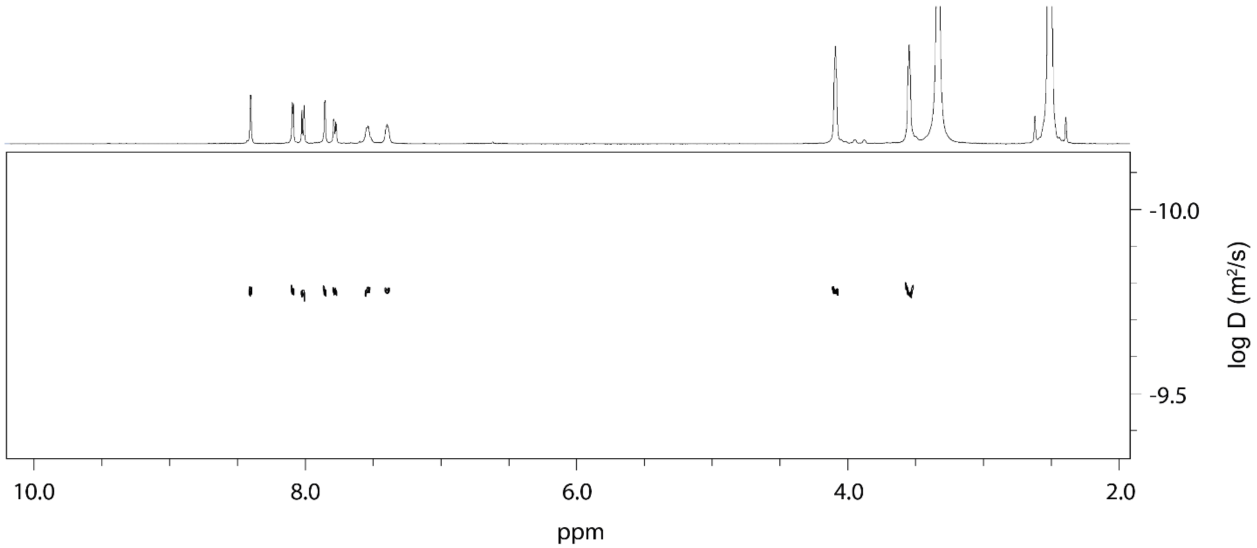


**Figure S12:** $^{1}H$ DOSY spectrum (500 MHz, 298K, DMSO-$d_6$) of **MB-P**$BF_4$ (2.8 mM). Diffusion coefficient: $1.625\times10^{-10}$ $m^2s^{-1}$, log D=-9.789. Hydrodynamic radius= 6.75 Å.

**MB-P**$NO_3$

$^{1}H$ NMR (600 MHz, 298 K, Methanol-$d_4$) δ 8.33 (s, 1H, Ha), 8.10 – 8.05 (m, 2H, Hb, Hc), 7.72 (dd, *J* = 9.7, 2.8 Hz, 1H, Hg), 7.63 - 7.67 (m, 2H, Hd, Hi), 7.50 (dd, *J* = 8.9, 4.9 Hz, 1H, Hc), 4.12 (t, *J* = 5.4 Hz, 4H, Hf), 3.63 (t, *J* = 5.4 Hz, 4H, He).

$^{13}C$ NMR (151 MHz, 298 K, Methanol-$d_4$) δ 155.16 ($C^q$, Cg-**C**-Ci), 148.40 ($C^q$, Ca-**C**-Cd), 140.05 (Ch), 137.93 (Cb), 136.75 (2 overlapping $C^q$, Ch-**C** and Ci-**C**), 135.06 (Ca) 126.31 (Cd), 125.85 (Cc), 120.49 (Cg), 107.90 (Ci), 48.10 (Ce), 48.02 (Cf).

| | | |
|---|---|---|
| HR ESI-MS: | measured for ($C_{30}H_{30}N_7S^+$): | 520.2338 |
| | calculated: | 520.2278 |

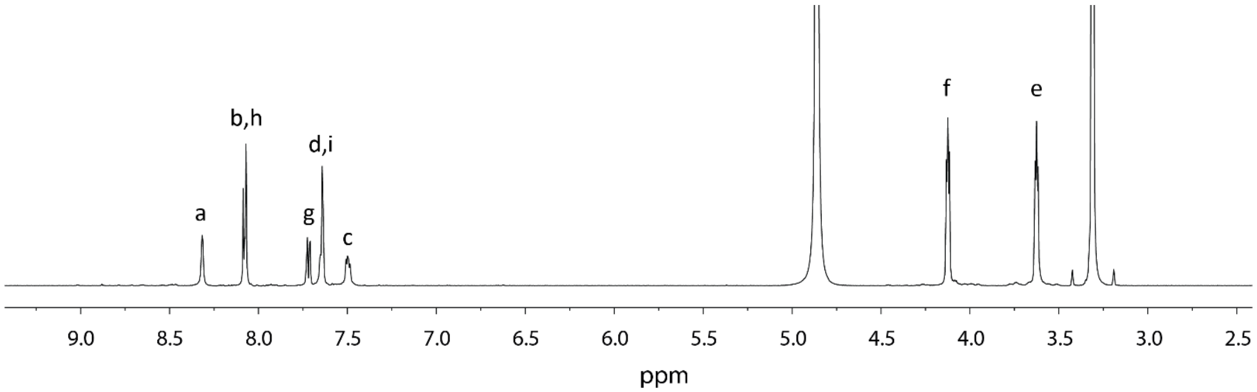


**Figure S13:** $^1$H NMR spectrum (600 MHz, 298K, MeOD) of ligand **MB-P**$NO_3$.

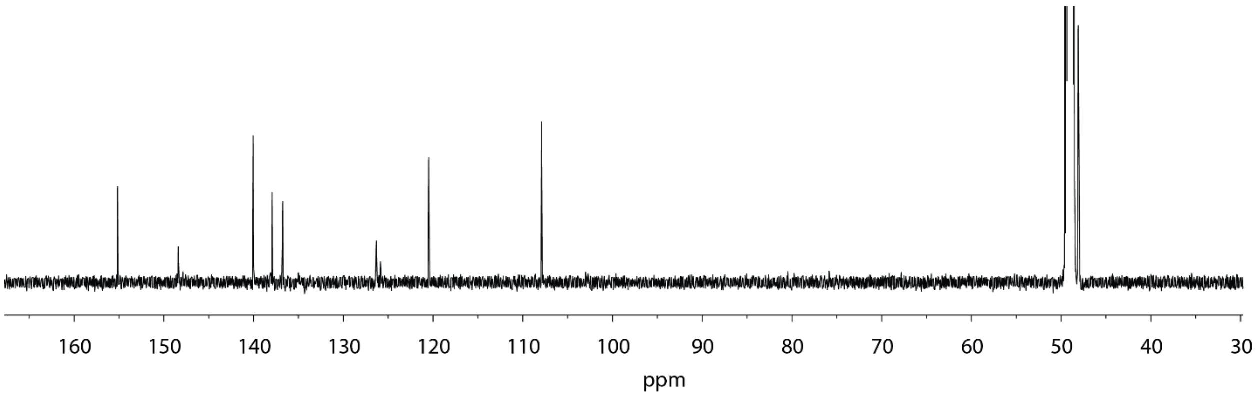


**Figure S14:** $^{13}$C NMR spectrum (151 MHz, 298K, MeOD) of ligand **MB-P**$NO_3$.

### 3.1.4 Synthesis of CV-P

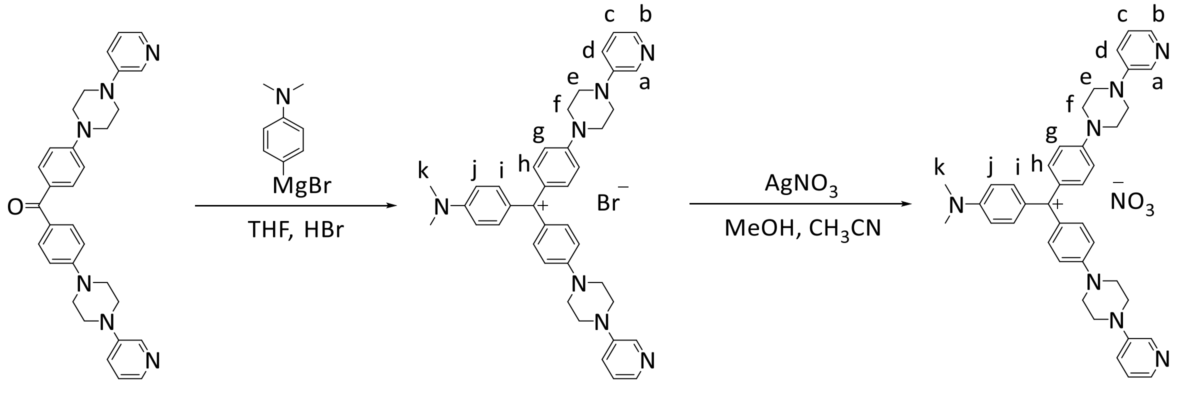


**MK-P** **CV-P**Br **CV-P**

**Scheme S4:** Synthesis of ligand **CV-P**.

### 3.1.4.1 Synthesis of **CV-P**Br

To a round bottom flask equipped with stir bar, Mg (160 mg, 1.3 equiv., 6.60 mmol) was added and heated under vacuum to remove any trace of water. 4-Bromo-N,N-dimethylaniline (1 g, 1 equiv., 5 mmol) was added and the whole system is evacuated for some minutes. To the solids, 10 mL of dry THF were added together with a small iodine crystal. The flask was gently warmed to reflux for 90 minutes. During this time the solution changes from dark to dingy gray. An ice bath was then used to cool down to room temperature the flask.

In a dry round bottom flask **MK-P** (113 mg, 1 equiv., 223 µmol) was added together with 4 mL of dry THF. To the yellow suspension, 1.5 mL of the grey suspension above described are added and the system was put under reflux for 5 minutes and again cooled down to room temperature with an ice bath. Slowly, 4 mL of HBr (10% W/W) are added to obtain the final product. The color of the solution changes from bright yellow to deep violet/blue. The solution was then neutralized with a saturated solution of $NaHCO_3$ and as soon as pH 7 was reached the desired product precipitates as blue flakes. All the volatiles are removed and the product purified by column chromatography (0-10 % MeOH /$CH_2Cl_2$). The product was isolated as a dark violet solid (90 mg, 130 µmol, 58%).

$^1$H NMR (500 MHz, 298 K, Methanol-$d_4$) δ 8.30 (d, J = 3.0 Hz, 1H, Ha), 8.02 (d, J = 4.7 Hz, 1H, Hb), 7.50 (dd, J = 8.5, 2.7 Hz, 1H, Hd), 7.43 (m, 3H, Hi, Hh), 7.35 (dd, J = 8.5, 4.6 Hz, 1H, Hc), 7.23 (d, J = 8.9 Hz, 2H, Hg), 7.04 (d, J = 9.1 Hz, 1H, Hj), 3.87 (t, J = 5.2 Hz, 4H, Hf), 3.66 (s, 3H, Hk), 3.49 (t, J = 5.1 Hz, 4H, He).

$^{13}$C NMR (151 MHz, 298 K, Methanol-$d_4$) δ 179.79 (C+), 158.14 (C$^q$, **C**-Cj), 156.91 (C$^q$, **C**-Cg), 148.50 (C$^q$, Ca-**C**-Cd), 141.63 (Ci), 140.81 (Ch), 140.79(Cb), 138.15 (Ca), 129.36(C$^q$, **C**-Ch), 128.22(C$^q$, **C**-Ci), 125.44 (Cc), 124.47 (Cd), 114.65 (Cg), 114.12 (Cj), 71.57 (Ck), 47.58 (Ce), 47.57 (Cf).

| | | |
|---|---|---|
| HR ESI-MS: | measured for ($C_{39}H_{42}N_7^+$): | 608.3498 |
| | calculated: | 608.3496 |

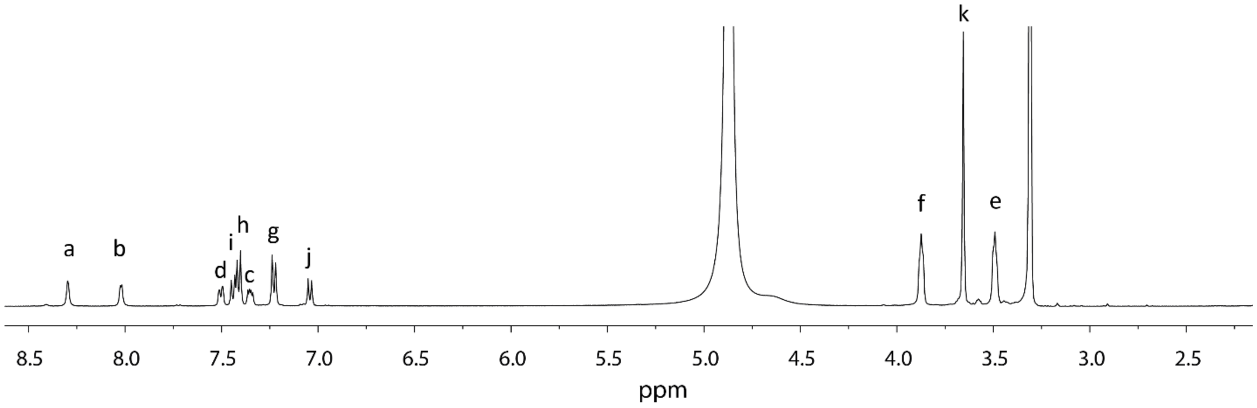


**Figure S15:** $^1$H NMR (500 MHz, 298K, MeOD) spectrum of **CV-P**Br.

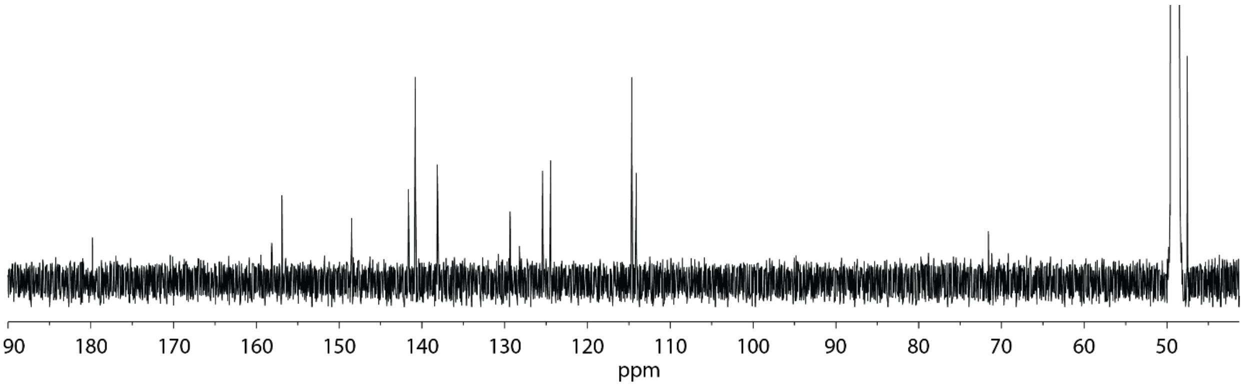


**Figure S16:** $^{13}$C NMR (151 MHz, 298K, MeOD) spectrum of **CV-P**Br.

3.1.4.2 Synthesis of **CV-P**

To a methanol (15 mL) solution of **CV-P**Br (40 mg, 1 equiv., 58 µmol) was added $AgNO_3$ (11 mg, 1.1 equiv., 64 µmol). The reaction was carried out under stirring at 60 °C for 1 h. After cooling down to r.t., the resulting solution was filtered and washed by using methanol to get the filtrate. The solvent was then evaporated to obtain the product (quantitative).

$^{1}$H NMR (500 MHz, 298 K, dimethyl sulfoxide-*d*$_6$) δ 8.38 (s, 1H, Ha), 8.05 (s, 1H, Hb), 7.42 (s, 1H, Hd), 7.39 – 7.21 (m, 5H, Hc, Hh, Hi), 7.05 (d, *J* = 9.0 Hz, 1H, Hj), 3.83 (t, *J* = 5.2 Hz, 4H, Hf), 3.45 (d, *J* = 5.3 Hz, 4H, He), 3.26 (s, 3H, Hk).

$^{1}$H NMR (600 MHz, Methanol-*d*$_4$) δ 8.30 (s, 1H, Ha), 8.03 (d, *J* = 4.2 Hz, 1H, Hb), 7.53 – 7.40 (m, 4H, Hd, Hi, Hh), 7.34 (dd, *J* = 8.5, 4.6 Hz, 1H, Hc), 7.23 (d, *J* = 9.3 Hz, 2H, Hg), 7.05 (d, *J* = 9.3 Hz, 1H, Hj), 3.93 – 3.77 (m, 4H, Hf), 3.54 – 3.45 (m, 4H, He).

$^{13}$C NMR (151 MHz, Methanol-*d*$_4$) δ 179.78 (C+), 158.13 (C$^q$, **C**-Cj), 156.90 (C$^q$, **C**-Cg), 148.49(C$^q$, Ca-**C**-Cd), 141.62 (Cb), 140.80 (Ch), 140.76 (Ci), 138.13 (Ca), 129.35(C$^q$, **C**-Cg), 128.21 (C$^q$, **C**-Ci), 125.44 (Cc), 124.47 (Cd), 114.65 (Cg), 114.11 (Cj), 71.54, 66.91,(Ce under MeOD signal) 47.57 (Cf), 40.72 (Ck).

| | | |
|---|---|---|
| HR ESI-MS: | measured for ($C_{39}H_{42}N_7^+$): | 608.3500 |
| | calculated: | 608.3496 |

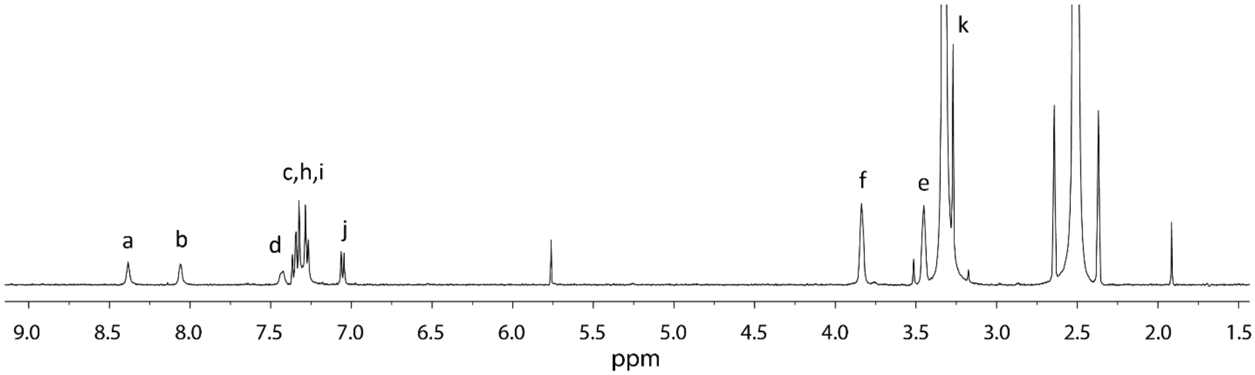


**Figure S17:** $^{1}$H NMR (500 MHz, 298K, DMSO-*d*$_6$) spectrum of **CV-P**.

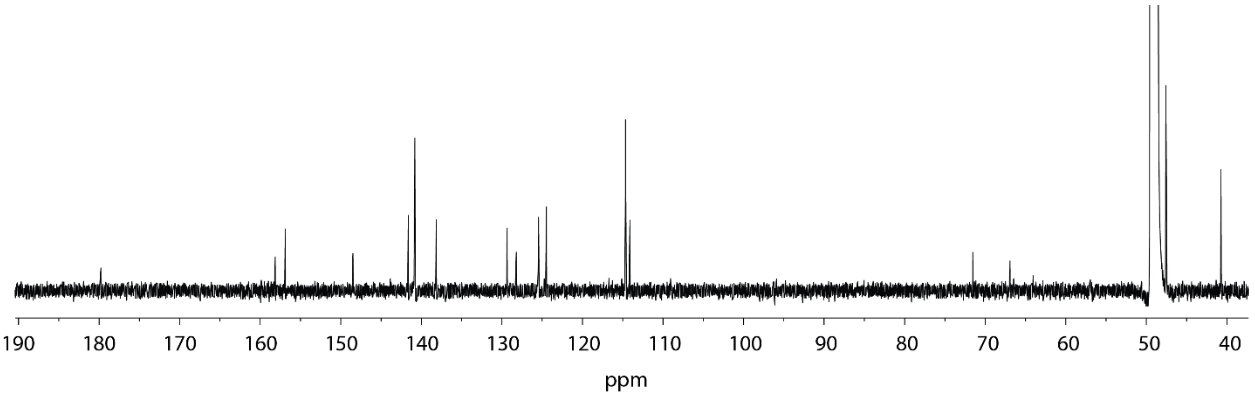


**Figure S18:** $^{13}$C NMR (151 MHz, 298K, MeOD) spectrum of **CV-P**.

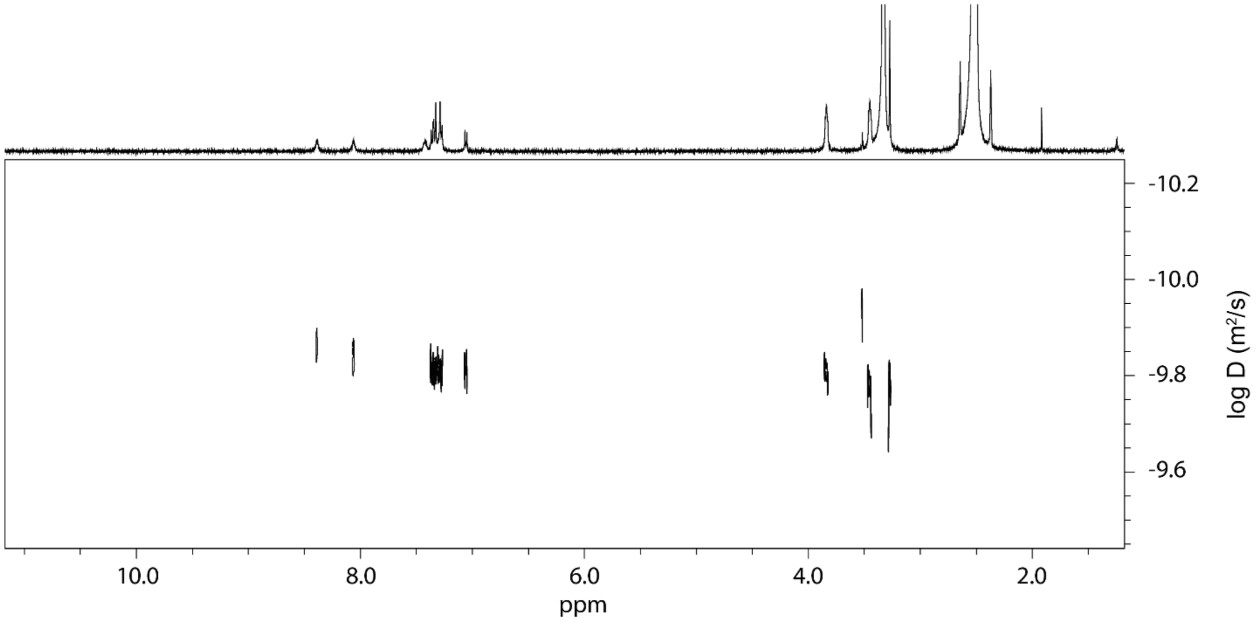


**Figure S19:** $^{1}$H DOSY spectrum (500 MHz, 298K, DMSO-$d_6$) of **CV-P** (2.8 mM). Diffusion coefficient: 1.599×10×10$^{-10}$ m$^{2}$s$^{-1}$, log D=-9.797. Hydrodynamic radius= 6.87 Å.

### 3.1.5 Synthesis of RE-P

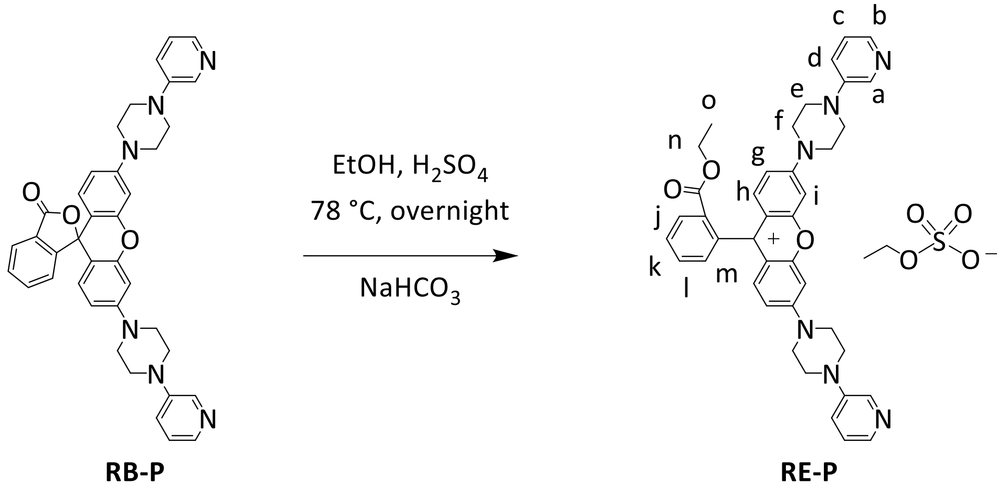


**Scheme S5:** Synthesis of ligand **RE-P**.

Ligand **RB-P** (200 mg, 321 mmol) was dissolved in ethanol (5 mL, 321 mmol). Concentrated sulfuric acid (300 µL) was added, and the mixture was heated at 78°C for 16 h. The mixture was than cool down to r.t. and neutralized with a saturated solution of $NaHCO_3$. The solvents were removed in vacuo and the dark viscous solid was purified with column chromatography on silica gel ($CH_2Cl_2$/MeOH: 95/5) to give ligand **RE-P** (120 mg, 0.184 mmol, 48%). The ligand was further purified with reprecipitation from EtOH/$EtO_2$.

$^1$H NMR (500 MHz, dimethyl sulfoxide-$d_6$) δ 8.35 (d, $J$ = 3.0 Hz, 2H, Ha), 8.27 (dd, $J$ = 7.9, 1.4 Hz, 1H, Hj), 8.04 (dd, $J$ = 4.5, 1.3 Hz, 2H, Hb), 7.93 (td, $J$ = 7.5, 1.4 Hz, 1H, Hl), 7.87 (td, $J$ = 7.7, 1.4 Hz, 1H, Hk), 7.53 (dd, $J$ = 7.6, 1.3 Hz, 1H, Hm), 7.42 – 7.33 (m, 4H, Hd, Hg), 7.31 (d, $J$ = 2.4 Hz, 2H, Hi), 7.26 (dd, $J$ = 8.5, 4.5 Hz, 2H, Hc), 7.08 (d, $J$ = 9.5 Hz, 2H, Hh), 3.96 (m, 10H, Hf, Hn), 3.73 (q, $J$ = 7.1 Hz, 2H, -$CH_2$ counterion), 3.45 (t, $J$ = 5.3 Hz, 8H, He), 1.10 (t, $J$ = 7.1 Hz, 3H, -$CH_3$ counterion), 0.89 (t, $J$ = 7.1 Hz, 3H, Ho).

$^{13}$C NMR (151 MHz, dimethyl sulfoxide-$d_6$) δ 164.69 (0-**C**=0), 158.63 ($C^q$, **C**-Cm), 157.45 ($C^q$, **C**-Ci), 156.48 ($C^q$, **C**-Ch), 145.84 ($C^q$, Ca-**C**-Cd), 139.93 (Cb), 137.41 (Ca), 133.20 (C-Cj), 132.89 (Cl), 131.01 (Ch), 130.89 (C+), 130.65 (Cj), 130.35 (Ck), 129.70 (Cm), 123.61 (Cc), 121.62 (Cd), 115.31 (Cg), 113.83 ($C^q$, **C**-Cg), 97.16 (Ci), 61.16(-**C**$H_2$ counterion), 61.11 (Cn), 46.81 (Cf), 46.36 (Ce), 15.14 (-**C**$H_3$ counterion), 13.45 (Co).

| | | |
|---|---|---|
| HR ESI-MS: | measured for ($C_{40}H_{39}N_6O_3^+$): | 651.3060 |
| | calculated: | 651.3078 |

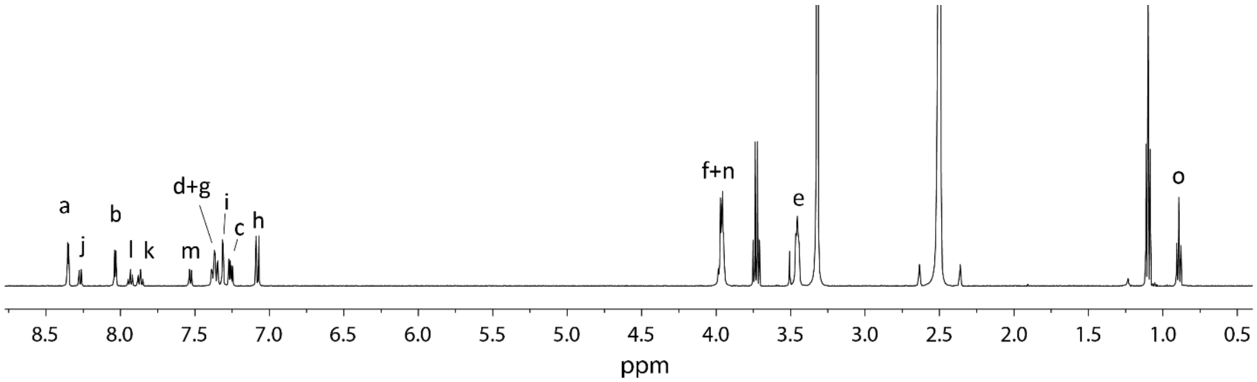


**Figure S20:** $^{1}$H NMR (500 MHz, 298K, DMSO-$d_6$) spectrum of **RE-P**.

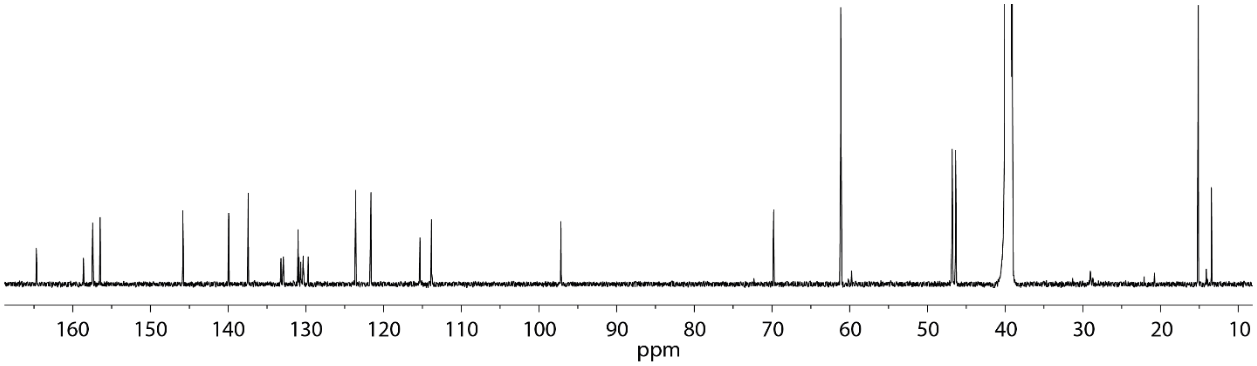


**Figure S21:** $^{13}$C NMR spectrum (151 MHz, 298K, DMSO-$d_6$) of **RE-P**.

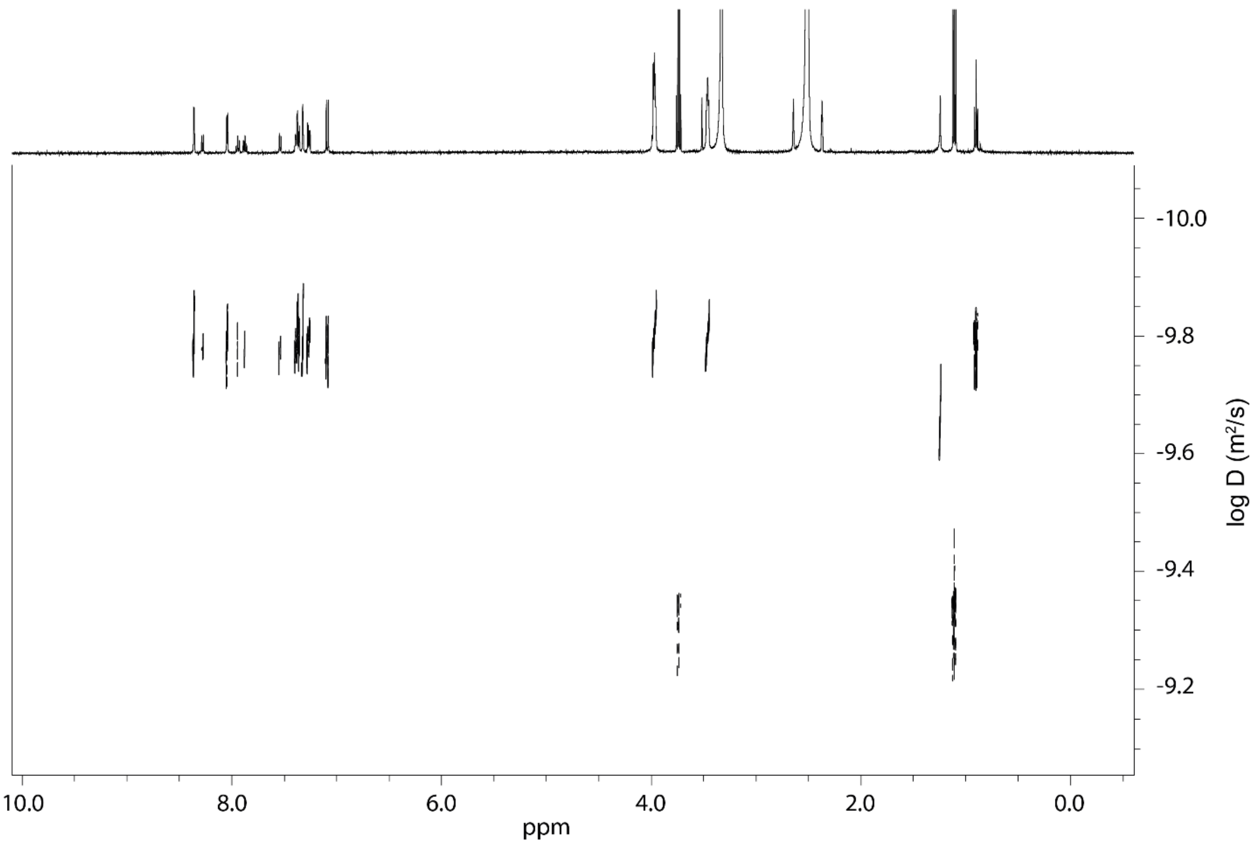


**Figure S22:** $^{1}$H DOSY spectrum (500 MHz, 298K, DMSO-$d_6$) of **RE-P** (2.8 mM). Diffusion coefficient: $1.558\times10^{-10}$ $m^2s^{-1}$, log D=-9. 808. Hydrodynamic radius= 7.05 Å.

### 3.1.6 Synthesis of 8-(piperazin-1-yl)isoquinoline (7)

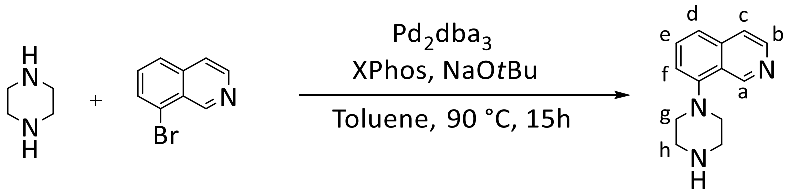


**7**

**Scheme S6:** Synthesis of the linker 8-(piperazin-1-yl)isoquinoline (**7**)

8-Bromoisoquinoline (500 mg, 2.4 mmol, 1 equiv.) was dissolved in toluene (10mL). To the solution was added piperazine (516 mg, 6.0 mmol, 2.5 equiv.), $Pd_2dba_3$ (110 mg, 0.12 mmol, 0.05 equiv.), XPhos (172 mg, 0.36 mmol, 0.15 equiv.), and sodium *t*-butoxide (324 mg, 3.36 mmol, 1.4 equiv.). The mixture was degassed with the *freeze and pump method* and then heated to 90 °C for about 12 hours. The reaction was allowed to cool to room temperature, diluted with diethyl ether and filtered through celite. The filtrate was concentrated, and the dark residue was chromatographed on silica gel ($CH_2Cl_2$/MeOH/EtOAc : 70/20/10) to give about 450 mg of 8-(piperazin-1-yl)isoquinoline (88%).

$^1H$ NMR (500 MHz, 298 K, chloroform-*d*) δ 9.52 (s, 1H, Ha), 8.43 (d, *J* = 5.6 Hz, 1H, Hb), 7.58 – 7.48 (m, 2H, Hc, He), 7.41 (d, *J* = 8.2 Hz, 1H, Hd), 7.06 (dd, *J*=7.5, 0.6 Hz, 1H, Hf), 3.11 (s, 8H).

$^{13}C$ NMR (126 MHz, 298 K, chloroform-*d*) δ 151.06 ($C^q$, **C**-Cc), 149.13 (Ca), 143.00 (Cb), 137.41 ($C^q$, **C**-Ca), 130.72 (Ce), 123.64 ($C^q$, **C**-Cf), 121.44 (Cd), 120.69 (Cc), 115.58 (Cf), 54.56 (Cg), 46.32 (Ch).

HR ESI-MS: measured for ($C_{13}H_{15}N_3H^+$): 214.1330

calculated: 214.1339

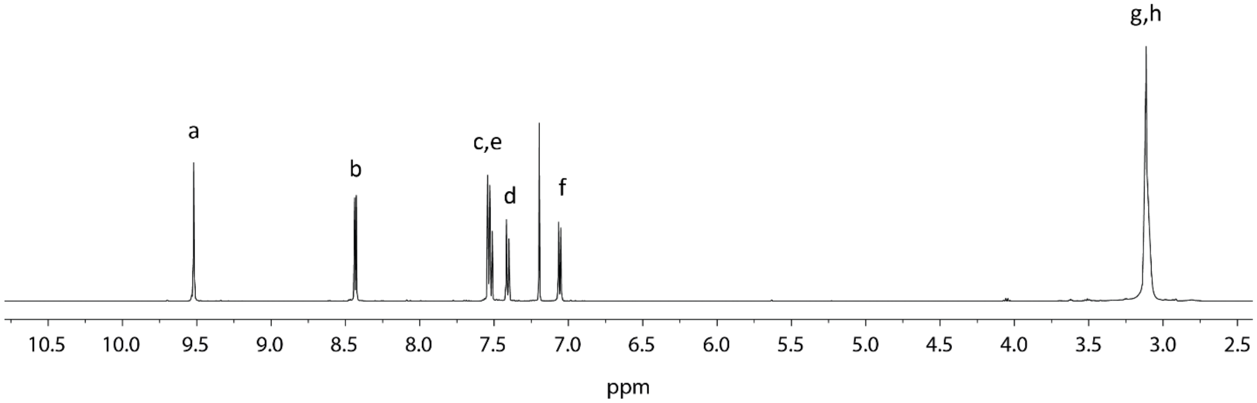


**Figure S23:** $^1H$ NMR spectrum (600 MHz, 298K, $CDCl_3$) of 8-(piperazin-1-yl)isoquinoline.

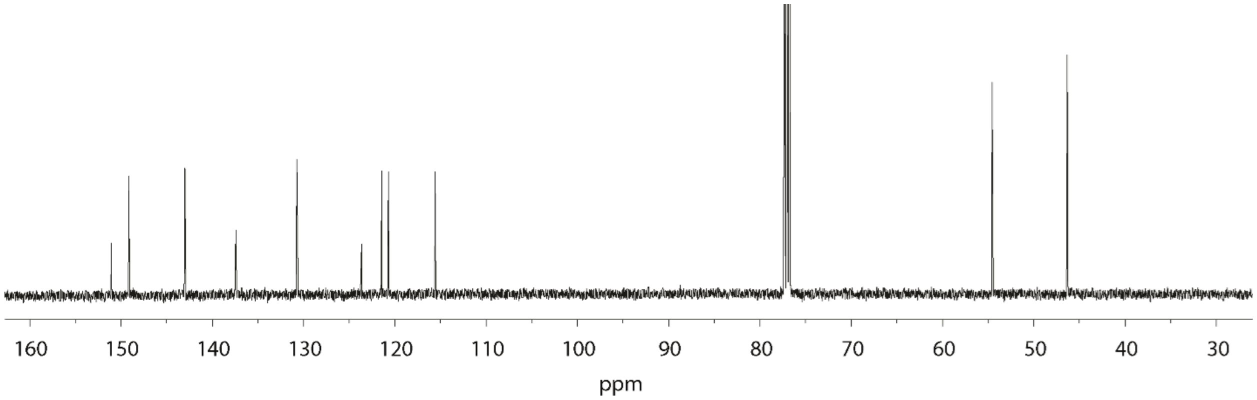


**Figure S24:** $^{13}C$ NMR spectrum (151 MHz, 298K, $CDCl_3$) of 8-(piperazin-1-yl)isoquinoline.

## 3.1.7 Synthesis of MK-Q

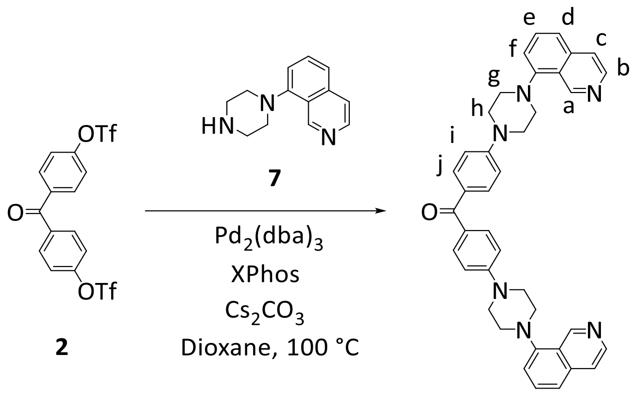


**Scheme S7:** Synthesis of ligand **MK-Q**.

### 3.1.7.1 Synthesis of **MK-Q**

Compound **2** (500 mg, 1.52 mmol, 1.0 equiv.) was added together with $Pd_2(dba)_3$ (278 mg, 0.3 mmol, 0.2 equiv.), XPhos (217 mg, 0.45 mmol, 0.3 equiv.), $Cs_2CO_3$ (1.39 g, 4.25 mmol, 2.8 equiv.) and **7** (712.5 mg, 3.34 mmol, 2.2 equiv.) in dioxane into a Schlenk tube und the mixture was degassed via the *freeze and pump-method*. The mixture was heated to 100 °C for 18 h. The solvent was evaporated and the reaction mixture was purified by column chromatography (0-5 % $CH_2Cl_2$/MeOH). The product was isolated as a yellow solid (625 mg, 1.03 mmol, 68 %).

$^1$H NMR (500 MHz, 298 K, dimethyl sulfoxide-$d_6$) δ 9.56 (s, 1H, Ha), 8.52 (d, *J* = 5.6 Hz, 1H, Hb), 7.81 (dd, *J* = 5.6, 1.0 Hz, 1H, Hc), 7.77 – 7.55 (m, 4H, He, Hd, Hj), 7.30 (dd, *J* = 7.6, 1.1 Hz, 1H, Hf), 7.15 (d, *J* = 9.0 Hz, 2H, Hi), 3.75 – 3.59 (t, *J* = 5.0 Hz, 4H, Hh), 3.27 (t, *J* = 5.0 Hz, 4H, Hg).

$^{13}$C NMR (176 MHz, 298 K, dimethyl sulfoxide-$d_6$) δ 192.41 (C=O), 153.24 ($C^q$, **C**-Cj), 149.77 ($C^q$, **C**-Cc), 148.32 (Ca), 142.96 (Cb), 136.85 ($C^q$, **C**-Ca), 131.49 (Cj), 130.95 (Ce), 127.49 ($C^q$, **C**-Ci), 122.78 ($C^q$, **C**-Cf), 121.57 (Cd), 120.66 (Cc), 115.95 (Cf), 113.45 (Ci), 52.78 (Cg), 47.17 (Ch).

HR ESI-MS: measured for ($C_{39}H_{36}N_6OH^+$): 605.3021

calculated: 605.3023

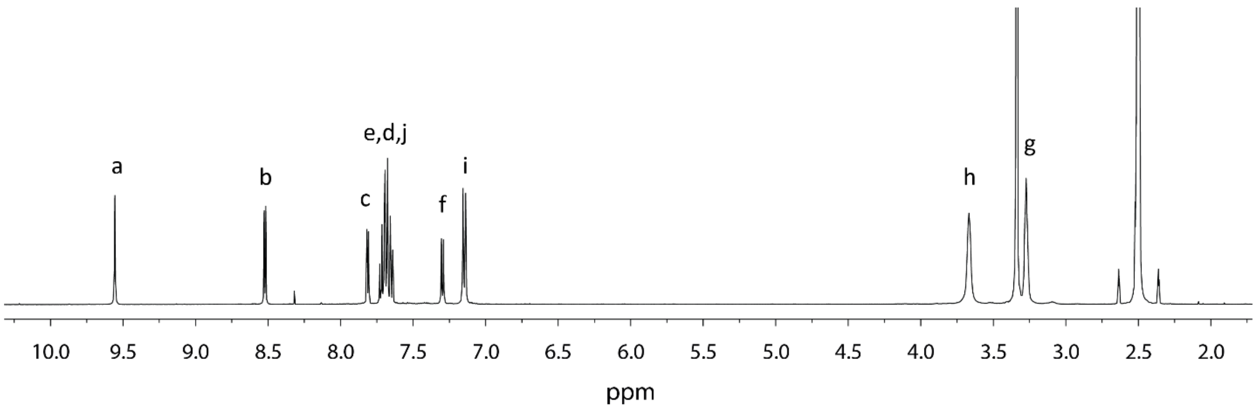


**Figure S25:** $^1$H NMR spectrum (600 MHz, 298K, DMSO-d6) of ligand **MK-Q**.

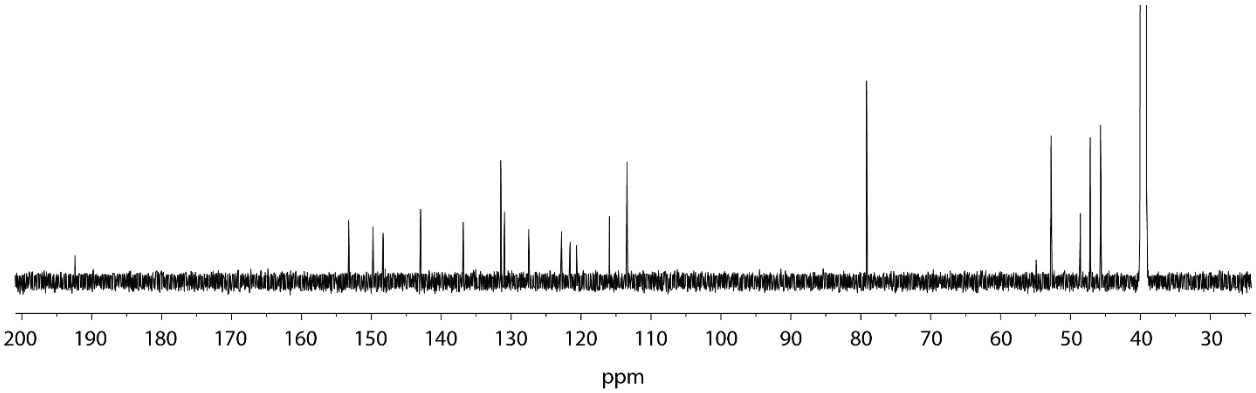


**Figure S26:** $^{13}C$ NMR spectrum (151 MHz, 298K, $CD_3CN$) of ligand **MK-Q**.

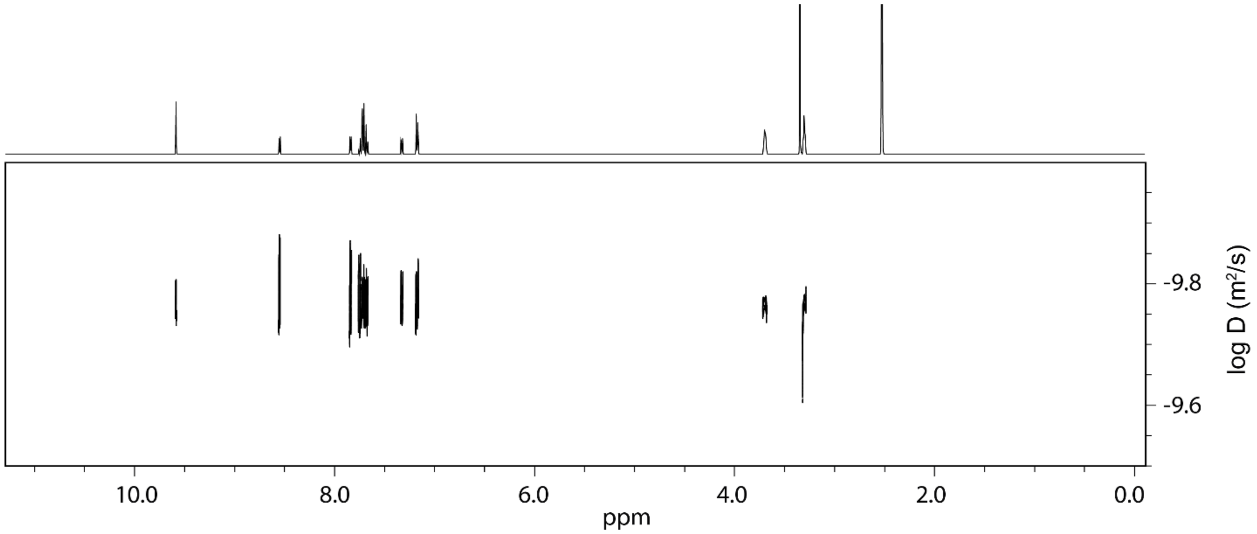


**Figure S27:** $^{1}H$ DOSY spectrum (500 MHz, 298K, DMSO-$d_6$) of **MK-Q** (2.8 mM). Diffusion coefficient: $1.705{\times}10^{-10}$ $m^2s^{-1}$, log D=-9. 768. Hydrodynamic radius= 6.44 Å.

### 3.1.8 Synthesis of RB-Q

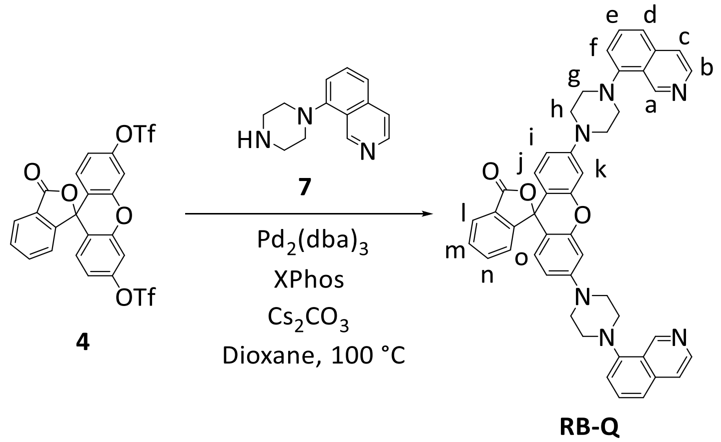


**Scheme S8:** Synthesis of ligand **RB-Q**.

Compound **4** (280 mg, 470 µmol, 1.0 equiv.) was added together with $Pd_2(dba)_3$ (86 mg, 94 µmol, 0.2 equiv.), XPhos (67.0 mg, 141 µmol, 0.3 equiv.), $Cs_2CO_3$ (430 mg, 1.3 mmol, 2.8 equiv.) and **7** (220 mg, 1.0 mmol, 2.2 equiv.) in dioxane into a Schlenk tube und the mixture was degassed via the pump- and freeze-method. The mixture was heated to 100 °C for 18 h. The solvent was evaporated and the reaction mixture was purified by column chromatography (0-10 % MeOH /$CH_2Cl_2$). The product was isolated as a pink solid (185 mg, 256 µmol, 54 %).

$^1$H NMR (500 MHz, 298 K, acetonitrile-$d_3$) δ 9.59 (s, 2H, Ha), 8.49 (d, *J* = 5.7 Hz, 2H, Hb), 7.99 (d, *J* = 7.6 Hz, 1H, Hl), 7.76 (t, *J* = 7.5 Hz, 1H, Ho), 7.73 – 7.62 (m, 3H, Hc, Hm), 7.58 (m, 4H, He, Hd), 7.25 (m, 3H, Hf, Hn), 6.86 (d, *J* = 2.4 Hz, 2H, Hk), 6.79 (dd, *J* = 9.1, 2.4 Hz, 2H, Hj), 6.70 (d, *J* = 8.8 Hz, 2H, Hi), 3.53-3.56 (m, 8H, Hh), 3.39 – 3.21 (m, 8H, Hg).

$^{13}$C NMR (126 MHz, 298 K, acetonitrile-$d_3$) δ 182.01 (from HMBC, C=O), 153.55, 151.27 (from HMBC), 149.65 (Ca), 144.10 (Cb), 131.81, 136.10 (from HSQC, Co), 131.40 (from HSQC, Cm), 129.70 (Ci), 125.37 (from HSQC, Cl), 124.43 (Cn), 122.69 (Ce and Cd, two signals overlaying), 121.56 (Cc), 116.79 (from HSQC, Cf), 112.67 (from HSQC, Cj), 110.31, 102.63 (Ck), 53.99 (Cg), 49.23 (Ch). 3 $C^q$ are not distinguishable from the noise.

HR ESI-MS: measured for ($C_{46}H_{38}N_6O_3H^+$): 723.3079

calculated: 723.3078

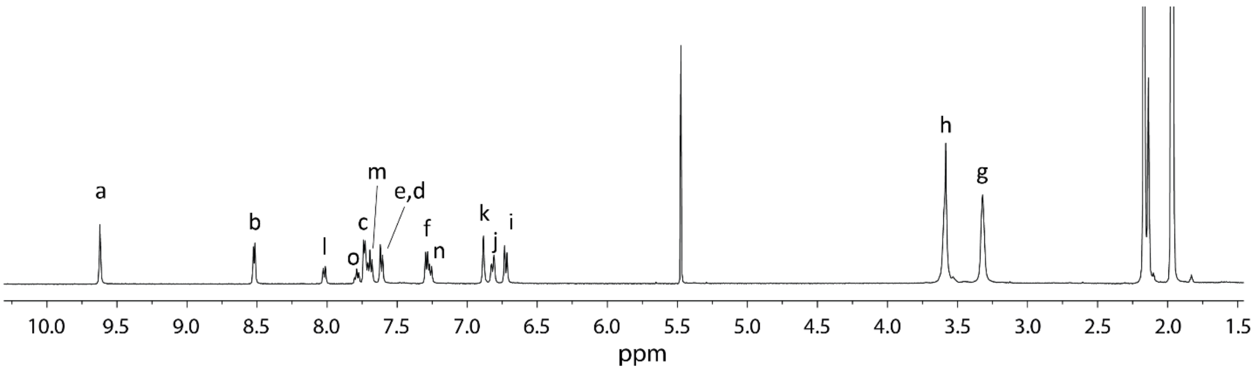


**Figure S28:** $^1$H NMR spectrum (500 MHz, 298K, $CD_3CN$) of ligand **RB-Q**.

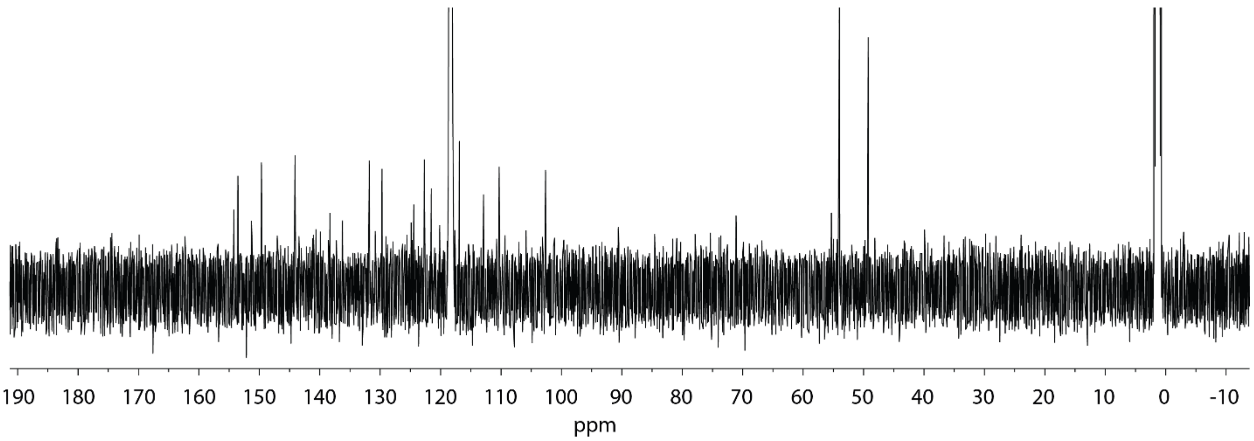


**Figure S29:** $^{13}$C NMR spectrum (126 MHz, 298K, $CD_3CN$) of ligand **RB-Q**.

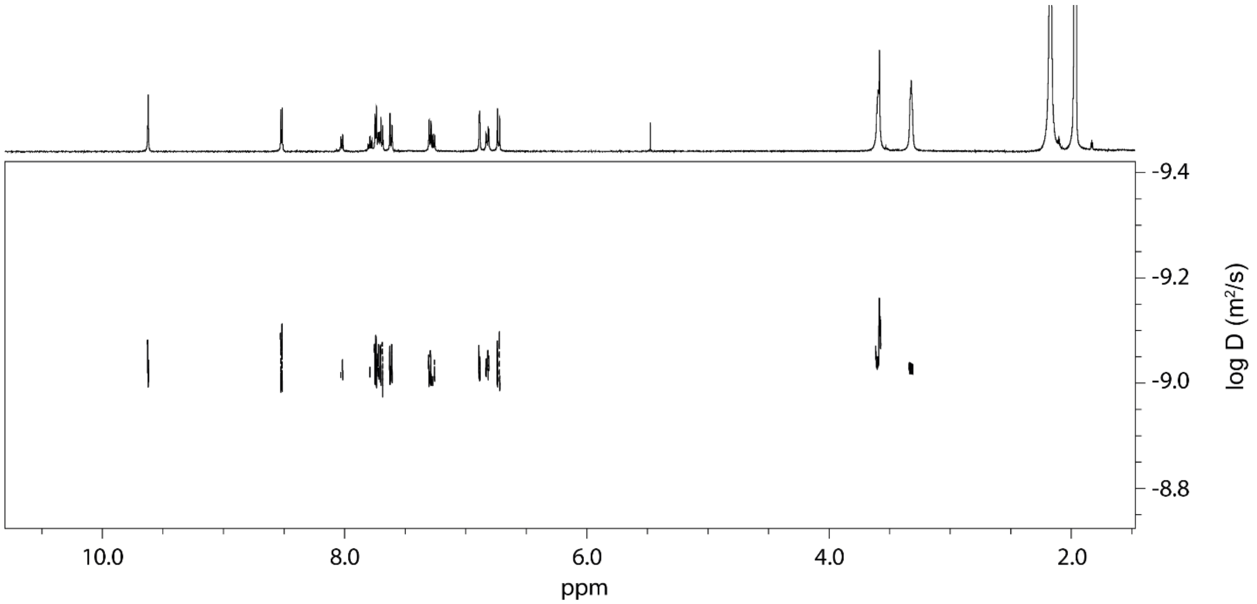


**Figure S30:** $^1$H DOSY spectrum (500 MHz, 298K, $CD_3CN$) of **RB-Q** (2.8 mM). Diffusion coefficient: $9.206\times10^{-10}$ $m^2s^{-1}$, log D=-9.036. Hydrodynamic radius= 7.11 Å.

### 3.1.9 Synthesis of MB-Q

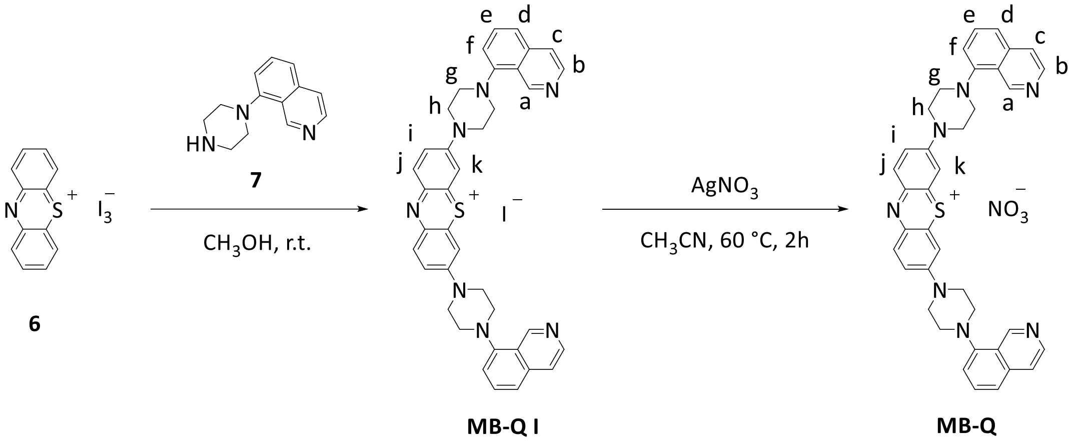


**Scheme S9:** Synthesis of ligand **MB-Q**.

#### 3.1.9.1 Synthesis of ligand **MB-Q**I

To a solution of the oxidized 10H-phenotiazine (0.300 g, 1.0 equiv.) in methanol (15 mL) was added dropwise the methanol (15 mL) solution of 8-(piperazin-1-yl)isoquinoline (0.885 g, 8.0 equiv.). The reaction was carried out under stirring at room temperature for 15 h. When the reaction was complete, the solution was evaporated to dryness under vacuum and purified by flash column chromatography ($R_f$=0.5, $CH_2Cl_2$/MeOH=95/5). After collection and evaporation, the compound was recrystallized by using $CH_3OH$/EtOAc as a blue powder.

$^1$H NMR (600 MHz, 298 K, acetonitrile-*d*$_3$) δ 9.69 (s, 1H, Ha), 8.56 (d, *J* = 5.6 Hz, 1H, Hb), 8.05 (d, *J* = 9.6 Hz, 1H, Hj), 7.77 (d, *J* = 5.7 Hz, 1H, Hc), 7.71 (t, *J* = 7.8 Hz, 1H, He), 7.69 – 7.64 (m, 2H, Hd, Hi), 7.55 (d, *J* = 2.8 Hz, 1H, Hk), 7.30 (dd, *J* = 7.4, 1.1 Hz, 1H, Hf), 4.16 (t, *J* = 4.9 Hz, 4H, Hh), 3.40 (t, *J* = 5.0 Hz, 4H, Hg).

$^{13}C$ NMR (151 MHz, 298 K, acetonitrile-$d_3$) δ 154.73 ($C^q$, **C**-Cj), 150.26 ($C^q$, **C**-Cf), 149.48 (Ca), 144.12 (Cb), 139.68 (Cj), 138.27 ($C^q$, Ck-**C**-Ci), 137.53 ($C^q$, Cc-**C**-Cd), 136.26 ($C^q$, **C**-Ck), 131.72 (Ce), 124.36 ($C^q$, **C**-Ca), 123.30 (Ci), 121.57 (Cc), 120.41 (Cd), 117.45 (Cf), 107.98 (Ck), 53.69 (Cg), 49.00 (Ch).

| | | |
|---|---|---|
| HR ESI-MS: | measured for ($C_{38}H_{34}N_7S^+$): | 620.2594 |
| | calculated: | 620.2634 |

### 3.1.9.2 Synthesis of ligand **MB-Q**

To an acetonitrile (15 mL) solution of **MB-Q**I (40 mg, 1 equiv.) was added $AgNO_3$ (12 mg, 1.3 equiv.). The reaction was carried out under stirring at 60 °C for 1 h. After cooling down to room temperature, the resulting solution was filtered and washed by using acetonitrile to get the filtrate. The solvent was then evaporated to obtain the product.

$^1H$ NMR (600 MHz, 298 K, acetonitrile-$d_3$) δ 9.69 (s, 1H, Ha), 8.56 (d, $J$ = 5.6 Hz, 1H, Hb), 8.05 (d, $J$ = 9.6 Hz, 1H, Hj), 7.77 (d, $J$ = 5.7 Hz, 1H, Hc), 7.71 (t, $J$ = 7.8 Hz, 1H, He), 7.69 – 7.64 (m, 2H, Hd, Hi), 7.55 (d, $J$ = 2.8 Hz, 1H, Hk), 7.30 (dd, $J$ = 7.4, 1.1 Hz, 1H, Hf), 4.16 (t, $J$ = 4.9 Hz, 4H, Hh), 3.40 (t, $J$ = 5.0 Hz, 4H, Hg).

$^{13}C$ NMR (151 MHz, 298 K, acetonitrile-$d_3$) δ 154.38 ($C^q$, **C**-Cj), 149.91 ($C^q$, **C**-Cf), 149.12 (Ca), 143.76 (Cb), 139.33 (Cj), 137.92 ($C^q$, Ck-**C**-Ci), 137.19 ($C^q$, Cc-**C**-Cd), 135.92 ($C^q$, **C**-Ck), 131.37 (Ce), 124.01 ($C^q$, **C**-Ca), 122.95 (Ci), 121.22 (Cc), 120.06 (Cd), 117.10 (Cf), 107.61 (Ck), 53.32 (Cg), 48.63 (Ch).

| | | |
|---|---|---|
| HR ESI-MS: | measured for ($C_{38}H_{34}N_7S^+$): | 620.2591 |
| | calculated: | 620.2634 |

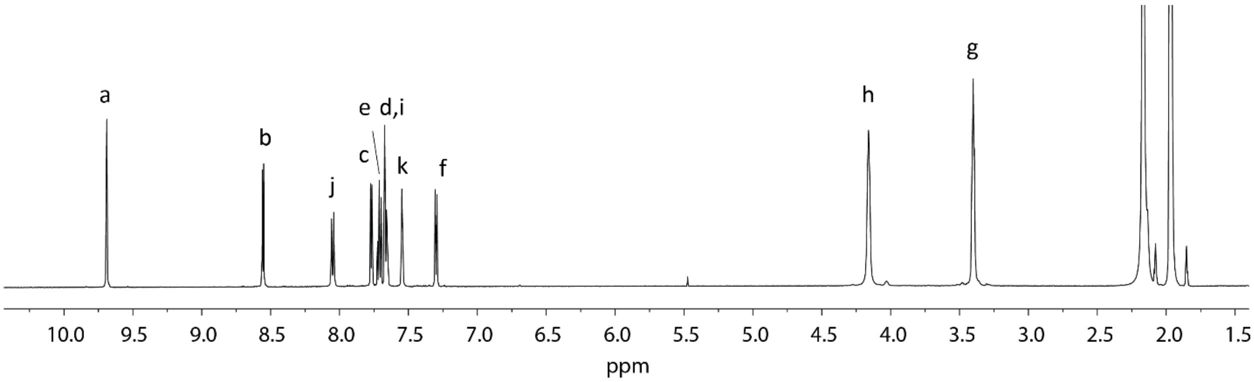


**Figure S31:** $^1H$ NMR spectrum (600 MHz, 298K, $CD_3CN$) of ligand **MB-Q**.

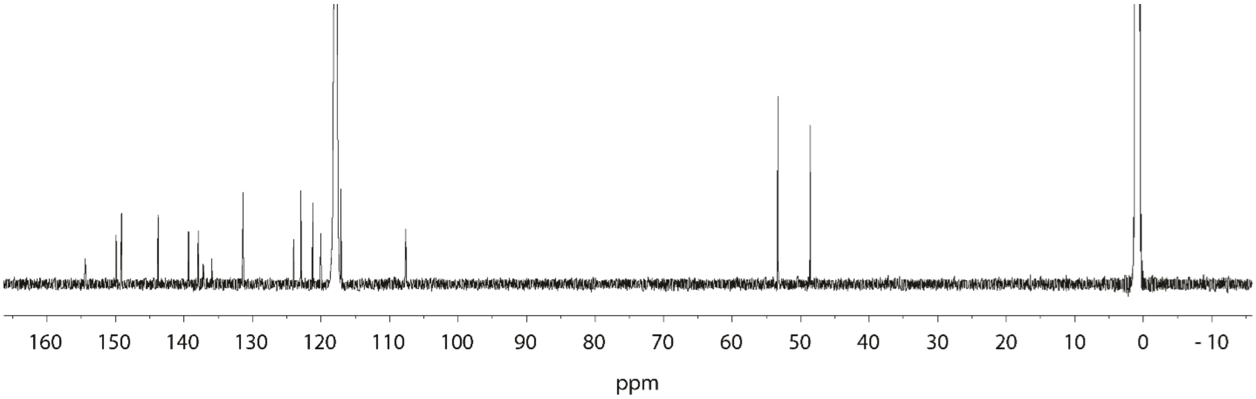


**Figure S32:** $^{13}C$ NMR spectrum (151 MHz, 298K, $CD_3CN$) of ligand **MB-Q**.

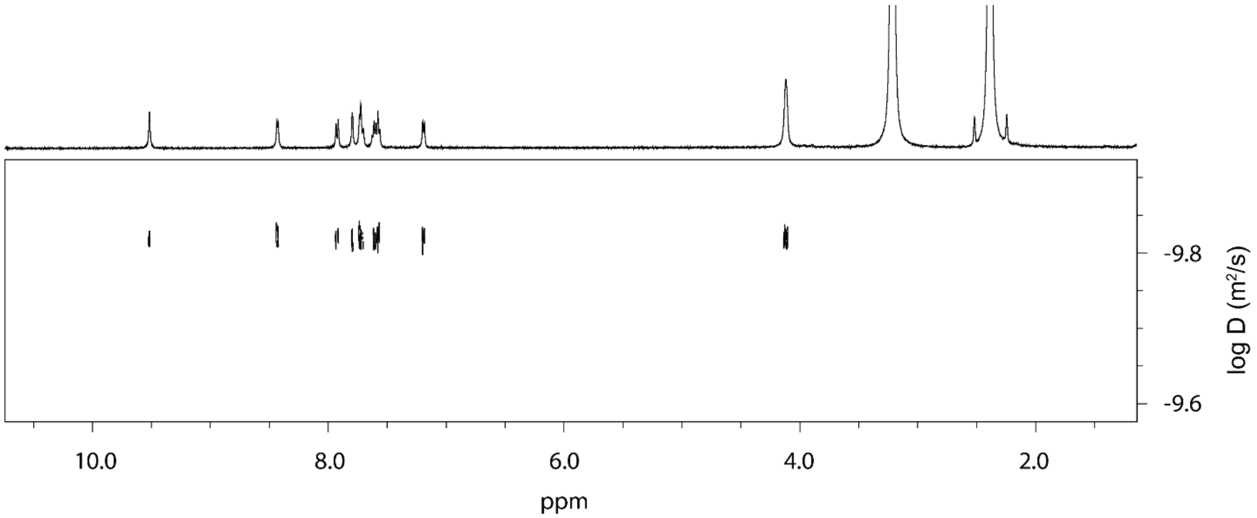


**Figure S33:** $^1$H DOSY spectrum (500 MHz, 298K, DMSO-$d_6$) of **MB-Q** (2.8 mM). Diffusion coefficient: $1.500{\times}10^{-10}$ $m^2s^{-1}$, log D=-9. 824. Hydrodynamic radius= 7.32 Å.

### 3.1.10 Synthesis of CV-Q

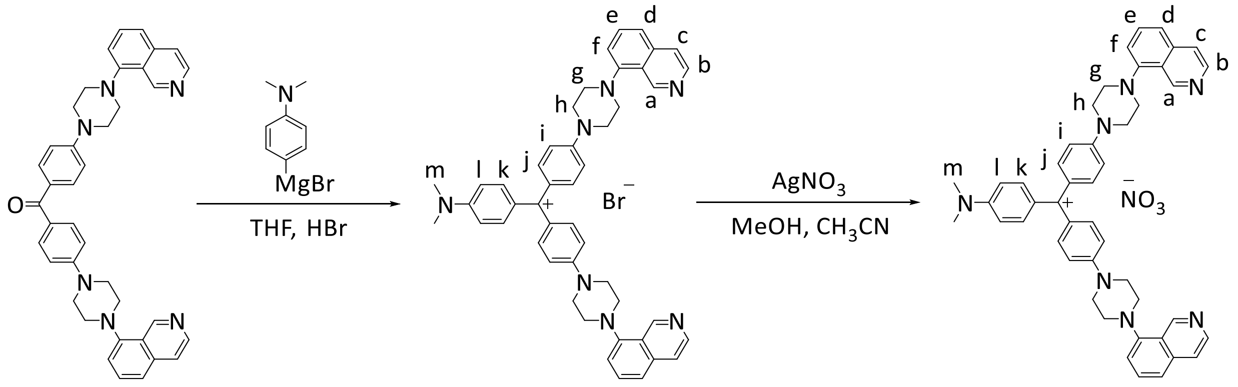


**BPQ** **CV-Q Br** **CV-Q**

**Scheme S10:** Synthesis of ligand **CV-Q**.

#### 3.1.10.1 Synthesis of **CV-Q**Br

To a round bottom flask equipped with stir bar, Mg (160 mg, 1.3 equiv., 6.60 mmol) was added and heated under vacuum to remove any trace of water. 4-Bromo-N,N-dimethylaniline (1 g, 5 mmol) was then added and the whole system was evacuated for some minutes. To the solids 10 mL of dry THF are added together with a small iodine crystal. The flask was gently warmed to reflux for 90 minutes. During this time the solution changes from dark to dingy gray. An ice bath was then used to cool down to room temperature the flask.

In a dry round bottom flask **MK-Q** (300 mg, 490 µmol) was added together with 8 mL of dry THF. To the yellow suspension, 3 mL of the grey suspension above described are added and the system was put under reflux for 5 minutes and then again cooled down to room temperature with an ice bath. Slowly 8 mL of HBr 10% are added to obtain the final product. The color of the solution changes from bright yellow to deep violet/blue. The solution was then neutralized with a saturated solution of $NaHCO_3$ and as soon as pH 7 was reached the desired product precipitates as blue flakes. All the volatiles are removed and the product purified

by column chromatography (0-10 % MeOH /$CH_2Cl_2$). The product was isolated as a dark violet solid (213 mg, 270 µmol, 55%).

$^1$H NMR (600 MHz, 298 K, acetonitrile-*d*$_3$) δ 9.65 (d, *J* = 0.9 Hz, 1H, Ha), 8.52 (d, *J* = 5.7 Hz, 1H, Hb), 7.75 (dd, *J* = 5.7, 1.0 Hz, 1H, Hc), 7.70 (dd, *J* = 8.2, 7.5 Hz, 1H, He), 7.63 (d, *J* = 8.2 Hz, 1H, Hd), 7.46 – 7.38 (m, 3H, Hk, Hj), 7.29 (dd, *J* = 7.5, 1.1 Hz, 1H, Hf), 7.23 – 7.16 (m, 2H, Hi), 6.98 (d, *J* = 9.3 Hz, 1H, Hl), 3.92 (t, *J* = 4.8 Hz, 4H, Hh), 3.34 (t, *J* = 5.1 Hz, 4H, Hg), 3.26 (s, 3H, Hm).

$^{13}$C NMR (151 MHz, 298 K, acetonitrile-*d*$_3$) δ 178.77 (C+), 157.22 ($C^q$, **C**-Ck), 156.33 ($C^q$, **C**-Cj), 150.53 ($C^q$, **C**-Cc), 148.96 (Ca), 143.35 (Cb), 141.04 (Ck), 140.14 (Cj), 138.02 ($C^q$, **C**-Ca), 131.58 (Ce), 128.59 ($C^q$, **C**-Ci), 127.33 ($C^q$, **C**-Cl), 124.00 ($C^q$, **C**-Cf), 122.63 (Cd), 121.35 (Cc), 117.27 (Cf), 114.09 (Ci), 113.49 (Cl), 53.38 (Cg), 47.58 (Ch), 40.64 (Cm).

HR ESI-MS: measured for ($C_{47}H_{46}N_7^+$): 708.3854

calculated: 708.3809

### 3.1.10.2 Synthesis of **CV-Q**

To a methanol (15 mL) solution of **CV-Q**Br (60 mg, 76 µmol, 1 equiv.) was added $AgNO_3$ (14 mg, 84 µmol, 1.1 equiv.). The reaction was carried out under stirring at 60 °C for 1 h. After cooling down to room temperature, the resulting solution was filtered and washed by using methanol to get the filtrate. The solvent was then evaporated to obtain the product.

$^1$H NMR (500 MHz, 298 K, acetonitrile-*d*$_3$) δ 9.66 (s, 1H, Ha), 8.51 (d, *J* = 5.8 Hz, 1H, Hb), 7.77 (d, *J* = 5.7 Hz, 1H, Hc), 7.71 (t, *J* = 7.9 Hz, 1H, He), 7.64 (d, *J* = 8.2 Hz, 1H, Hd), 7.41 (m, 3H, Hk, Hj), 7.30 (dd, *J* = 7.5, 1.0 Hz, 1H, Hf), 7.20 (d, *J* = 9.2 Hz, 2H, Hi), 6.98 (d, *J* = 9.4 Hz, 1H, Hl), 3.92 (t, *J* = 5.0 Hz, 4H, Hh), 3.34 (t, *J* = 5.0 Hz, 4H, Hg), 3.26 (s, 3H, Hm).

$^{13}$C NMR (151 MHz, 298 K, acetonitrile-*d*$_3$) δ 178.74 (C+), 157.23 ($C^q$, **C**-Ck), 156.31 ($C^q$, **C**-Cj), 150.66 ($C^q$, **C**-Cc), 149.08 (Ca), 143.04 (Cb), 141.04 (Ck), 140.14 (Cj), 138.16 ($C^q$, **C**-Ca), 131.98 (Ce), 128.59 ($C^q$, **C**-Ci), 127.33 ($C^q$, **C**-Cl), 124.02 ($C^q$, **C**-Cf), 122.63 (Cd), 121.66 (Cc), 117.93 (Cf), 114.10 (Ci), 113.51 (Cl), 53.40 (Cg), 47.57 (Ch), 40.65 (Cm).

HR ESI-MS: measured for ($C_{47}H_{46}N_7^+$): 708.3783

calculated: 708.3809

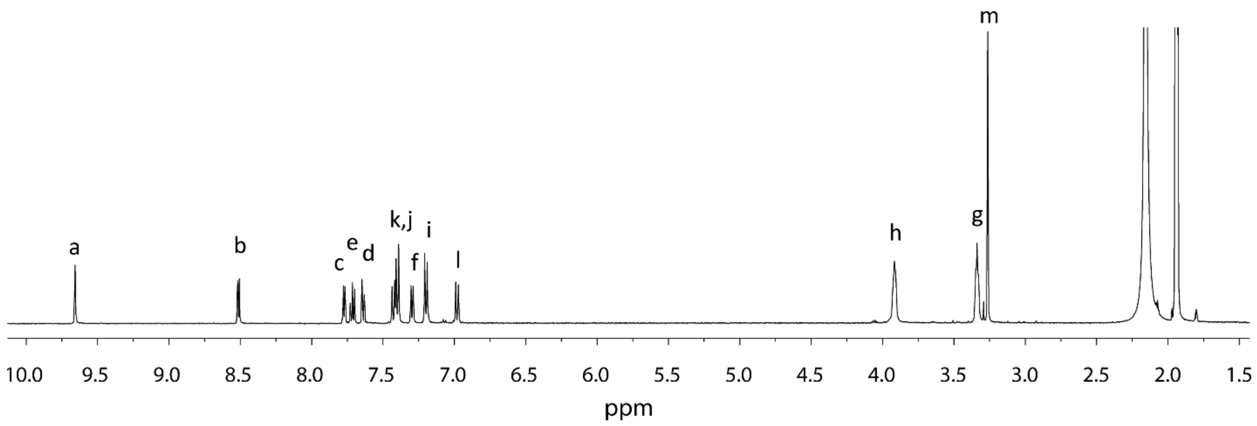


**Figure S34:** $^1$H NMR spectrum (600 MHz, 298K, $CD_3CN$) of ligand **CV-Q**.

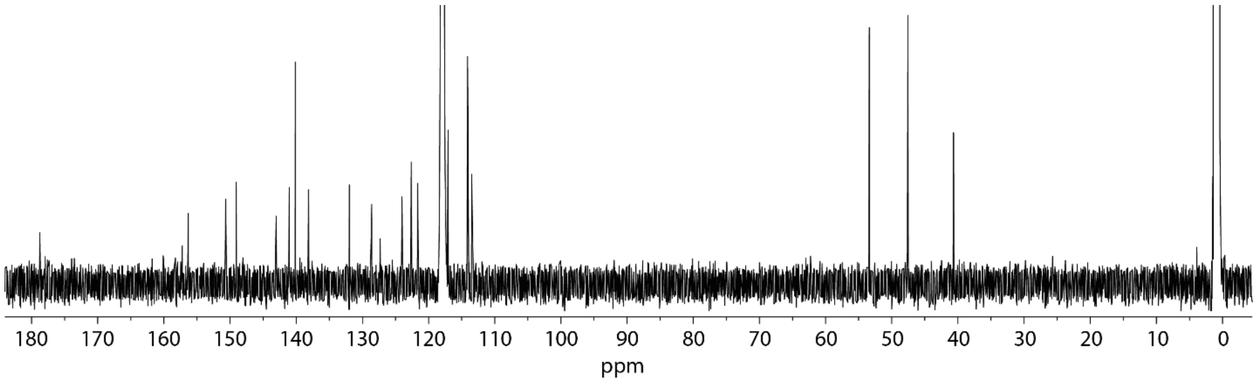


**Figure S35:** $^{13}C$ NMR spectrum (151 MHz, 298K, $CD_3CN$) of ligand **CV-Q**.

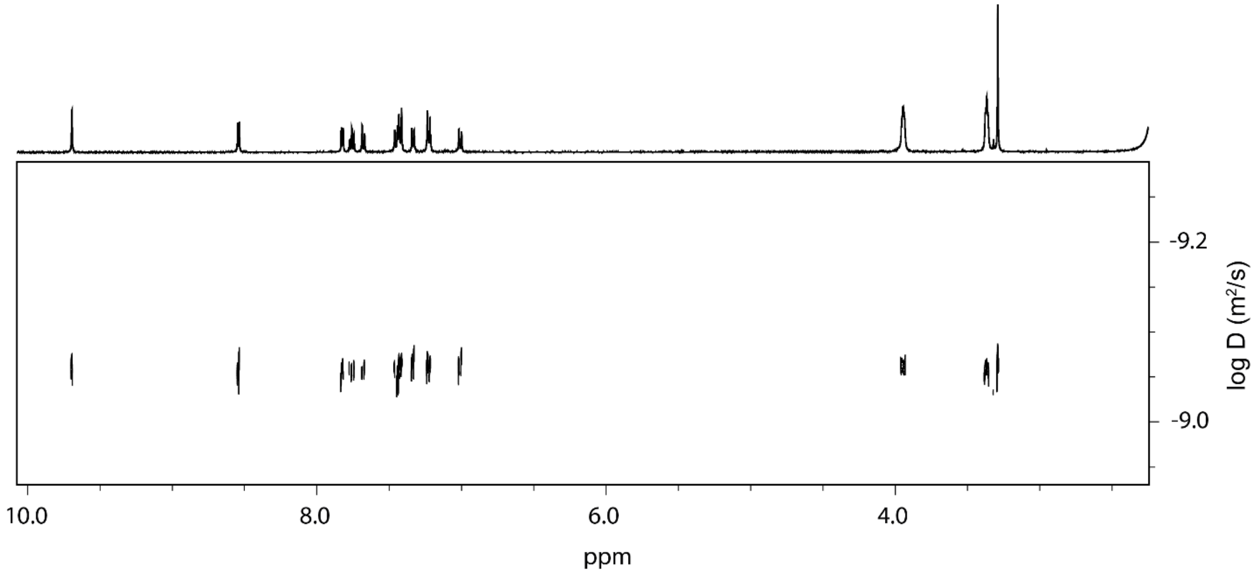


**Figure S36:** $^{1}H$ DOSY spectrum (500 MHz, 298K, $CD_3CN$) of **CV-Q** (2.8 mM). Diffusion coefficient: $8.771\times10^{-10}$ $m^2s^{-1}$, log D=-9.057. Hydrodynamic radius= 7.44 Å.

### 3.2 Assembly of the cages and helicates

All the $Pd_2L_4$ assemblies were synthetized accordingly to the same general procedure. To 450 µL of a 3.11 mM solution of the ligands in the specified solvent, 50 µL of a 15 mM solution of the Pd(II) salts in the same solvent (unless otherwise stated) are added. The mixture was then heated to 70 °C for 2 hours.

#### 3.2.1 $[Pd_2(\mathbf{MK\text{-}P})_4](BF_4)_4$

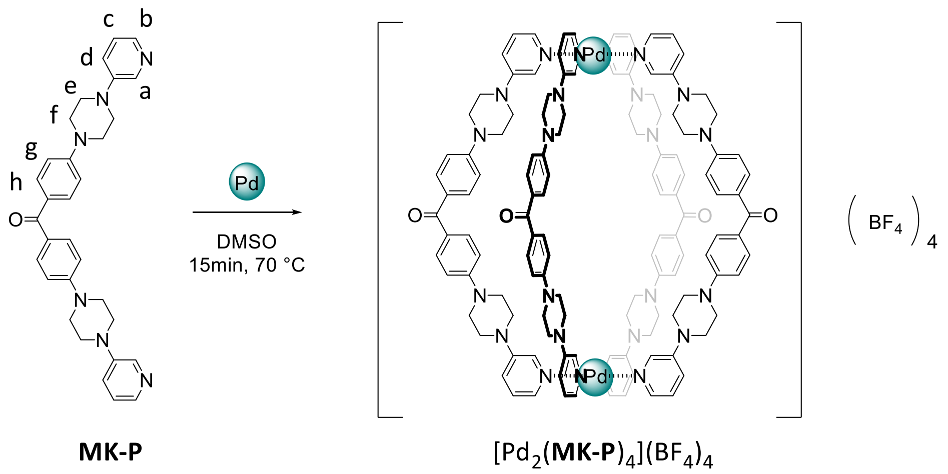


**Scheme S11:** Self-Assembly of cage $[Pd_2(\mathbf{MK\text{-}P})_4](BF_4)_4$.

A mixture of ligand **MK-P** (450 µL of a 3.11 mM solution in DMSO-$d_6$) and $[Pd(CH_3CN)_4](BF_4)_2$ (50 µL of a 15 mM solution in DMSO-$d_6$) was heated at 70 °C for 15 min to afford a 0.7 mM solution of $[Pd_2(\mathbf{MK\text{-}P})_4](BF_4)_4$.

$^1$H NMR (600 MHz, 298 K, dimethyl sulfoxide-$d_6$) δ 8.92 (s, 1H, Ha), 8.72 (d, $J$ = 5.3 Hz, 1H, Hb), 7.67-7.60 (m, 3H, Hd, Hh), 7.51 (dd, J=8.4, 5.3 Hz, 1H, Hc), 7.14 (d, $J$ = 8.4 Hz, 2H, Hg), 3.58 (s, 8H, Hf, He).

$^{13}$C NMR (151 MHz, 298 K, dimethyl sulfoxide-$d_6$) δ 192.40 (C=O), 153.47 ($C^q$, **C**-Cg), 148.07 ($C^q$, Cd-**C**-Ca), 140.00 (Cb), 136.27 (Ca), 132.03 (Ch), 127.86 ($C^q$, Ch-**C**), 126.99 (Cd), 124.70 (Cc), 113.87 (Cg), 46.48 (Ce), 46.19 (Cf).

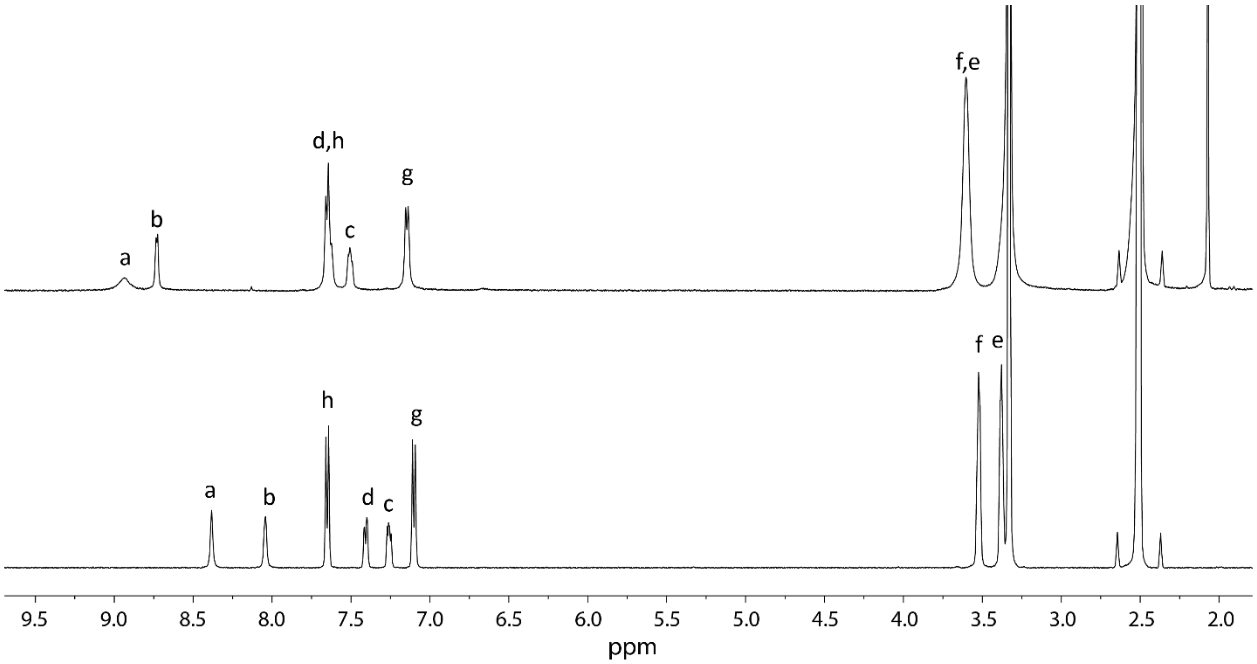


**Figure S37:** $^{1}H$ NMR stacked spectra (600 MHz, 298K, DMSO-$d_6$) of ligand **MK-P** (bottom) and the correspondent cage $[Pd_2($**MK-P**$)_4](BF_4)_4$ (top) upon addition of 0.5 equiv. of Pd(II) salt.

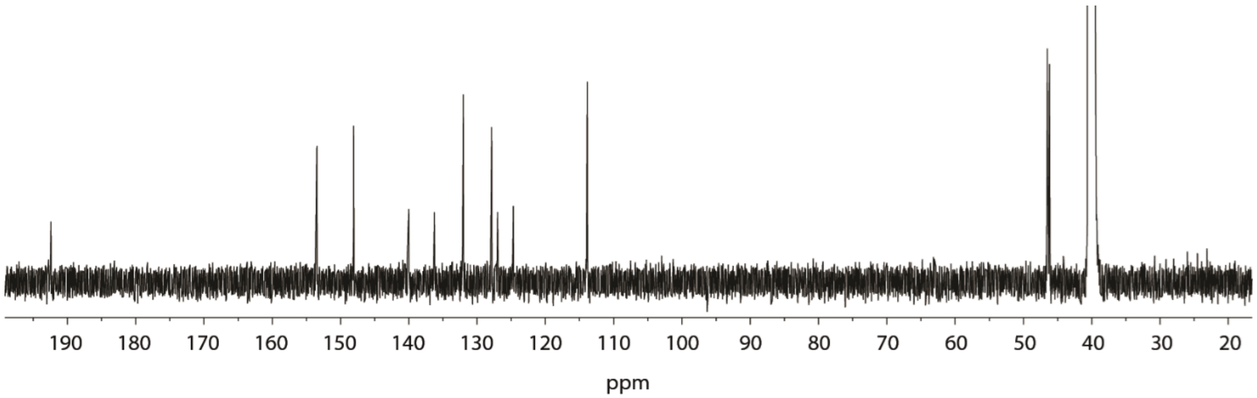


**Figure S38:** $^{13}C$ NMR spectrum (151 MHz, 298K, DMSO-$d_6$) of cage $[Pd_2($**MK-P**$)_4](BF_4)_4$.

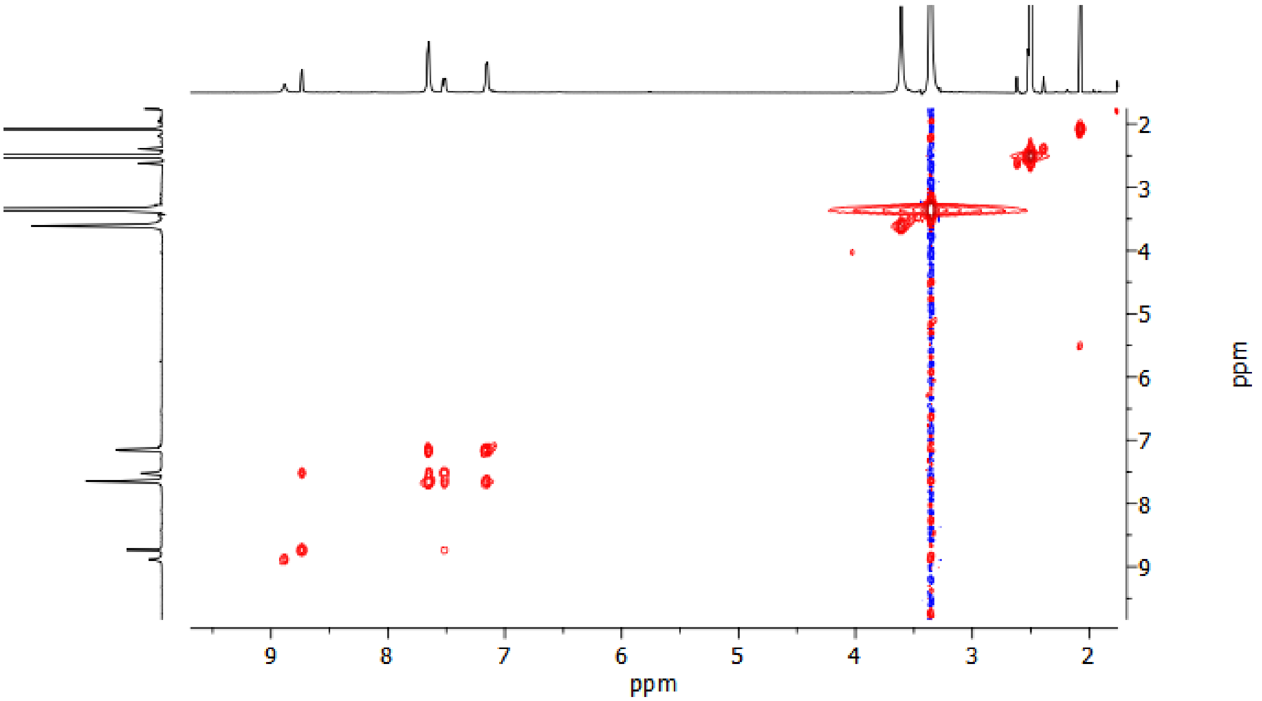


**Figure S39:** $^1H$ – $^1H$ COSY spectrum (600 MHz, 298K, DMSO-$d_6$) of cage $[Pd_2($**MK-P**$)_4](BF_4)_4$.

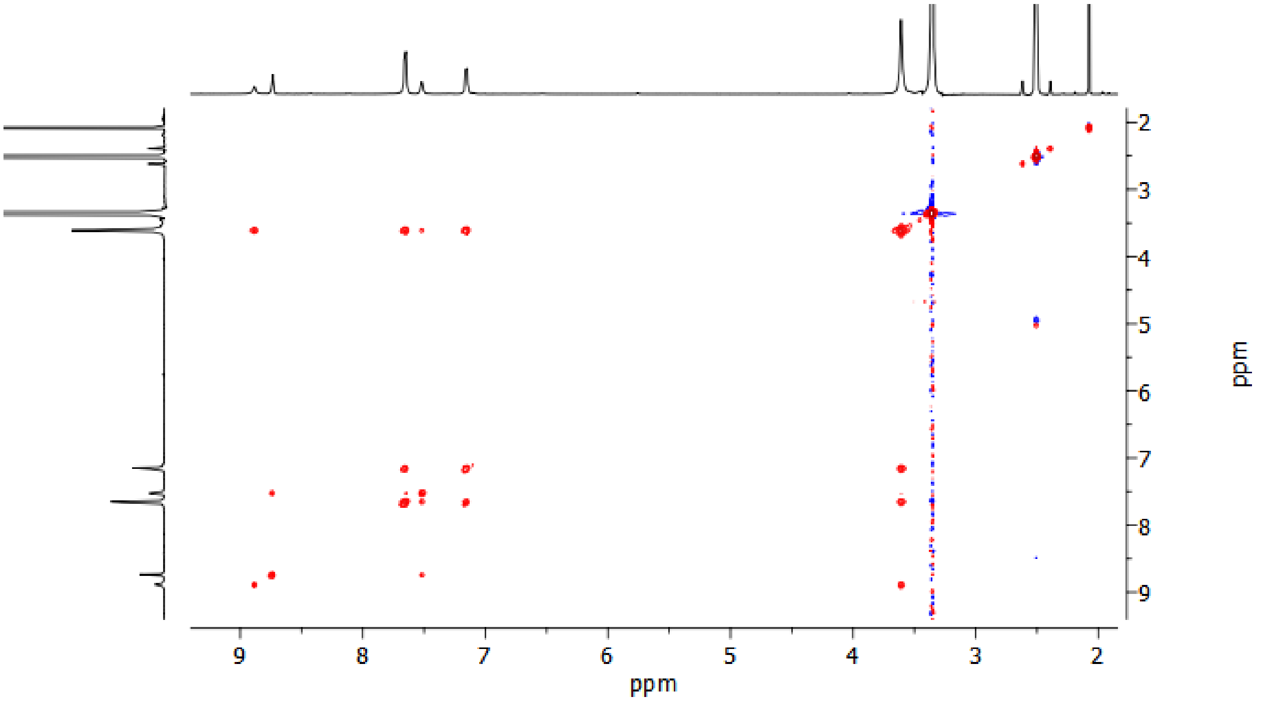


**Figure S40:** $^1H$ – $^1H$ NOESY spectrum (600 MHz, 298K, DMSO-$d_6$) of cage $[Pd_2($**MK-P**$)_4](BF_4)_4$.

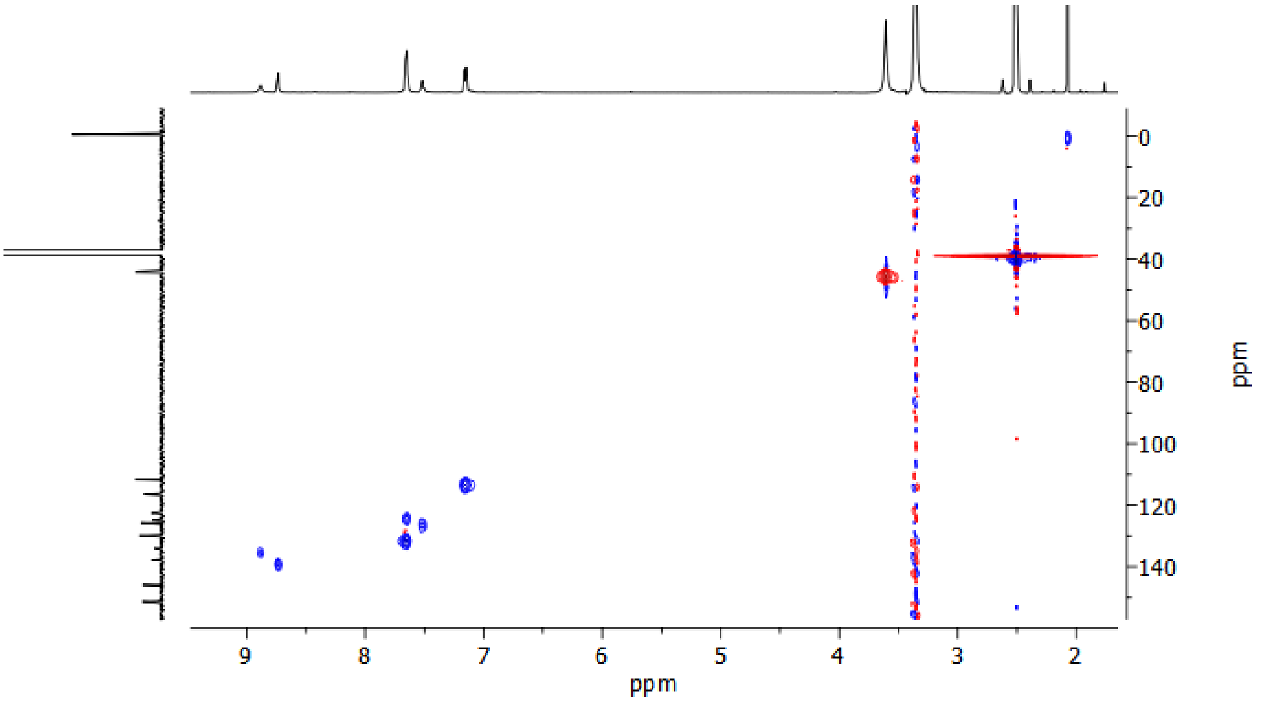


**Figure S41:** $^{1}H$ – $^{13}C$ HSQC spectrum (600 MHz, 298K, DMSO-$d_6$) of cage $[Pd_2($**MK-P**$)_4](BF_4)_4$.

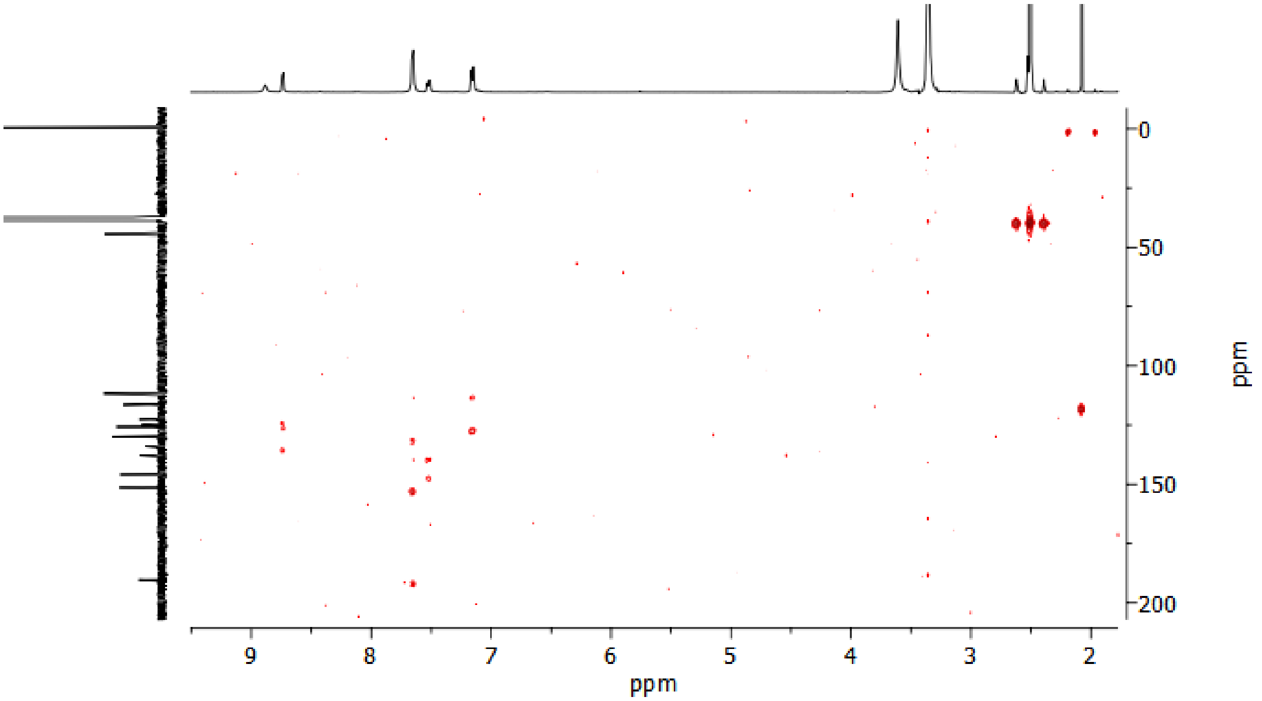


**Figure S42:** $^{1}H$ – $^{13}C$ HMBC spectrum (600 MHz, 298K, DMSO-$d_6$) of cage $[Pd_2($**MK-P**$)_4](BF_4)_4$.

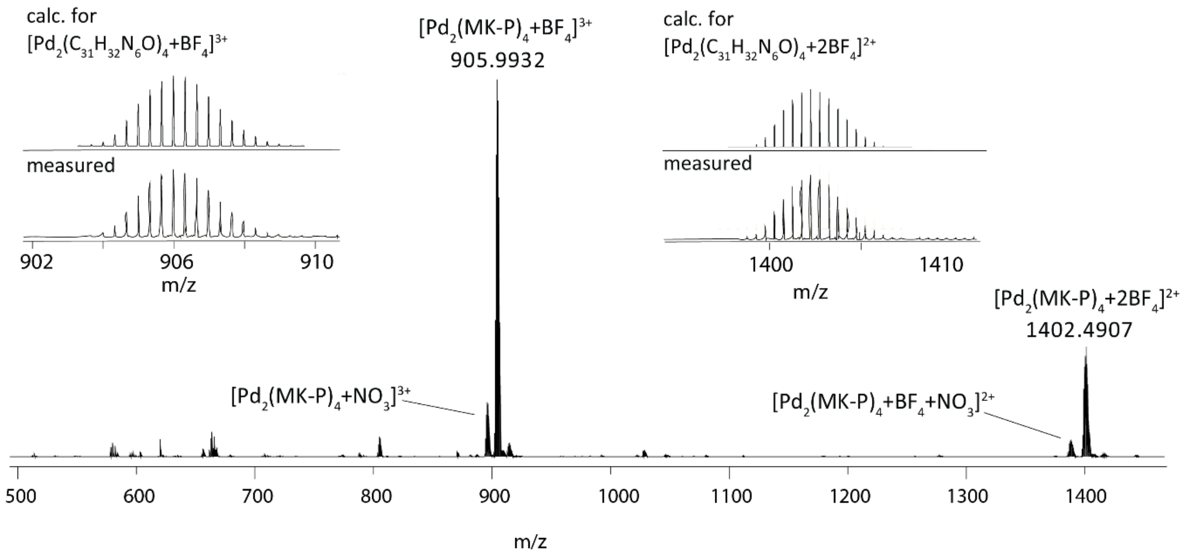


**Figure S43:** ESI-MS spectrum of $[Pd_2(\mathbf{MK\text{-}P})^4+nBF_4]^{(4-n)+}$ with n=0-2. The observed and calculated isotopic patterns of $[Pd_2(\mathbf{MK\text{-}P})_4+BF_4]^{3+}$ and $[Pd_2(\mathbf{MK\text{-}P})_4+2BF_4]^{2+}$ are shown in the inset.

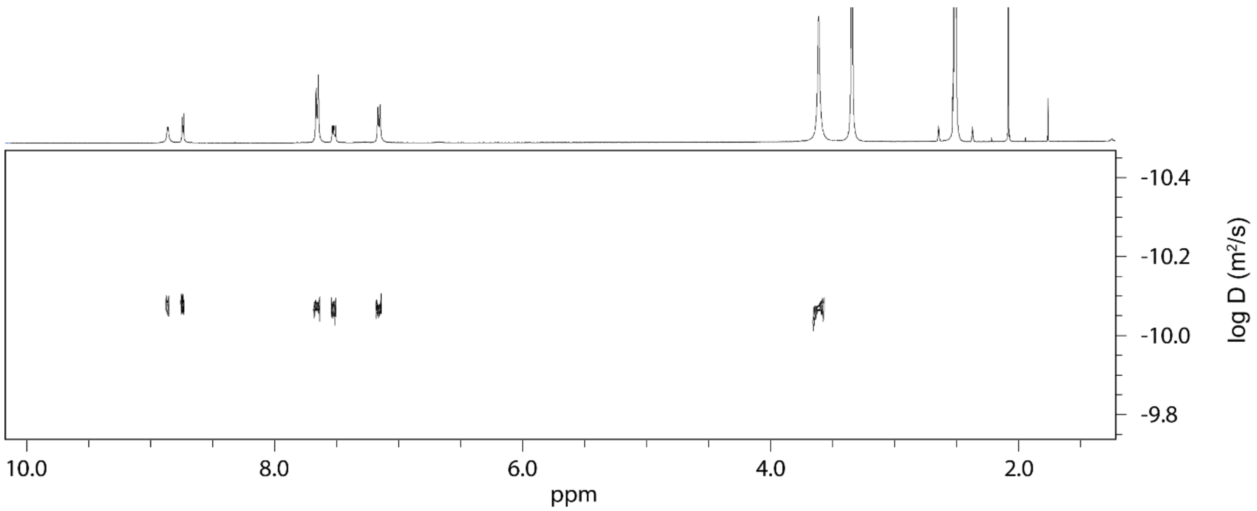


**Figure S44:** $^1$H DOSY spectrum (500 MHz, 298K, DMSO-$d_6$) of $[Pd_2(\mathbf{MK\text{-}P})_4](BF_4)$ (0.7 mM). Diffusion coefficient: $8.559\times10^{-11}$ $m^2s^{-1}$, log D=-10.068. Hydrodynamic radius= 12.83 Å.

### 3.2.2 [$Pd_2$(RB-P)$_4$]($BF_4$)$_4$

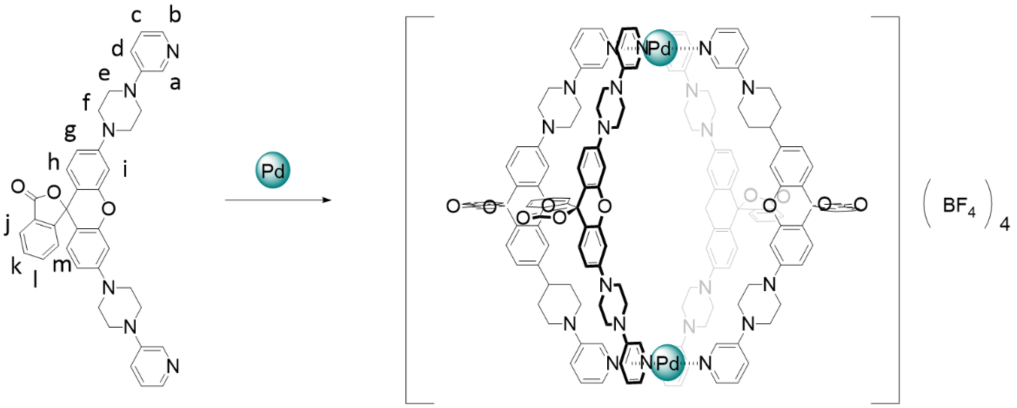


**RB-P** [$Pd_2$(**RB-P**)$_4$]($BF_4$)$_4$

**Scheme S12:** Self-Assembly of cage [$Pd_2$(**RB-P**)$_4$]($BF_4$)$_4$.

A mixture of ligand **RB-P** (450 µL of a 3.11 mM solution in DMSO-$d_6$) and [Pd($CH_3CN$)$_4$]($BF_4$)$_2$ (50 µL of a 15 mM solution in DMSO-$d_6$) was heated at 70 °C for 15 min to afford a 0.7 mM solution of [$Pd_2$(**RB-P**)$_4$]($BF_4$)$_4$.

$^1$H NMR (500 MHz, 298 K, dimethyl sulfoxide-$d_6$) δ 8.91 – 8.72 (m, 4H, Ha, Hb), 7.96 (s, 1H, Hj), 7.76 (t, *J* = 6.9 Hz, 1H, Hl), 7.64 (d, *J* = 9.2 Hz, 3H, Hd, Hk), 7.51 (dd, *J* = 8.7, 5.2 Hz, 2H, Hc), 7.24 – 7.13 (m, 1H, Hm), 6.93 (s, 2H, Hi), 6.83 (d, *J* = 9.2 Hz, 2H, Hg), 6.60 (d, *J* = 8.7 Hz, 2H, Hh), 3.79 – 3.43 (m, 16H, Hf, He).

$^{13}$C NMR (151 MHz, 298 K, dimethyl sulfoxide-$d_6$) δ 171.43 (C=O), 168.80 (C$^q$), 152.24 (C$^q$), 151.92 (C$^q$, C-Cm), 147.69 (C$^q$, Ca-**C**-Cd), 139.84 (Cb), 135.90 (Ca), 135.60 (Cl), 130.11 (Ck), 128.76 (Ch), 126.55 (Cc), 126.10 (C$^q$), 124.66 (Cd and Cj overlapping from HSQC), 123.82 (Cm), 118.10 (acetronitrile), 111.86 (Cg), 108.85 (C$^q$, Cg-C-Ci), 101.14 (Ci), 83.01 (C$^q$, spiroC), 46.63 (Ce), 45.84 (Cf).

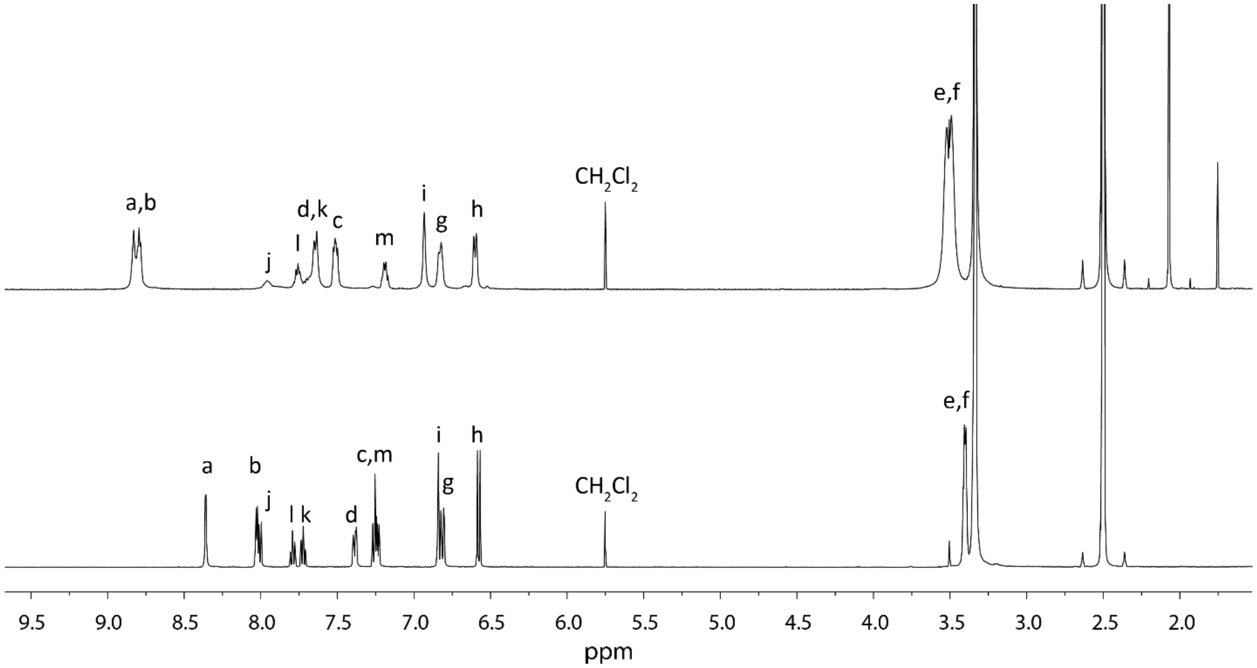


**Figure S45:** $^1$H NMR stacked spectra (600 MHz, 298K, DMSO-$d_6$) of ligand **RB-P** (bottom) and the correspondent cage [$Pd_2$(**RB-P**)$_4$]($BF_4$)$_4$ (top) upon addition of 0.5 equiv. of Pd(II) salt.

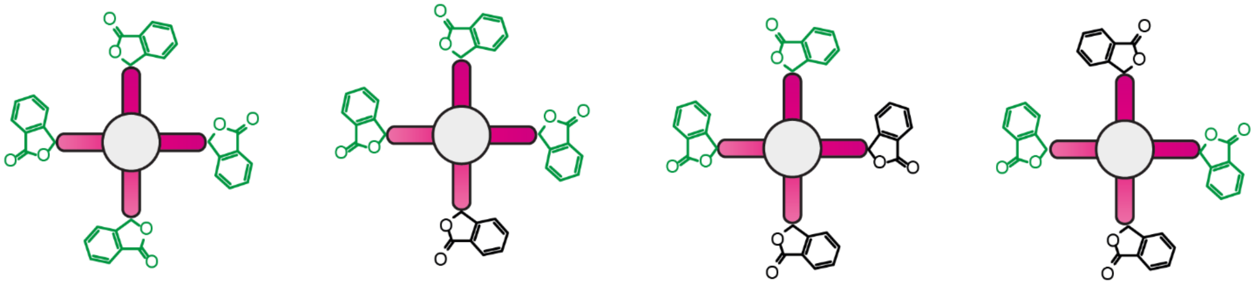

**Figure S46:** Schematic top view on the four different isomers of [$Pd_2$(**RB-P**)$_4$]($BF_4$)$_4$ that can originate from the flip of the spirolactone functional groups in ligand **RB-P**.

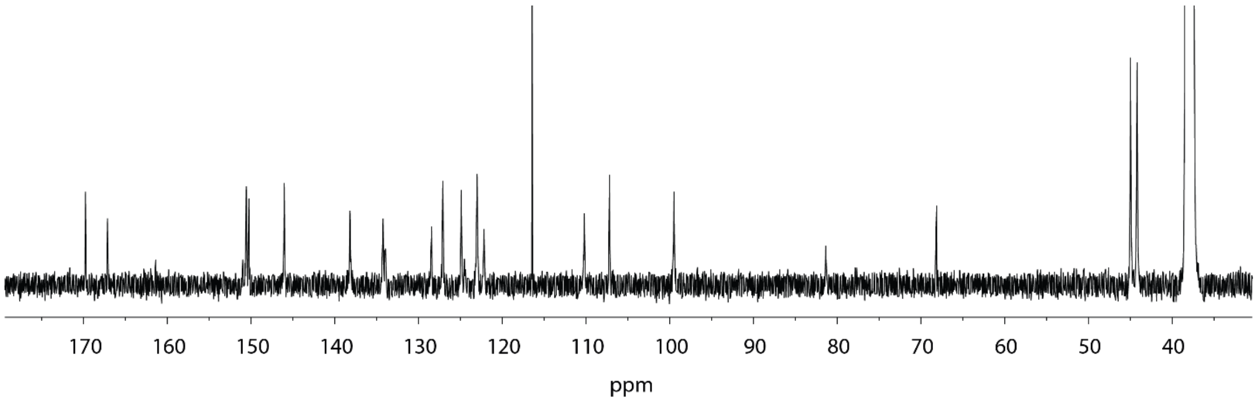


**Figure S47:** $^{13}C$ NMR spectrum (151 MHz, 298K, DMSO-$d_6$) of cage [$Pd_2$(**RB-P**)$_4$]($BF_4$)$_4$.

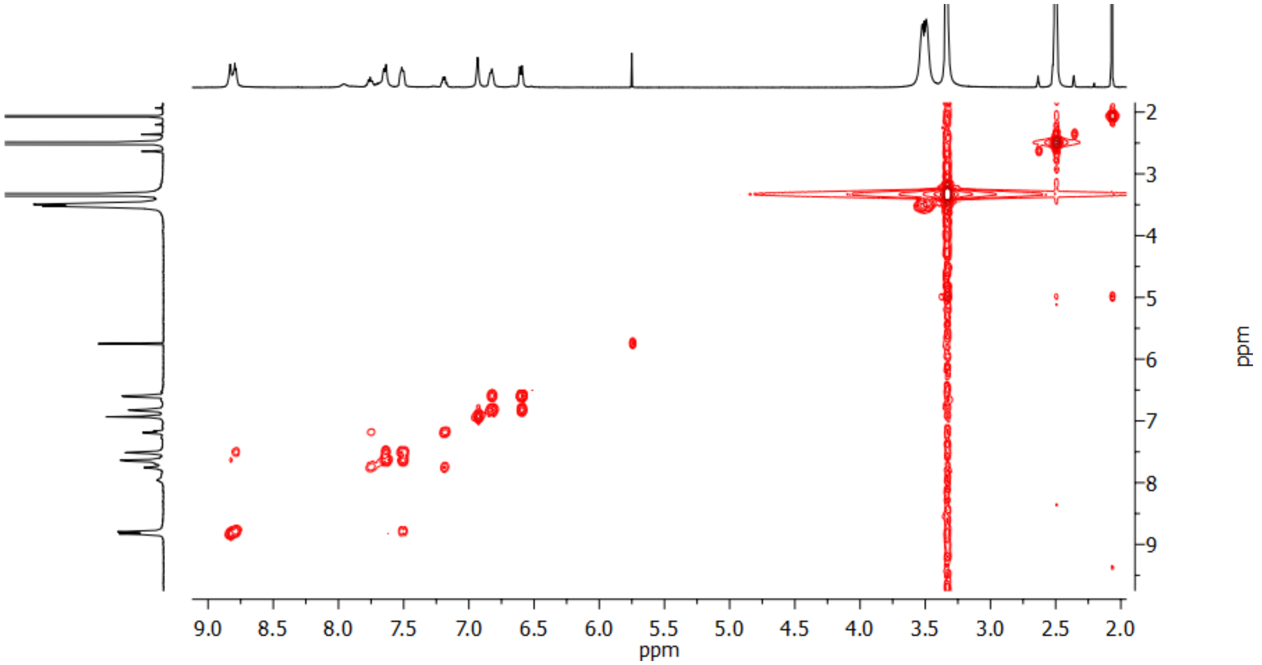


**Figure S48:** $^1H$ – $^1H$ COSY spectrum (600 MHz, 298K, DMSO-$d_6$) of cage [$Pd_2$(**RB-P**)$_4$]($BF_4$)$_4$.

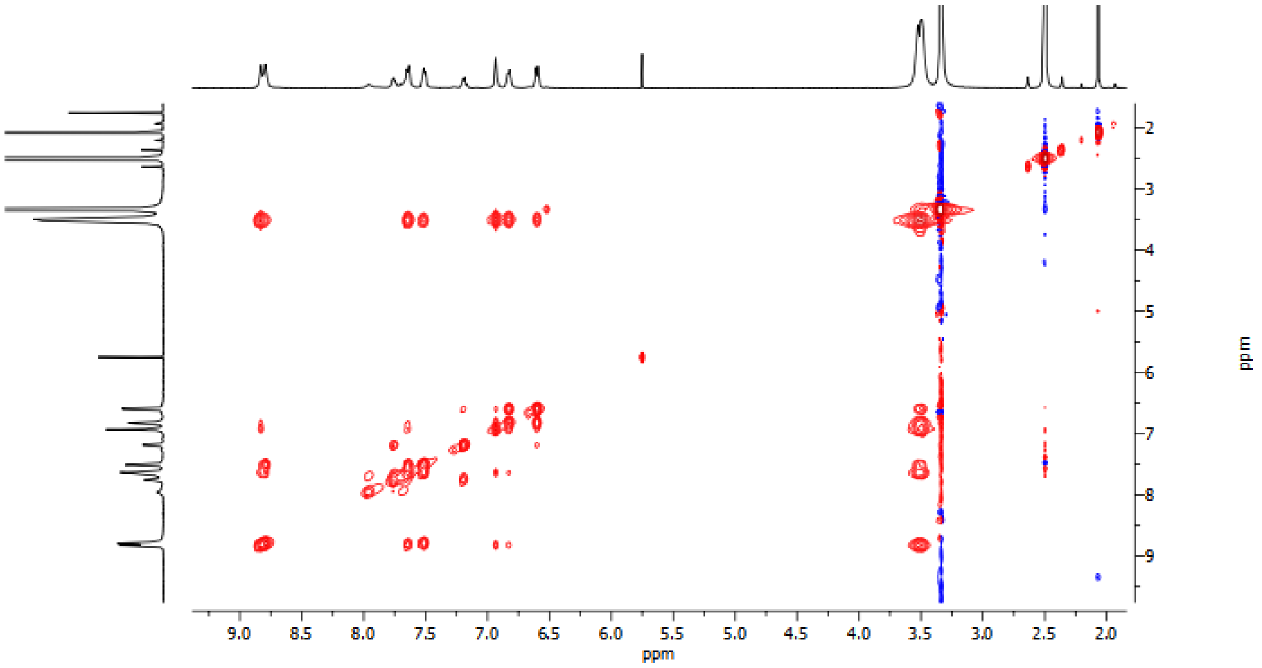

**Figure S49:** $^1H$ – $^1H$ NOESY spectrum (600 MHz, 298K, DMSO-$d_6$) of cage $[Pd_2(\mathbf{RB\text{-}P})_4](BF_4)_4$.

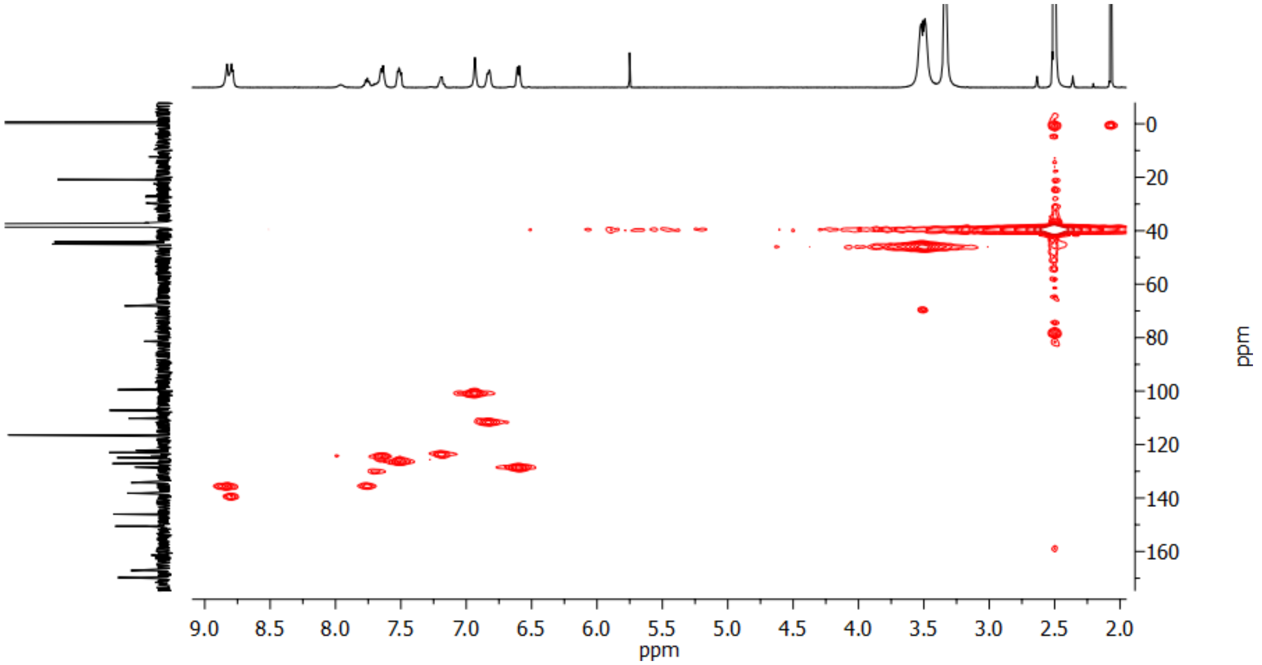

**Figure S50:** $^1H$ – $^{13}C$ HSQC spectrum (600 MHz, 298K, DMSO-$d_6$) of cage $[Pd_2(\mathbf{RB\text{-}P})_4](BF_4)_4$.

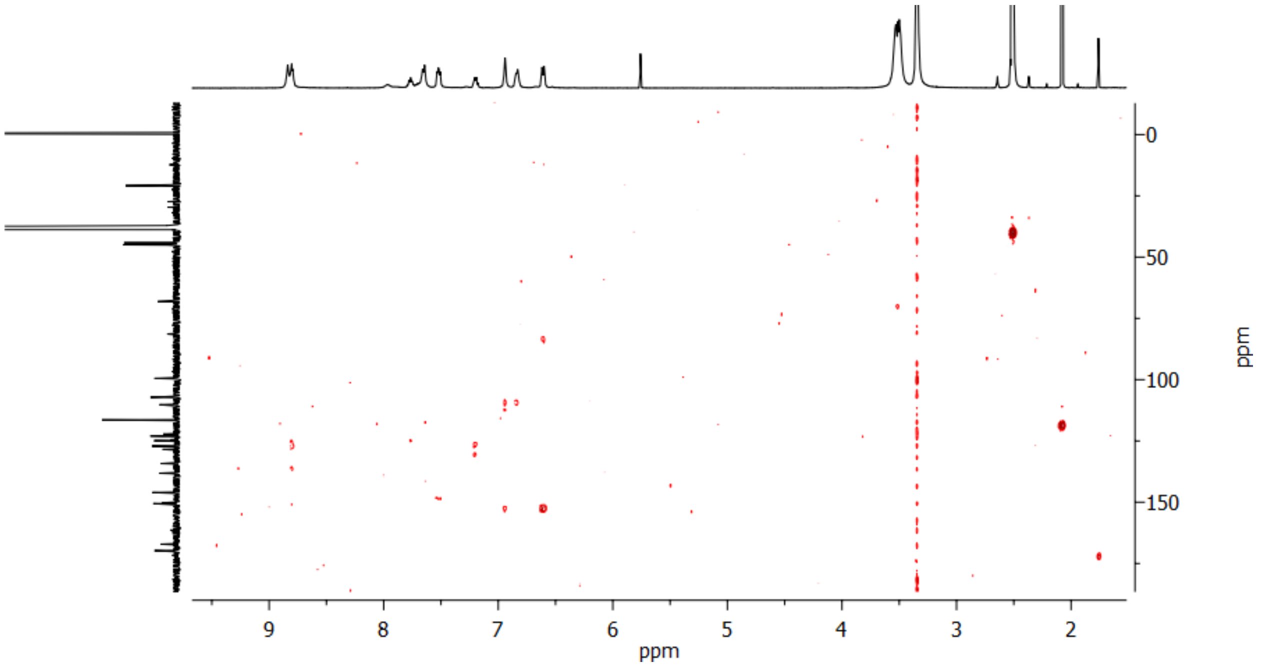


**Figure S51:** $^{1}H$ – $^{13}C$ HMBC spectrum (600 MHz, 298K, DMSO-$d_6$) of cage $[Pd_2($**RB-P**$)_4](BF_4)_4$.

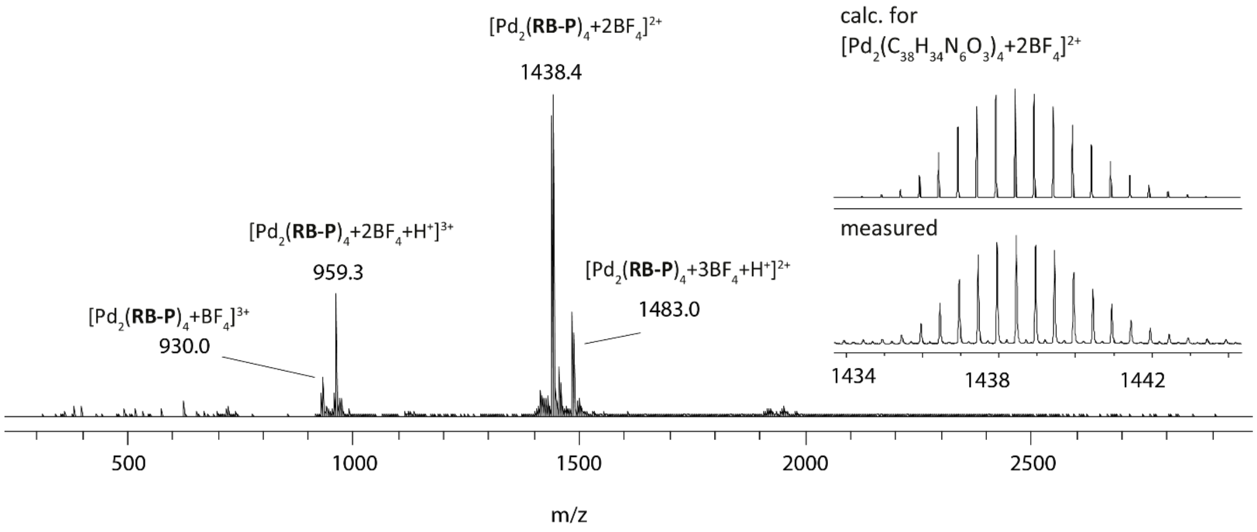


**Figure S52:** ESI-MS spectrum of $[Pd_2($**RB-P**$)^4+nBF_4]^{(4-n)+}$ with n=0-2. The observed and calculated isotopic pattern of $[Pd_2($**RB-P**$)_4+2BF_4]^{2+}$ is shown in the inset.

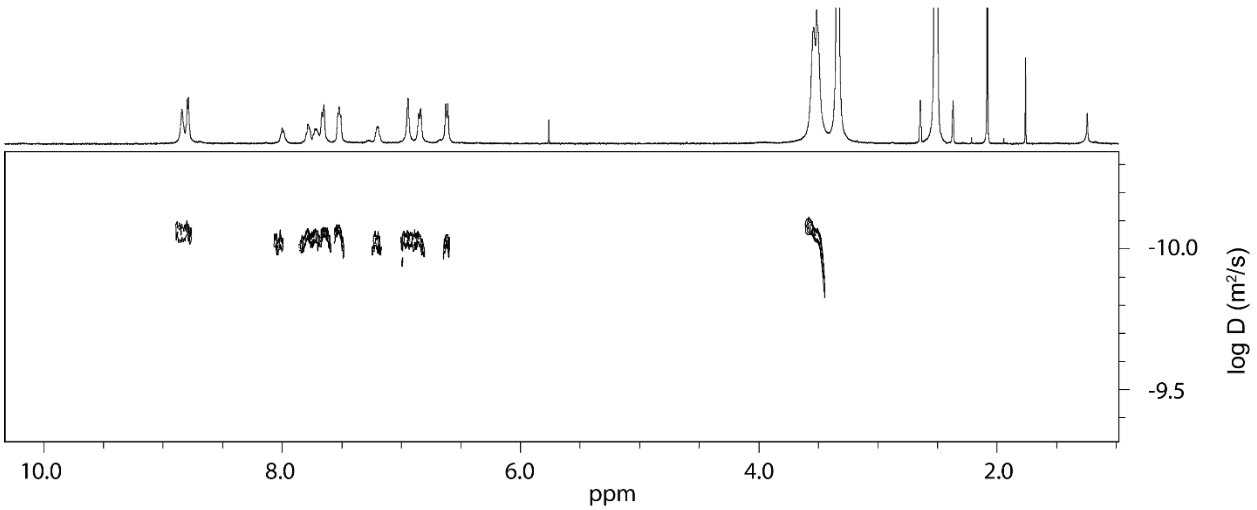


**Figure S53:** $^1$H DOSY spectrum (500 MHz, 298K, DMSO-$d_6$) of [$Pd_2$(**RB-P**)$_4$]($BF_4$) (0.7 mM). Diffusion coefficient: $8.642\times10^{-11}$ $m^2s^{-1}$, log D=-10.063. Hydrodynamic radius= 12.71 Å.

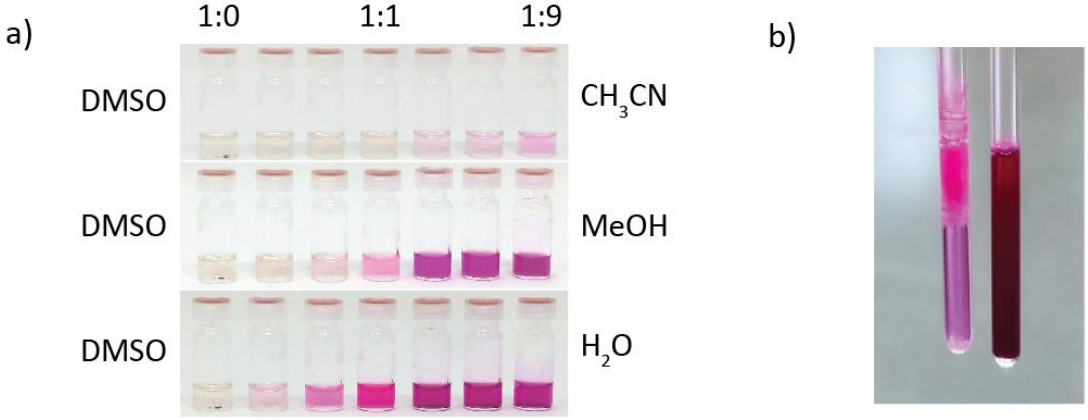


**Figure S54:** a) Pictures of vials containing [$Pd_2$(**RB-P**)$_4$] in DMSO and mixtures with increasing amounts of the polar solvents $CH_3CN$, MeOH and $H_2O$. The arising pink coloration corresponds to the open zwitterionic form of the dye. b) Picture of two NMR tubes containing ligand **RB-P** and cage [$Pd_2$(**RB-P**)$_4$] in DMSO:$D_2O$ = 1:9. The ligand is almost not soluble in this condition whereas the cage represents a homogeneous solution.

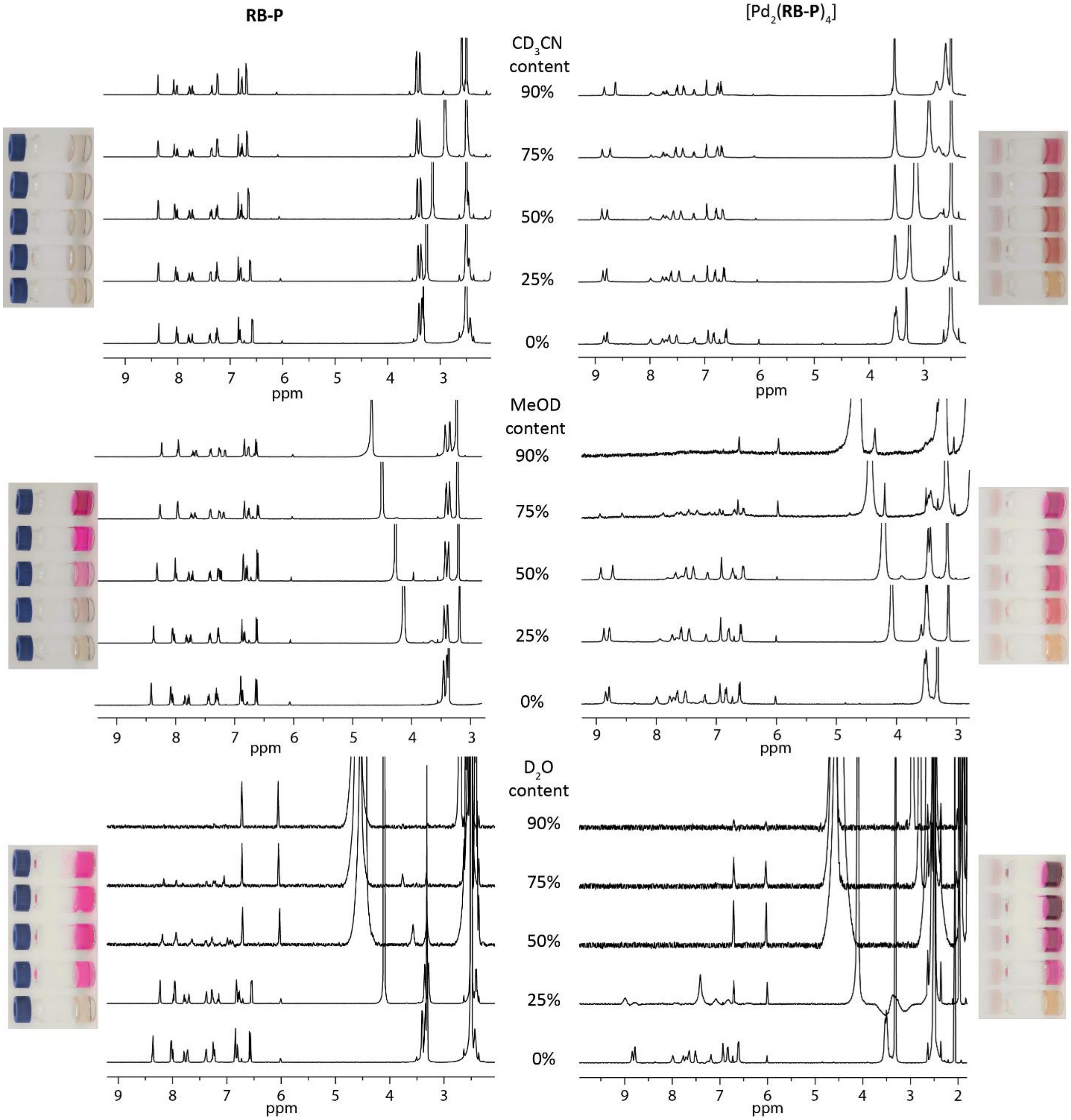


**Figure S55:** Stacked $^{1}$H NMR spectra and pictures of samples (**RB-P** on the left and $[Pd_2($**RB-P**$)_4](BF_4)_4$ on the right) in DMSO-$d_6$ with different amounts of $CD_3CN$, MeOD or $D_2O$ added. The specific contents are indicated in the Figure. Increasing contents of the more polar solvent have the general effect of broadening the spectra and causing an overall shift of the signals assigned to the cage protons (smaller shifts are observed for the signals assigned to the ligands). However, in none of the cases, increasing amounts of the polar solvent lead to appearance of ligand signals confirming the cages remain fully assembled under these conditions. In view of the low solubilty of **RB-P** in high water contents, free ligand signals would not be observed, but we refer to Figure S54 where the high coloration of the solution is attributed to the intact cage assembly.

### 3.2.3 $[Pd_2$(MB-P)$_4](BF_4)_8$ and $[Pd_2$(MB-P)$_4](NO_3)_8$

#### 3.2.3.1 $[Pd_2$(**MB-P**)$_4](BF_4)_8$

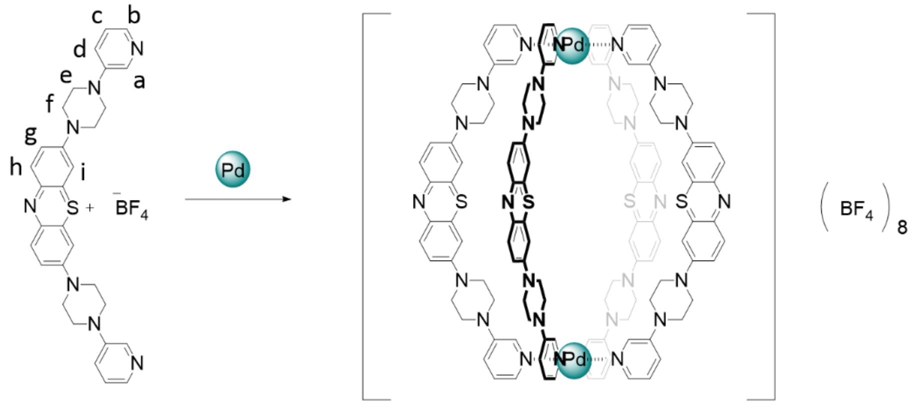


**MB-P**$BF_4$ $[Pd_2$(**MB-P**)$_4](BF_4)_8$

**Scheme S13:** Self-Assembly of cage $[Pd_2$(**MB-P**)$_4](BF_4)_8$

A mixture of ligand **MB-P**$BF_4$ (450 µL of a 3.11 mM solution in DMSO-*d*$_6$) and $[Pd(CH_3CN)_4](BF_4)_2$ (50 µL of a 15 mM solution in DMSO-*d*$_6$) was heated at 70 °C for 15 min to afford a 0.7 mM solution of $[Pd_2$(**MB-P**)$_4](BF_4)_8$.

$^1$H NMR (600 MHz, 298 K, dimethyl sulfoxide-*d*$_6$) δ 8.76 (m, 2H, Ha, Hb), 8.03 (d, *J* = 9.5 Hz, 1H, Hh), 7.82 (d, *J* = 2.5 Hz, 1H, Hi), 7.72 (dd, *J* = 9.5, 2.5 Hz, 1H, Hg), 7.69 – 7.59 (m, 1H, Hd), 7.53 (dd, *J* = 8.6, 5.4 Hz, 1H, Hc), 4.11 (m, 4H, Hf), 3.73 (m, 4H, He).

$^{13}$C NMR (151 MHz, 298 K, dimethyl sulfoxide-*d*$_6$) δ 153.13 (C$^q$, **C**-Ch), 146.73(C$^q$, **C**-Ci), 139.27 (Cb), 138.25 (Ch), 135.83 (C$^q$, Cg-**C**-Ci), 134.75 (Ca-**C**-Cd), 134.31 (Ca), 126.40 (Cc), 123.75 (Cd), 119.38 (Cg), 106.95 (Ci), 46.00 (Ce), 44.60 (Cf).

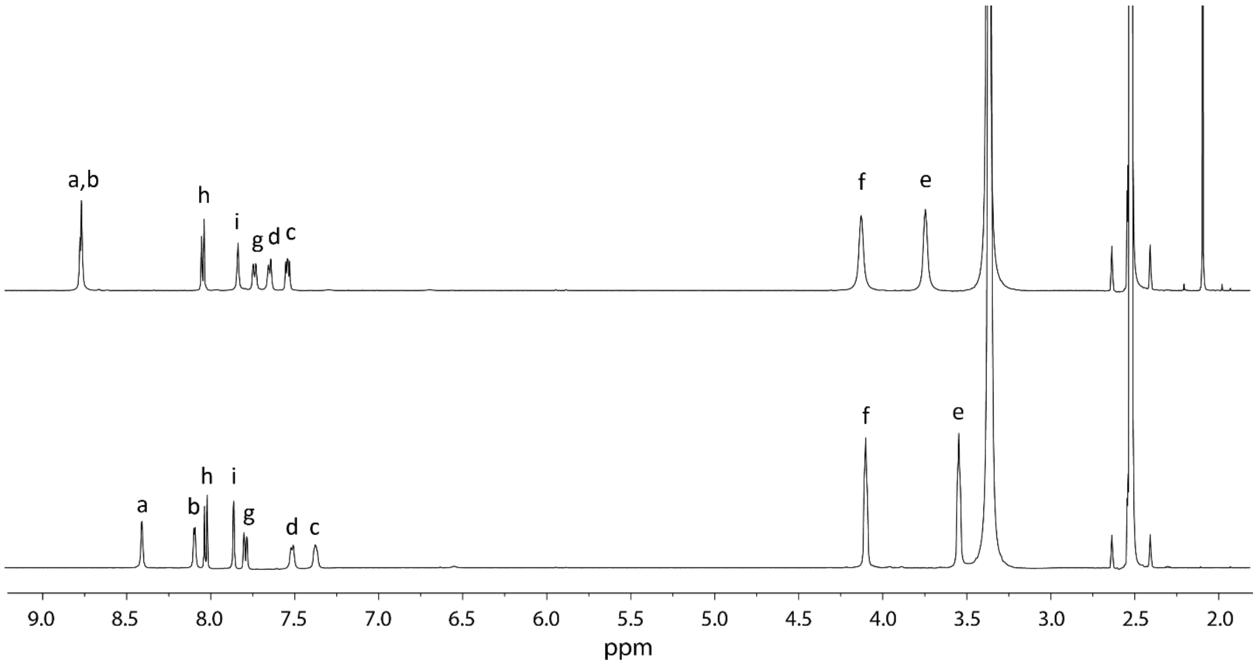


**Figure S56:** $^1$H NMR stacked spectra (600 MHz, 298K, DMSO-*d*$_6$) of ligand **MB-P**$BF_4$ (bottom) and the correspondent cage $[Pd_2$(**MB-P**)$_4](BF_4)_8$ (top) upon addition of 0.5 equiv. of Pd(II) salt.

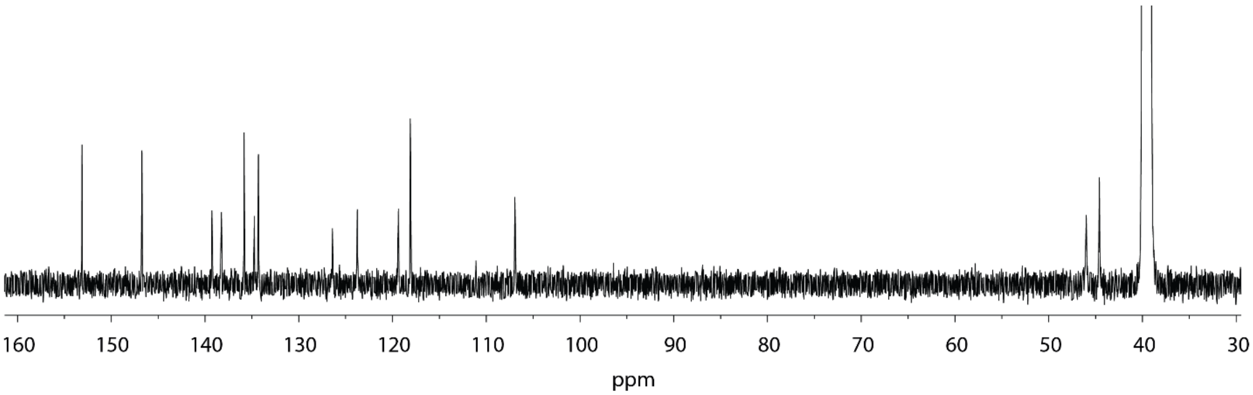


**Figure S57:** $^{13}$C NMR spectrum (151 MHz, 298K, DMSO-$d_6$) of cage $[Pd_2($**MB-P**$)_4](BF_4)_8$.

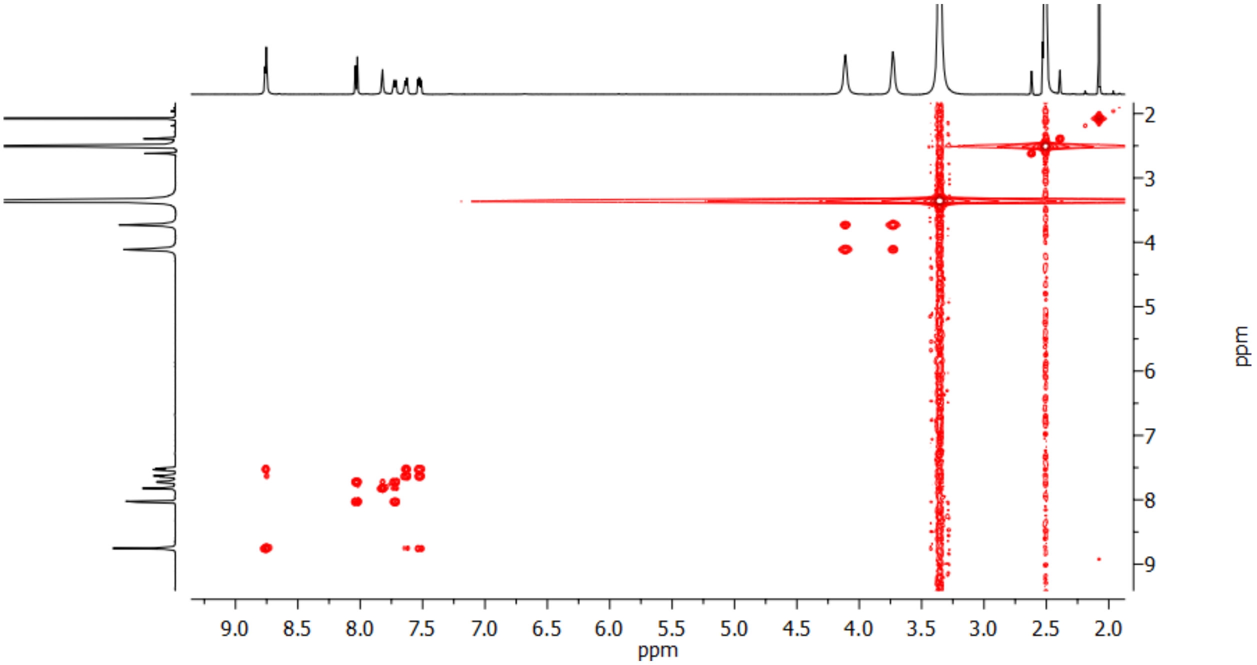


**Figure S58:** $^{1}$H – $^{1}$H COSY spectrum (600 MHz, 298K, DMSO-$d_6$) of cage $[Pd_2($**MB-P**$)_4](BF_4)_8$.

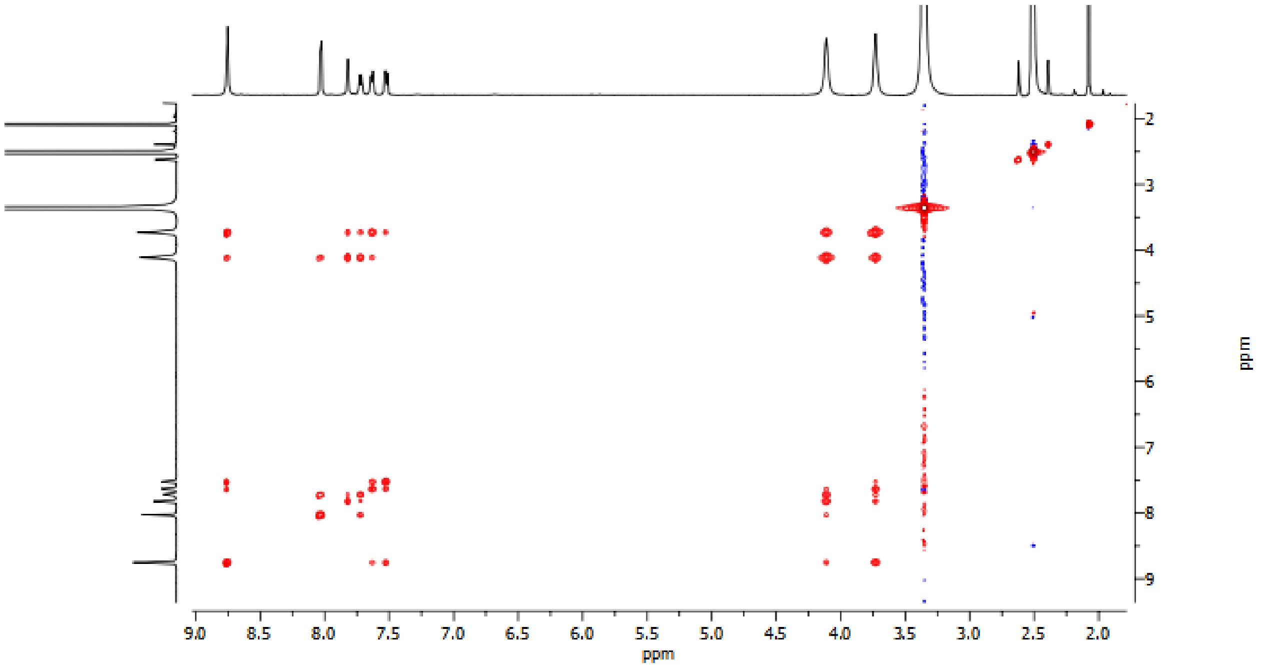


**Figure S59:** $^{1}H$ – $^{1}H$ NOESY spectrum (600 MHz, 298K, DMSO-$d_6$) of cage $[Pd_2(\mathbf{MB\text{-}P})_4](BF_4)_8$.

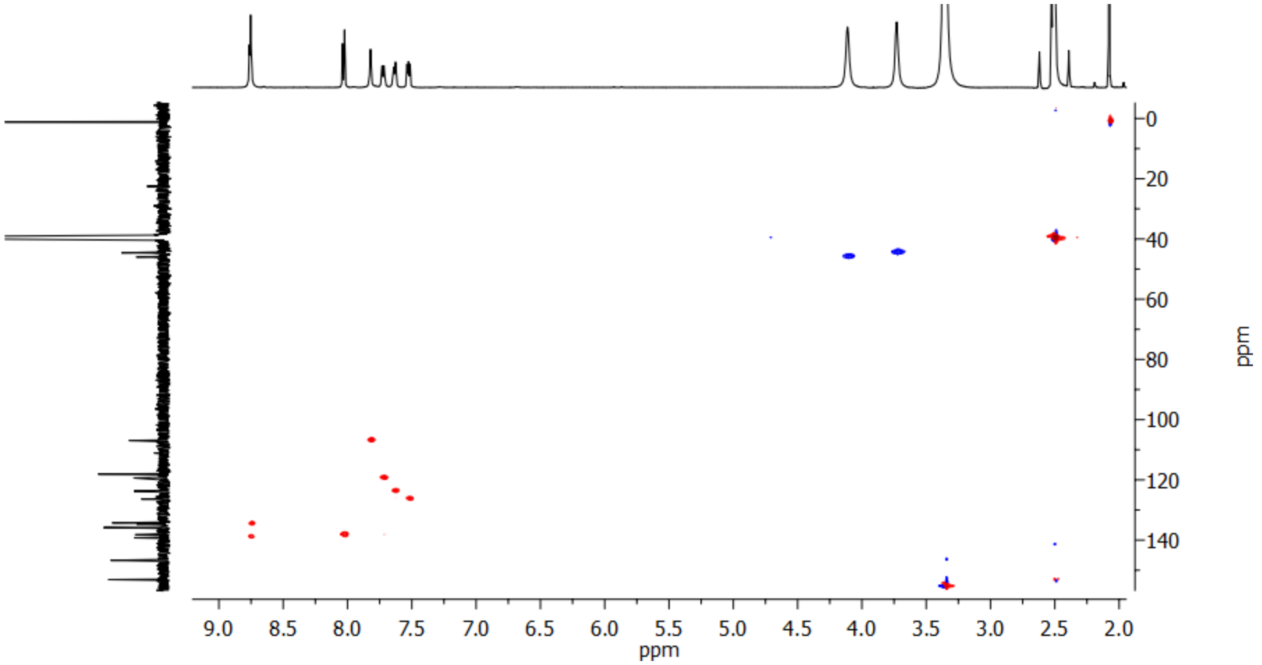


**Figure S60:** $^{1}H$ – $^{13}C$ HSQC spectrum (600 MHz, 298K, DMSO-$d_6$) of cage $[Pd_2(\mathbf{MB\text{-}P})_4](BF_4)_8$.

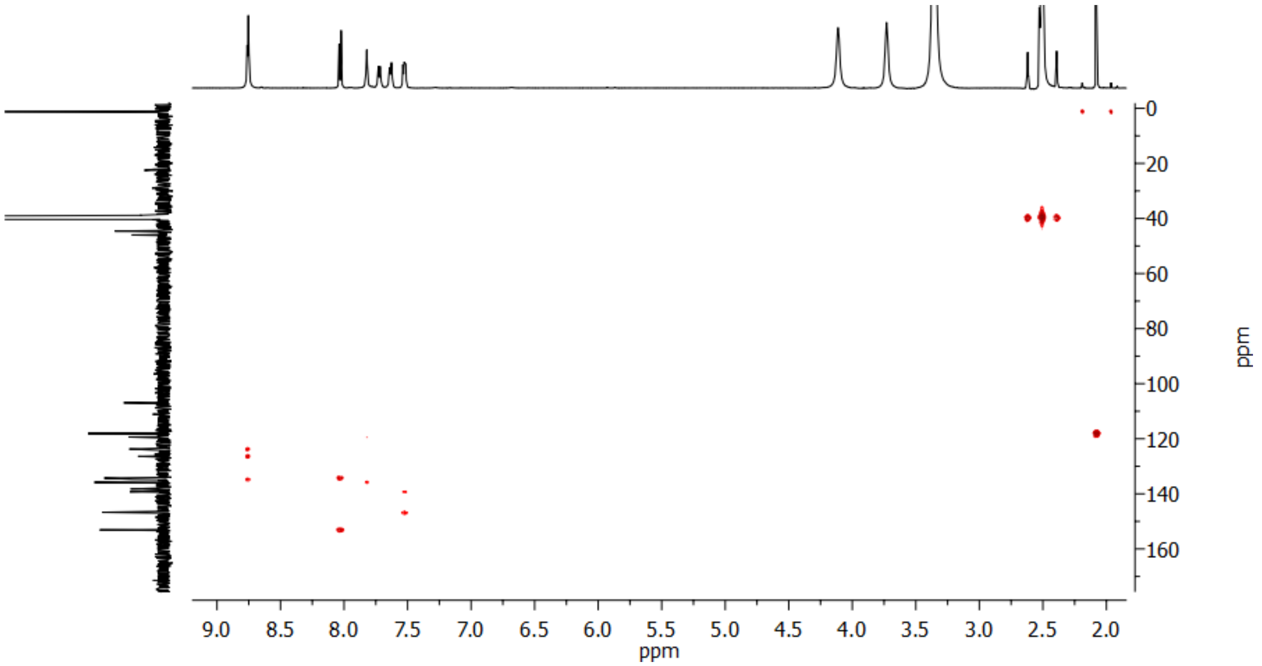


**Figure S61:** $^{1}H$ – $^{13}C$ HMBC spectrum (600 MHz, 298K, DMSO-$d_6$) of cage $[Pd_2($**MB-P**$)_4](BF_4)_8$.

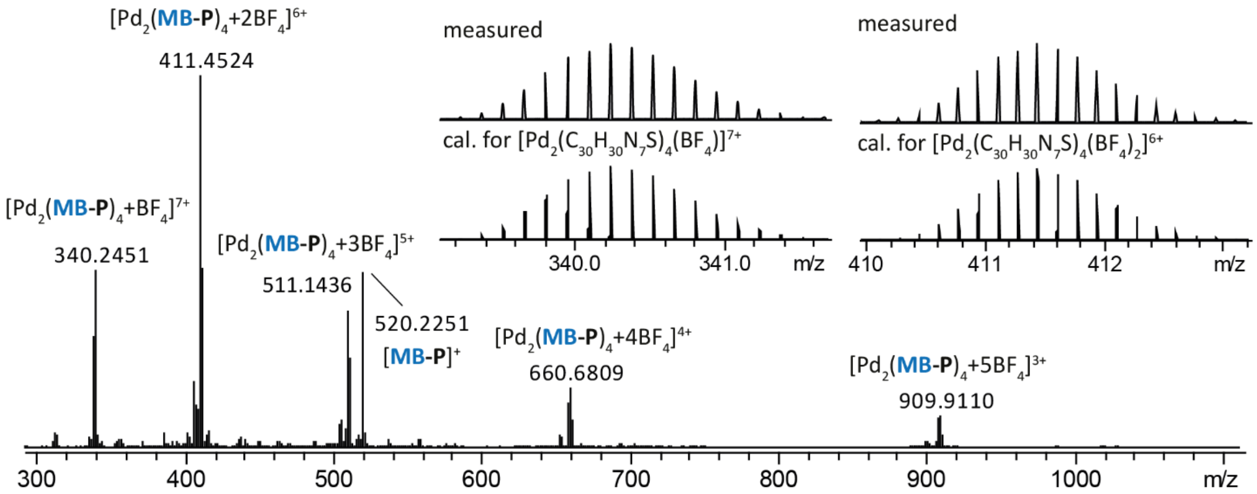


**Figure S62:** ESI-MS spectrum of $[Pd_2($**MB-P**$)_4+nBF_4]^{(8-n)+}$ with n=1-5. The observed and calculated isotopic pattern of $[Pd_2($**MB-P**$)_4+BF_4]^{7+}$ and $[Pd_2($**MB-P**$)_4+2BF_4]^{6+}$ are shown in the inset.

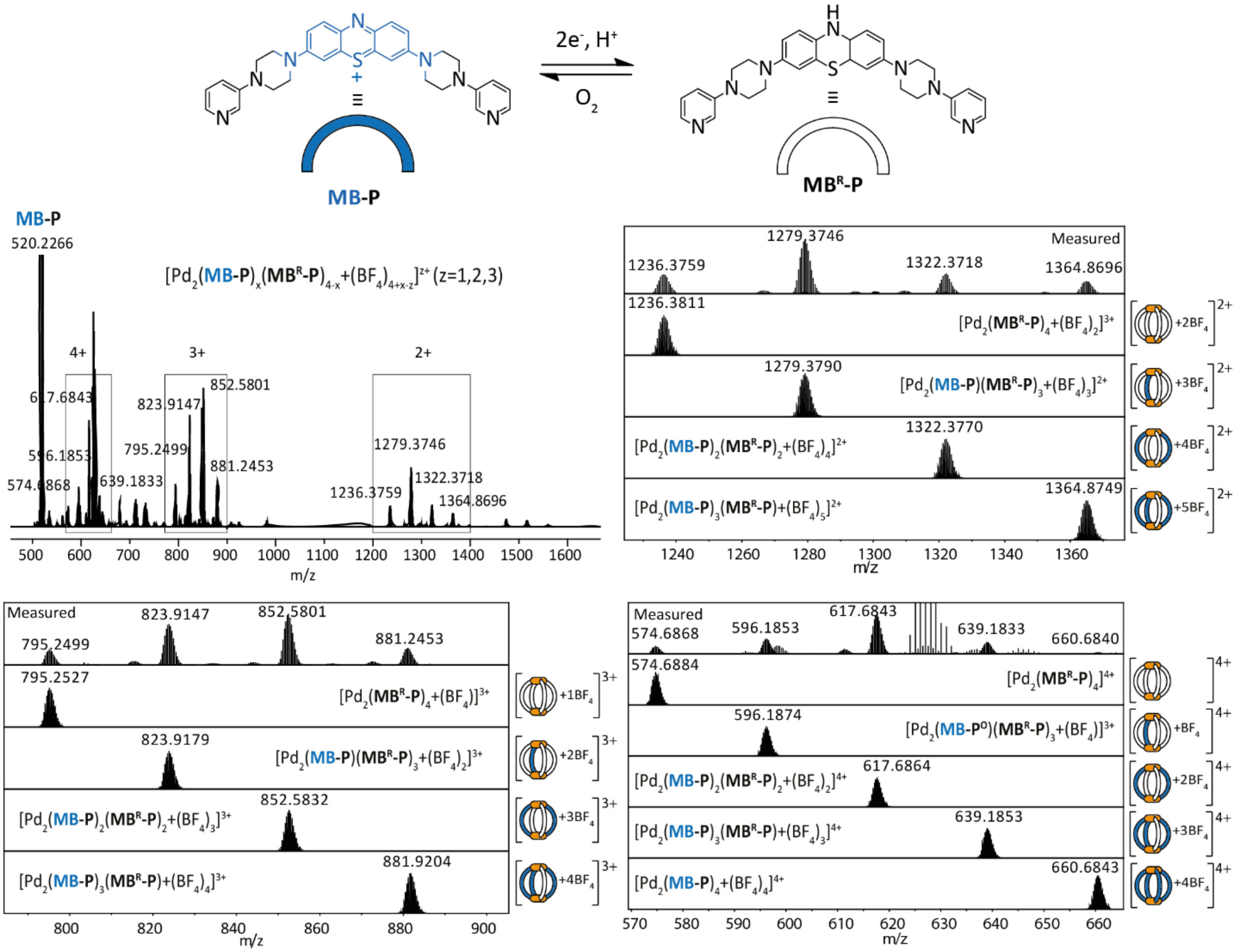


**Figure S63:** ESI-MS spectrum of $\{[Pd_2(\mathbf{MB\text{-}P})_x(\mathbf{MB^R\text{-}P})_{4-x}](BF_4)_{4+x-z}\}^{z+}$ with x=0-4 and z=2-4, obtained by treating the 0.7 mM DMSO solution of $[Pd_2(\mathbf{MB\text{-}P})_4](BF_4)_8$ with Zn powder, diluting with degassed acetonitrile and directly injected in the mass spectrometer. Top left, full spectrum with assignment of the peak at 520.2266 to ligand **MB-P**. Top right, zoom in the spectral area 1230-1380 m/z where the 2+ peaks are present; isotopic pattern simulations with formula and assignment are shown. Bottom left, zoom in the spectral area 785-910 m/z where the 3+ peaks are present; isotopic pattern simulations with formula and assignment are shown. Bottom right, zoom in the spectral area 570-670 m/z where the 4+ peaks are present; isotopic pattern simulations with formula and assignment are shown.

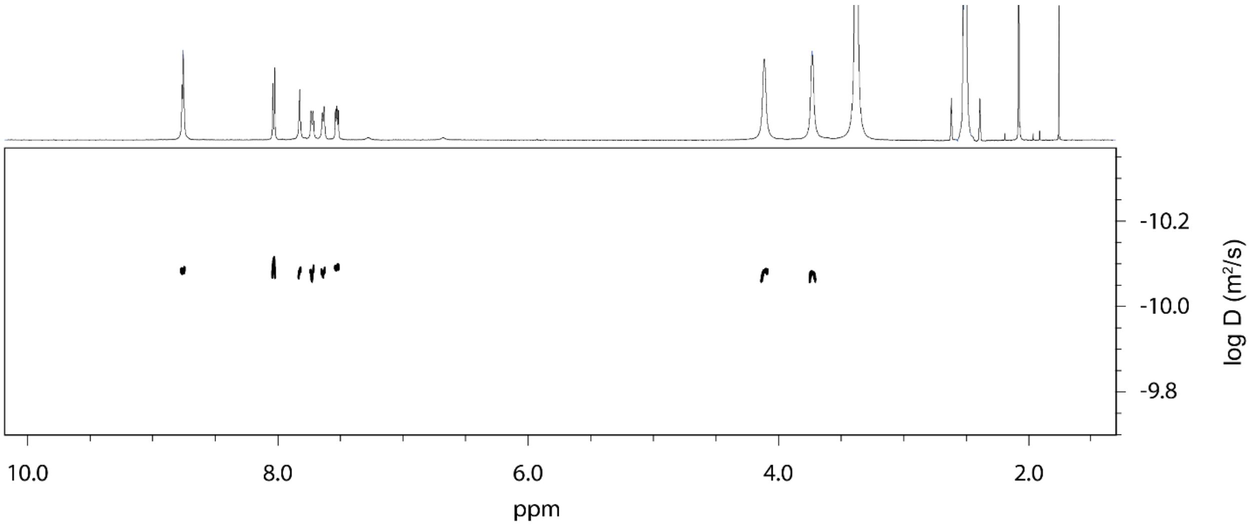


**Figure S64:** $^1$H DOSY spectrum (500 MHz, 298K, DMSO-$d_6$) of [$Pd_2$(**MB-P**)$_4$]($BF_4$)$_8$ (0.7 mM). Diffusion coefficient: $8.253\times10^{-11}$ $m^2s^{-1}$, log D=-10.083. Hydrodynamic radius= 13.304 Å.

### 3.2.3.2 [$Pd_2$(**MB-P**)$_4$]($NO_3$)$_8$

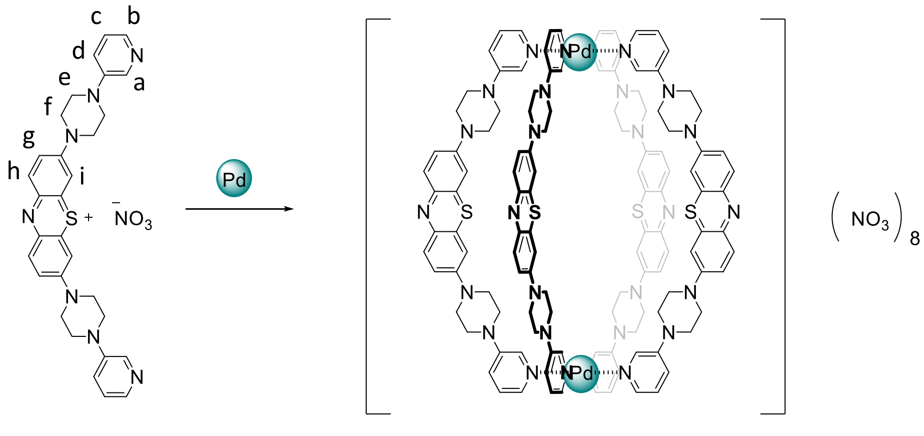


**MB-P**$NO_3$ [$Pd_2$(**MB-P**)$_4$]($NO_3$)$_8$

**Scheme S14:** Self-Assembly of cage [$Pd_2$(**MB-P**)$_4$]($NO_3$)$_8$.

A mixture of ligand **MB-P**$NO_3$ (450 µL of a 3.11 mM solution in DMSO-$d_6$) and Pd($NO_3$)$_2$ (50 µL of a 15 mM solution in DMSO-$d_6$) was heated at 70 °C for 15 min to afford a 0.7 mM solution of [$Pd_2$(**MB-P**)$_4$]($NO_3$)$_8$.

$^1$H NMR (600 MHz, 298 K, dimethyl sulfoxide-$d_6$) δ 8.95 (s, 1H, Ha), 8.73 (d, *J* = 5.5 Hz, 1H, Hb), 8.09 – 7.97 (m, 2H, Hh, Hi), 7.71 (d, *J* = 9.5 Hz, 1H, Hg), 7.62 (d, *J* = 8.8 Hz, 1H, Hd), 7.50 (dd, *J* = 8.6, 5.5 Hz, 1H, Hc), 4.20-4.06 (m, 4H, Hf), 3.85 – 3.63 (m, 4H, He).

$^{13}$C NMR (151 MHz, 298 K, dimethyl sulfoxide-$d_6$) δ 153.10 ($C^q$, **C**-Ch), 146.77 ($C^q$, **C**-Ci), 139.32 (Cb), 138.20 (Ch), 135.85 ($C^q$, Cg-**C**-Ci), 135.20 ($C^q$, Ca-**C**-Cd), 134.42 (Ca), 126.37 (Cc), 123.72 (Cd), 119.32 (Cg), 107.34 (Ci), 46.17 (Ce), 44.88 (Cf).

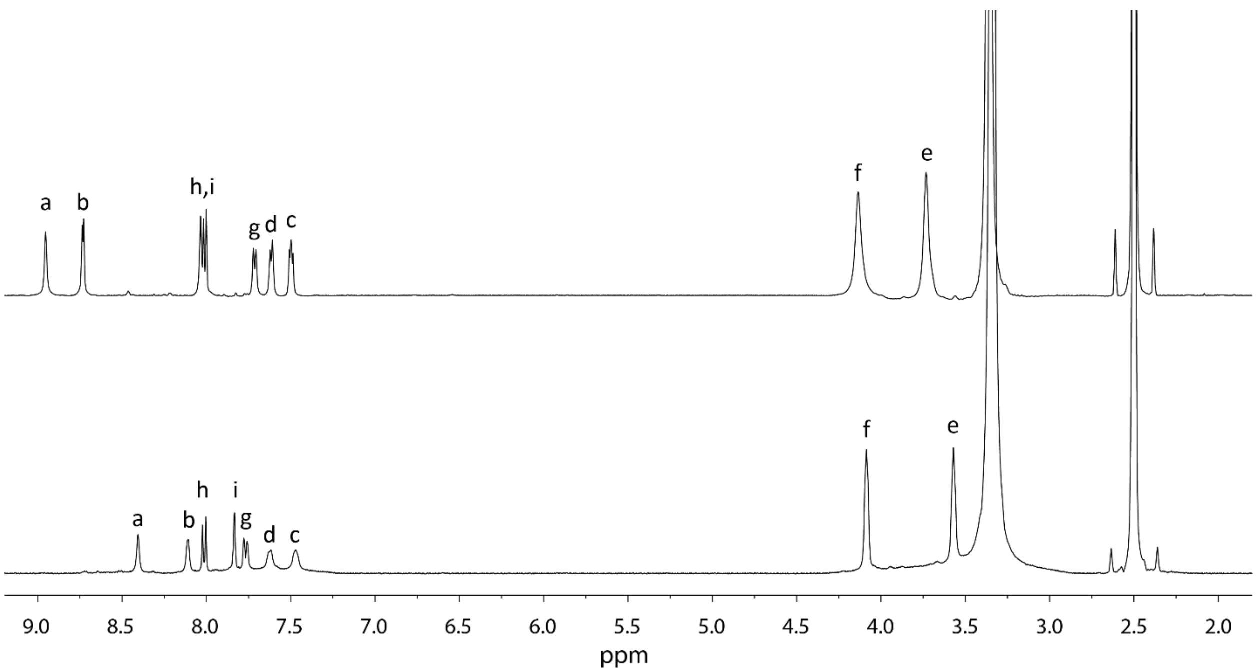


**Figure S65:** $^{1}$H NMR stacked spectra (600 MHz, 298K, DMSO-$d_6$) of ligand **MB-P**$NO_3$ (bottom) and the correspondent cage $[Pd_2($**MB-P**$)_4](NO_3)_8$ (top) upon addition of 0.5 equiv. of Pd(II) salt.

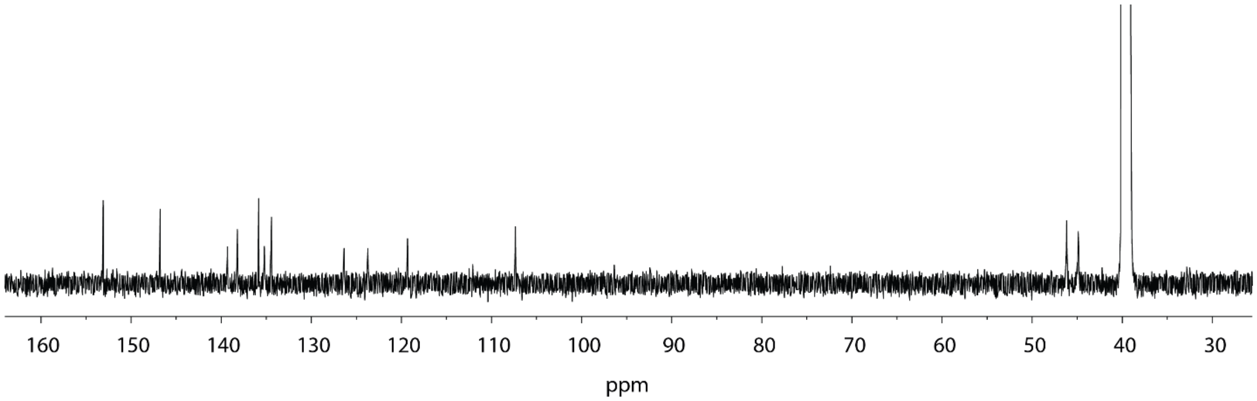


**Figure S66:** $^{13}$C NMR spectrum (151 MHz, 298K, DMSO-$d_6$) of cage $[Pd_2($**MB-P**$)_4](NO_3)_8$.

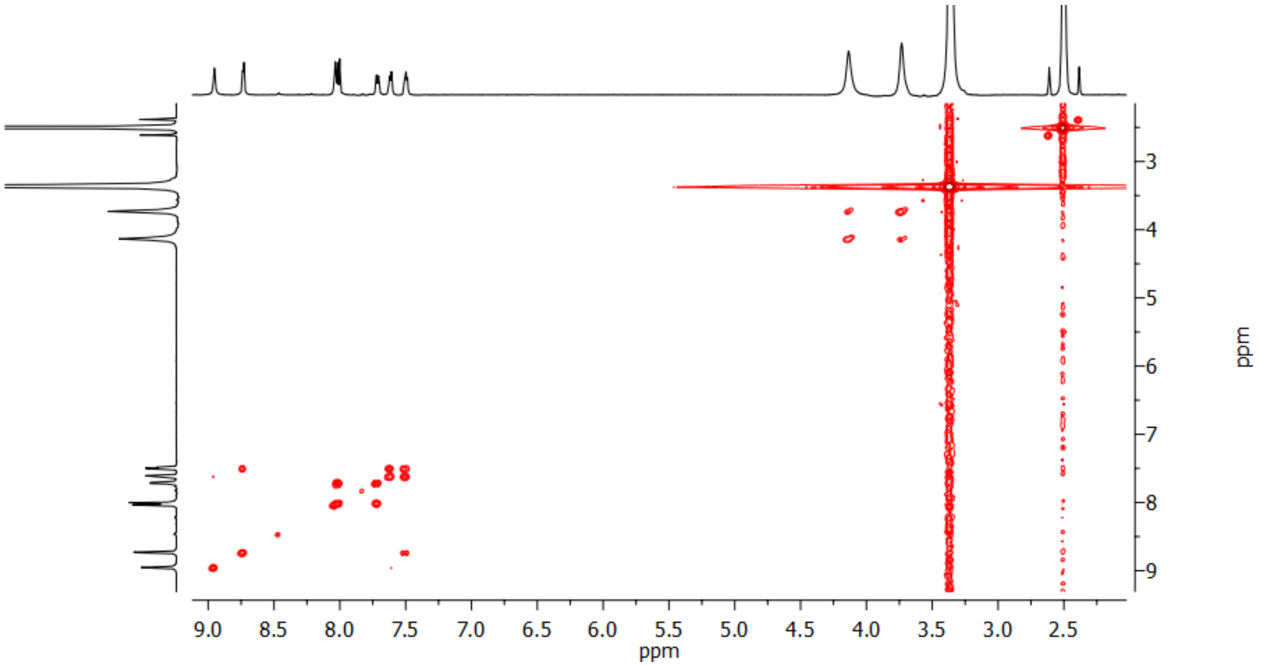


**Figure S67:** $^{1}H$ – $^{1}H$ COSY spectrum (600 MHz, 298K, DMSO-$d_6$) of cage $[Pd_2($**MB-P**$)_4](NO_3)_8$.

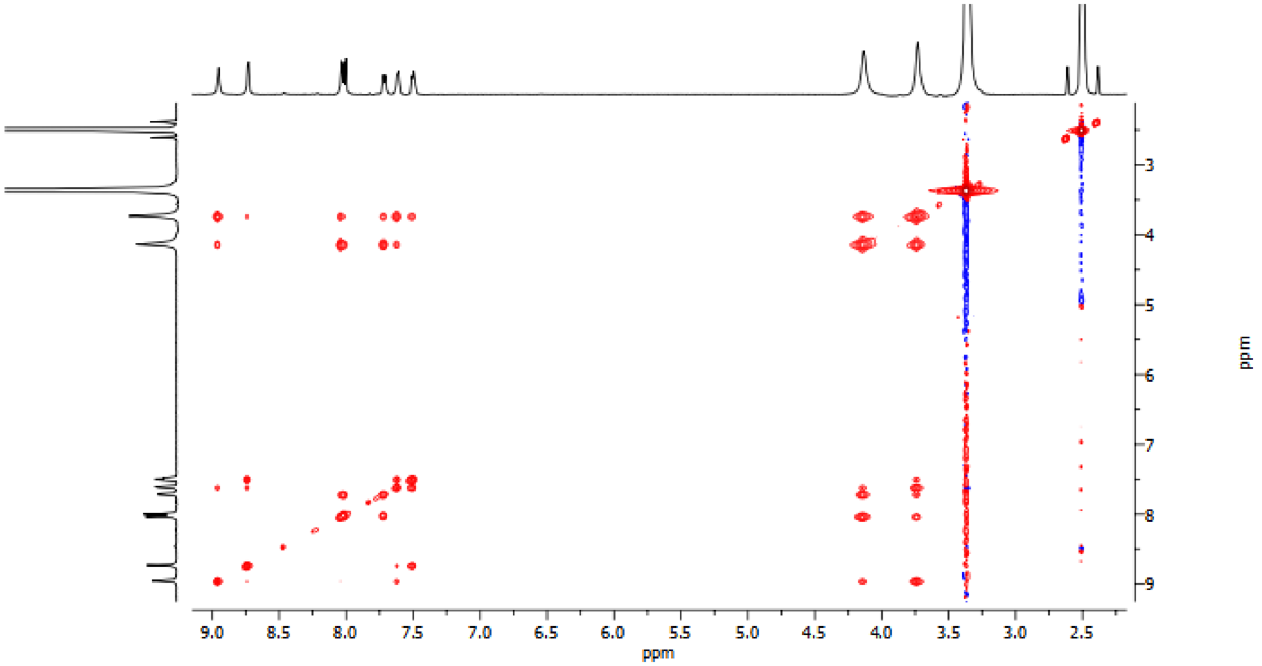


**Figure S68:** $^{1}H$ – $^{1}H$ NOESY spectrum (600 MHz, 298K, DMSO-$d_6$) of cage $[Pd_2($**MB-P**$)_4](NO_3)_8$.

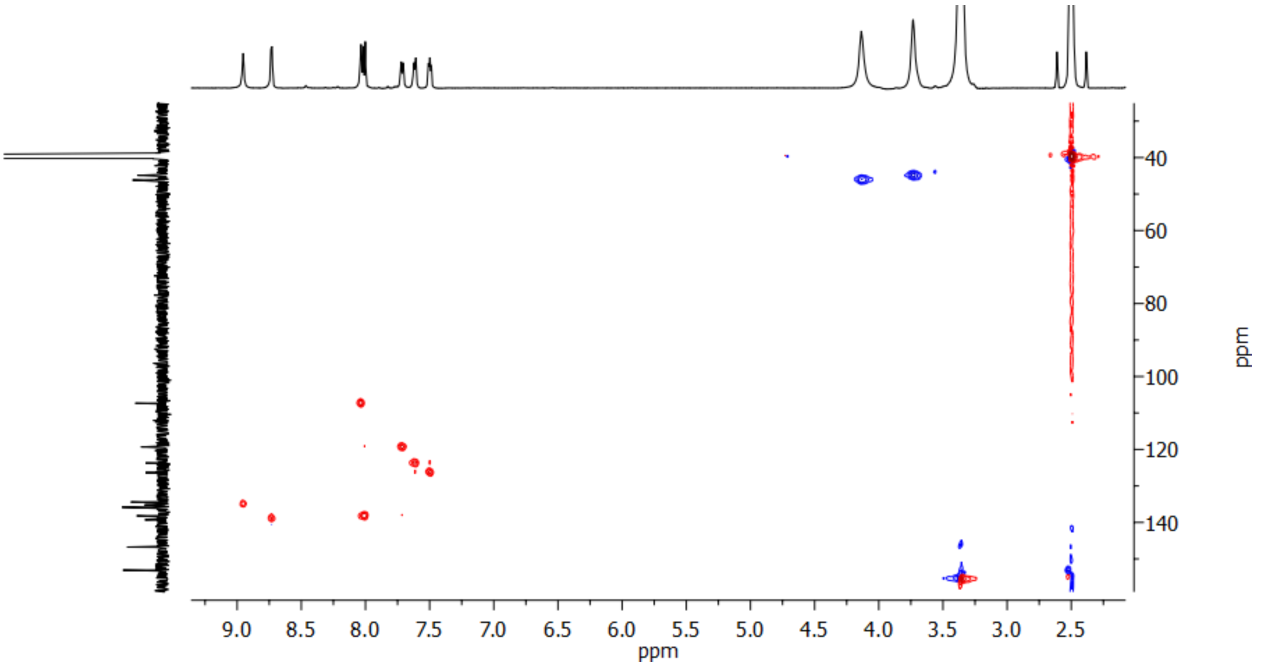


**Figure S69:** $^{1}H$ – $^{13}C$ HSQC spectrum (600 MHz, 298K, DMSO-$d_6$) of cage $[Pd_2($**MB-P**$)_4](NO_3)_8$.

A mixture of ligand **MB-P** in $D_2O$ (450 µL of a 3.11 mM solution) and $Pd(NO_3)_2$ (50 µL of a 15 mM solution in DMSO-$d_6$) was heated at 70 °C for 15 min to afford a 0.7 mM solution of $[Pd_2($**MB-P**$)_4](NO_3)_8$.

$^{1}H$ NMR (500 MHz, Deuterium Oxide) δ 8.30 – 8.25 (m, 1H, Ha), 8.11 (d, J = 5.4 Hz, 1H, Hb), 8.04 (d, *J* = 9.7 Hz, 1H, Hh), 7.99 (d, *J* = 8.4 Hz, 1H, Hd), 7.84 (dd, *J* = 8.9, 5.4 Hz, 1H, Hc), 7.65 (d, *J* = 9.8 Hz, 1H, Hg), 7.53 (s, 1H, Hi), 4.17 – 4.10 (m, 2H), 3.83 – 3.72 (m, 4H).

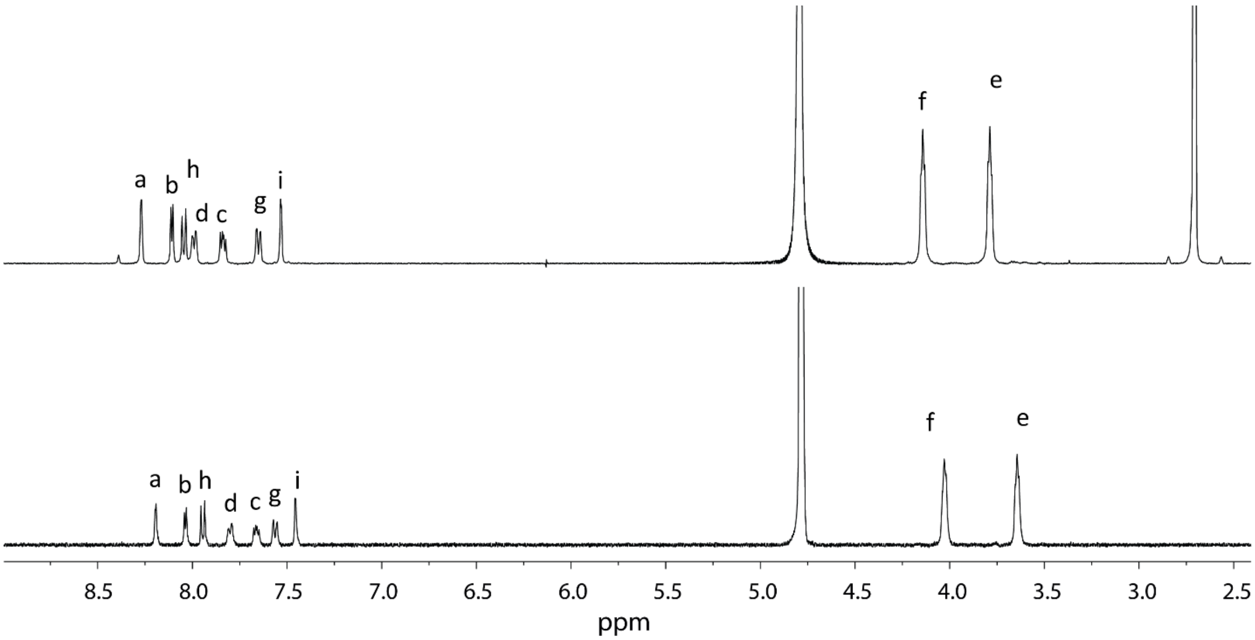


**Figure S70:** $^{1}H$ NMR stacked spectra (500 MHz, 298K, $D_2O$) of ligand **MB-P**$NO_3$ (bottom) and the correspondent cage $[Pd_2($**MB-P**$)_4](NO_3)_8$ (top) upon addition of 0.5 equiv. of Pd(II) salt.

### 3.2.4 $[Pd_2(\textbf{CV-P})_4](NO_3)_8$

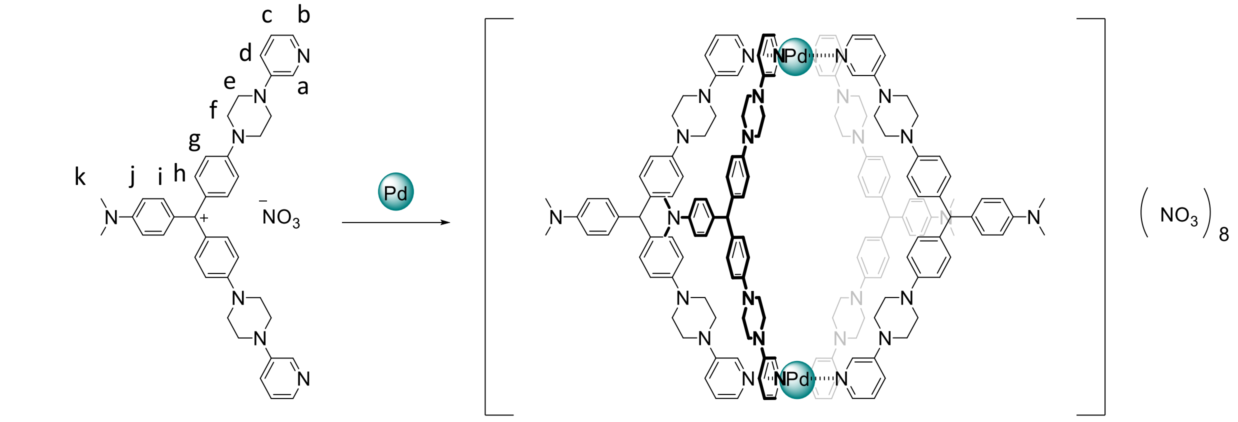


**CV-P** $[Pd_2(\textbf{CV-P})_4](NO_3)_8$

**Scheme S15:** Self-Assembly of cage $[Pd_2(\textbf{CV-P})_4](NO_3)_8$.

A mixture of ligand **CV-P** (450 µL of a 3.11 mM solution in DMSO-$d_6$) and $Pd(NO_3)_2$ (50 µL of a 15 mM solution in DMSO-$d_6$) was heated at 70 °C for 15 min to afford a 0.7 mM solution of $[Pd_2(\textbf{CV-P})_4](NO_3)_8$.

$^1$H NMR (500 MHz, 298 K, dimethyl sulfoxide-$d_6$) δ 8.92 (s, 1H, Ha), 8.70 (d, *J* = 4.9 Hz, 1H, Hb), 7.47 (dd, *J* = 20.9, 7.9 Hz, 2H, Hd, Hc), 7.22 (m, 5H, Hg, Hh, Hi), 6.96 (d, *J* = 9.2 Hz, 1H, Hj), 3.98 – 3.72 (m, 4H, Hf), 3.72 – 3.55 (m, 4H, He), 3.18 (s, 3H, Hk).

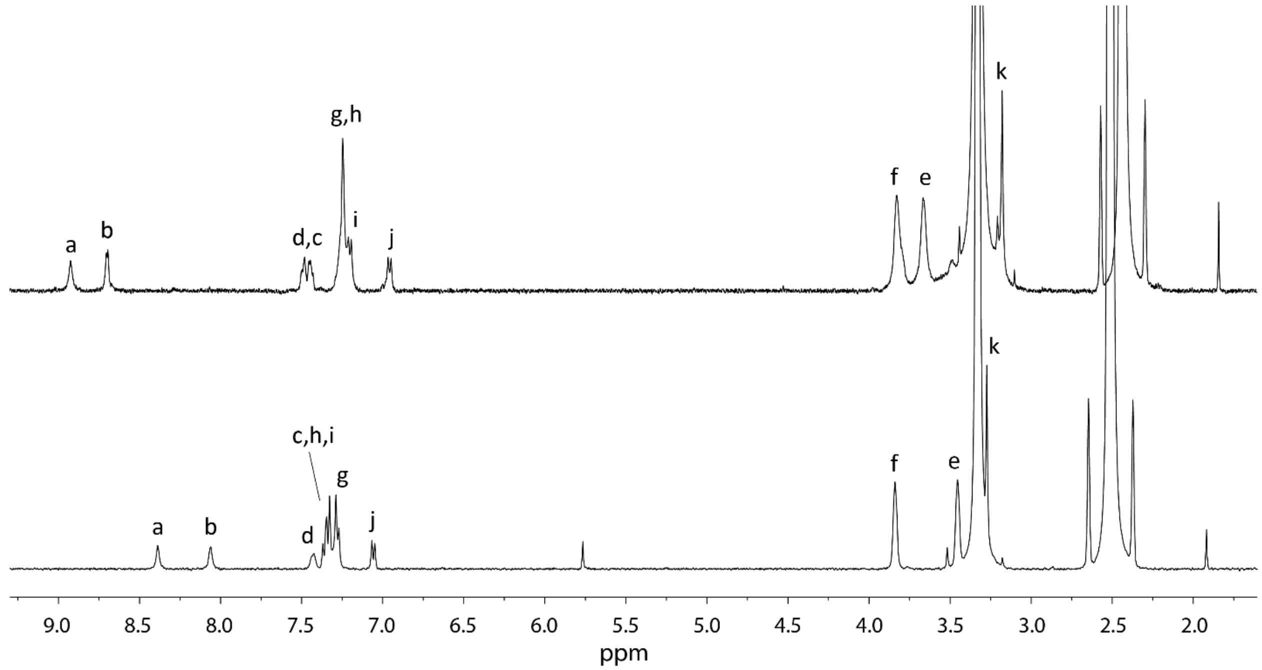


**Figure S71:** $^1$H NMR stacked spectra (600 MHz, 298K, DMSO-$d_6$) of ligand **CV-P** (bottom) and the correspondent cage $[Pd_2(\textbf{CV-P})_4](NO_3)_8$ (top) upon addition of 0.5 equiv. of Pd(II) salt.

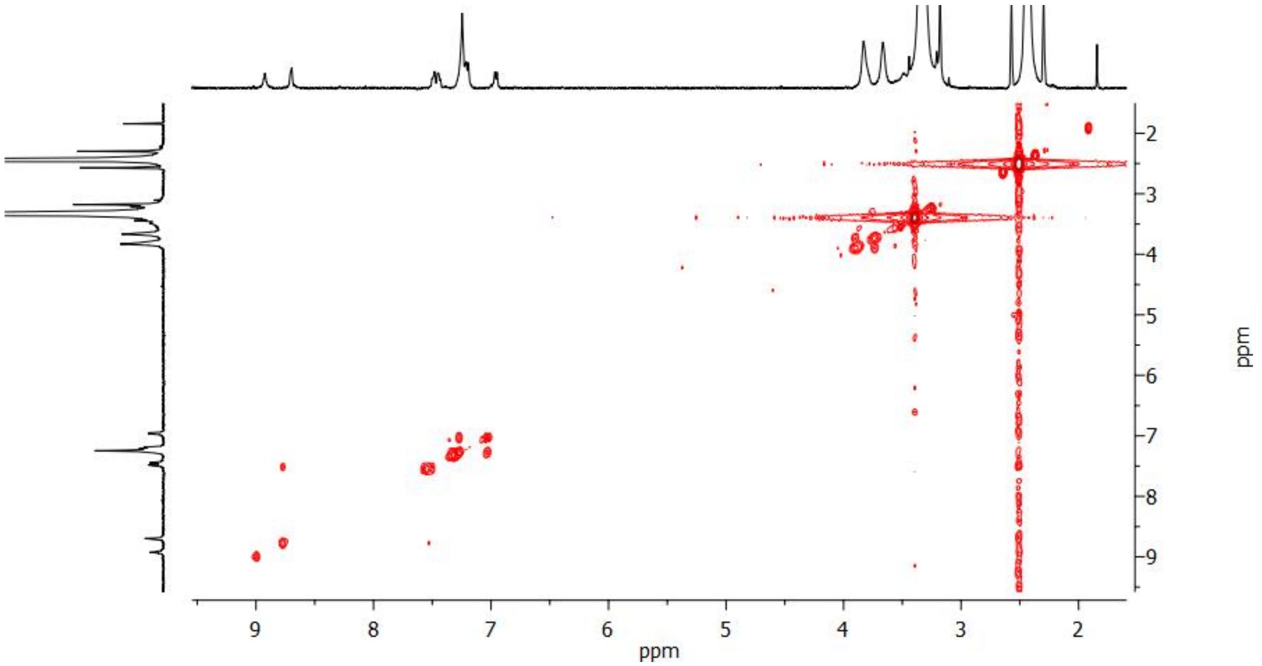


**Figure S72:** $^{1}H$ – $^{1}H$ COSY spectrum (600 MHz, 298K, DMSO-$d_6$) of cage [$Pd_2$(**CV-P**)$_4$]($NO_3$)$_8$.

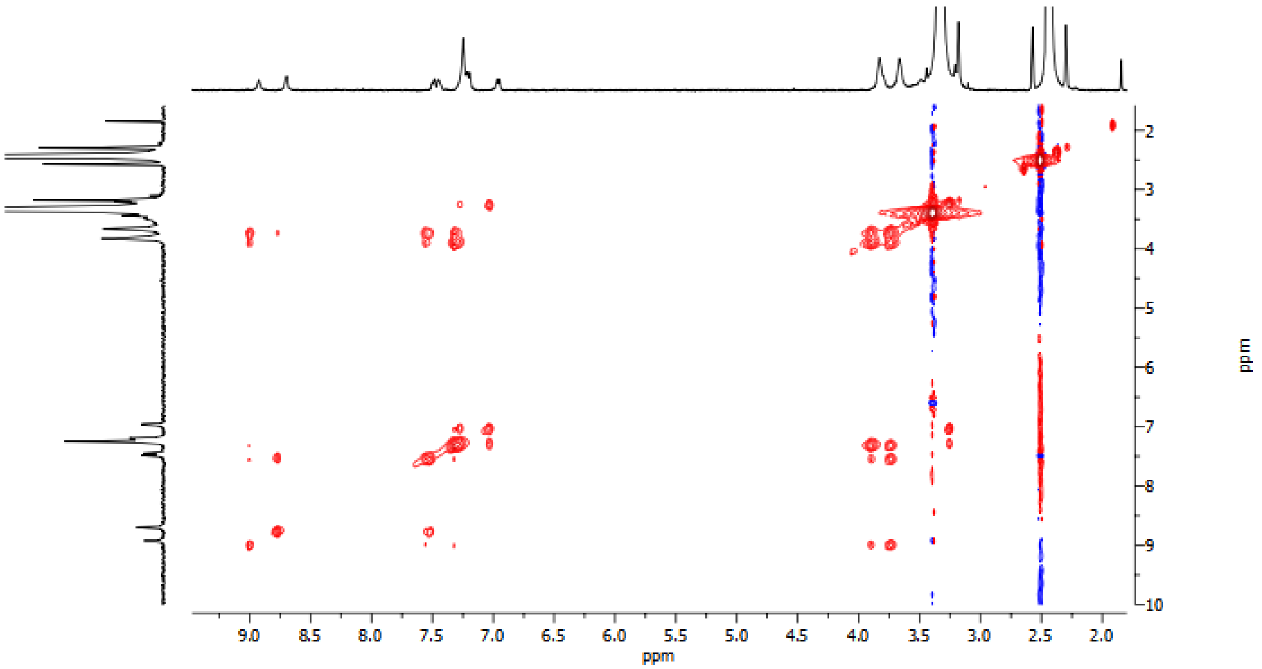


**Figure S73:** $^{1}H$ – $^{1}H$ NOESY spectrum (600 MHz, 298K, DMSO-$d_6$) of cage [$Pd_2$(**CV-P**)$_4$]($NO_3$)$_8$.

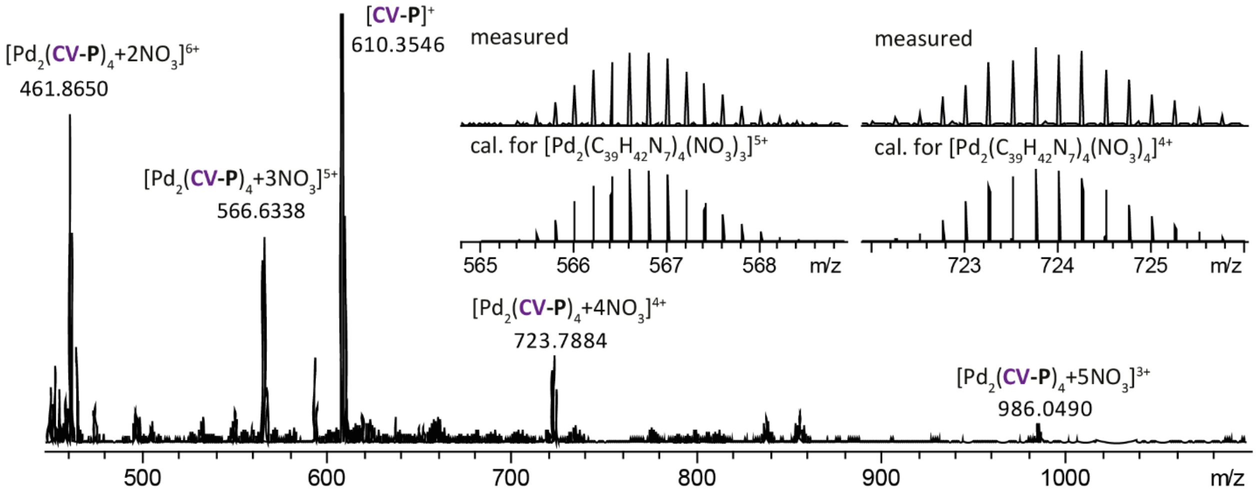


**Figure S74:** ESI-MS spectrum of $[Pd_2(\textbf{CV-P})_4{+}nNO_3]^{(8-n)+}$ with n=2-5. The observed and calculated isotopic patterns of $[Pd_2(\textbf{CV-P})_4{+}3NO_3]^{5+}$ and $[Pd_2(\textbf{CV-P})_4{+}4NO_3]^{4+}$ are shown in the inset.

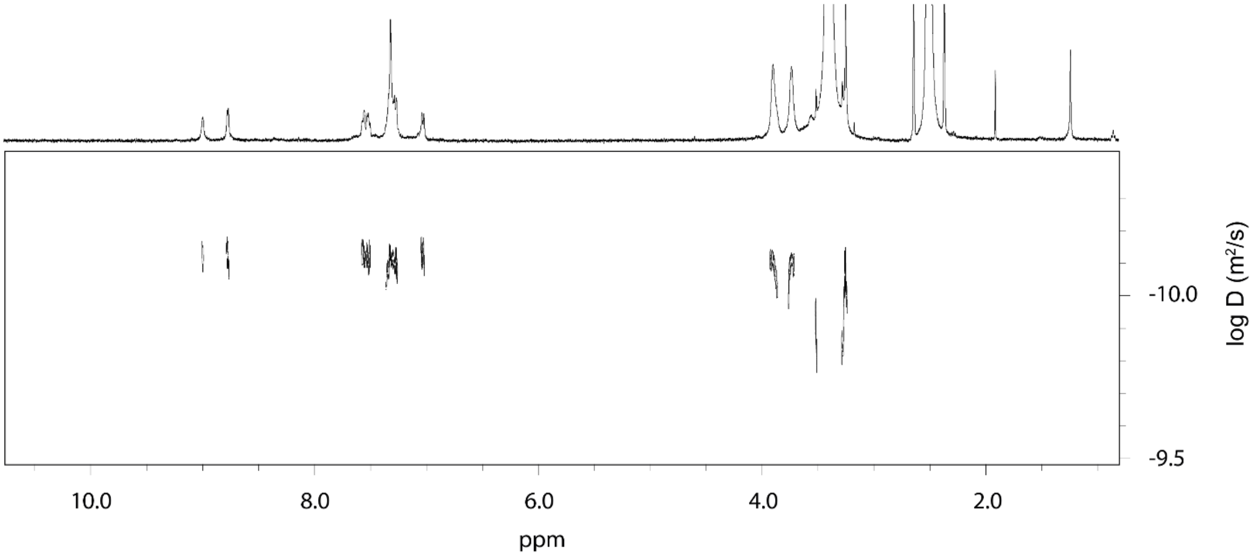


**Figure S75:** $^{1}$H DOSY spectrum (500 MHz, 298K, DMSO-$d_6$) of $[Pd_2(\textbf{CV-P})_4](NO_3)_8$ (0.7 mM). Diffusion coefficient: $7.990\times10^{-11}$ $m^2s^{-1}$, log D=-10.098. Hydrodynamic radius= 13.74 Å.

### 3.2.5 [$Pd_2$(RE-P)$_4$]($EtSO_4$)$_4$($BF_4$)$_4$

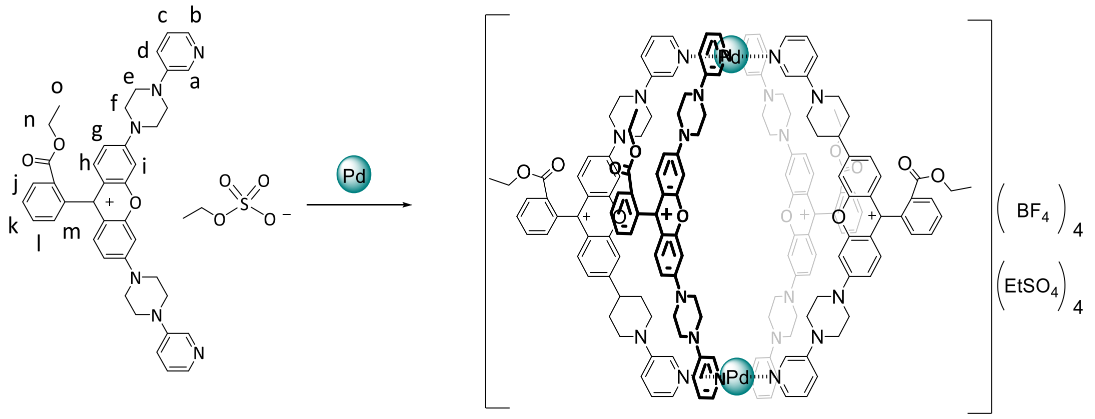

**RE-P** [$Pd_2$(**RE-P**)$_4$]($EtSO_4$)$_4$($BF_4$)$_4$

**Scheme S16:** Self-Assembly of cage [$Pd_2$(**RE-P**)$_4$]($EtSO_4$)$_4$($BF_4$)$_4$.

A mixture of ligand **RE-P** (450 µL of a 3.11 mM solution in DMSO-$d_6$) and [Pd($CH_3CN$)$_4$]($BF_4$)$_2$ (50 µL of a 15 mM solution in DMSO-$d_6$) was heated at 70 °C for 15 min to afford a 0.7 mM solution of [$Pd_2$(**RE-P**)$_4$]($EtSO_4$)$_4$($BF_4$)$_4$.

$^1$H NMR (600 MHz, dimethyl sulfoxide-$d_6$) δ 9.00 (s, 1H), 8.77 (d, *J* = 5.5 Hz, 1H), 8.26 (d, *J* = 7.8 Hz, 1H), 7.92 (t, *J* = 7.6 Hz, 0H), 7.86 (t, *J* = 7.4 Hz, 1H), 7.57 (d, *J* = 8.9 Hz, 1H), 7.53 – 7.42 (m, 1H), 7.34 (t, *J* = 8.3 Hz, 2H), 7.15 – 7.05 (m, 1H), 3.98 (s, 6H), 3.94 – 3.87 (m, 1H), 3.81 (q, *J* = 7.1 Hz, 4H), 3.71 (s, 3H), 1.15 (t, *J* = 7.1 Hz, 5H), 0.96 – 0.81 (m, 2H).

$^{13}$C NMR (151 MHz, dimethyl sulfoxide-$d_6$) δ 164.57(C$^q$), 157.46 (C$^q$), 156.62 (C$^q$), 146.77 (C$^q$), 146.77 (C$^q$), 139.20 (Cb), 135.46 (Ca), 133.24 (Cl), 132.82 (Ch), 131.02 (Cj and Ck), 129.57 (Cm), 126.42 (Cc), 123.43 (Cd), 118.08 (acetonitrile?), 115.38 (Cg), 113.92 (C$^q$), 96.97 (Ci), 61.28 (-**C**$H_2$ counterion), 61.04 (Cn), 45.80 (Cf), 44.76 (Ce), 15.21(-**C**$H_3$ counterion), 13.51 (Co).

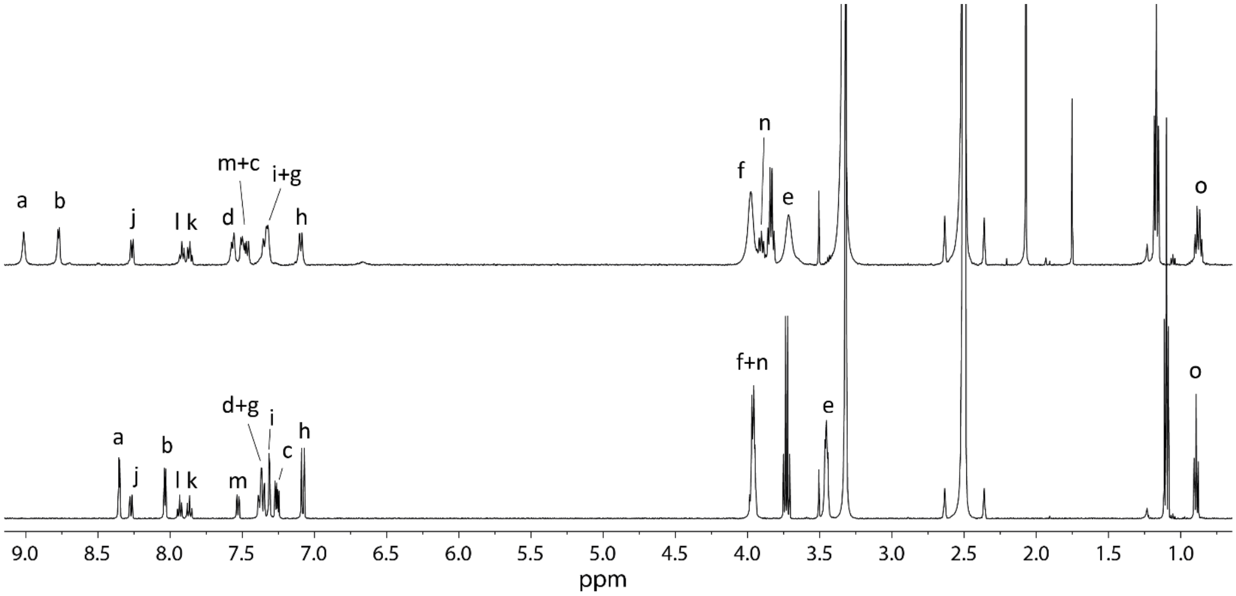


**Figure S76:** $^1$H NMR stacked spectra (600 MHz, 298K, DMSO-$d_6$) of ligand **RE-P** (bottom) and the correspondent cage [$Pd_2$(**RE-P**)$_4$]($EtSO_4$)$_4$($BF_4$)$_4$ (top) upon addition of 0.5 equiv. of Pd(II) salt.

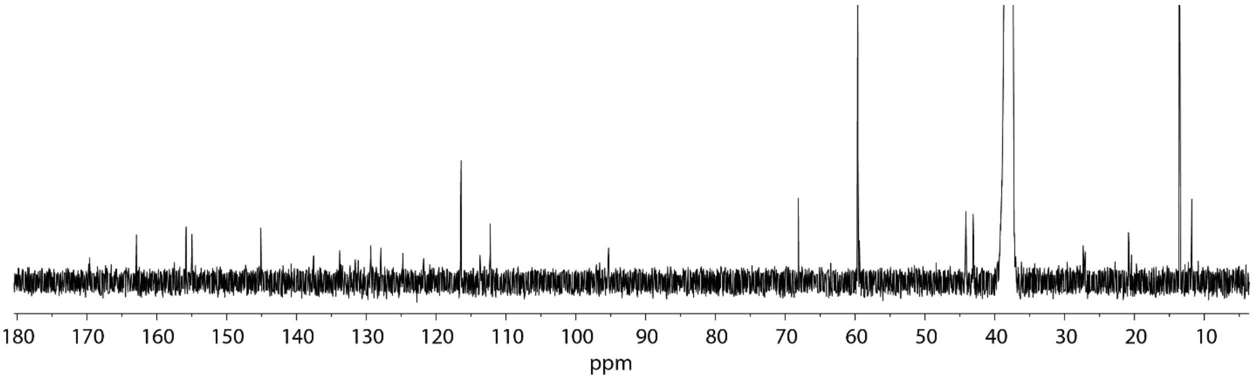


**Figure S77:** $^{13}C$ NMR spectrum (151 MHz, 298K, DMSO-*d*$_6$) of cage $[Pd_2(\mathbf{RE\text{-}P})_4](EtSO_4)_4(BF_4)_4$.

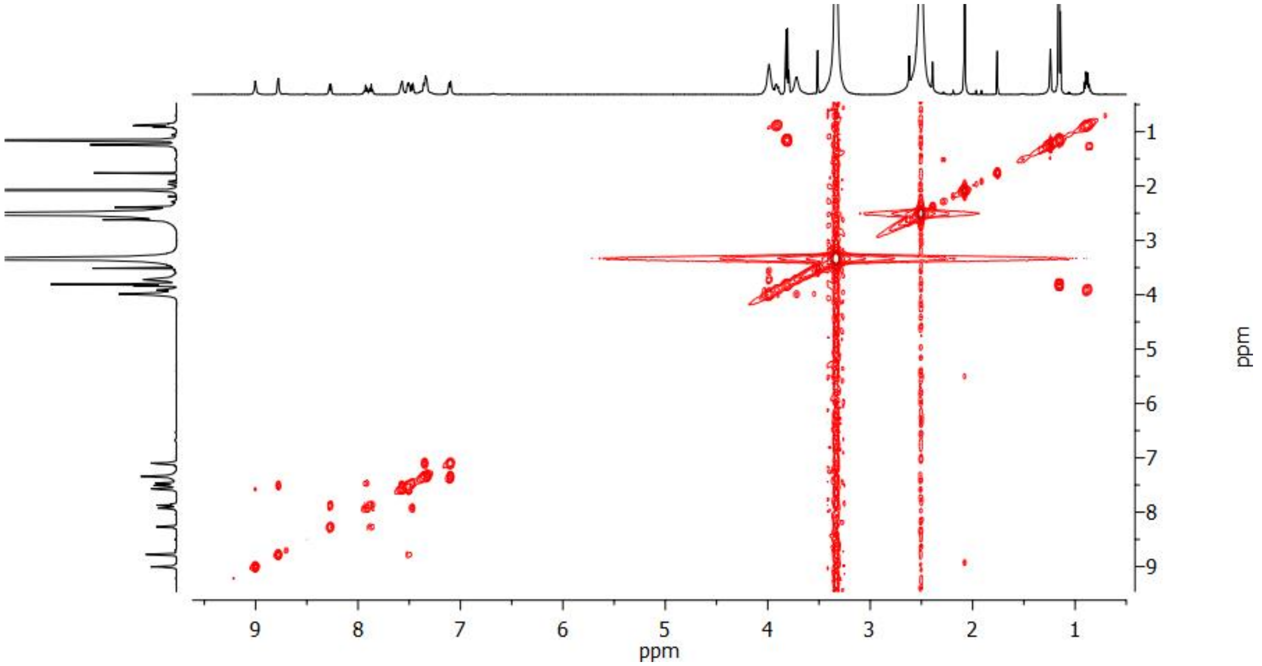


**Figure S78:** $^{1}H$ – $^{1}H$ COSY spectrum (600 MHz, 298K, DMSO-*d*$_6$) of cage $[Pd_2(\mathbf{RE\text{-}P})_4](EtSO_4)_4(BF_4)_4$.

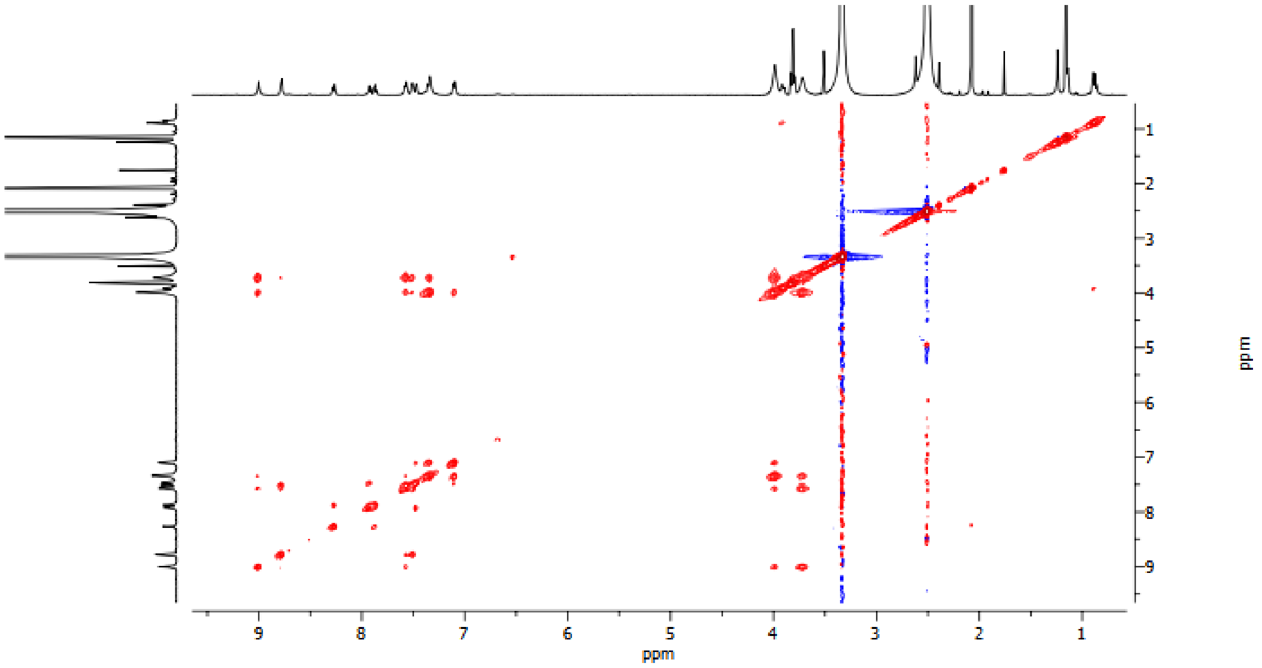


**Figure S79:** $^{1}H$ – $^{1}H$ NOESY spectrum (600 MHz, 298K, DMSO-$d_6$) of cage $[Pd_2($**RE-P**$)_4](EtSO_4)_4(BF_4)_4$.

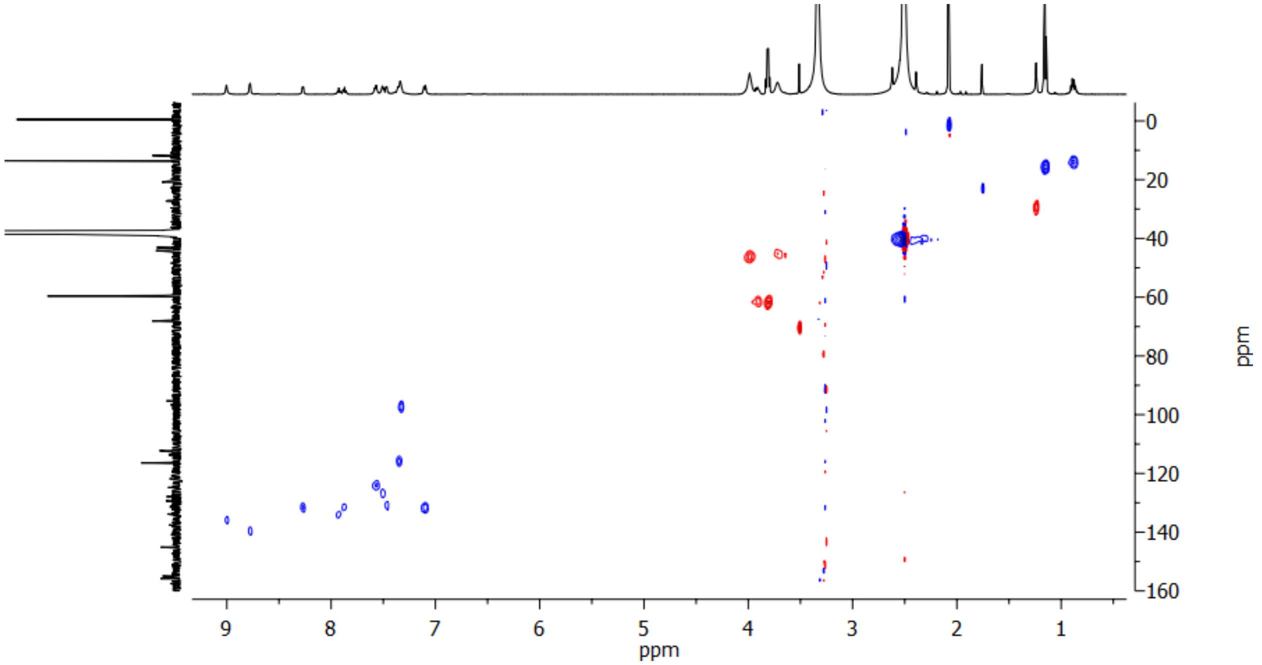


**Figure S80:** $^{1}H$ – $^{13}C$ HSQC spectrum (600 MHz, 298K, DMSO-$d_6$) of cage $[Pd_2($**RE-P**$)_4](EtSO_4)_4(BF_4)_4$.

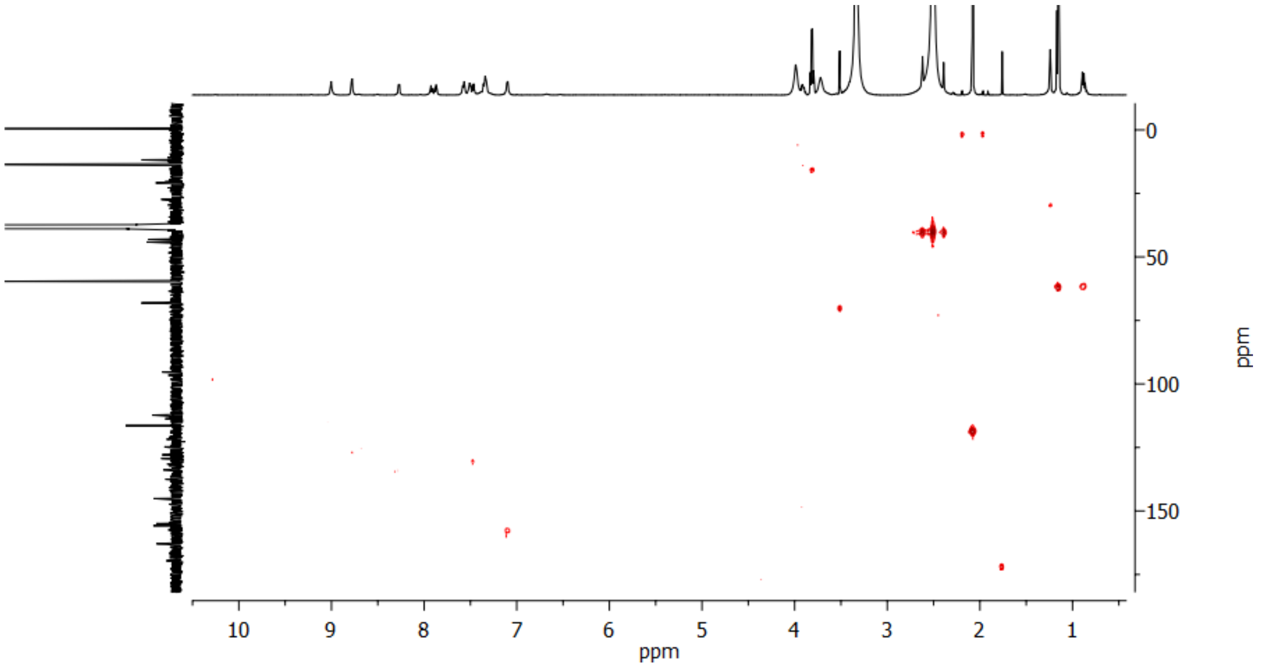


**Figure S81:** $^{1}H - ^{13}C$ HMBC spectrum (600 MHz, 298K, DMSO-$d_6$) of cage $[Pd_2(\mathbf{RE\text{-}P})_4](EtSO_4)_4(BF_4)_4$.

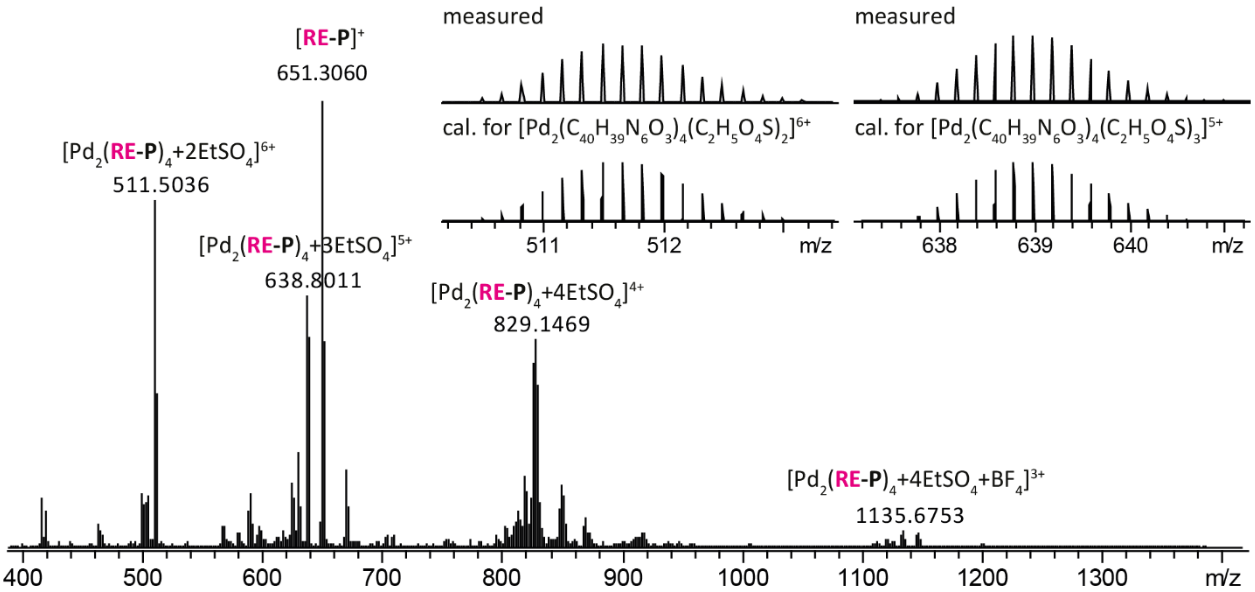


**Figure S82:** ESI-MS spectrum of $[Pd_2(\mathbf{RE\text{-}P})4{+}nEtSO_4{+}mBF_4]^{(8-n)+}$ with n=2-4 and m=0,1. The observed and calculated isotopic patterns of $[Pd_2(\mathbf{RE\text{-}P})_4{+}2EtSO_4]^{6+}$ and $[Pd_2(\mathbf{RE\text{-}P})_4{+}3EtSO_4]^{5+}$ are shown in the inset.

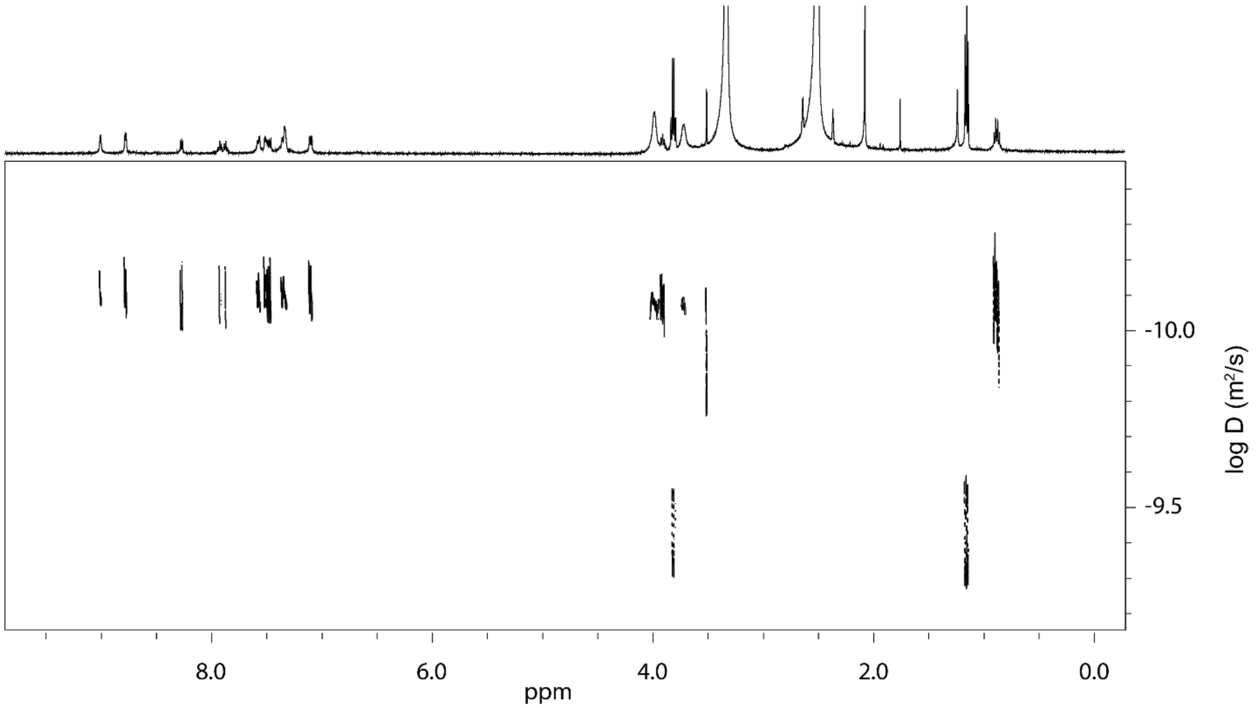


**Figure S83:** $^{1}$H DOSY spectrum (500 MHz, 298K, DMSO-$d_6$) of $[Pd_2($**RE-P**$)_4](EtSO_4)_4(BF_4)_4$ (0.7 mM). Diffusion coefficient: $8.232\times10^{-10}$ $m^2s^{-1}$, log D=-10.085. Hydrodynamic radius=13.54 Å.

### 3.2.6 $[Pd_2(\mathbf{MK\text{-}Q})_4](BF_4)_4$

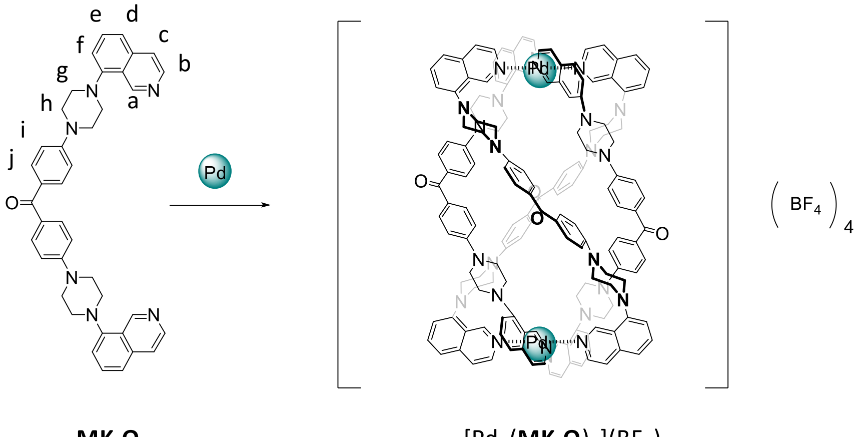


**MK-Q** $[Pd_2($**MK-Q**$)_4](BF_4)_4$

**Scheme S17:** Self-Assembly of helicate $[Pd_2($**MK-Q**$)_4](BF_4)_4$.

A mixture of ligand **MK-Q** (450 µL of a 3.11 mM solution in DMSO-$d_6$) and $[Pd(CH_3CN)_4](BF_4)_2$ (50 µL of a 15 mM solution in DMSO-$d_6$) was heated at 70 °C for 15 min to afford a 0.7 mM solution of $[Pd_2($**MK-Q**$)_4](BF_4)_4$.

$^{1}$H NMR (500 MHz, 298 K, dimethyl sulfoxide-$d_6$) δ 9.27 (s, 1H, Ha), 8.52 (d, $J$ = 6.5 Hz, 1H, Hb), 8.12 – 8.05 (m, 2H, Hc, He), 7.85 (d, $J$ = 8.3 Hz, 1H, Hd), 7.58 (d, $J$ = 8.4 Hz, 2H, Hj), 7.43 (d, $J$ = 7.7 Hz, 1H, Hf), 6.62 (d, $J$ = 8.4 Hz, 2H, Hi). He and Hf broad.

$^{13}C$ NMR (176 MHz, 298 K, dimethyl sulfoxide-$d_6$) δ 171.40 (C=O), 153.56 (Ca), 151.12 ($C^q$, **C**-Ci), 149.61 ($C^q$, Cd-**C**-Cc), 143.20 (Cb), 137.30 ($C^q$, **C**-Ca), 135.26 (Ce), 130.87 (Cj), 129.08 ($C^q$, **C**-Ci), 124.72 (Cc), 123.25 ($C^q$, C-Cf), 121.94 (Cd), 119.17 (Cf), 118.05 (*$CH_3CN$*), 114.74 (Ci), 45.77 (Ch, Cg).

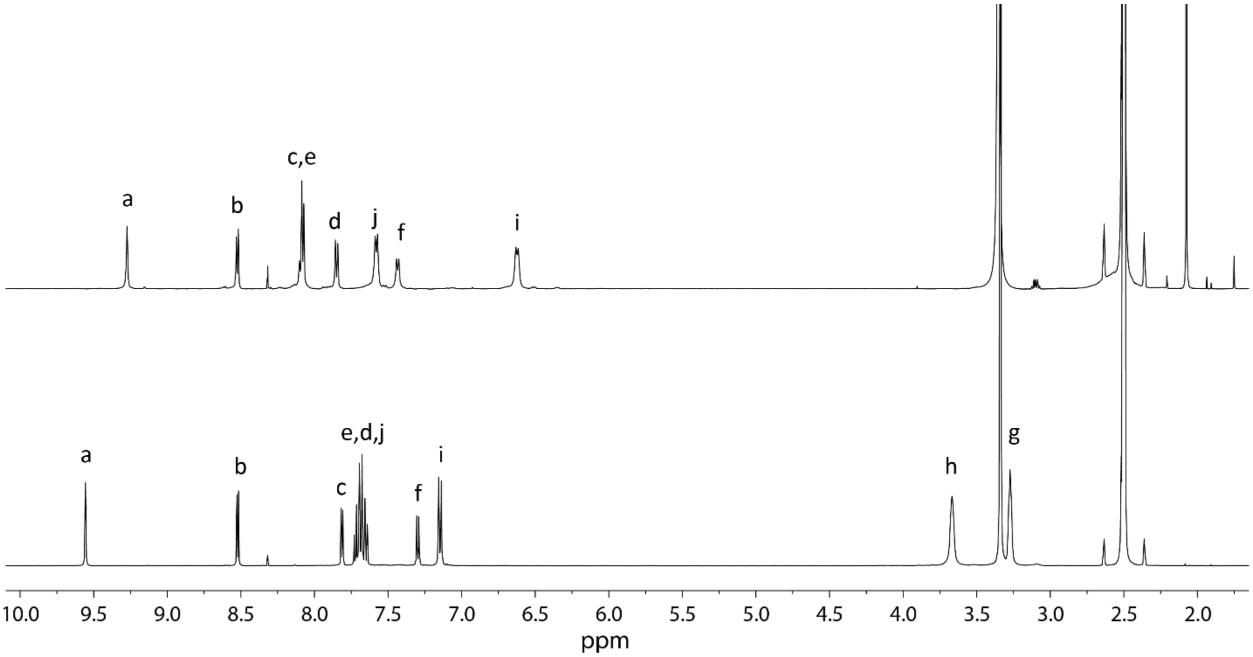


**Figure S84:** $^{1}H$ NMR stacked spectra (600 MHz, 298K, DMSO-$d_6$) of ligand **MK-Q** (bottom) and the correspondent cage $[Pd_2($**MK-Q**$)_4](BF_4)_4$ (top) upon addition of 0.5 equiv. of Pd(II) salt.

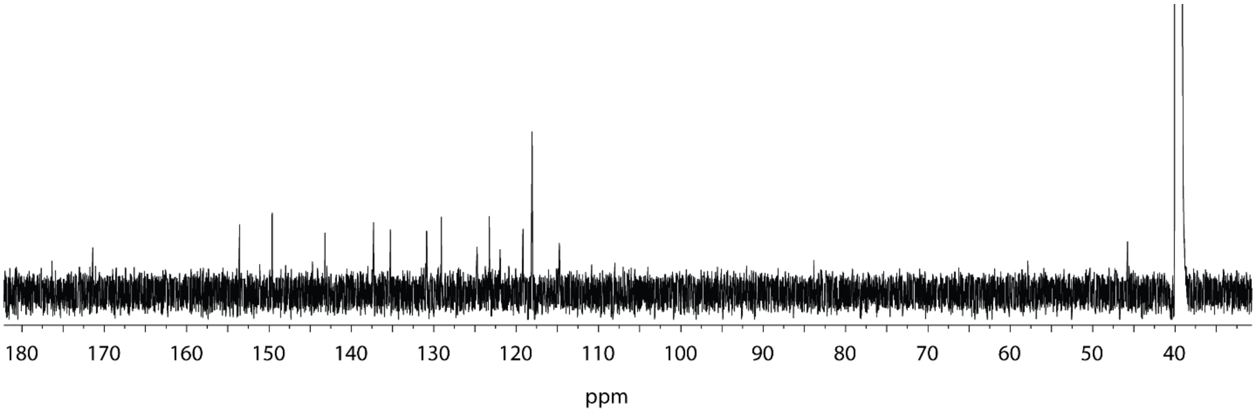


**Figure S85:** $^{13}C$ NMR spectrum (176 MHz, 298K, DMSO-$d_6$) of the helicate $[Pd_2($**MK-Q**$)_4](BF_4)_4$.

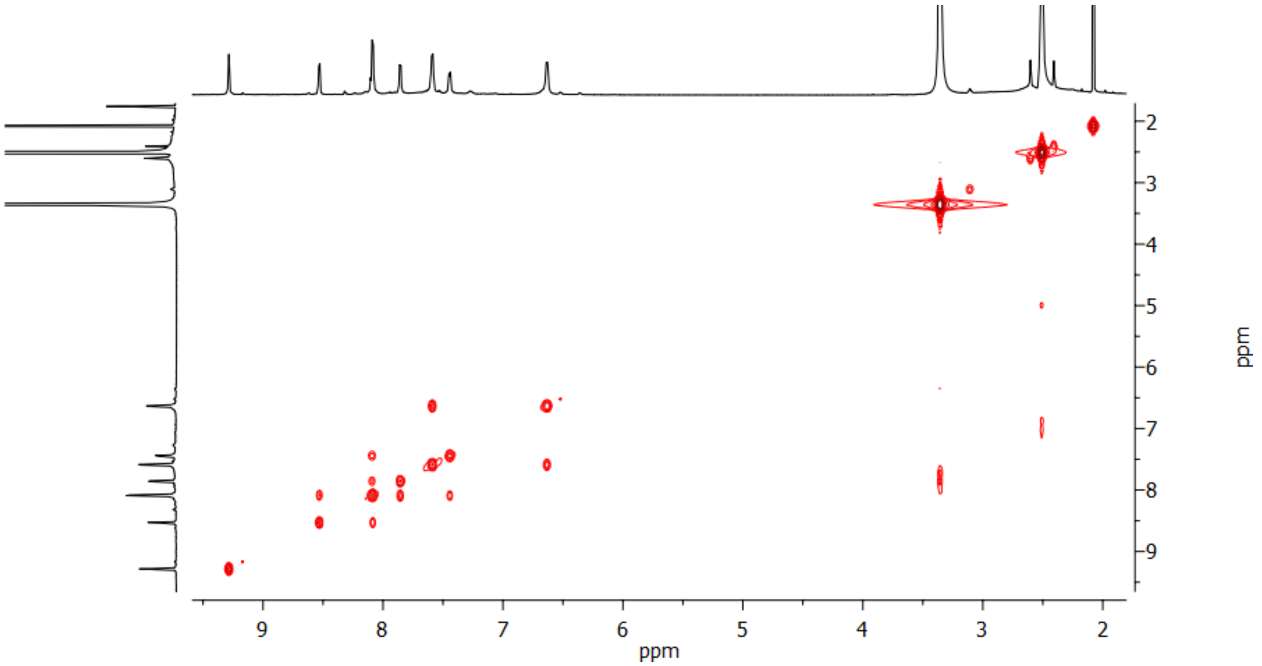

**Figure S86:** $^1$H – $^1$H COSY spectrum (600 MHz, 298K, DMSO-$d_6$) of helicate $[Pd_2($**MK-Q**$)_4](BF_4)_4$.

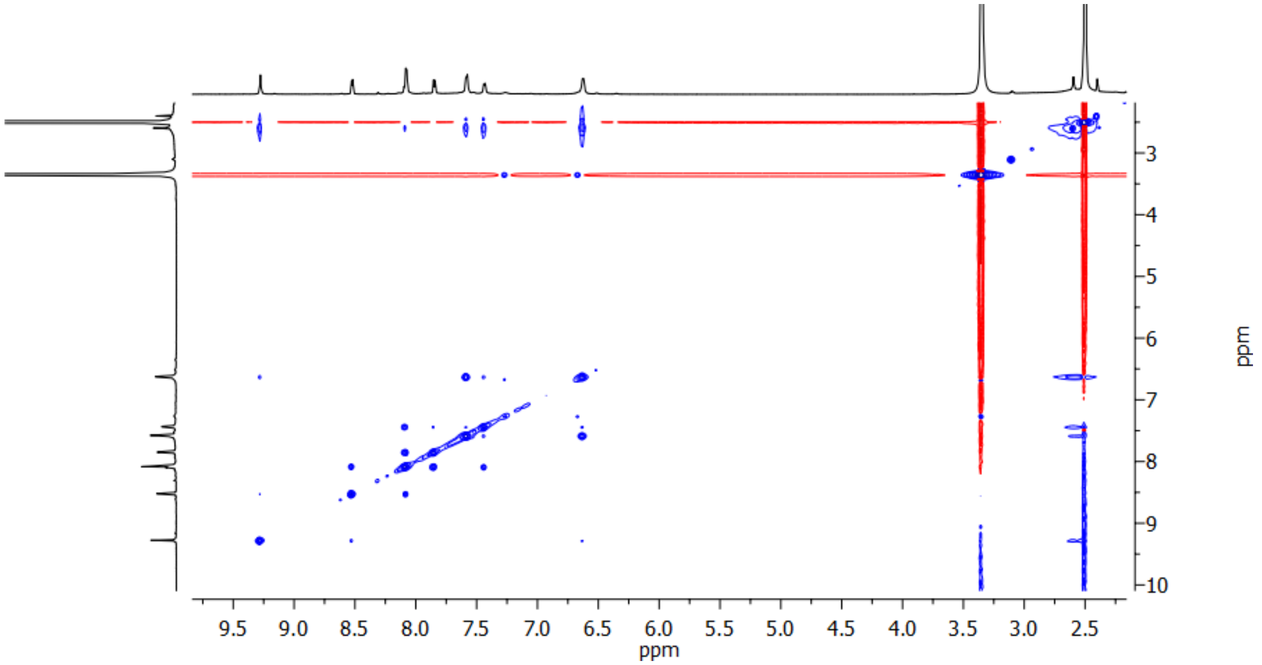

**Figure S87:** $^1$H – $^1$H NOESY spectrum (600 MHz, 298K, DMSO-$d_6$) of helicate $[Pd_2($**MK-Q**$)_4](BF_4)_4$.

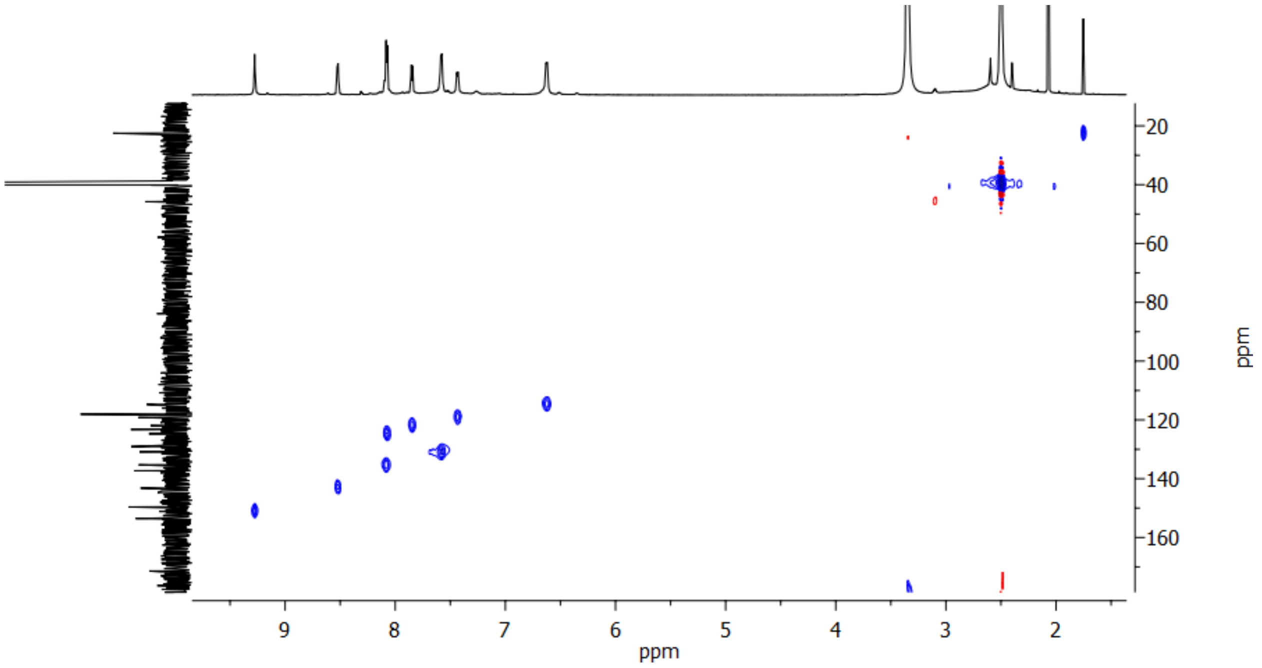


**Figure S88:** $^{1}H$ – $^{13}C$ HSQC spectrum (600 MHz, 298K, DMSO-$d_6$) of helicate $[Pd_2(\mathbf{MK\text{-}Q})_4](BF_4)_4$.

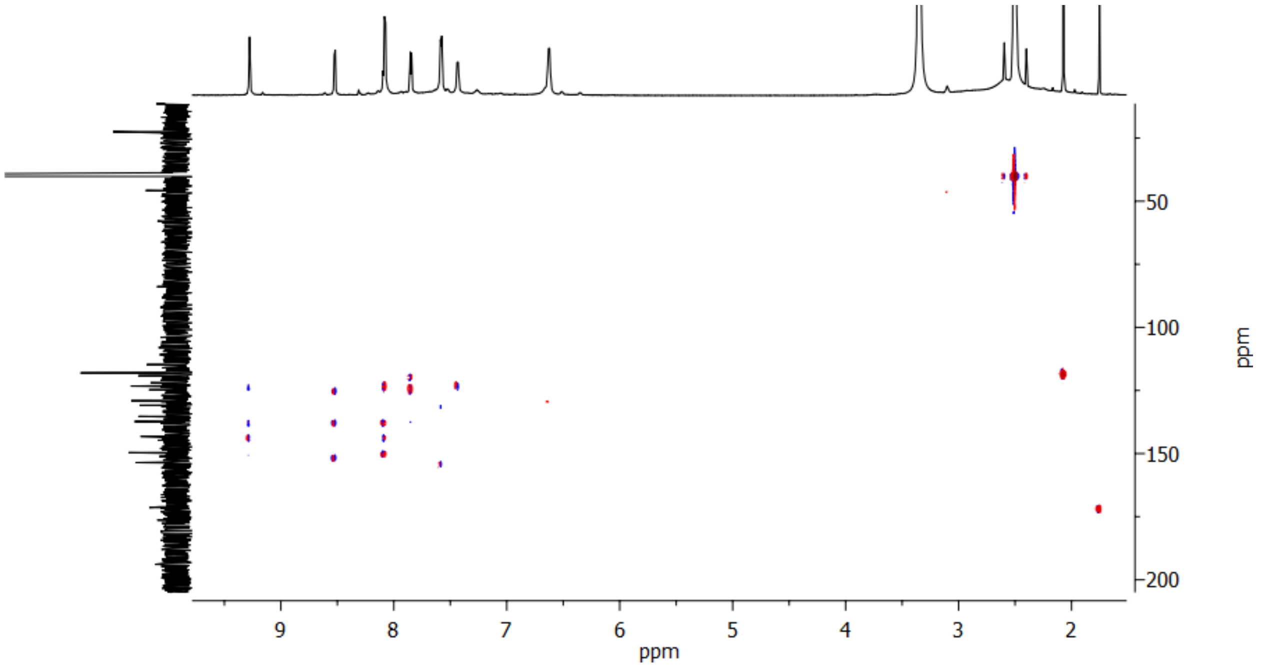


**Figure S89:** $^{1}H$ – $^{13}C$ HMBC spectrum (600 MHz, 298K, DMSO-$d_6$) of helicate $[Pd_2(\mathbf{MK\text{-}Q})_4](BF_4)_4$.

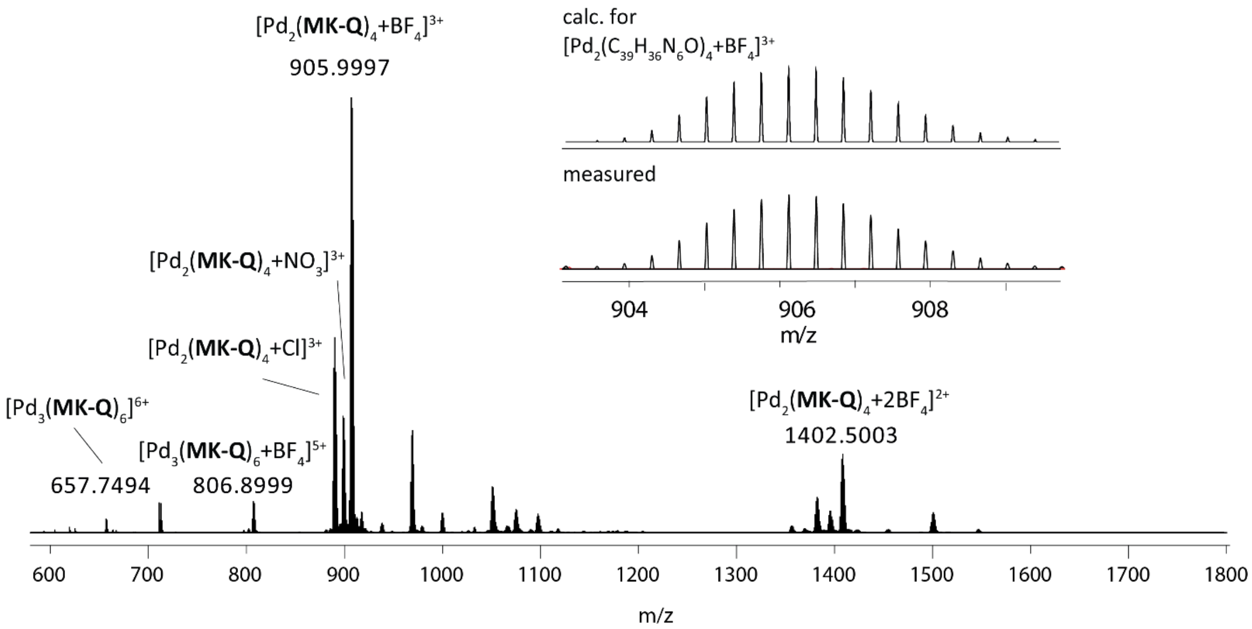


**Figure S90:** ESI-MS spectrum of $[Pd_2(\mathbf{MK\text{-}Q})^4+nBF_4]^{(4-n)+}$ with n=0-2. The observed and calculated isotopic pattern of $[Pd_2(\mathbf{MK\text{-}Q})_4+BF_4]^{3+}$ is shown in the inset.

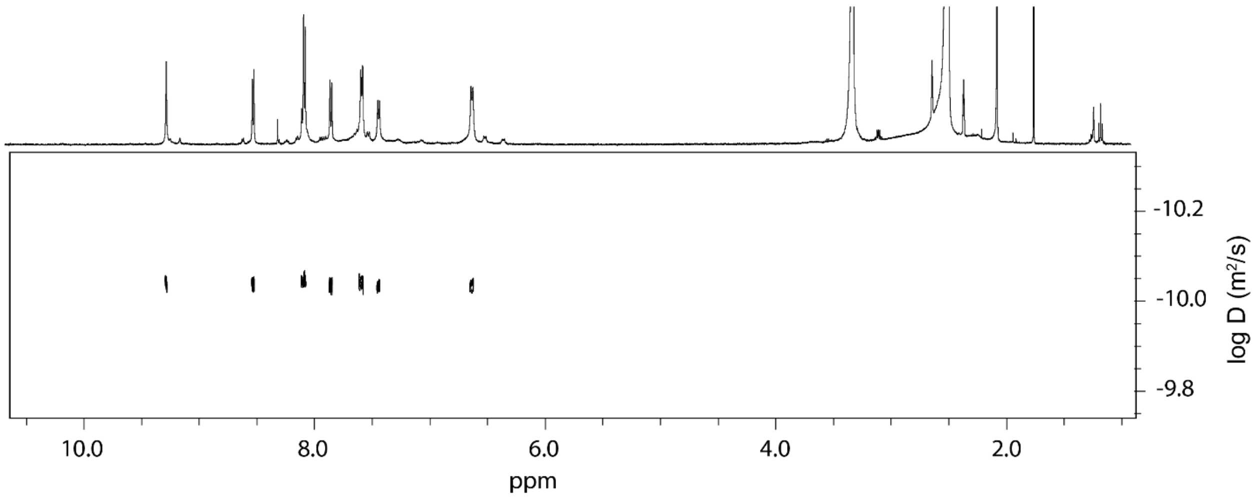


**Figure S91:** $^1$H DOSY spectrum (500 MHz, 298K, DMSO-$d_6$) of $[Pd_2(\mathbf{MK\text{-}Q})_4](BF_4)_8$ (0.7 mM). Diffusion coefficient: $9.399\times10^{-11}$ $m^2s^{-1}$, log D=-10.003. Hydrodynamic radius=11.68 Å.

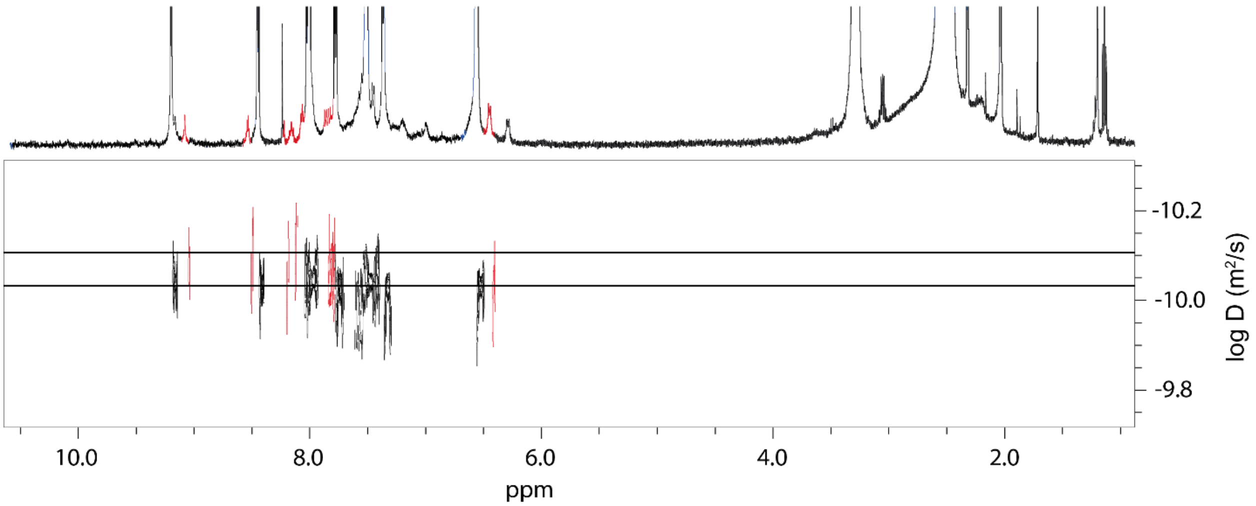


**Figure S92:** Enlargement of $^{1}$H DOSY spectrum (500 MHz, 298K, DMSO-$d_6$) of $[Pd_2($**MK-Q**$)_4](BF_4)_4$. We can observe a second species with higher diffusion coefficient: $8.039\times10^{-11}$ $m^2s^{-1}$, log D=-10.095. Hydrodynamic radius=13.65 Å which matches with the expected hydrodynamic radius for the three-membered ring $[Pd_3($**MK-Q**$)_6](BF_4)_6$.

[1]H DOSY analysis showed the coexistence of two species in the solution. Next to the $[Pd_2L_4]$ helicate, a larger assembly was assumed to be the $[Pd_3L_6]$ ring. In order to support this hypothesis, the expected increase in hydrodynamic radius from a $[Pd_2L_4]$ helicate to a $[Pd_3L_6]$ ring was calculated. In a 2D geometrical simplification where the Pd-Pd distances in the two assemblies are assumed to be the same, the two hydrodynamic radii would be associated via the formula:

$$R_{Pd_3L_6} \simeq \frac{2}{\sqrt{3}} R_{Pd_2L_4}$$

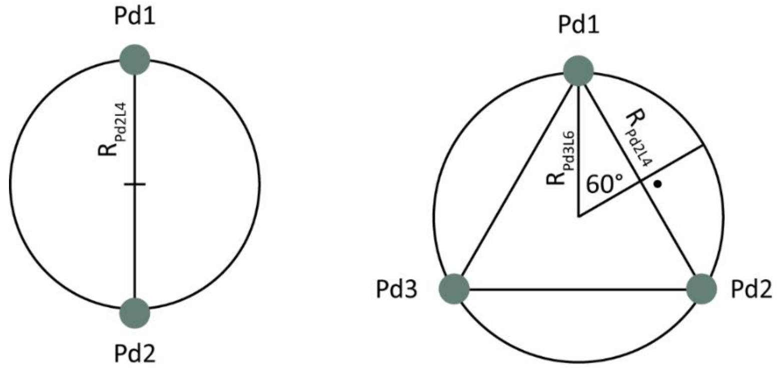


**Figure S93:** Geometrical estimation of the expected hydrodynamic radius for $[Pd_3($**MK-Q**$)_6](BF_4)_6$. In the 2D simplification, the Pd-Pd distances in the helicate $[Pd_2L_4]$ and in the ring $[P_3L_6]$ are assumed to be equal.

**Table S1**: Hydrodynamic radii calculated from the diffusion coefficient deriving from DOSY experiments and the calculated hydrodynamic radius calculated using the formula described here above.

| | DOSY | Calc. |
|---|---|---|
| $R_{Pd_2L_4}$ | 11.63 Å | 11.63 Å (input) |
| $R_{Pd_3L_6}$ | 13.65 Å | 13.43 Å (result) |

In Table S2, the hydrodynamic radii extrapolated from DOSY measurement of the two species, the smaller one attributed to [$Pd_2L_4$] with 11.63 Å and the larger one assigned to [$Pd_3L_6$] with 13.65 Å, and the estimated hydrodynamic radius for [$Pd_3L_6$] = 13.43 Å are shown. It can be concluded that ligand **MK-Q** mainly assembles to give the [$Pd_2L_4$] topology in solution, however, yielding also a small percentage of [$Pd_3L_6$] which turned out to be the preferred assembly crystallizing from the particular conditions employed (DMSO/toluene mixture).

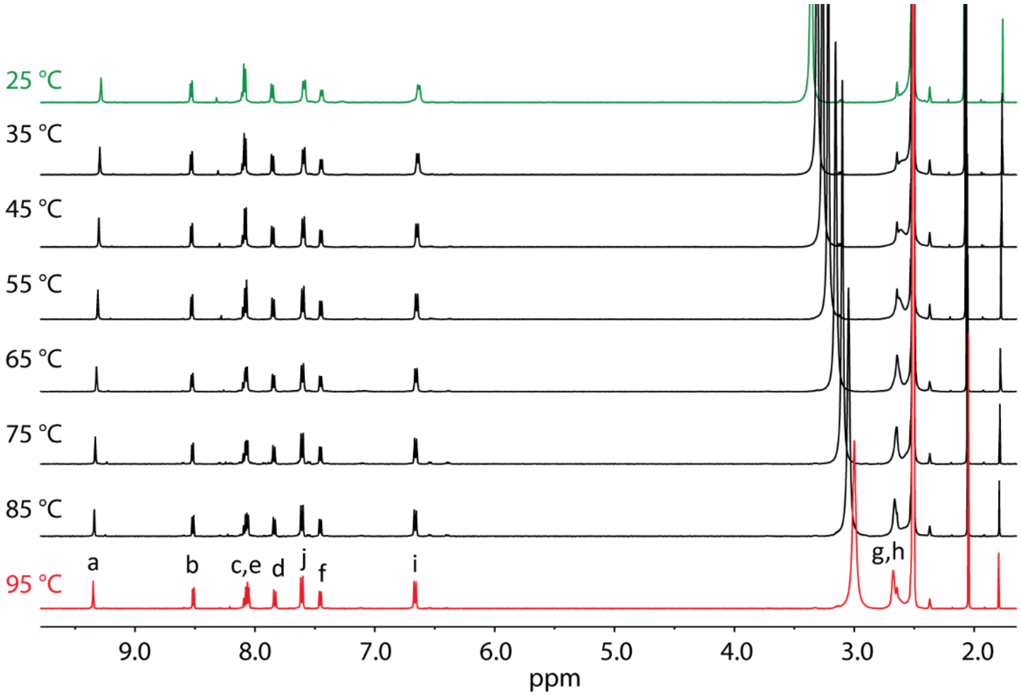


**Figure S94:** Variable temperature $^1$H-NMR (500 MHz, DMSO-$d_6$) of [$Pd_2$(**MK-Q**)$_4$]($BF_4$)$_4$ (0.7 mM). The temperatures are indicated in the Figure.

### 3.2.7 [$Pd_2$(RB-Q)$_4$]($BF_4$)$_4$

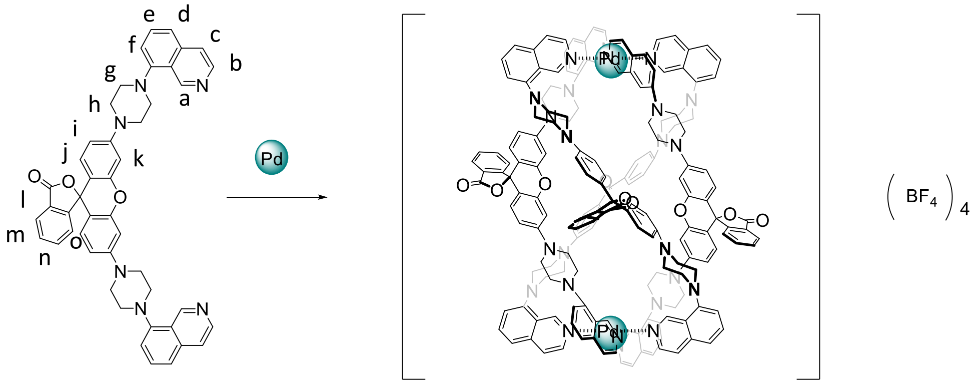


**RB-Q** [$Pd_2$(**RB-Q**)$_4$]($BF_4$)$_4$

**Scheme S18:** Self-Assembly of helicate [$Pd_2$(**RB-Q**)$_4$]($BF_4$)$_4$.

A mixture of ligand **RB-Q** (450 µL of a 3.11 mM solution in $CD_3CN$) and [Pd($CH_3CN$)$_4$]($BF_4$)$_2$ (50 µL of a 15 mM solution in $CD_3CN$) was heated at 70 °C for 15 min to afford a 0.7 mM solution of [$Pd_2$(**RB-Q**)$_4$]($BF_4$)$_4$.

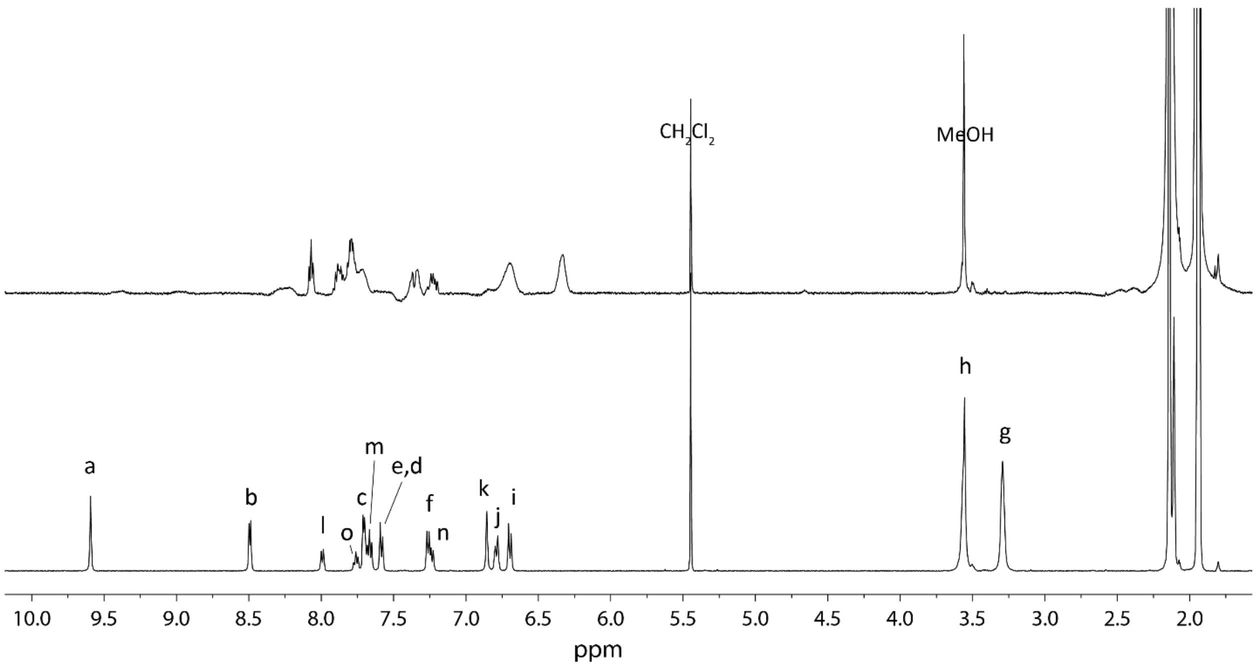


**Figure S95:** $^1H$ NMR stacked spectra (600 MHz, 298K, $CD_3CN$) of ligand **RB-Q** (bottom) and the correspondent helicate $[Pd_2($**RB-Q**$)_4](BF_4)_4$ (top) upon addition of 0.5 equiv. of Pd(II) salt.

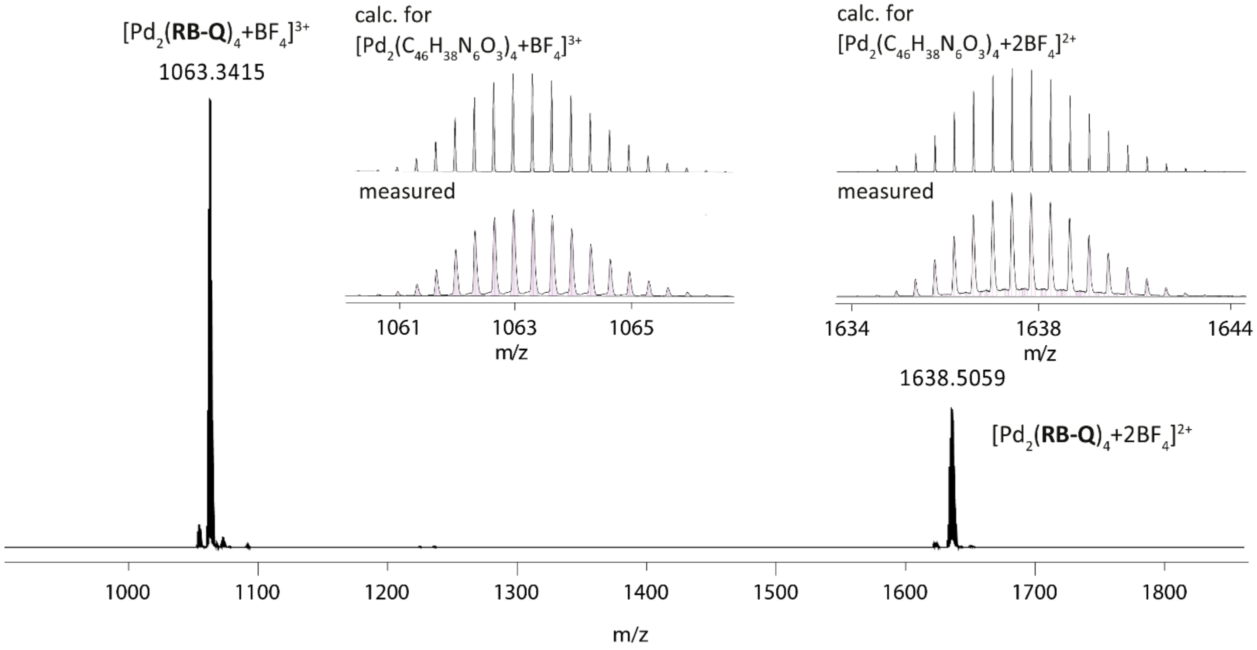


**Figure S96:** ESI-MS spectrum of $[Pd_2($**RB-Q**$)^4+nBF_4]^{(4-n)+}$ with n=0-2. The observed and calculated isotopic patterns of $[Pd_2($**RB-Q**$)_4+BF_4]^{3+}$ and $[Pd_2($**RB-Q**$)_4+2BF_4]^{2+}$ are shown in the inset.

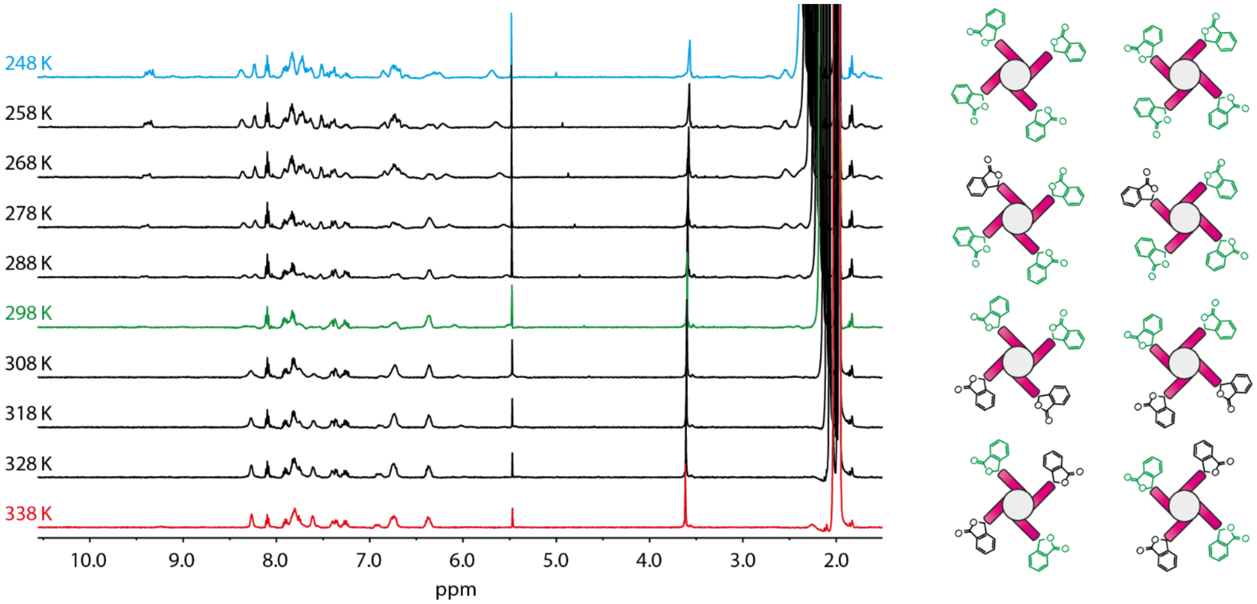


**Figure S97:** On the left: variable temperature $^{1}$H-NMR (500 MHz, $CD_3CN$) of [$Pd_2$(**RB-Q**)$_4$]($BF_4$)$_4$ (0.7 mM). The temperatures are indicated in the Figure. On the right: schematic top view on the four different isomers for [$Pd_2$(**RB-Q**)$_4$]($BF_4$)$_4$ that can originate from the flip of the spirolactone functional groups in ligand **RB-Q** in combination with the direction of the overall helical twist.

### 3.2.8 [$Pd_2$(MB-Q)$_4$]($NO_3$)$_8$

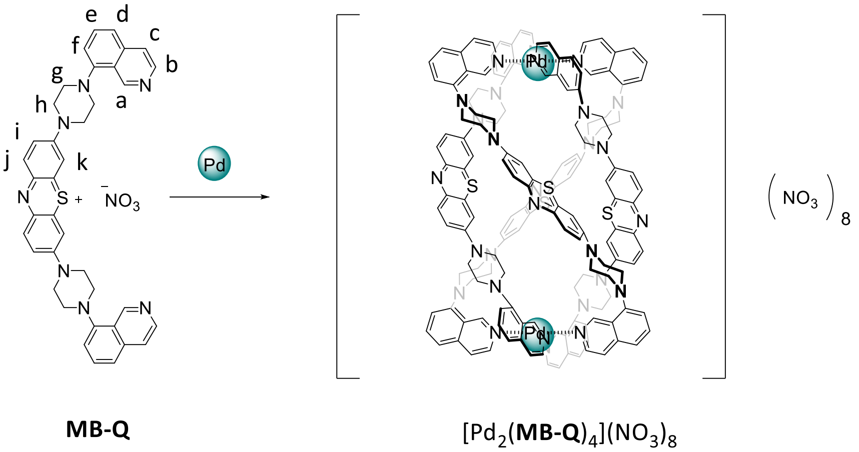


**Scheme S19:** Self-Assembly of helicate [$Pd_2$(**MB-Q**)$_4$]($NO_3$)$_8$.

A mixture of ligand **MB-Q** (450 µL of a 3.11 mM solution in DMSO-$d_6$) and Pd($NO_3$)$_2$ (50 µL of a 15 mM solution in DMSO-$d_6$) was heated at 70 °C for 15 min to afford a 0.7 mM solution of [$Pd_2$(**MB-Q**)$_4$]($NO_3$)$_8$.

$^{1}$H NMR (500 MHz, 298 K, dimethyl sulfoxide-$d_6$) δ 10.56 (s, 1H, Ha), 9.84 (d, $J$ = 6.8 Hz, 1H, Hb), 8.65 (d, $J$ = 8.0 Hz, 1H, He), 8.23 (d, $J$ = 6.6 Hz, 1H, Hc), 7.90 (t, $J$ = 7.8 Hz, 1H, Hi), 7.74 (d, $J$ = 8.3 Hz, 2H, Hd, Hk), 7.57 (d, $J$ = 6.6 Hz, 2H, Hf, Hj), 4.82 (s, 1H), 4.43 (s, 1H), 3.76 (s, 1H), 3.12(s, 1H), 2.73(s, 1H) (one signal overlapping with the solvent).

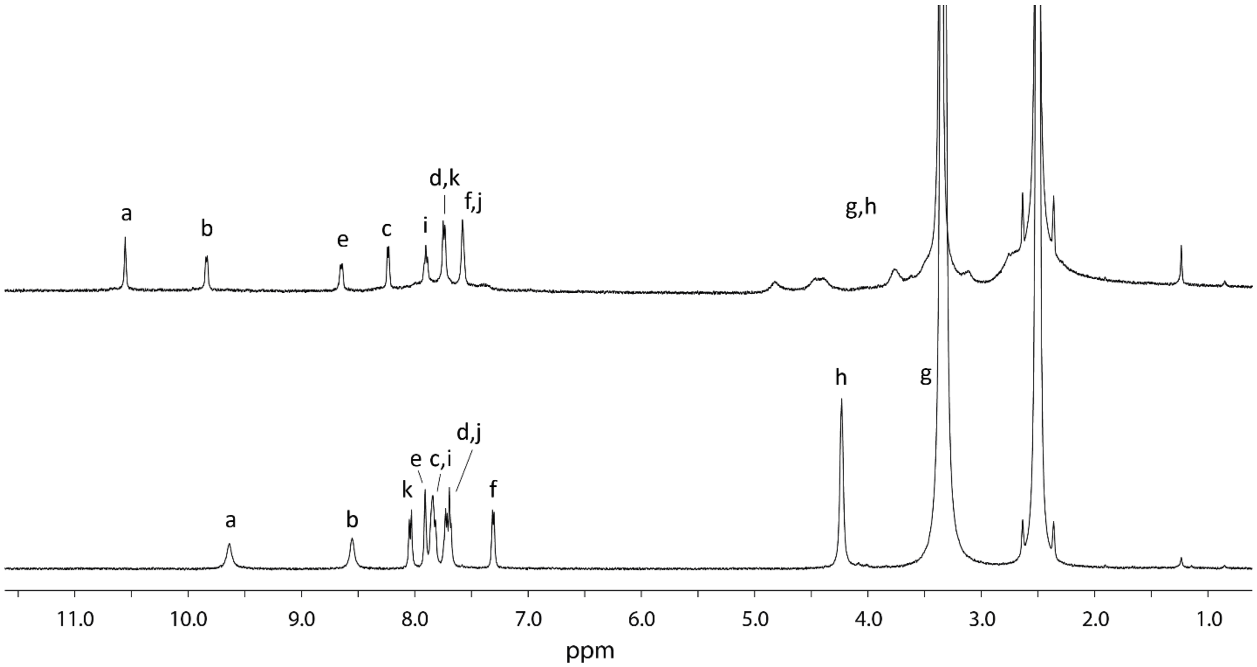


**Figure S98:** $^{1}$H NMR stacked spectra (600 MHz, 298K, DMSO-$d_6$) of ligand **MB-Q** (bottom) and the correspondent helicate $[Pd_2($**MB-Q**$)_4](NO_3)_8$ (top) upon addition of 0.5 equiv. of Pd(II) salt.

A mixture of ligand **MB-Q** suspended in $D_2O$ (450 µL of a 3.11 mM solution) and $Pd(NO_3)_2$ (50 µL of a 15 mM solution in $D_2O$) was heated at 70 °C for 15 min to afford a 0.7 mM solution of $[Pd_2($**MB-Q**$)_4](NO_3)_8$.

$^{1}$H NMR (600 MHz, 298 K, Deuterium Oxide) δ 9.70 (s, 1H, Ha), 8.43 (d, *J* = 6.9 Hz, 1H, Hb), 8.34 (d, *J* = 6.6 Hz, 1H, Hc), 8.02 (t, *J* = 8.0 Hz, 1H, He), 7.98 (d, *J* = 9.5 Hz, 1H, Hj), 7.86 (d, *J* = 8.3 Hz, 1H, Hd), 7.66 (d, *J* = 9.7 Hz, 1H, Hk), 7.56 (d, *J* = 8.8 Hz, 2H, Hf, Hi), 4.17 (t, *J* = 5.0 Hz, 4H, Hh), 3.46 (t, *J* = 5.0 Hz, 4H, Hg).

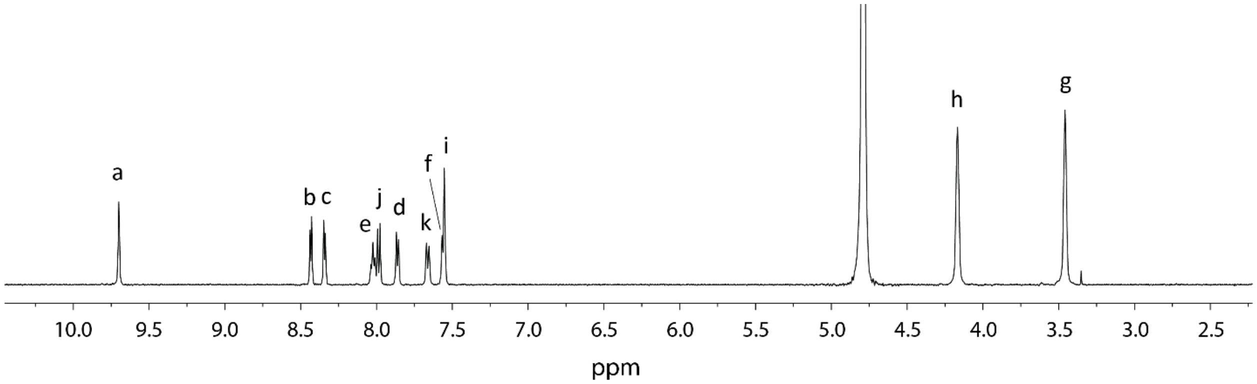


**Figure S99:** $^{1}$H NMR spectrum (600 MHz, 298K, $D_2O$) of the helicate $[Pd_2($**MB-Q**$)_4](NO_3)_8$.

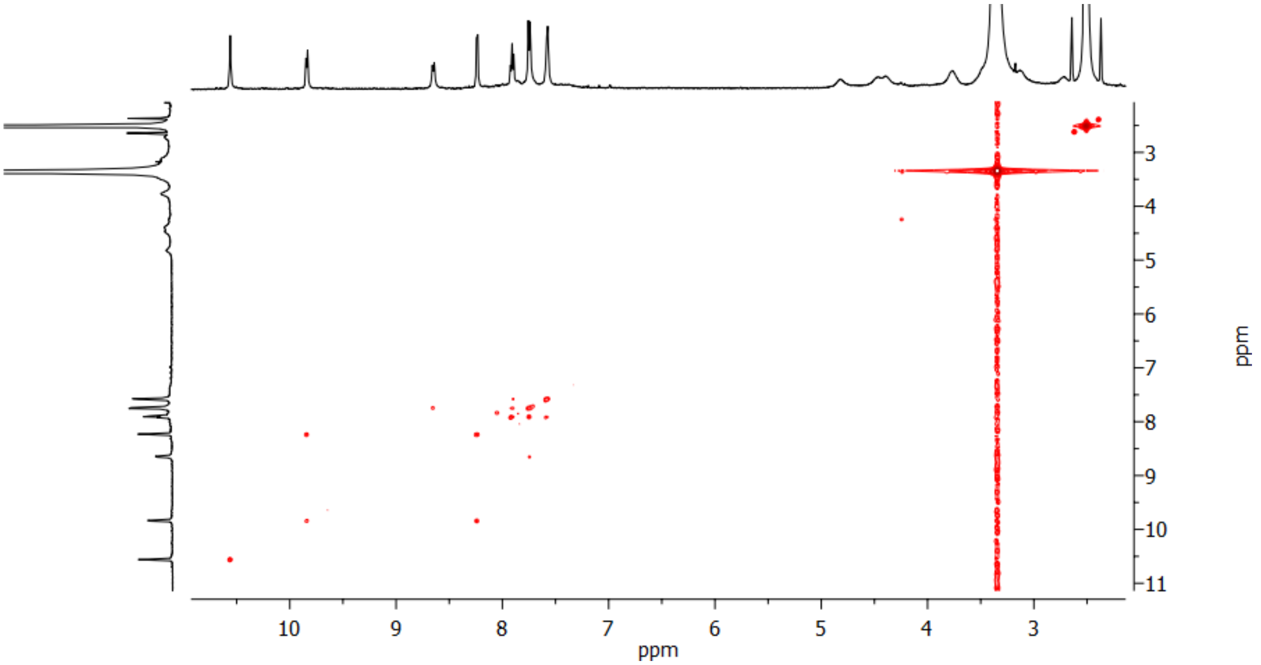


**Figure S100:** $^{1}H$ – $^{1}H$ COSY spectrum (600 MHz, 298K, DMSO-$d_6$) of helicate $[Pd_2($**MB-Q**$)_4](NO_3)_8$.

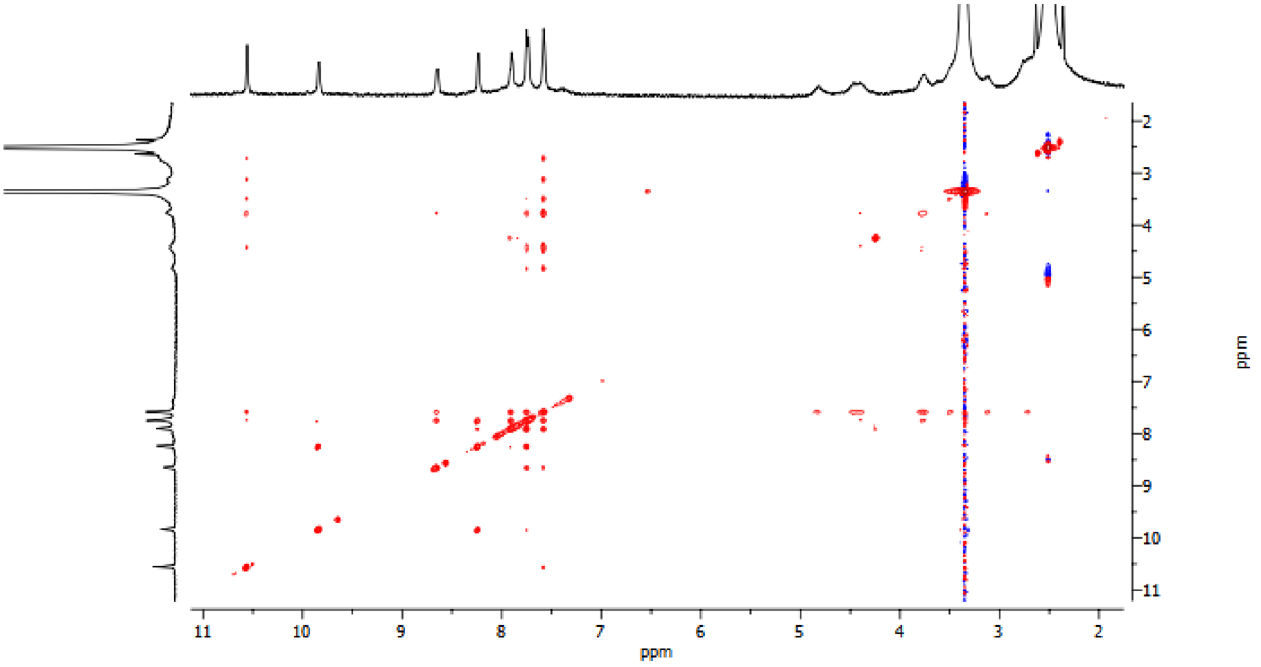


**Figure S101:** $^{1}H$ – $^{1}H$ NOESY spectrum (600 MHz, 298K, DMSO-$d_6$) of helicate $[Pd_2($**MB-Q**$)_4](NO_3)_8$.

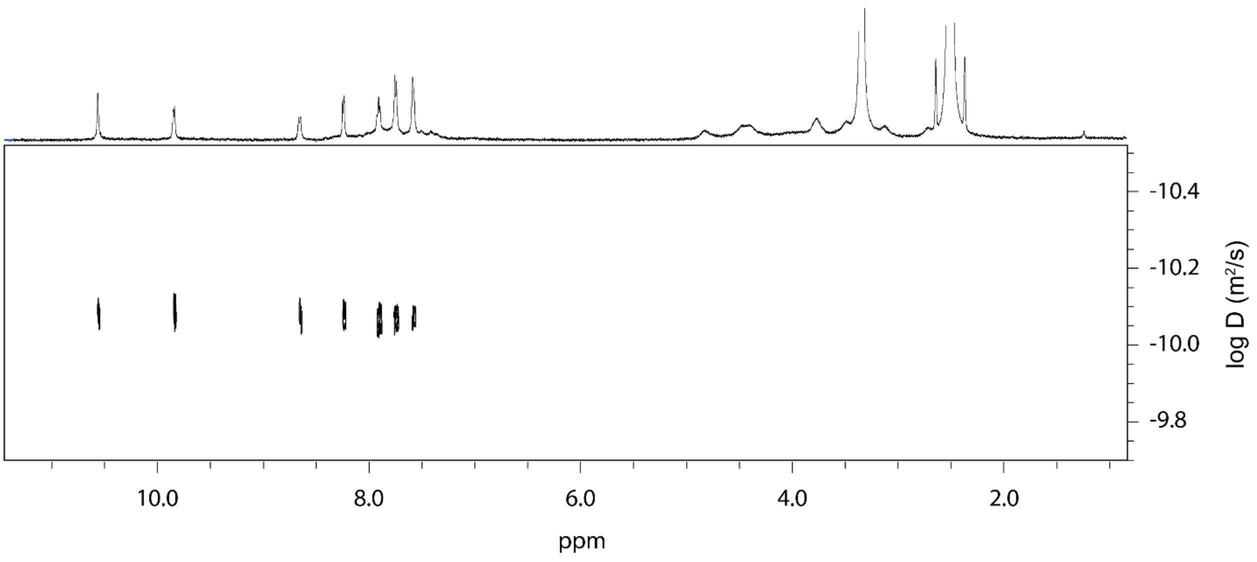


**Figure S102:** $^{1}$H DOSY spectrum (500 MHz, 298K, DMSO-$d_6$) of $[Pd_2(\mathbf{MB\text{-}Q})_4](NO_3)_8$ (0.7 mM). Diffusion coefficient: $10.928\times10^{-11}$ $m^2s^{-1}$, log D=-9.967. Hydrodynamic radius= 10.05 Å.

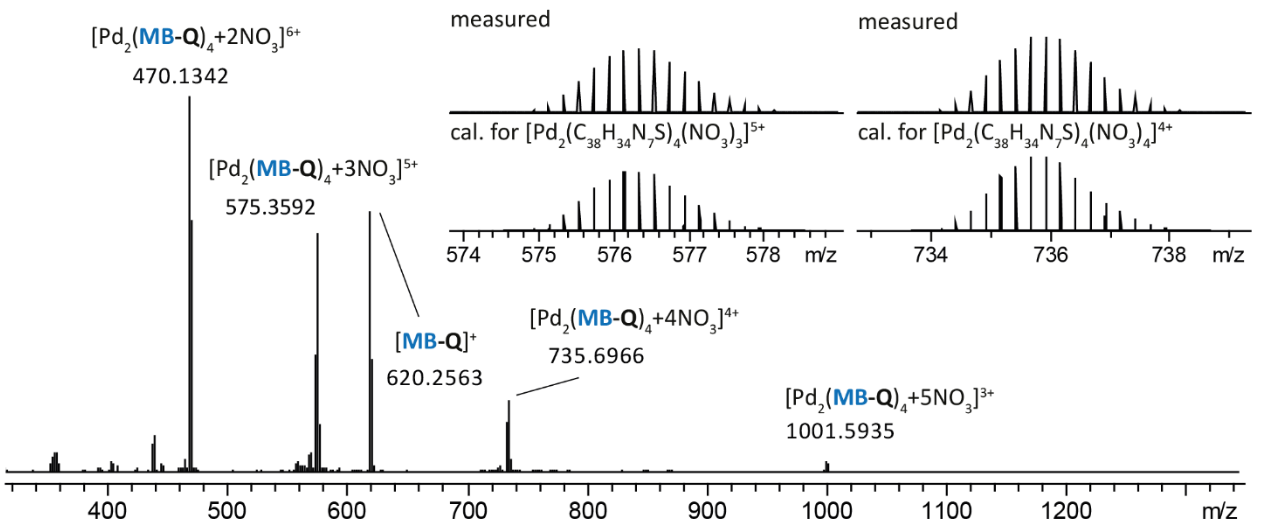


**Figure S103:** ESI-MS spectrum of $[Pd_2(\mathbf{MB\text{-}Q})_4+nNO_3]^{(8-n)+}$ with n=2-5. The observed and calculated isotopic patterns of $[Pd_2(\mathbf{MB\text{-}Q})_4+3NO_3]^{5+}$ and $[Pd_2(\mathbf{MB\text{-}Q})_4+4NO_3]^{4+}$ are shown in the inset.

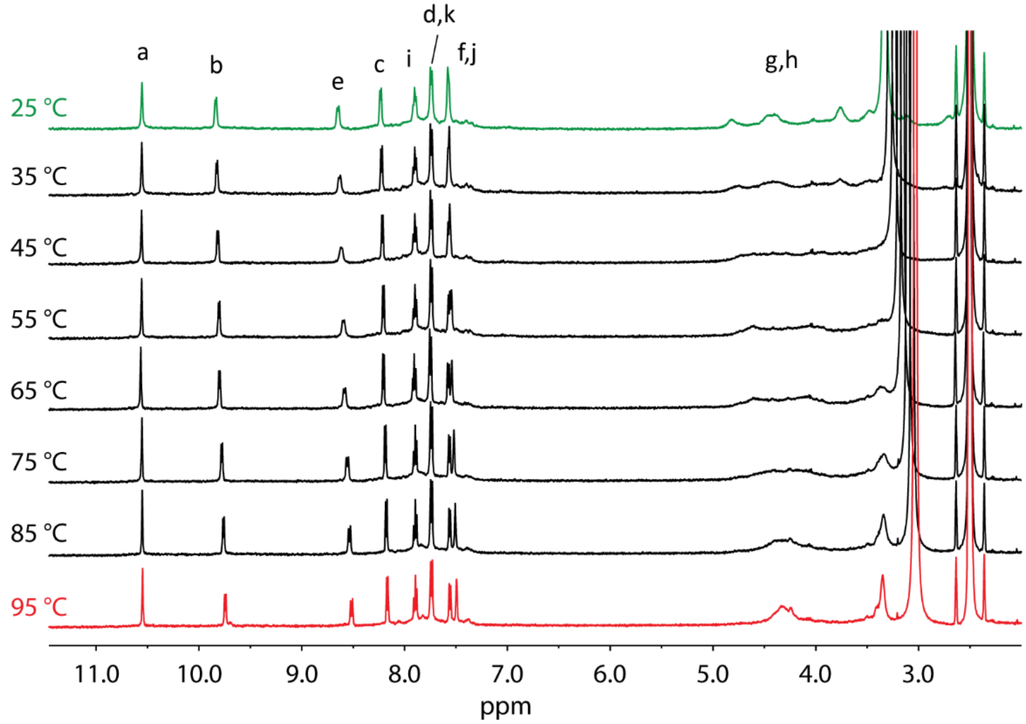


**Figure S104:** Variable temperature $^{1}$H-NMR (500 MHz, DMSO-$d_6$) of $[Pd_2(\textbf{MB-Q})_4](NO_3)_8$ (0.7 mM). The temperatures are indicated in the Figure.

### 3.2.9 [Pd₂(CV-Q)₄](NO₃)₈

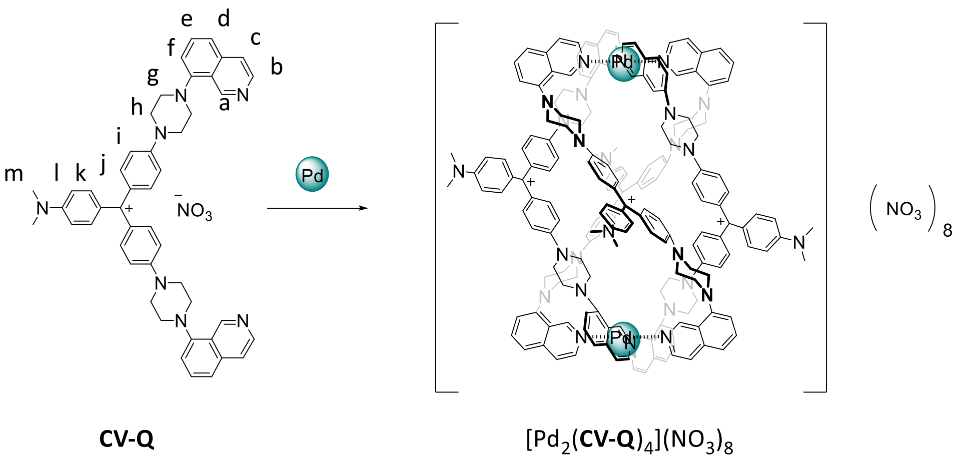


**Scheme S20:** Self-Assembly of helicate $[Pd_2(\textbf{CV-Q})_4](NO_3)_8$.

A mixture of ligand **CV-Q** (450 µL of a 3.11 mM solution in $CD_3CN$) and $Pd(NO_3)_2$ (50 µL of a 15 mM solution in $CD_3CN$) was heated at 70 °C for 15 min to afford a 0.7 mM solution of $[Pd_2(\textbf{CV-Q})_4](NO_3)_8$.

$^{1}$H NMR (500 MHz, 298 K, acetonitrile-$d_3$) δ 9.92 (s, 1H, Ha), 8.49 (d, *J* = 6.6 Hz, 1H, Hb), 7.95 – 7.82 (m, 2H, He, Hc), 7.70 (d, *J* = 8.2 Hz, 1H, Hd), 7.42 (d, *J* = 9.2 Hz, 1H, Hk), 7.31 (d, *J* = 8.8 Hz, 2H, Hj), 7.21 (d, *J* = 7.6 Hz, 1H, Hf), 6.99 (d, *J* = 9.4 Hz, 1H, Hl), 6.72 (d, *J* = 8.8 Hz, 2H, Hi), 3.13 (s, 3H, Hm), 2.80 (s, 2H), 2.61 (s, 2H).

$^{13}C$ NMR (151 MHz, 298 K, acetonitrile-*d*$_3$) δ 178.39 (C+), 157.70 (C$^q$, **C**-Ck), 156.36 (C$^q$, **C**-Cj), 153.90 (Ca), 151.21 (C$^q$, **C**-Cc), 143.57 (Cb), 141.54 (Ck), 139.62 (Cj), 138.45(C$^q$, **C**-Ca), 135.44 (Ce), 128.83 (C$^q$, **C**-Ci), 127.29 (C$^q$, **C**-Cl), 125.00 (Cc), 124.83 (C$^q$, **C**-Cf), 122.26 (Cd), 118.98 (Cf) 114.18 (Ci), 113.98 (Cj), 46.58 (Cg, Ch, broad…), 40.88 (Cm).

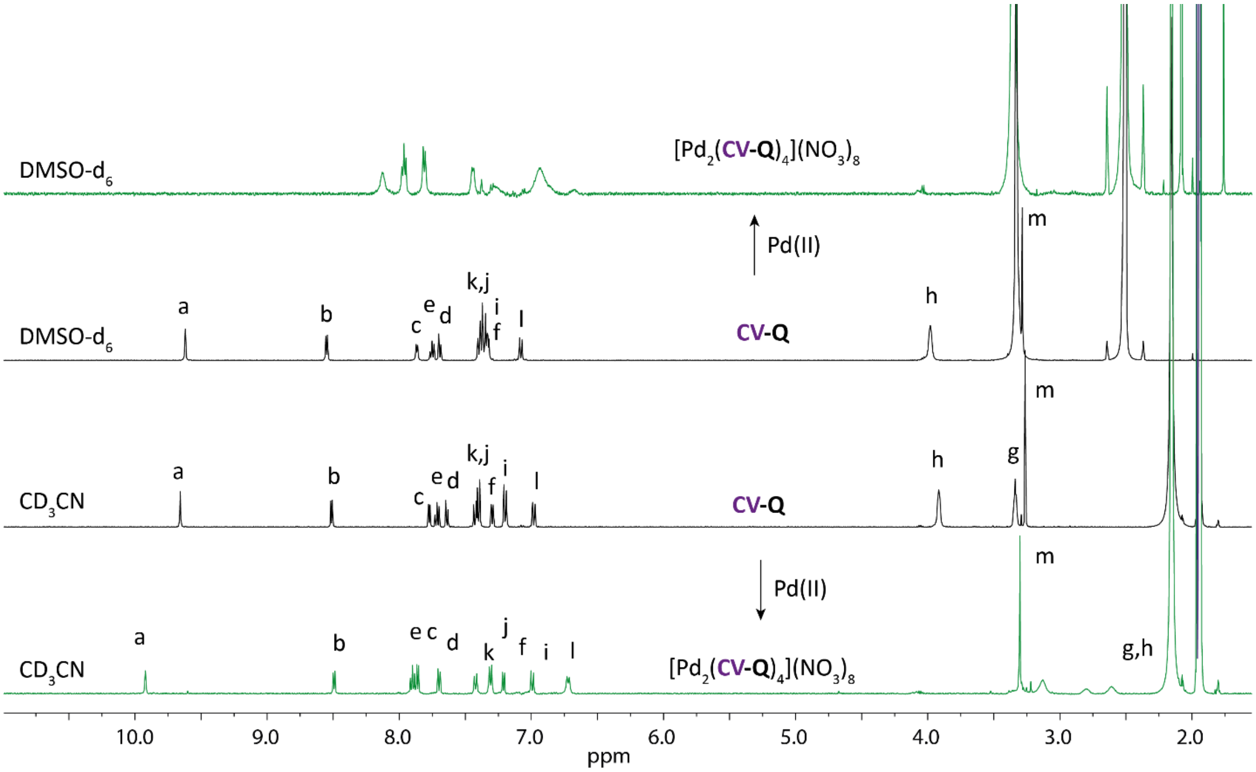


**Figure S105:** Stacked $^{1}H$ NMR spectra (500 MHz, 298K) of ligand **CV-Q** (in the middle in black) in $CD_3CN$ and DMSO-*d*$_6$ and the correspondent helicate $[Pd_2($**CV-Q**$)_4](NO_3)_8$ (in green) upon addition of 0.5 equiv. of Pd(II) salt in DMSO-*d*$_6$ on the top and $CD_3CN$ on the bottom, as indicated in the figure.

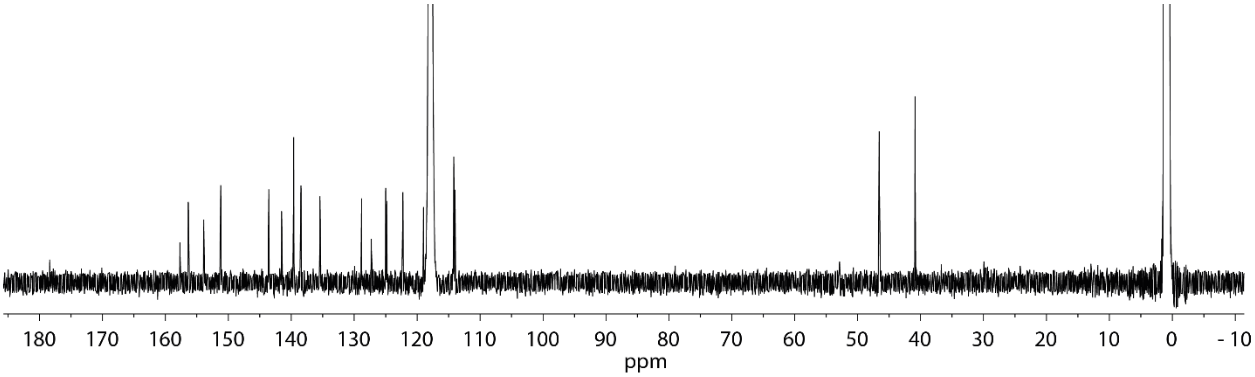


**Figure S106:** $^{13}C$ NMR spectrum (151 MHz, 298K, $CD_3CN$) of the helicate $[Pd_2($**CV-Q**$)_4](NO_3)_8$.

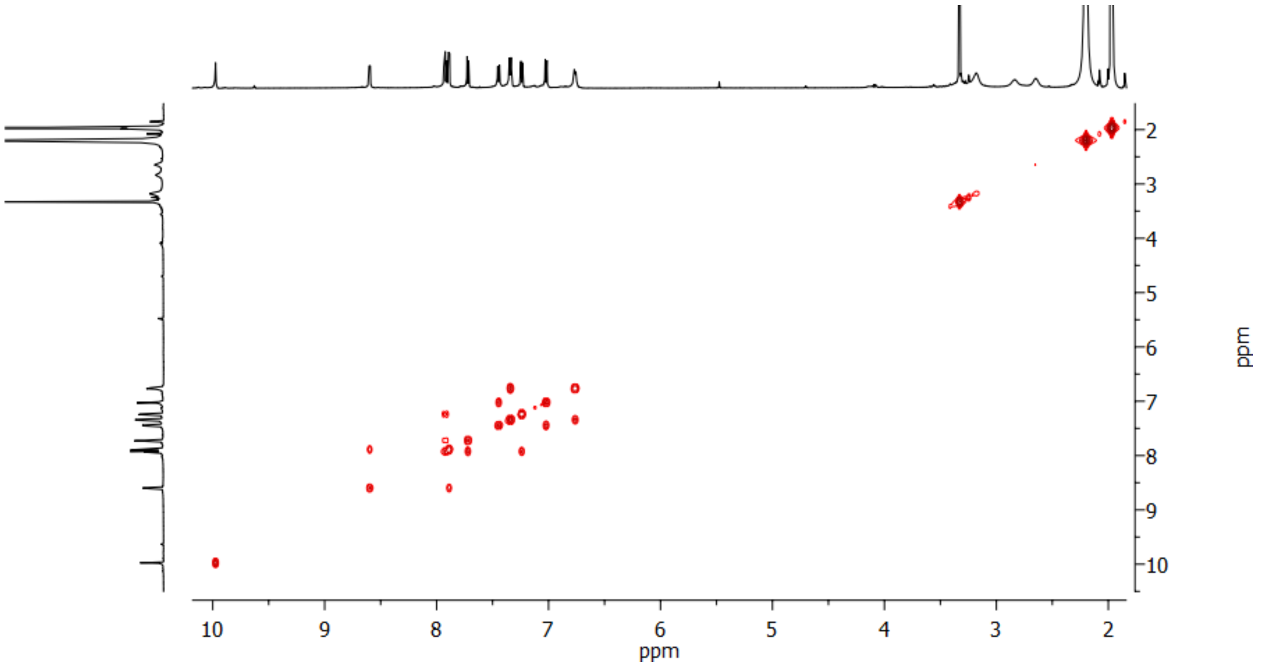


**Figure S107:** $^{1}H$ – $^{1}H$ COSY spectrum (600 MHz, 298K, $CD_3CN$) of helicate $[Pd_2($**CV-Q**$)_4](NO_3)_8$.

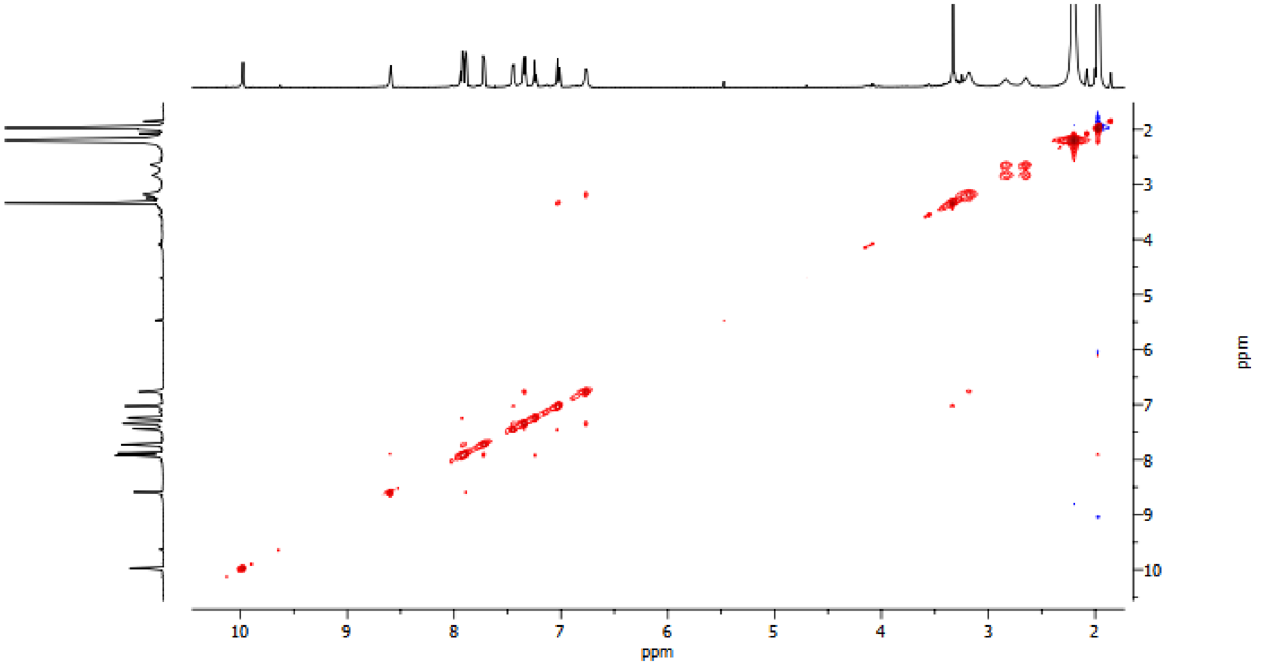


**Figure S108:** $^{1}H$ – $^{1}H$ NOESY spectrum (600 MHz, 298K, $CD_3CN$) of helicate $[Pd_2($**CV-Q**$)_4](NO_3)_8$.

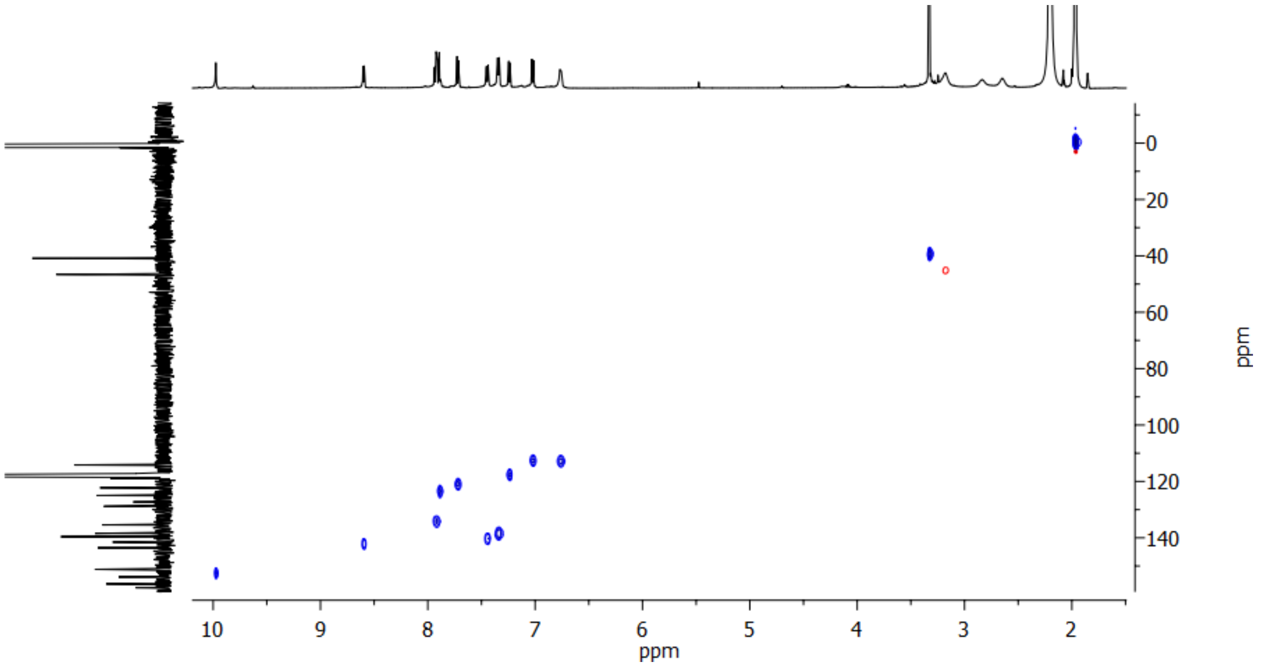


**Figure S109:** $^{1}H$ – $^{13}C$ HSQC spectrum (600 MHz, 298K, $CD_3CN$) of helicate $[Pd_2($**CV-Q**$)_4](NO_3)_8$.

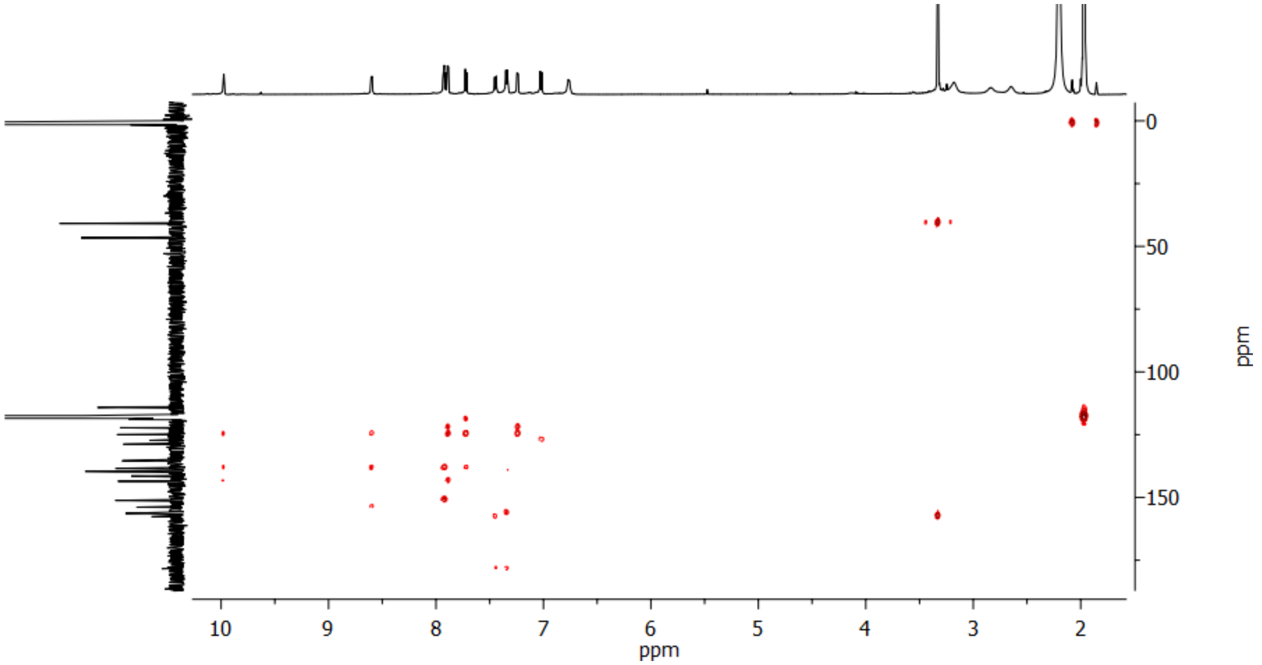


**Figure S110:** $^{1}H$ – $^{13}C$ HMBC spectrum (600 MHz, 298K, $CD_3CN$) of helicate $[Pd_2($**CV-Q**$)_4](NO_3)_8$.

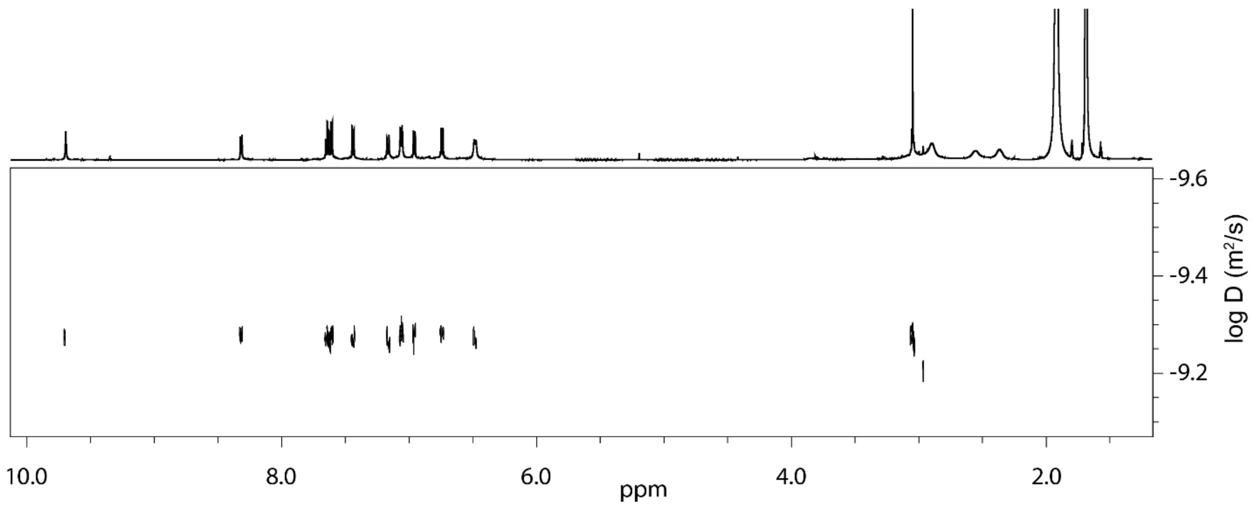


**Figure S111:** $^1$H DOSY spectrum (500 MHz, 298K, $CD_3CN$) of $[Pd_2(\mathbf{CV\text{-}Q})_4](NO_3)_8$ (0.7 mM). Diffusion coefficient: $5.293\times10^{-10}$ $m^2s^{-1}$, log D=-9.276. Hydrodynamic radius= 12.36 Å.

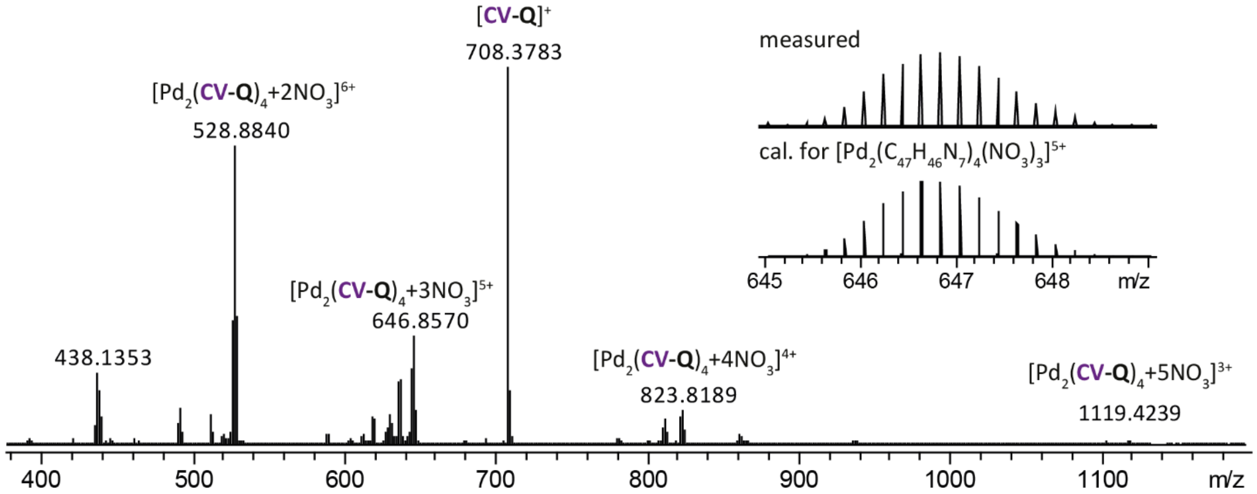


**Figure S112:** ESI-MS spectrum of $[Pd_2(\mathbf{CV\text{-}Q})_4\text{+}nNO_3]^{(8-n)+}$ with n=3-6. The observed and calculated isotopic pattern of $[Pd_2(\mathbf{CV\text{-}Q})_4\text{+}3NO_3]^{5+}$ is shown in the inset.

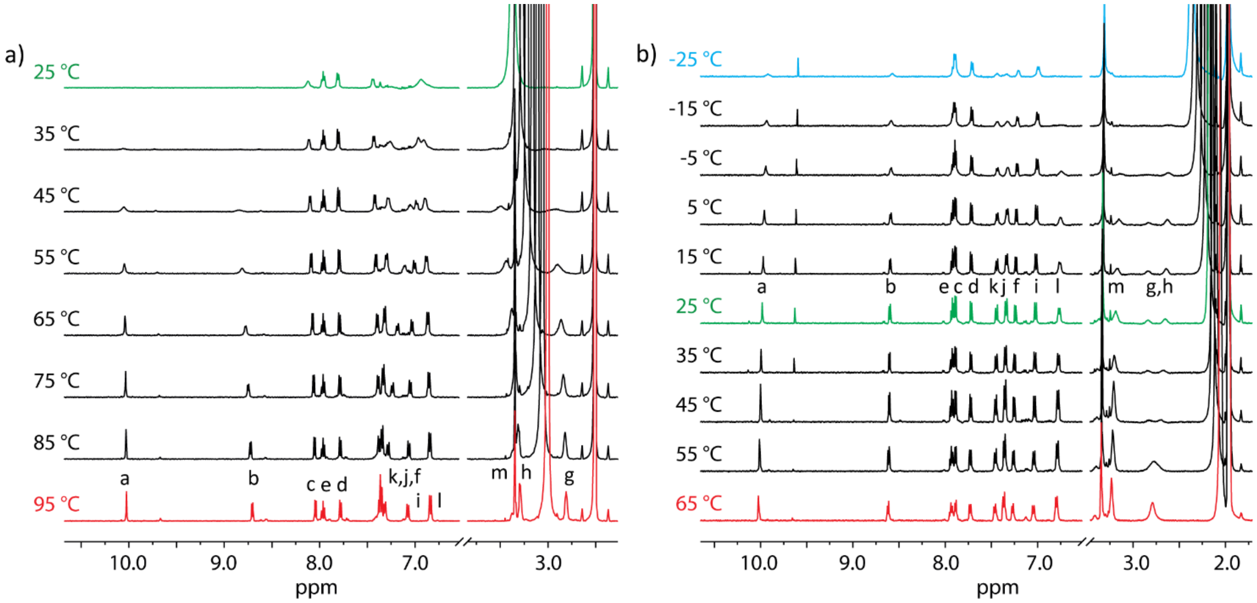


**Figure S113:** Stacked $^1H$ NMR (500 MHz) spectra of helicate $[Pd_2($**CV-Q**$)_4]$ at the different temperature indicated in the Figure. In green r.t., in blue the lowest temperature and in red the highest temperature. a) In DMSO-*d*$_6$ , the signals become sharper with increasing temperature until full assignable spectra are measured from 75°C and higher. b) In $CD_3CN$, increasing the temperature from r.t. causes coalescence of the piperazine signals around 3 ppm, while decreasing the temperature has the effect of broadening the signals with a situation at -25°C being similar to the r.t. spectrum in DMSO-*d*$_6$ (blue line in b) and green line in a). The signal at 9.6 ppm in b) that is not assigned to the helicate, is attributed to decomposition caused by the measurements at high temperature.

# 4 $^1$H-DOSY spectroscopy

$^1$H DOSY NMR spectra were recorded with a dstebpgp3s pulse sequence[7,8] with diffusion delays D20 of 0.06-0.10 s and gradient powers P30 of 800 to 2000 µs for each species optimized. Diffusion coefficients were extrapolated with the T1 relaxation module analysis from the fitting with the Stejskal-Tanner-Equation[9] of the decay of the signals´ integration. In Figure S114, a representative example is reported. In Table S2 the diffusion coefficients and hydrodynamic radius calculated with the Stokes-Einstein equation[10,11] for all ligands and corresponding assemblies are reported.

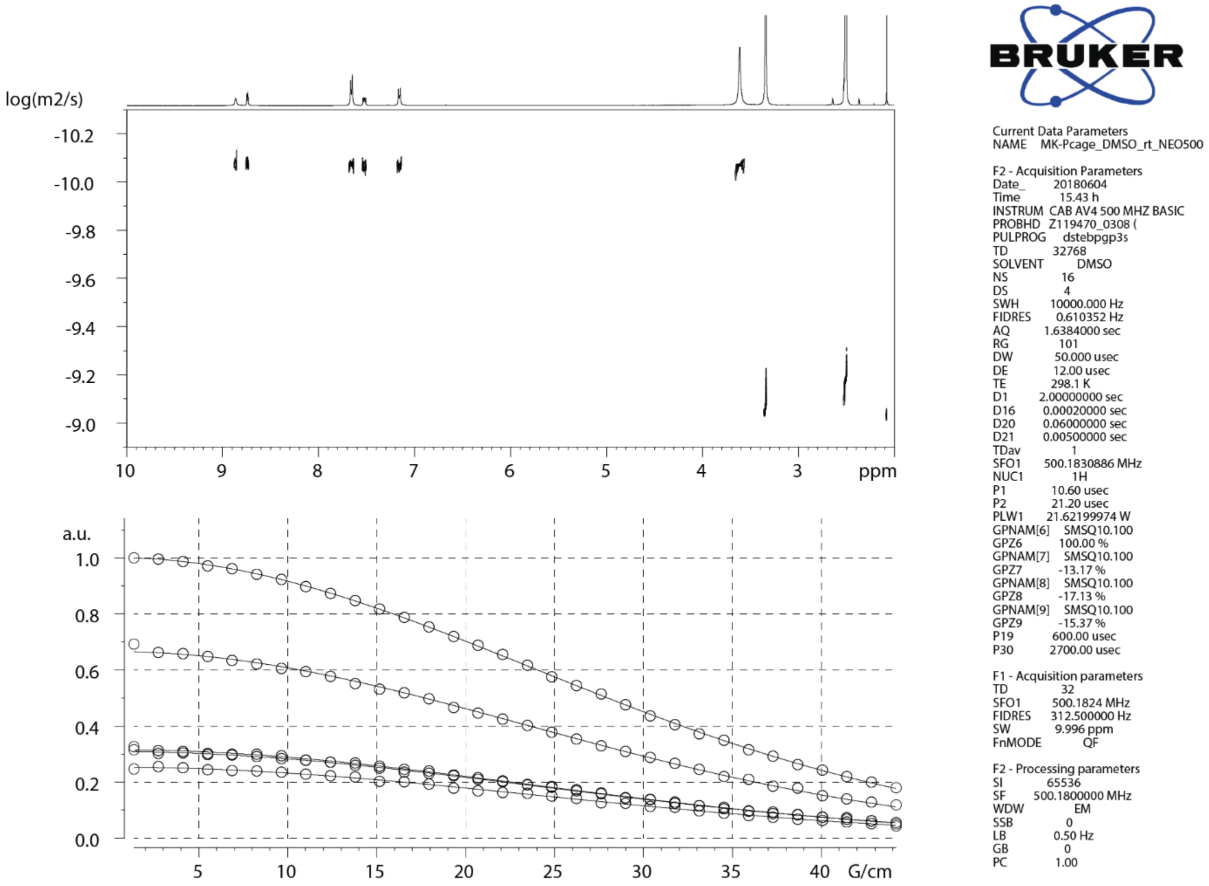


**Figure S114:** $^1$H DOSY spectrum (500 MHz, 298K, DMSO-$d_6$) of $[Pd_2(\textbf{MK-P})_4](BF_4)$ (0.7 mM), T1 analysis and acquisition parameters.

**Table S2**: Experimental diffusion coefficient values D determined from $^1$H-DOSY experiments. Hydrodynamic radius rH calculated from Stokes-Einstein equation**.**

| Ligand | D ($m^2s^{-1}$) | Log D | rH (Å) | Assembly | D ($m^2s^{-1}$) | Log D | rH (Å) |
|---|---|---|---|---|---|---|---|
| **MK-P** [a] | $1.902\times10^{-10}$ | -9.721 | 5.77 | $[Pd_2(\textbf{MK-P})_4]$[a] | $8.559\times10^{-11}$ | -10.068 | 12.83 |
| **RB-P** [a] | $1.667\times10^{-10}$ | -9.778 | 6.59 | $[Pd_2(\textbf{RB-P})_4]$[a] | $8.642\times10^{-11}$ | -10.063 | 12.71 |
| **MB-P** [a] | $1.625\times10^{-10}$ | -9.789 | 6.75 | $[Pd_2(\textbf{MB-P})_4]$[a] | $8.253\times10^{-11}$ | -10.083 | 13.30 |
| **CV-P** [a] | $1.599\times10^{-10}$ | -9.797 | 6.87 | $[Pd_2(\textbf{CV-P})_4]$[a] | $7.990\times10^{-11}$ | -10.098 | 13.74 |
| **RE-P** [a] | $1.558\times10^{-10}$ | -9.808 | 7.05 | $[Pd_2(\textbf{RE-P})_4]$ [a] | $8.232\times10^{-11}$ | -10.085 | 13.54 |
| **MK-Q** [a] | $1.705\times10^{-10}$ | -9.768 | 6.44 | $[Pd_2(\textbf{MK-Q})_4]$[a] | $9.399\times10^{-11}$ | -10.003 | 11.68 |
| **RB-Q** [b] | $9.206\times10^{-10}$ | -9.036 | 7.11 | $[Pd_2(\textbf{RB-Q})_4]$[a] | - | - | - |
| **MB-Q** [a] | $1.500\times10^{-10}$ | -9.824 | 7.32 | $[Pd_2(\textbf{MB-Q})_4]$[a] | $10.928\times10^{-11}$ | -9.967 | 10.05 |
| **CV-Q** [b] | $8.771\times10^{-10}$ | -9.057 | 7.44 | $[Pd_2(\textbf{CV-Q})_4]$[b] | $5.293\times10^{-10}$ | -9.276 | 12.36 |

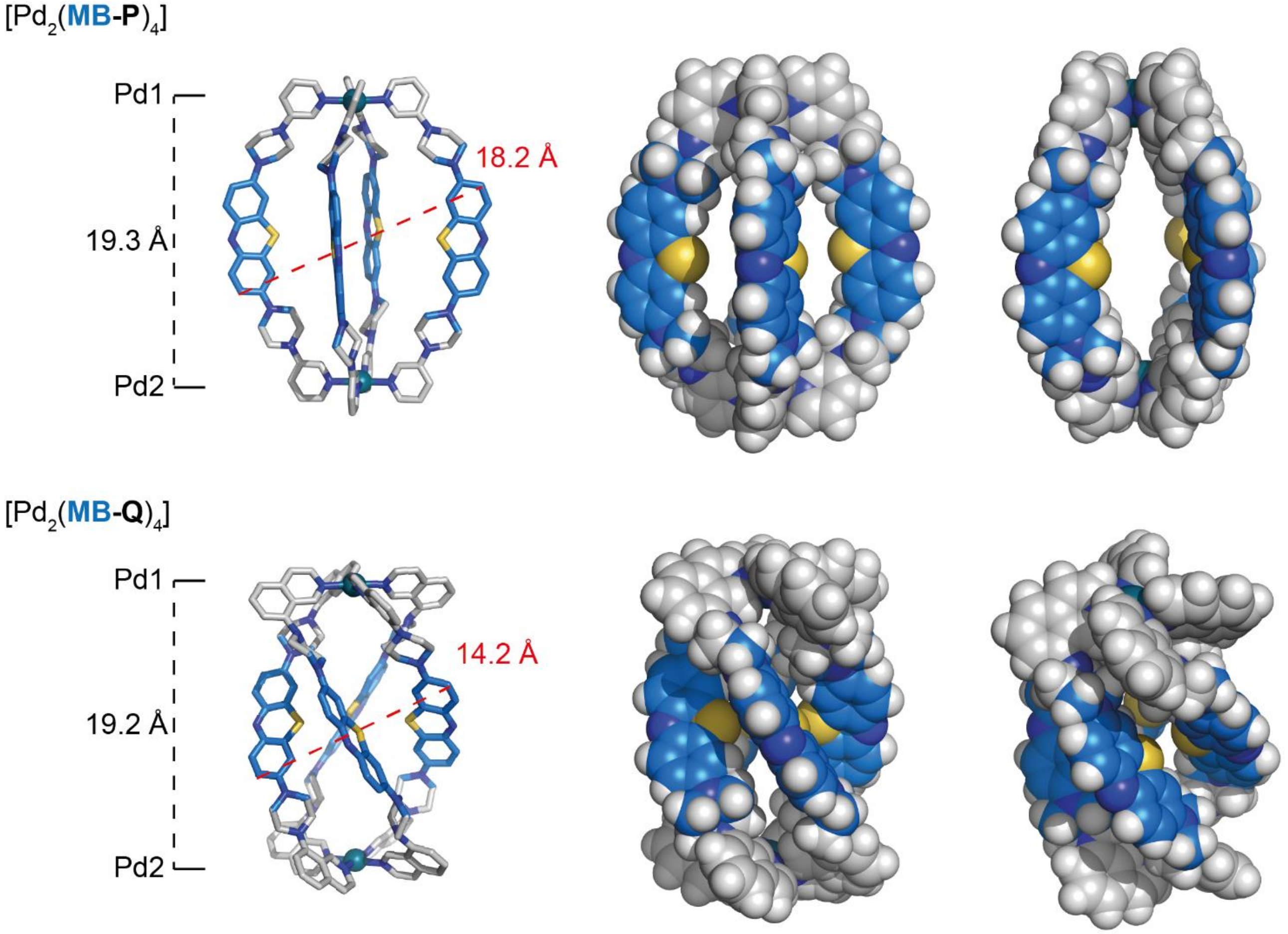


**Figure S115:** Stick and space filling representation of calculated DFT (gas-phase B3LYP/def2-SV(P)) models of [Pd$_2$(**MB-P**)$_4$] and [Pd$_2$(**MB-Q**)$_4$]. Counterions omitted for clarity. For the two structures, in black is indicated the Pd-Pd distance and in red the trasversal distance (the same two C atoms of the **MB** backbones were chosen for the different structures). While the pyridine based ligand coordinates the metal cations almost perfectly perpendicularly to the plane that cuts the Pd-Pd axis in half (with a small extent of propeller disposition of the pyridines around the coordination center), the isoquinoline-based ones are highly twisted. Moreover, the structures are visualized in the space filling mode and the different extent of accessible cavity can be noted. The [Pd$_2$(**DYE-P**)$_4$] cage offers a larger available space for small molecule to bind inside the cavity.

## 5 X-ray Crystal Structures

Four different supramolecular assemblies [$Pd_2$(**MK-P**)$_4$], [$Pd_2$(**RB-P**)$_4$], [$Pd_2$(**CV-P**)$_4$] and [$Pd_3$(**MK-Q**)$_6$] were studied using single-crystal X-ray crystallography. Cage [$Pd_2$(**MK-P**)$_4$] could be studied on an in-house diffractometer using microfocussed CuK$_\alpha$ radiation. In contrast, crystals of the other supramolecular assemblies were extremely sensitive to loss of organic solvent. Due to very thin plate or needle-shaped crystals, the analysis was further hampered by the limited scattering power of the samples not allowing to reach the desired (sub-)atomic resolution using the microfocussed X-ray in-house CuK$_\alpha$ source. Gaining detailed structural insight thus required cryogenic crystal handling and highly brilliant synchrotron radiation. Hence, diffraction data of most of supramolecular assemblies was collected during three beamtime shifts at macromolecular synchrotron beamline P11, PETRA III, DESY.[12] Counterion and solvent flexibility required carefully adapted macromolecular refinement protocols employing geometrical restraint dictionaries, similarity restraints and restraints for anisotropic displacement parameters (ADPs).

| Compound | [$Pd_2$(**MK-P**)$_4$] | [$Pd_2$(**RB-P**)$_4$] | [$Pd_2$(**CV-P**)$_4$] | [$Pd_3$(**MK-Q**)$_6$] |
|---|---|---|---|---|
| CIF ID | **ir92c** | **ir5b_7** | **ir22h** | **ir15** |
| CCDC number | **2035822** | **2035823** | **2035824** | **2035825** |
| Empirical formula | $C_{128}H_{140}B_4F_{16}N_{24}O_6Pd_2S_2$ | $C_{152}H_{136}N_{24}O_{12}Pd_2$ | $C_{156}H_{168}N_{31}O_9Pd_2$ | $C_{240}H_{234}B_2F_8N_{36}O_9Pd_3S_3$ |
| Formula weight | 2734.79 | 2703.64 | 2834.00 | 4355.62 |
| Temperature [K] | 100(2) | 80(2) | 80(2) | 80(2) |
| Crystal system | triclinic | tetragonal | monoclinic | triclinic |
| Space group (number) | $P\overline{1}$ (2) | $I4/\mathrm{m}$ (87) | $P2_1/\mathrm{m}$ (11) | $P\overline{1}$ (2) |
| *a* [Å] | 15.1314(5) | 23.200(3) | 25.502(5) | 20.140(4) |
| *b* [Å] | 16.6317(5) | 23.200(3) | 21.570(4) | 24.726(5) |
| *c* [Å] | 17.0412(4) | 27.606(6) | 26.148(5) | 32.071(6) |
| α [Å] | 94.829(2) | 90 | 90 | 104.73(3) |
| β [Å] | 100.421(2) | 90 | 113.39(3) | 101.04(3) |
| γ [Å] | 92.016(2) | 90 | 90 | 105.04(3) |
| Volume [$Å^3$] | 4197.3(2) | 14859(5) | 13201(5) | 14339(6) |
| *Z* | 1 | 2 | 2 | 2 |
| $\rho_{calc}$ [$g/cm^3$] | 1.082 | 0.604 | 0.713 | 1.009 |
| $\mu$ [$mm^{-1}$] | 2.527 | 0.140 | 0.463 | 0.241 |
| *F*(000) | 1412 | 2808 | 2970 | 4532 |
| Crystal size [$mm^3$] | 0.120×0.100×0.020 | 0.170×0.050×0.040 | 0.050×0.020×0.010 | 0.090×0.020×0.010 |
| Crystal colour | yellow | colorless | violet | yellow |
| Crystal shape | plate | needle | plate | needle |
| Radiation | Cu$K_\alpha$ (λ=1.54178 Å) | synchrotron (λ=0.6888 Å) | synchrotron (λ=1.0332 Å) | synchrotron (λ=0.6888 Å) |
| 2Θ range [°] | 5.30 to 148.99 (0.80 Å) | 2.22 to 38.29 (1.05 Å) | 2.47 to 50.78 (1.20 Å) | 1.32 to 42.98 (0.94 Å) |
| Index ranges | -18 ≤ h ≤ 18<br>-20 ≤ k ≤ 20<br>-21 ≤ l ≤ 20 | -22 ≤ h ≤ 22<br>-22 ≤ k ≤ 22<br>-25 ≤ l ≤ 25 | -21 ≤ h ≤ 21<br>-17 ≤ k ≤ 17<br>-21 ≤ l ≤ 21 | -21 ≤ h ≤ 21<br>-26 ≤ k ≤ 26<br>-34 ≤ l ≤ 34 |
| Reflections collected | 121896 | 43623 | 47588 | 118447 |
| Independent reflections | 17076<br>$R_{int}$ = 0.1085<br>$R_{sigma}$ = 0.0589 | 3402<br>$R_{int}$ = 0.0758<br>$R_{sigma}$ = 0.0371 | 7661<br>$R_{int}$ = 0.0615<br>$R_{sigma}$ = 0.0404 | 34065<br>$R_{int}$ = 0.0574<br>$R_{sigma}$ = 0.0528 |
| Completeness to θ = 67.679° | 99.8 % | 97.9 % | 93.1 % | 94.2 % |

| | | | | |
|---|---|---|---|---|
| Data / Restraints / Parameters | 17076/2346/1021 | 3402/965/287 | 7661/1726/889 | 34065/5456/2746 |
| Goodness-of-fit on $F^2$ | 1.036 | 2.020 | 1.769 | 1.300 |
| Final $R$ indexes [$I \geq 2\sigma(I)$] | $R_1$ = 0.0970<br>w$R_2$ = 0.2692 | $R_1$ = 0.1618<br>w$R_2$ = 0.4435 | $R_1$ = 0.1372<br>w$R_2$ = 0.4081 | $R_1$ = 0.1026<br>w$R_2$ = 0.3201 |
| Final $R$ indexes [all data] | $R_1$ = 0.1261<br>w$R_2$ = 0.2972 | $R_1$ = 0.1806<br>w$R_2$ = 0.4654 | $R_1$ = 0.1626<br>w$R_2$ = 0.4335 | $R_1$ = 0.1326<br>w$R_2$ = 0.3445 |
| Largest peak/hole [eÅ$^3$] | 1.47/-1.38 | 1.83/-0.65 | 0.78/-0.58 | 1.43/-0.89 |

## 5.1 Crystal structure of [$Pd_2$(**MK-P**)$_4$]

Yellow plate crystals of [$Pd_2$(**MK-P**)$_4$] were grown by slow vapor diffusion of toluene in the DMSO solution of [$Pd_2$(**MK-P**)$_4$] at room temperature. A single crystal in mother liquor was mounted onto a 0.1 mm nylon loop using NVH oil. Single crystal X-ray diffraction data was collected on a Bruker D8 venture equipped with an Incoatec microfocus source (Iμs 2.0) using Cukα radiation on a four axis κ-goniometer, equipped with an Oxford Cryostream 800 and a Photon 100 detector. Data integration was done with SAINT. Data scaling and absorption correction were performed with SADABS. The space group was determined using XPREP.[21] The structure was solved by intrinsic phasing/direct methods using SHELXT[14] and refined with SHELXL[15] for full-matrix least-squares routines on $F^2$ and ShelXle[16] as a graphical user interface.

Three of the four piperazine rings in the two ligands of the asymmetric unit as well as the co-crystallized DMSO solvent molecule were disordered and modelled with two discrete positions refining their occupancy factor using a free variable and ensuring sensible geometry by employing stereochemical restraints.

Stereochemical restraints for the **MK-P** ligands (residue class BPP) were generated by the GRADE program using the GRADE Web Server (http://grade.globalphasing.org) and applied in the refinement. A GRADE dictionary for SHELXL contains target values and standard deviations for 1,2-distances (DFIX) and 1,3-distances (DANG), as well as restraints for planar groups (FLAT). All displacements for non-hydrogen atoms were refined anisotropically. The refinement of ADP's for carbon, nitrogen and oxygen atoms was enabled by a combination of similarity restraints (SIMU) and rigid bond restraints (RIGU).[18] The contribution of the electron density from disordered counterions and solvent molecules, which could not be modeled with discrete atomic positions were handled using the SQUEEZE[19] routine in PLATON.[20] The solvent mask file (.fab) computed by PLATON were included in the SHELXL refinement via the ABIN instruction leaving the measured intensities untouched.

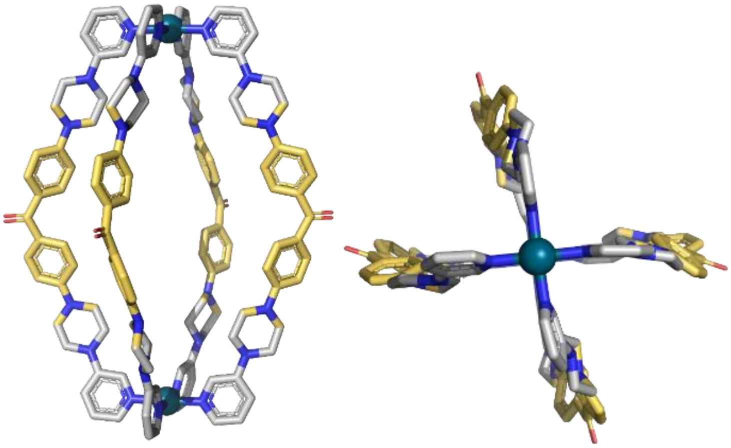

**Figure S116:** Two views of the X-ray crystal structure of [$Pd_2$(**MK-P**)$_4$]. Counterions and solvent molecules have been omitted for clarity.

## 5.2 Crystal structure of [$Pd_2$(**RB-P**)$_4$]

Colorless, needle shaped crystals of [$Pd_2$(**RB-P**)$_4$] were grown by slow vapor diffusion of ethyl acetate in the DMSO solution of [$Pd_2$(**RB-P**)$_4$] at room temperature. A single crystal of [$Pd_2$(**RB-P**)$_4$] in mother liquor was pipetted onto a glass slide containing NVH oil. To avoid collapse of the crystal lattice, the crystal was quickly mounted onto a 0.15 mm nylon loop and immediately flash cooled in liquid nitrogen. Crystals were stored at cryogenic temperature in dry shippers, in which they were safely transported to macromolecular beamline P11 at Petra III,[12] DESY Hamburg, Germany. A wavelength of $\lambda$ = 0.6888 Å was chosen using a liquid $N_2$ cooled double crystal monochromator. Single crystal X-ray diffraction data was collected at 80(2) K on a single axis goniometer, equipped with an Oxford Cryostream 800 and Pilatus 6M detector. 1800 diffraction images were collected in a 360° ϕ sweep at a detector distance of 190 mm, 100% filter transmission, 0.2° step width and 0.1 seconds exposure time per image. Data integration and reduction were undertaken using XDS.[13] The structure was solved by intrinsic phasing/direct methods using SHELXT[14] and refined with SHELXL[15] using 22 CPU cores for full-matrix least-squares routines on $F^2$ and ShelXle[16] as a graphical user interface and the DSR program plugin was employed for modeling.[17]

High space group symmetry was broken by disorder of the rhodamine backbone part located in the mirror plane. It was modelled with two discrete positions refining their occupancy factor using a free variable. Disorder and extremely poor crystal quality required stereochemical restraints to be employed for ensuring a sensible geometry of the organic part of the structure.

Stereochemical restraints for the **RB-P** ligands (residue class RPP) were generated by the GRADE program using the GRADE Web Server (http://grade.globalphasing.org) and applied in the refinement. A GRADE dictionary for SHELXL contains target values and standard deviations for 1,2-distances (DFIX) and 1,3-distances (DANG), as well as restraints for planar groups (FLAT). All displacements for non-hydrogen atoms were refined anisotropically. The refinement of ADP's for carbon, nitrogen and oxygen atoms was enabled by a combination of similarity restraints (SIMU) and rigid bond restraints (RIGU).[18] The contribution of the electron density from disordered counterions and solvent molecules, which could not be modeled with discrete atomic positions were handled using the SQUEEZE[19] routine in PLATON.[20] The solvent mask file (.fab) computed by PLATON were included in the SHELXL refinement via the ABIN instruction leaving the measured intensities untouched.

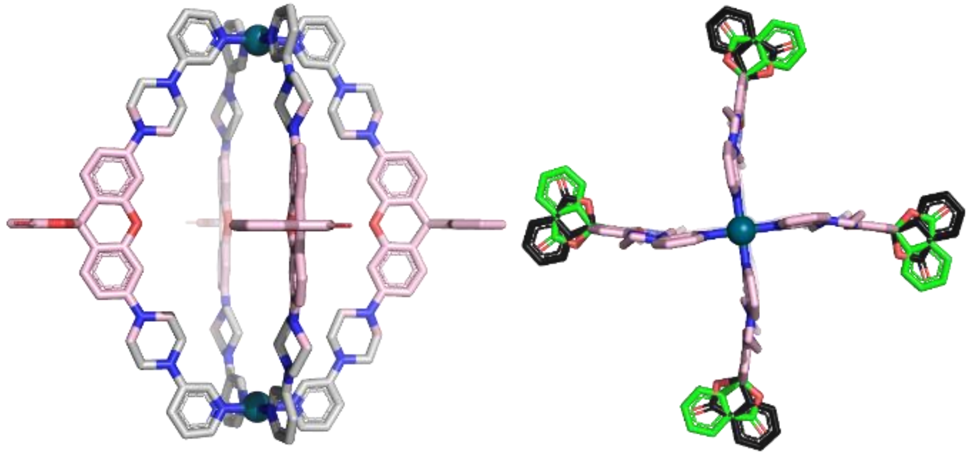

**Figure S117:** Two views of the X-ray crystal structure of [$Pd_2$(**RB-P**)$_4$]. Counterions and solvent molecules have been omitted for clarity.

### 5.3 Crystal structure of [$Pd_2$(**CV-P**)$_4$]

Violet plate crystals of [$Pd_2$(**CV-P**)$_4$] were grown by slow vapor diffusion of methyl tert-butyl ether in the dimethylformamide solution of [$Pd_2$(**CV-P**)$_4$] at room temperature. A single crystal of [$Pd_2$(**CV-P**)$_4$] in mother liquor was pipetted onto a glass slide containing NVH oil. To avoid collapse of the crystal lattice, the crystal was quickly mounted onto a 0.2 mm nylon loop and immediately flash cooled in liquid nitrogen. Crystals were stored at cryogenic temperature in dry shippers, in which they were safely transported to macromolecular beamline P11 at Petra III,[12] DESY Hamburg, Germany. A wavelength of $\lambda$ = 1.0332Å was chosen using a liquid $N_2$ cooled double crystal monochromator. Single crystal X-ray diffraction data was collected at 80(2) K on a single axis goniometer, equipped with an Oxford Cryostream 800 and Pilatus 6M detector. 3600 diffraction images were collected in a 360° ϕ sweep at a detector distance of 200 mm, 13.4% filter transmission, 0.1° step width and 0.1 seconds exposure time per image. Data integration and reduction were undertaken using XDS.[13] The structure was solved by intrinsic phasing/direct methods using SHELXT[14] and refined with SHELXL[15] using 22 cpu cores for full-matrix least-squares routines on $F^2$ and ShelXle[16] as a graphical user interface and the DSR program plugin was employed for modeling.[17]

All three nitrate counterions were disordered over special position (mirror plane) and occupancy factor were therefore fixed to 50%. Poor crystal quality and low experimental resolution of 1.2 Å required stereochemical restraints to be employed for ensuring a sensible geometry of the organic part of the structure.

Stereochemical restraints for the **CV-P** ligands (residue class CVP) were generated by the GRADE program using the GRADE Web Server (http://grade.globalphasing.org) and applied in the refinement. A GRADE dictionary for SHELXL contains target values and standard deviations for 1,2-distances (DFIX) and 1,3-distances (DANG), as well as restraints for planar groups (FLAT). All displacements for non-hydrogen atoms were refined anisotropically. The refinement of ADP's for carbon, nitrogen and oxygen atoms was enabled by a combination of similarity restraints (SIMU) and rigid bond restraints (RIGU).[18] Despite the use of these restraints, anisotropic refinement of disordered nitrate counterions remained unstable, so that all their atoms were only refined isotropically. The contribution of the electron density from disordered counterions and solvent molecules, which could not be modeled with discrete atomic positions were handled using the SQUEEZE[19] routine in PLATON.[20] The solvent mask file (.fab) computed by PLATON were included in the SHELXL refinement via the ABIN instruction leaving the measured intensities untouched.

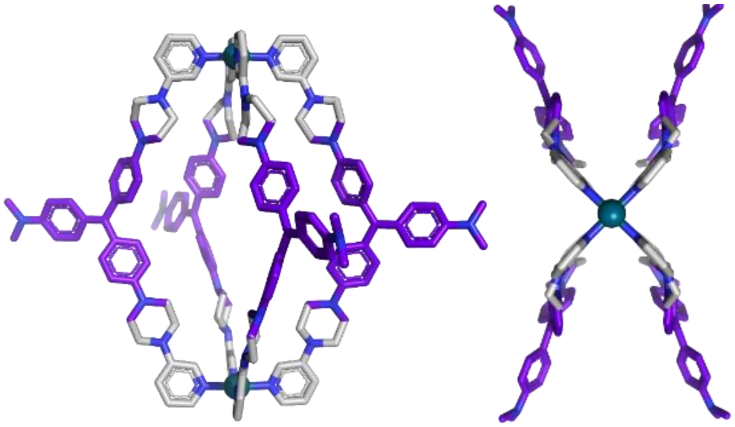

**Figure S118:** Two views of the X-ray crystal structure of [$Pd_2$(**CV-P**)$_4$]. Counterions and solvent molecules have been omitted for clarity.

### 5.4 Crystal structure of [$Pd_3$(**MK-Q**)$_6$]

Yellow, needle shaped crystals of [$Pd_3$(**MK-Q**)$_6$] were grown by slow vapor diffusion of toluene in the DMSO solution of [$Pd_2$(**MK-Q**)$_4$] at room temperature. A single crystal of [$Pd_3$(**MK-Q**)$_6$] in mother liquor was pipetted onto a glass slide containing NVH oil. To avoid collapse of the crystal lattice, the crystal was quickly mounted onto a 0.1 mm nylon loop and immediately flash cooled in liquid nitrogen. Crystals were stored at cryogenic temperature in dry shippers, in which they were safely transported to macromolecular beamline P11 at Petra III,[12] DESY Hamburg, Germany. A wavelength of $\lambda$ = 0.6888 Å was chosen using a liquid $N_2$ cooled double crystal monochromator. Single crystal X-ray diffraction data was collected at 80(2) K on a single axis goniometer, equipped with an Oxford Cryostream 800 and a Pilatus 6M detector. 3600 diffraction images 36were collected in a 360° ϕ sweep at a detector distance of 154.1 mm, 75.5% filter transmission, 0.1° step width and 0.1 seconds exposure time per image. Data integration and reduction were undertaken using XDS.[13] The structure was solved by intrinsic phasing/direct methods using SHELXT[14] and refined with SHELXL[15] using 22 cpu cores for full-matrix least-squares routines on $F^2$ and ShelXle[16] as a graphical user interface and the DSR program plugin was employed for modeling.[17]

Poor crystal quality and high flexibility required stereochemical restraints to be employed for ensuring a sensible geometry of the organic part of the structure.

Stereochemical restraints for the **MK-Q** ligands (residue class BPQ) were generated by the GRADE program using the GRADE Web Server (http://grade.globalphasing.org) and applied in the refinement. A GRADE dictionary for SHELXL contains target values and standard deviations for 1,2-distances (DFIX) and 1,3-distances (DANG), as well as restraints for planar groups (FLAT). All displacements for non-hydrogen atoms were refined anisotropically. The refinement of ADP's for carbon, nitrogen and oxygen atoms was enabled by a combination of similarity restraints (SIMU) and rigid bond restraints (RIGU).[18] The contribution of the electron density from disordered counterions and solvent molecules, which could not be modeled with discrete atomic positions were handled using the SQUEEZE[19] routine in PLATON.[20] The solvent mask file (.fab) computed by PLATON were included in the SHELXL refinement via the ABIN instruction leaving the measured intensities untouched.

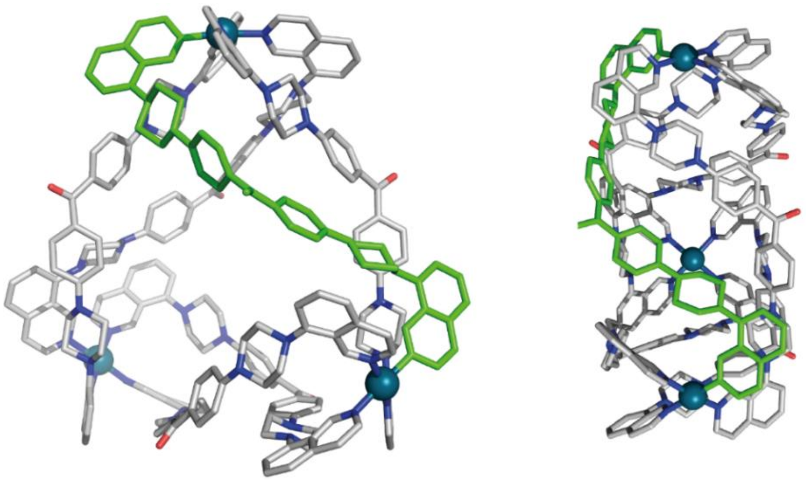

**Figure S119:** Two views of the X-ray crystal structure of one of the found enantiomers for [$Pd_3$(**MK-Q**)$_6$]. One ligand has been colored green for better comprehension of the structure. Counterions and solvent molecules have been omitted for clarity.

# 6 UV-Vis absorption spectroscopy

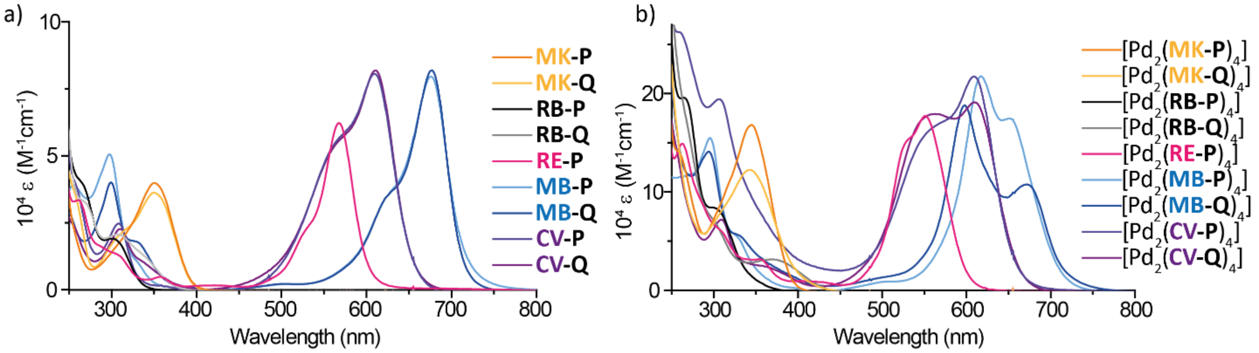


**Figure S120:** Absorption spectra in DMSO of a) the ligands **MK-P** (yellow), **MK-Q** (orange), **RB-P** (black) **RB-Q** (grey), **RE-P** (pink) **MB-P** (light blue), **MB-Q** (dark blue), **CV-P** (light violet), **CV-Q** (dark violet) and b) corresponding cages and helicates (with the same color scheme). Molar extinction coefficients (given on the y-axis) were extrapolated with linear regression from absoptions recorded at concentrations of a) the ligands 62.5 µM, 93.76 µM and 125 µM and b) the assemblies 15.6 µM, 23.44 µM and 31.25 µM in cuvettes with an optical path of 0.1 cm.

# 7 Host-Guest Chemistry

## 7.1 (R)-camphor sulfonate (**CSA**) as tetrabutyl ammonium salt[3]

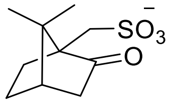


(*R*)-**CSA**

### 7.1.1 UV-Vis and CD spectroscopy

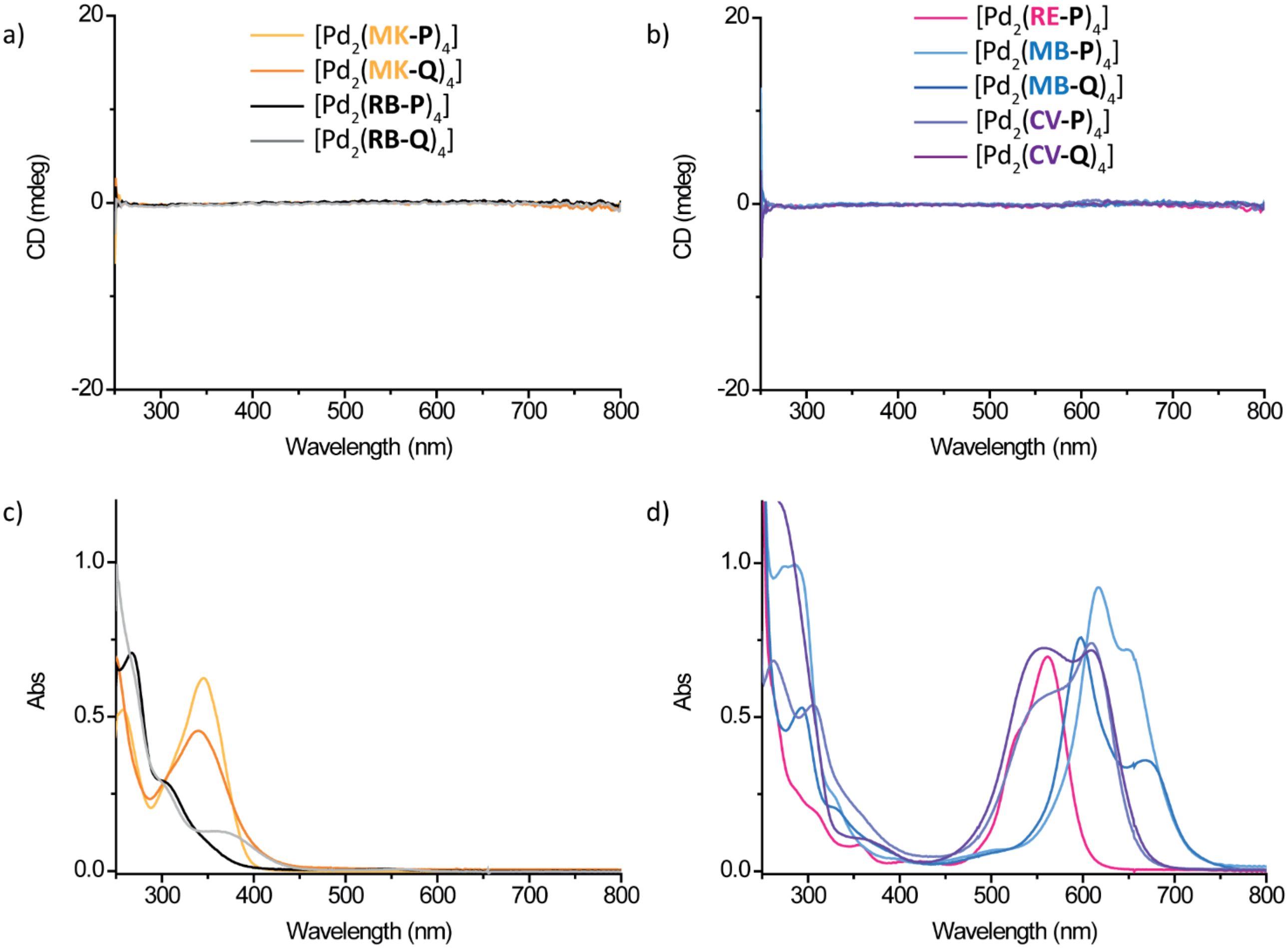


**Figure S121:** 1:1 solution (18.75 µM) in DMSO at 25 °C of the dye-based cages, helicates and guest (*R*)-**CSA**. a) CD and c) UV-Vis absorption spectra of the cages and helicates that show absorption below 400 nm: [$Pd_2$(**MK-P**)$_4$], [$Pd_2$(**MK-Q**)$_4$], [$Pd_2$(**RB-P**)$_4$] and [$Pd_2$(**RB-Q**)$_4$]. b) CD and d) UV-Vis absorption spectra of the cages and helicates that absorb also at higher wavelengths: [$Pd_2$(**RE-P**)$_4$], [$Pd_2$(**MB-P**)$_4$], [$Pd_2$(**MB-Q**)$_4$], [$Pd_2$(**CV-P**)$_4$] and [$Pd_2$(**CV-Q**)$_4$].

### 7.1.2 $^{1}$H NMR titration

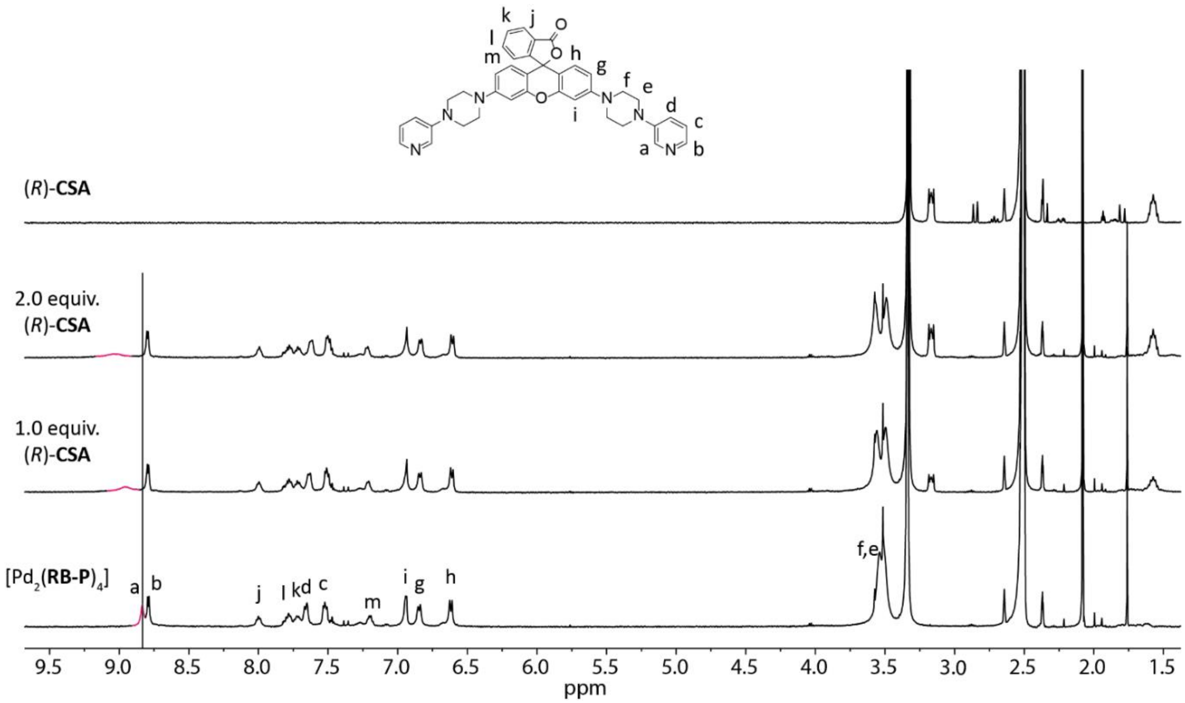


**Figure S122:** $^1$H NMR spectra at 25 °C of cage [$Pd_2$(**RB-P**)$_4$], with 1 and 2 equiv. of guest (*R*)-**CSA** and guest (*R*)-**CSA** alone.

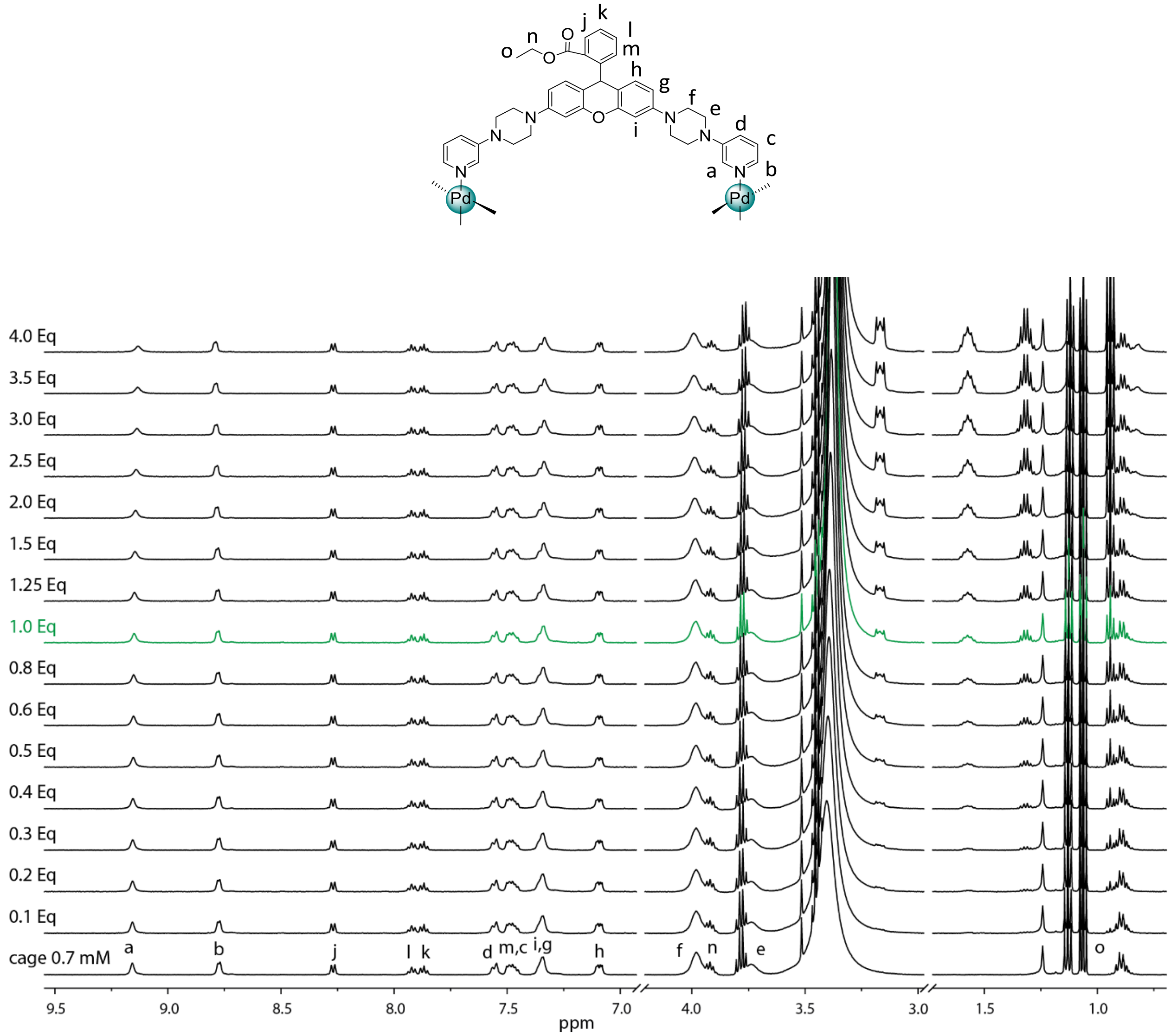


**Figure S 123:** $^{1}H$ NMR titration at 25 °C of guest (*R*)-**CSA** in cage $[Pd_2($**RE-P**$)_4]$. The green line corresponds to 1 equiv. of the guest.

### 7.1.3 ESI-MS spectra

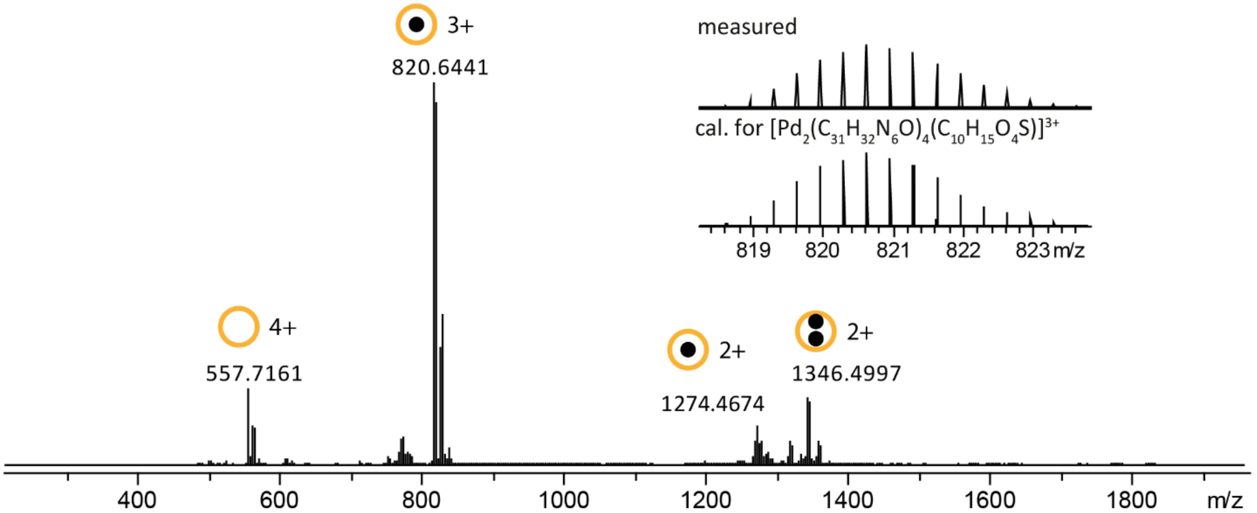


**Figure S124:** ESI-MS spectrum of cage [$Pd_2$(**MK-P**)$_4$] (yellow circle) with guest (*R*)-**CSA** (black dot) [(*R*)-**CSA**@$Pd_2$(**MK-P**)$_4$+n$BF_4$]$^{3-n+}$, n=0,1 with measured and calculated isotopic pattern of [(*R*)-**CSA**@$Pd_2$(**MK-P**)$_4$]$^{3+}$.

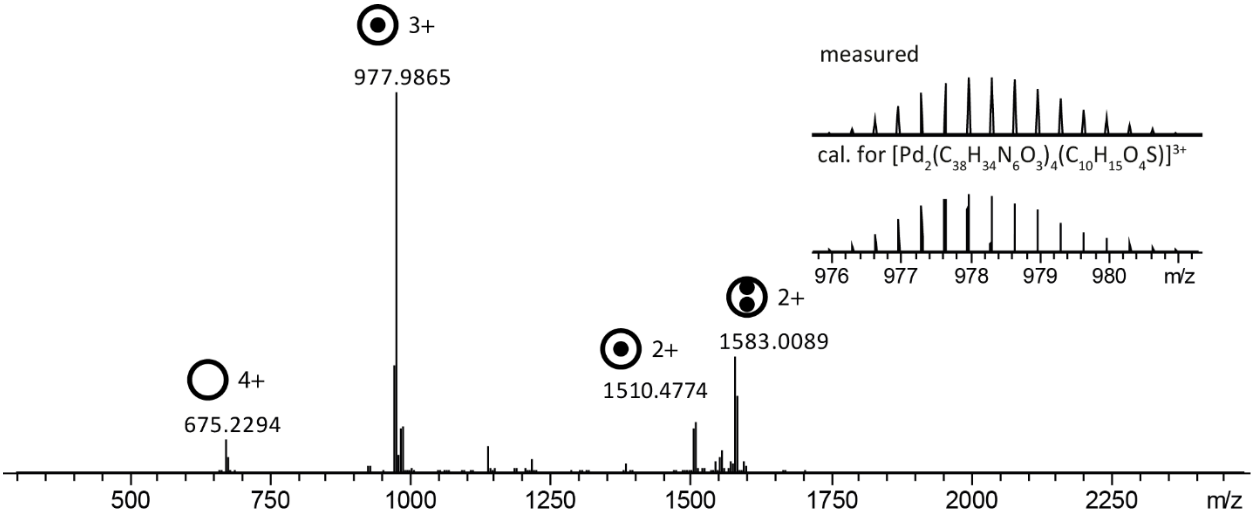


**Figure S125** ESI-MS spectrum of cage [$Pd_2$(**RB-P**)$_4$] (black circle) with guest (*R*)-**CSA** (black dot) [(*R*)-**CSA@**$Pd_2$(**RB-P**)$_4$+n$BF_4$]$^{3-n+}$, n=0,1 with measured and calculated isotopic pattern of [(*R*)-**CSA**@$Pd_2$(**RB-P**)$_4$]$^{3+}$.

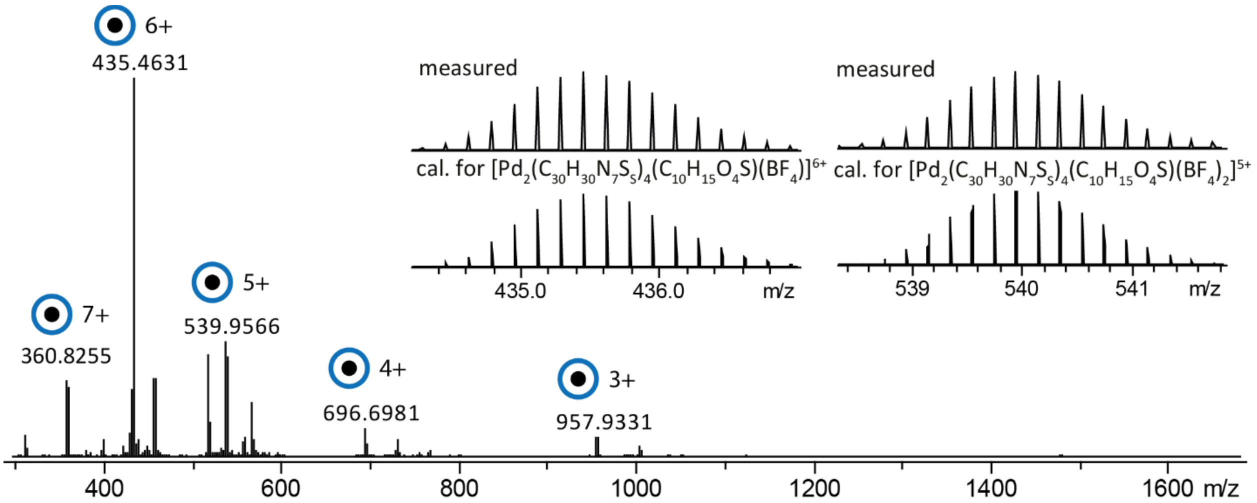


**Figure S126:** ESI-MS spectrum of cage [$Pd_2$(**MB-P**)$_4$] (blue circle) with guest (*R*)-**CSA** (black dot) [(*R*)-**CSA**@$Pd_2$(**MB-P**)$_4$+n$BF_4$]$^{7-n+}$, n=0-4 with measured and calculated isotopic pattern of [(*R*)-**CSA**@$Pd_2$(**MB-P**)$_4$+$BF_4$]$^{6+}$.

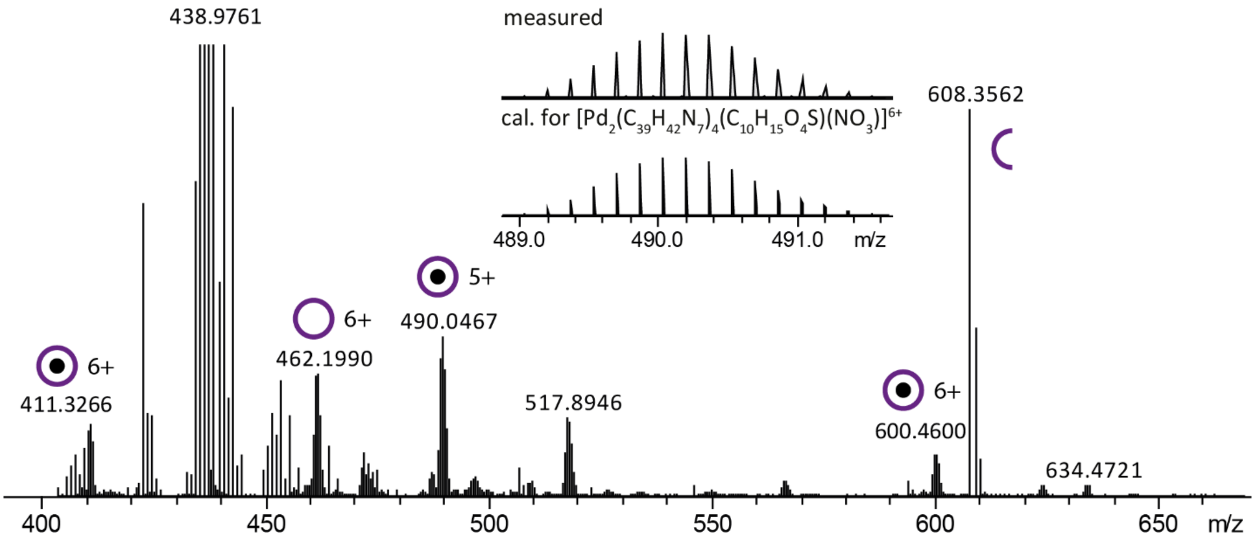


**Figure S127:** ESI-MS spectrum of cage [$Pd_2$(**CV-P**)$_4$] (violet circle) with guest (*R*)-**CSA** (black dot) [(*R*)-**CSA**@$Pd_2$(**CV-P**)$_4$+n$NO_3$]$^{7-n+}$, n=1-3 with measured and calculated isotopic pattern of [(*R*)-**CSA**@$Pd_2$(**CV-P**)$_4$+$NO_3$]$^{6+}$.

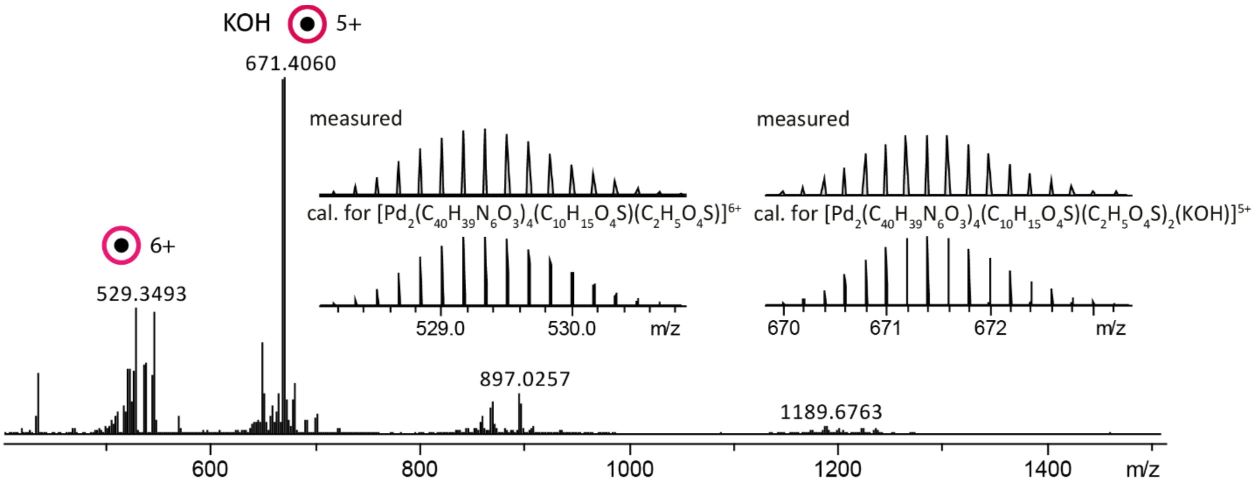


**Figure S128:** ESI-MS spectrum of cage [$Pd_2$(**RE-P**)$_4$] (pink circle) with guest (*R*)-**CSA** (black dot) [(*R*)-**CSA**@$Pd_2$(**RE-P**)$_4$]+n$EtSO_4$]$^{7-n+}$, n=1,2 (for n = 2 an adduct with KOH is suggested) with measured and calculated isotopic patterns of [(*R*)-**CSA**@$Pd_2$(**RE-P**)$_4$($C_2H_5O_4S$)]$^{6+}$ and tentative [(*R*)-**CSA**@$Pd_2$(**RE-P**)$_4$($C_2H_5O_4S$)$_2$(KOH)]$^{5+}$.

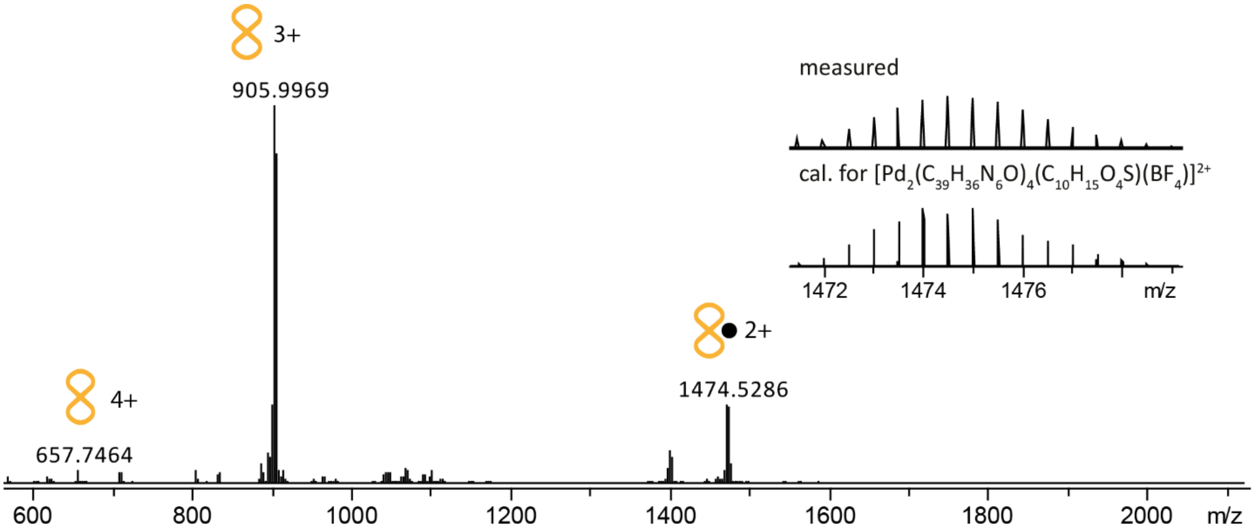


**Figure S129:** ESI-MS spectrum of helicate [$Pd_2$(**MK-Q**)$_4$] (twisted yellow circle) with guest (*R*)-**CSA** (black dot) [(*R*)-**CSA**+$Pd_2$(**MK-Q**)$_4$]+n$BF_4$]$^{3-n+}$, n=1 with measured and calculated isotopic pattern of [(*R*)-**CSA**+$Pd_2$(**MK-Q**)$_4$+$BF_4$]$^{2+}$.

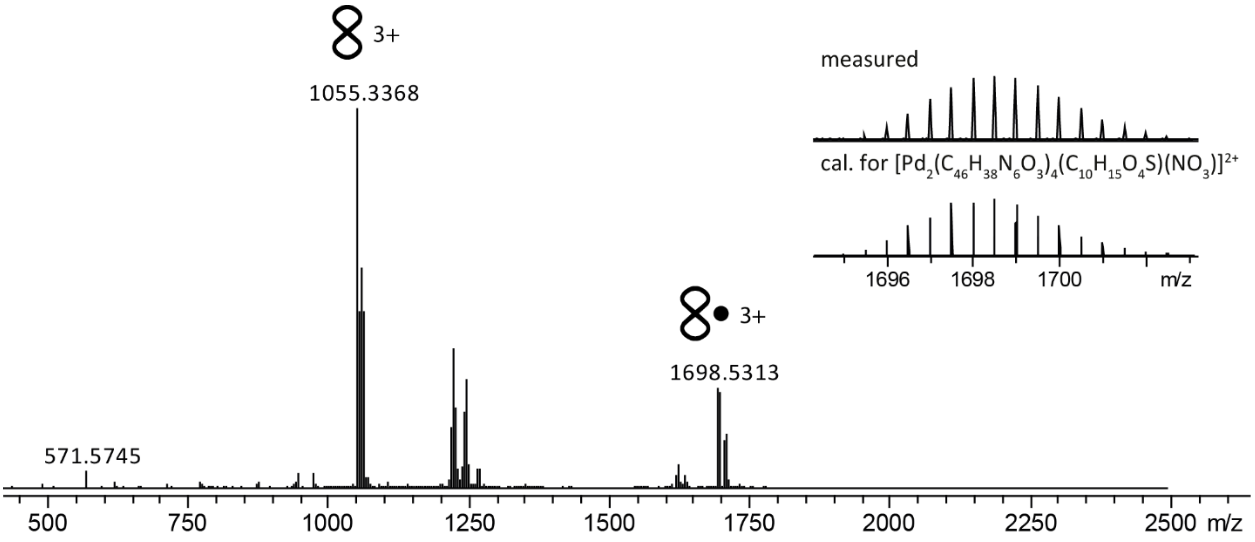


**Figure S130:** ESI-MS spectrum of helicate [$Pd_2$(**RB-Q**)$_4$] (twisted black circle) with guest (*R*)-**CSA** (black dot) [(*R*)- **CSA**+$Pd_2$(**RB-Q**)$_4$+n$NO_3$]$^{3-n+}$, n=1 with measured and calculated isotopic pattern of [(*R*)-**CSA**+$Pd_2$(**RB-Q**)$_4$+$NO_3$]$^{2+}$.

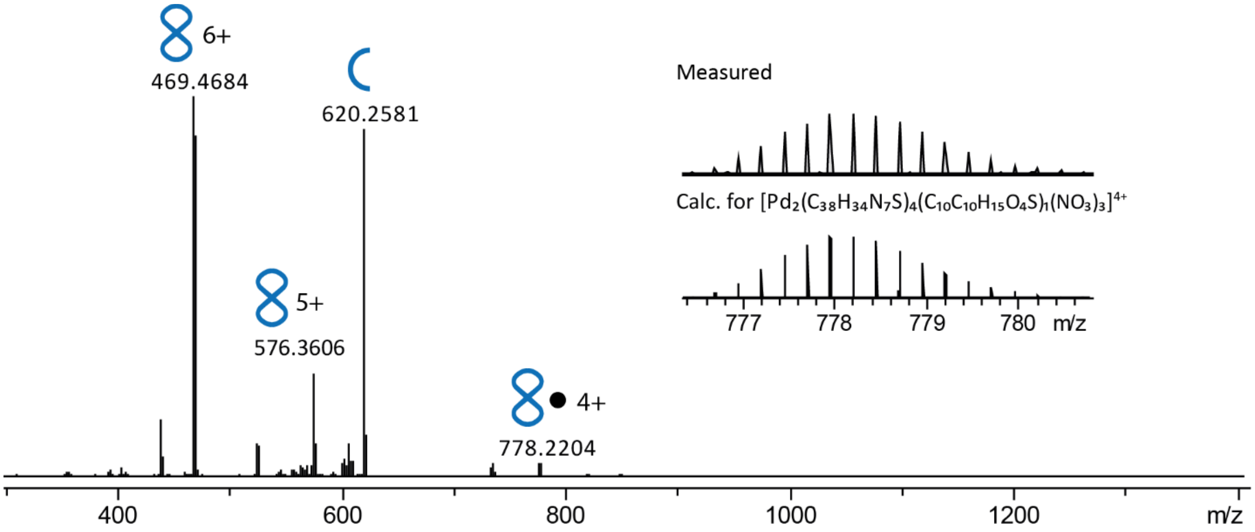


**Figure S131:** ESI-MS spectrum of helicate [$Pd_2$(**MB-Q**)$_4$] (twisted blue circle) with guest (*R*)-**CSA** (black dot) [(*R*)-**CSA**+$Pd_2$(**MB-Q**)$_4$+n$NO_3$]$^{3-n+}$, n=3 with measured and calculated isotopic pattern of [(*R*)-**CSA**+$Pd_2$(**MB-Q**)$_4$+3$NO_3$]$^{4+}$.

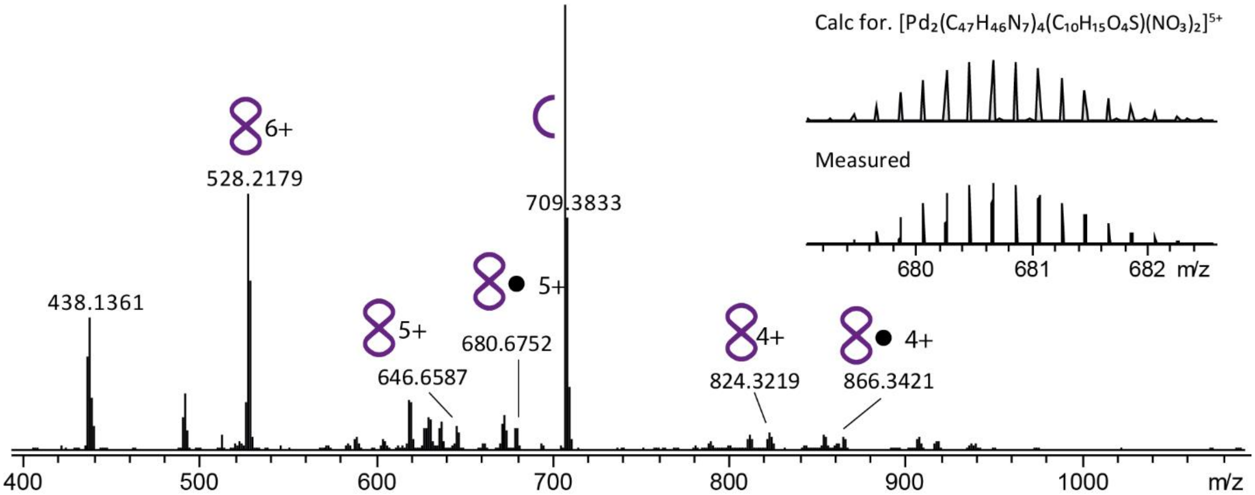


**Figure S132:** ESI-MS spectrum of helicate [$Pd_2$(**CV-Q**)$_4$] (twisted violet circle) with guest (*R*)-**CSA** (black dot) [(*R*)-**CSA**+$Pd_2$(**CV-Q**)$_4$ n$NO_3$]$^{3-n+}$, n=2,3 with measured and calculated isotopic pattern of [(*R*)-**CSA**+$Pd_2$(**CV-Q**)$_4$+2$NO_3$]$^{5+}$.

## 7.2 Dipotassium (R)-1,1'-binaphthyl-2,2'-disulfonate (**BINSO$_3$**)

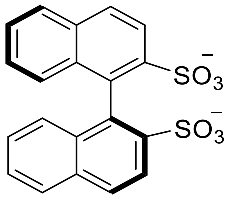


(*R*)-**BINSO$_3$**

### 7.2.1 UV-Vis and CD spectroscopy

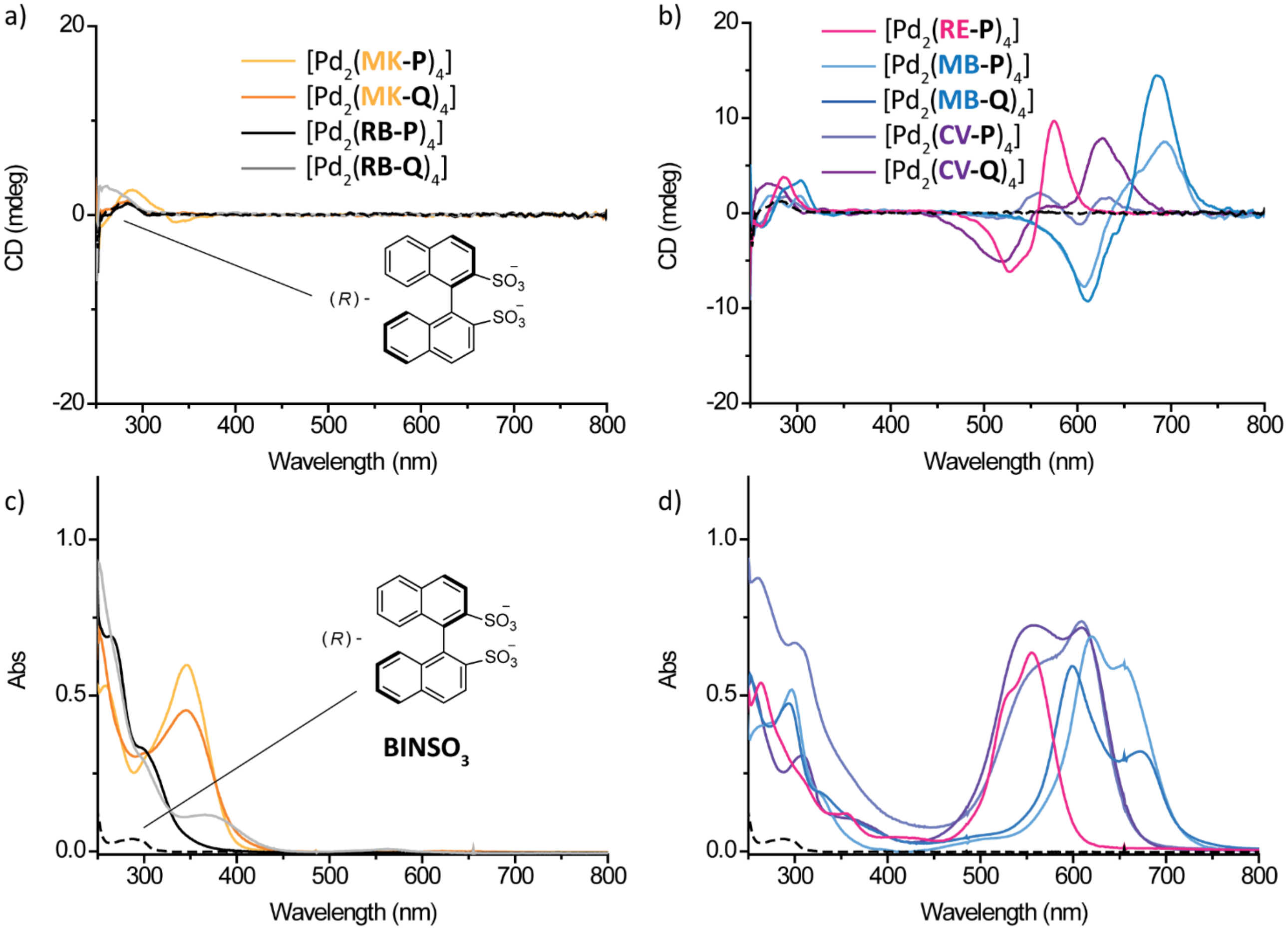


**Figure S133:** 1:1 solution (18.75 µM) in DMSO at 25 °C of the dye-based cages, helicates and guest (*R*)-**BINSO₃**. a) CD and c) UV-Vis absorption spectra of the cages and helicates that show absorption below 400 nm: [$Pd_2$(**MK-P**)$_4$], [$Pd_2$(**MK-Q**)$_4$], [$Pd_2$(**RB-P**)$_4$] and [$Pd_2$(**RB-Q**)$_4$]. b) CD and d) UV-Vis absorption spectra of the cages and helicates that absorb also at higher wavelengths: [$Pd_2$(**RE-P**)$_4$], [$Pd_2$(**MB-P**)$_4$], [$Pd_2$(**MB-Q**)$_4$], [$Pd_2$(**CV-P**)$_4$] and [$Pd_2$(**CV-Q**)$_4$].

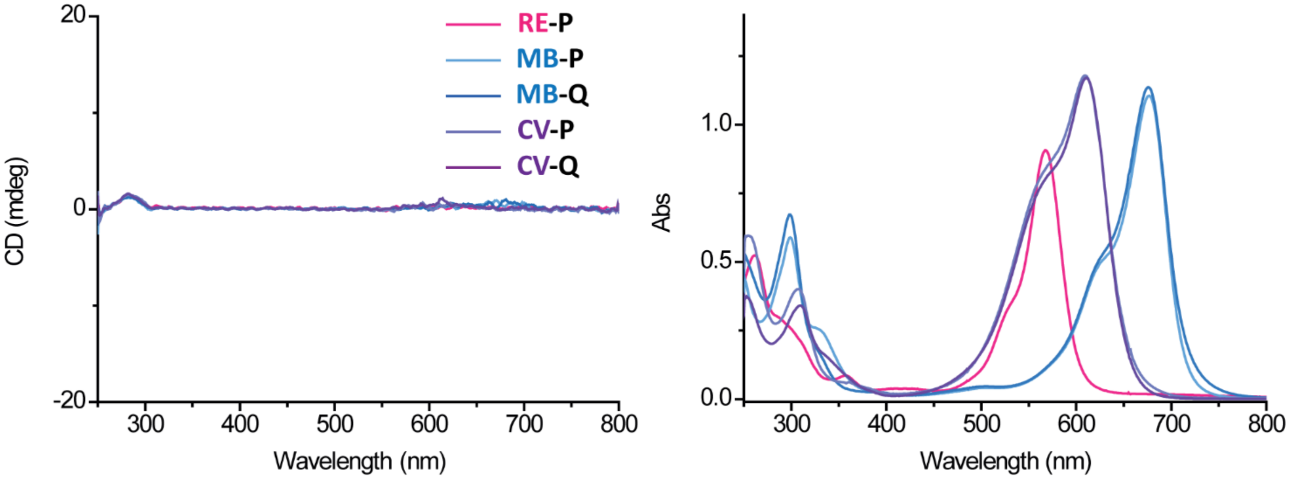


**Figure S134:** a) CD and b) UV-Vis absorption spectra of 1:1 solutions (18.75 µM) in DMSO at 25 °C of the dye-based ligands **RE-P**, **MB-P**, **MB-Q**, **CV-P**, **CV-Q** and the guest (*R*)-**$BINSO_3$**.

### 7.2.2 ¹H NMR titration

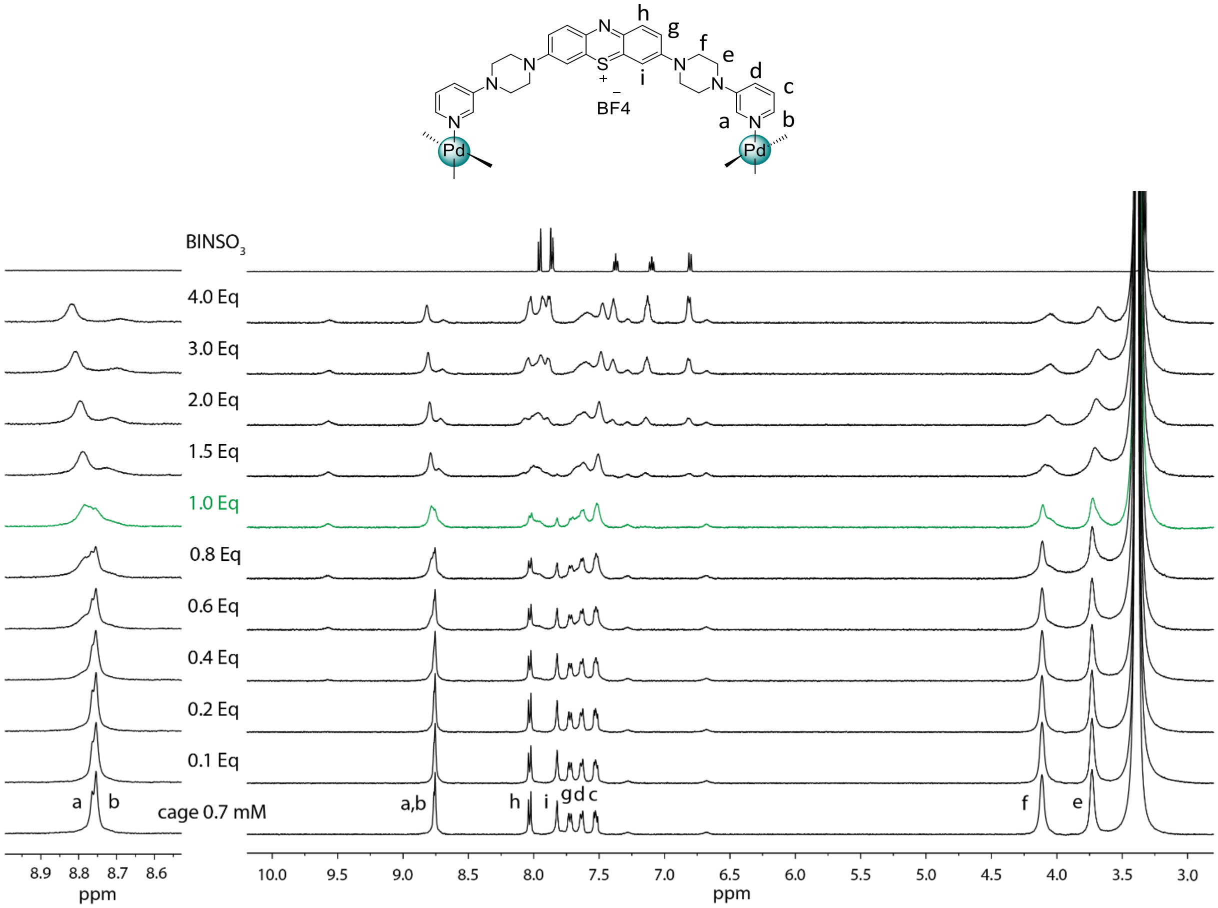


**Figure S135:** ¹H NMR titration at 25 °C of guest (*R*)-**$BINSO_3$** in cage [$Pd_2$(**MB-P**)$_4$]. The green line corresponds to 1 equiv. of the guest. On the left: enlargement of the region 9.0-8.4 ppm, on the right: the full spectrum.

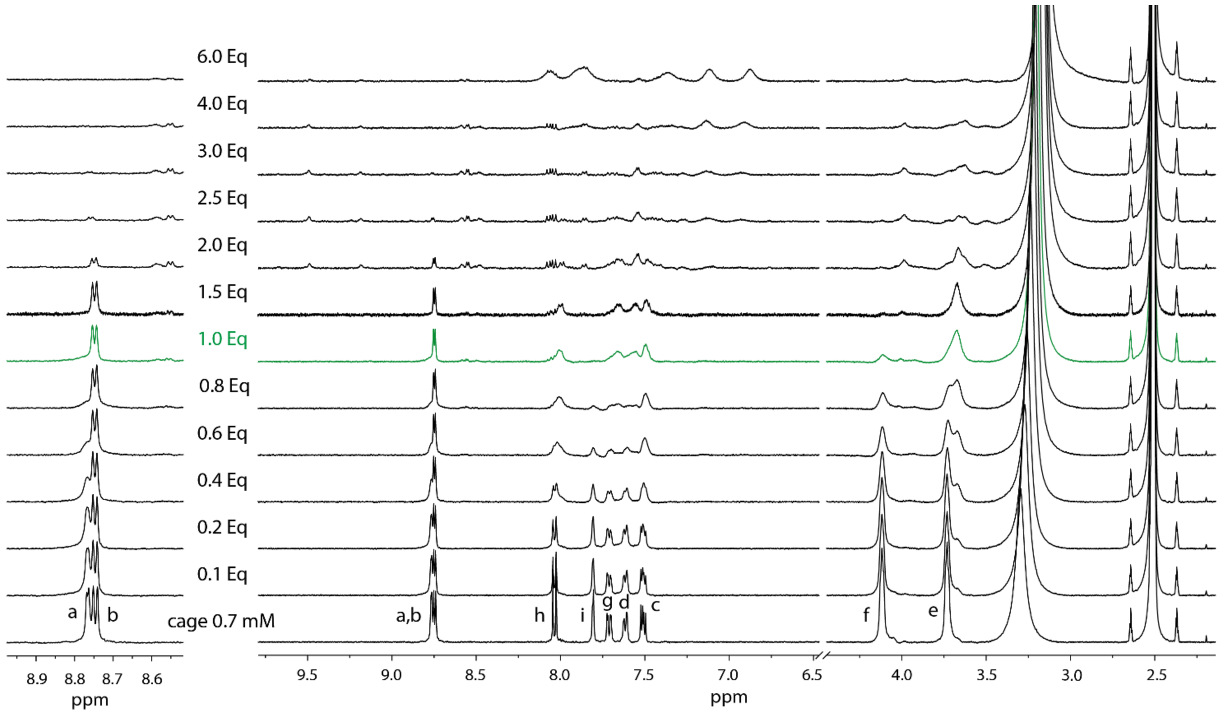


**Figure S136:** $^{1}$H NMR titration at 65 °C of guest (*R*)-**BINSO₃** in cage [Pd$_2$(**MB-P**)$_4$]. The green line corresponds to 1 equiv. of the guest. On the left: enlargement of the region 9.0-8.4 ppm, on the right: the full spectrum. We performed this experiment in order to escape the coalescence temperature. Unfortunately, also in this case we observe line broadening, but at least it becomes clear, observing protons **a** and **i** which point inside the cage cavity, that the guest is binding inside the cage, since only these signals gradually disappear upon stepwise addition of the guest until they are completely gone at 1 equiv.

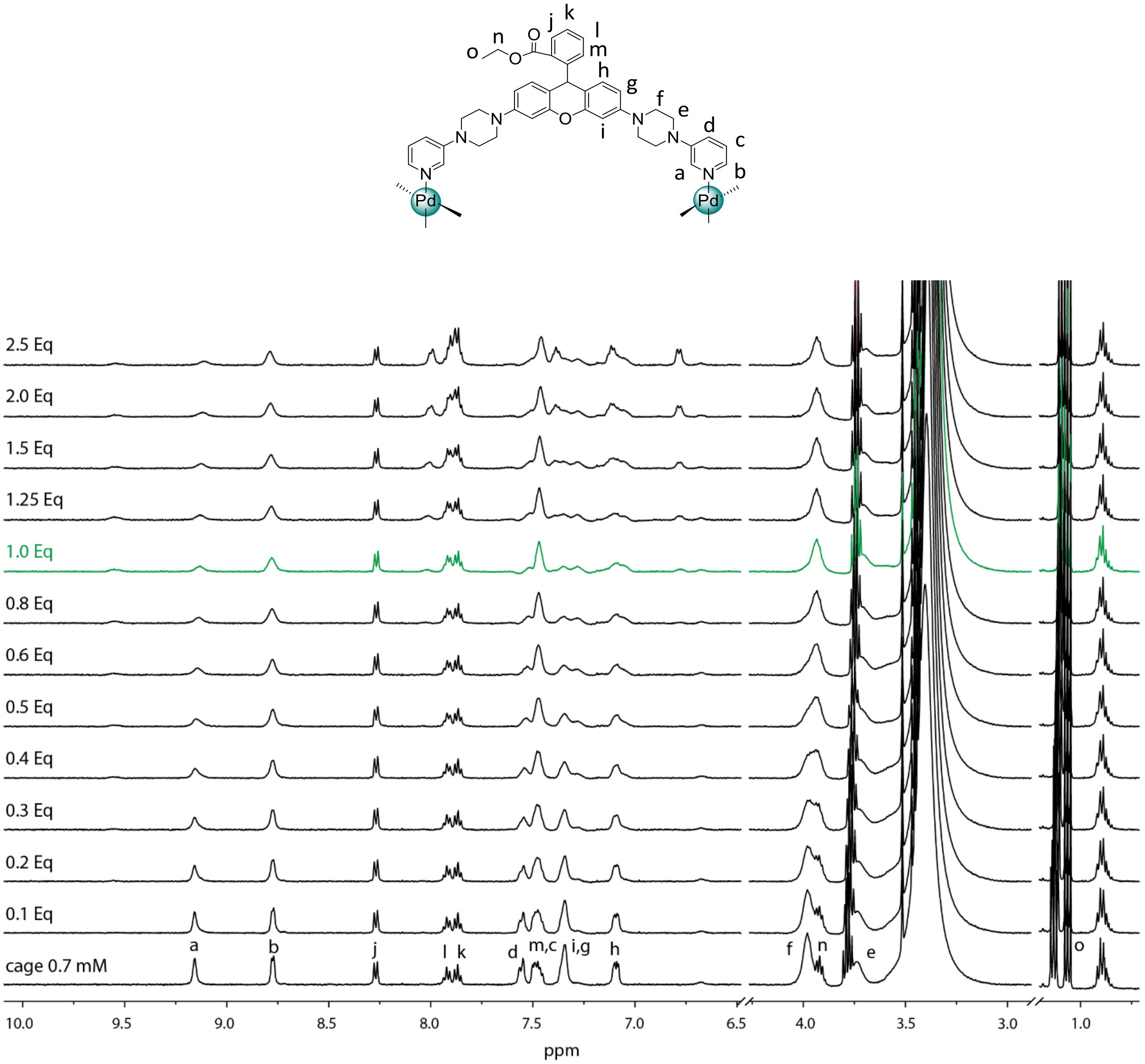


**Figure S137:** $^{1}$H NMR titration at 25 °C of guest (*R*)-**BINSO$_3$** in cage [Pd$_2$(**RE-P**)$_4$]. The green line corresponds to 1 equiv. of the guest.

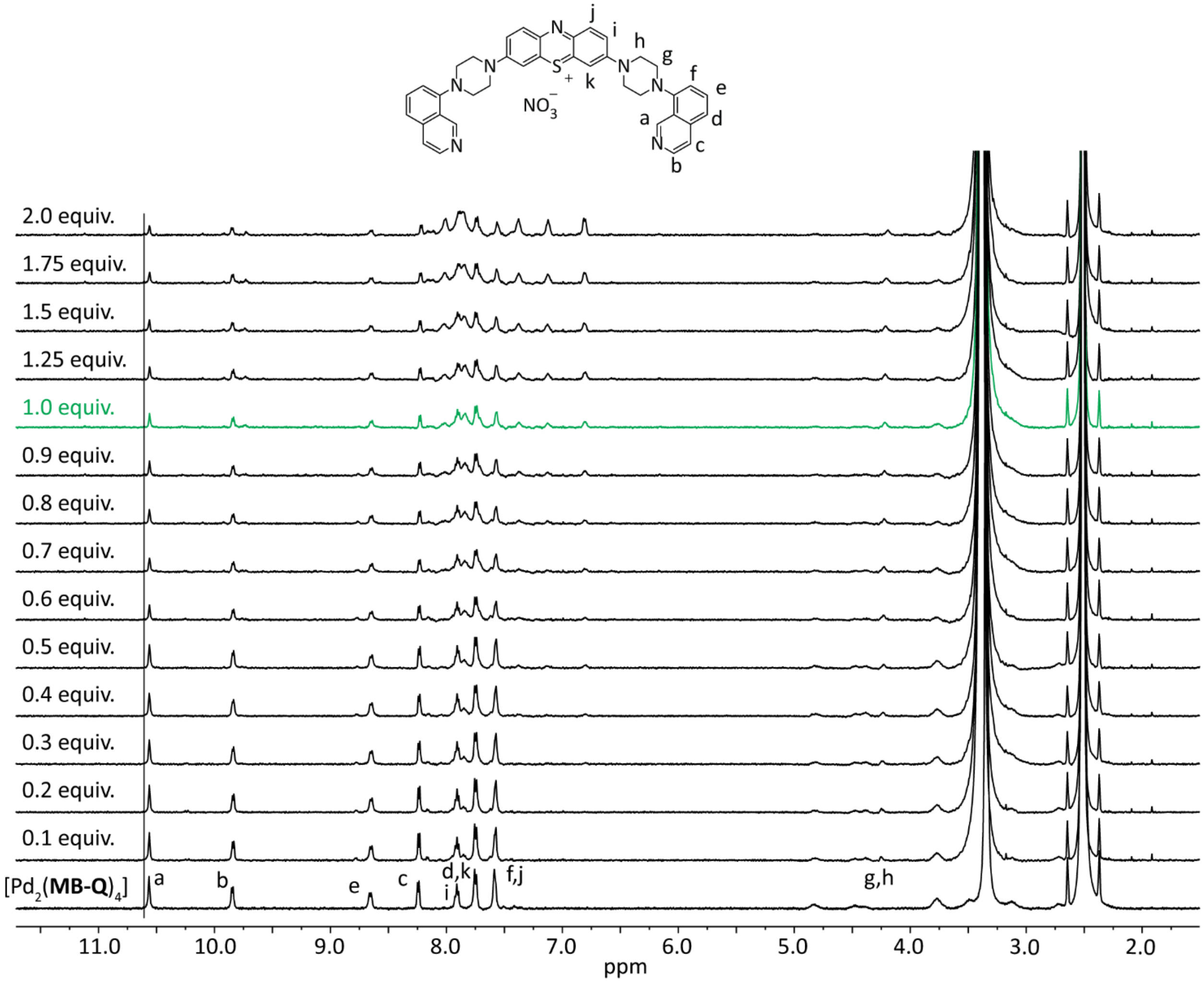


**Figure S138:** $^{1}$H NMR titration at 25 °C of guest (*R*)-**BINSO₃** in helicate [Pd₂(**MB-Q**)₄]. The green line corresponds to 1.0 equiv. of the guest. The signals that slowly increase around 7-8 ppm correspond to the free guest

### 7.2.3 ESI-MS Spectrometry

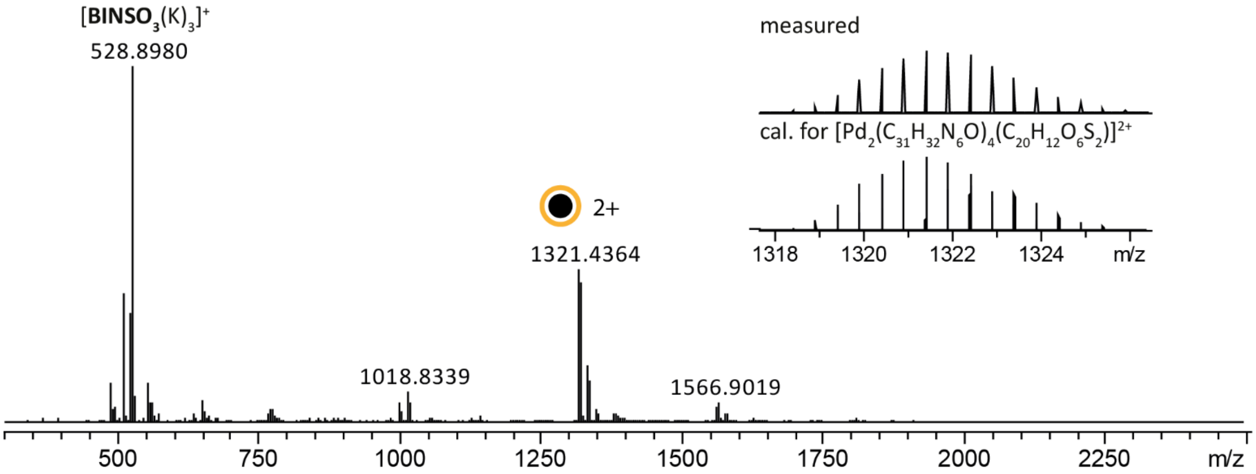


**Figure S139:** ESI-MS spectrum of cage $[Pd_2(\mathbf{MK\text{-}P})_4]$ (yellow circle) with guest (*R*)-$\mathbf{BINSO_3}$ (black dot) $[(R)\text{-}\mathbf{BINSO_3}@Pd_2(\mathbf{MK\text{-}P})_4+nBF_4]^{2-n+}$, n=0 with measure and calculated isotopic pattern of $[(R)\text{-}\mathbf{BINSO_3}@Pd_2(\mathbf{MK\text{-}P})_4]^{2+}$.

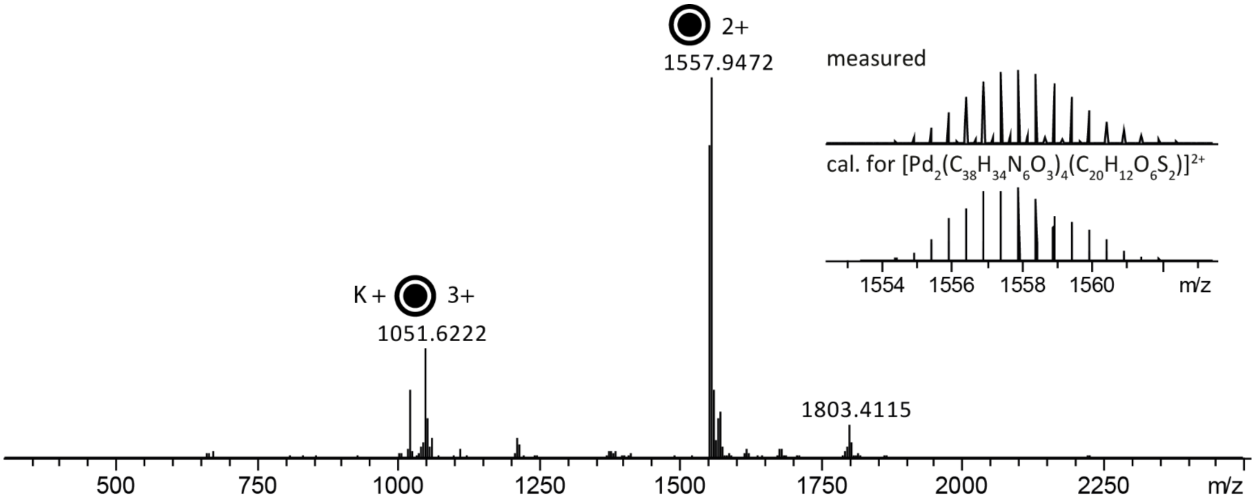


**Figure S140:** ESI-MS spectrum of cage $[Pd_2(\mathbf{RB\text{-}P})_4]$ with guest (*R*)-$\mathbf{BINSO_3}$ (black dot) b) $[(R)\text{-}\mathbf{BINSO_3}@Pd_2(\mathbf{RB\text{-}P})_4+nBF_4]^{2-n+}$, n=0 (and one $K^+$ adduct) with isotopic pattern simulation of $[(R)\text{-}\mathbf{BINSO_3}@Pd_2(\mathbf{RB\text{-}P})_4]^{2+}$.

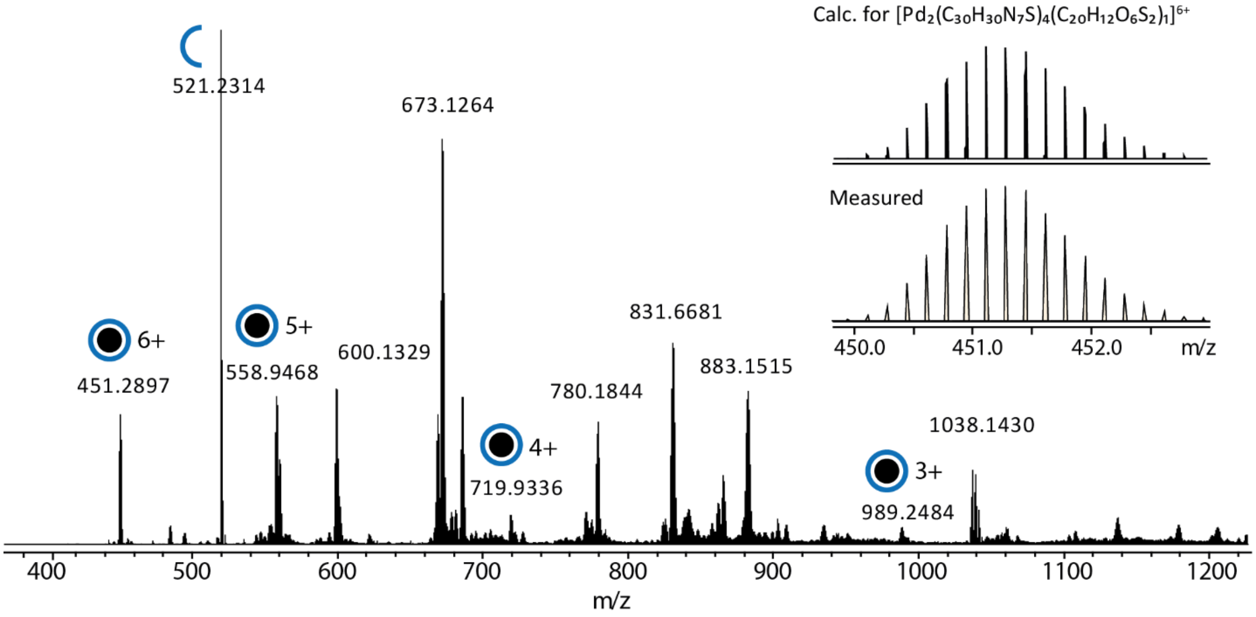


**Figure S141:** ESI-MS spectrum of cage [$Pd_2$(**MB-P**)$_4$] (blue circle) with guest (*R*)-**BINSO₃** (black dot) [(*R*)-**BINSO₃**@$Pd_2$(**MB-P**)$_4$+n$BF_4$]$^{6-n+}$, n=0-3 with isotopic pattern simulation of [(*R*)-**BINSO₃**@$Pd_2$(**MB-P**)$_4$]$^{6+}$.

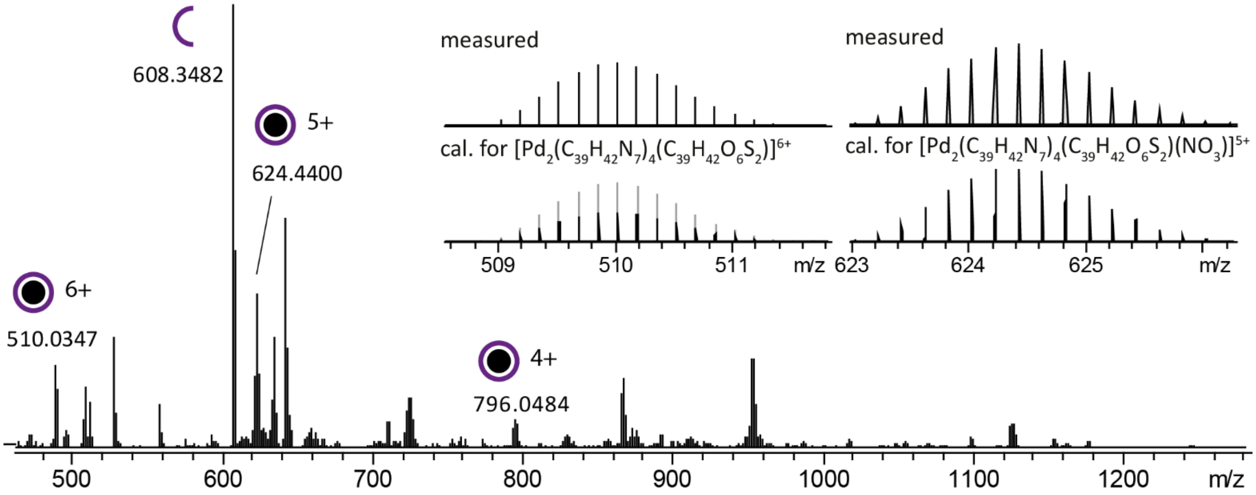


**Figure S142:** ESI-MS spectrum of cage [$Pd_2$(**CV-P**)$_4$] (violet circle) with guest (*R*)-**BINSO₃** (black dot) [(*R*)-**BINSO₃**@$Pd_2$(**CV-P**)$_4$+n$NO_3$]$^{6-n+}$, n=0-2 with isotopic pattern simulation of [(*R*)-**BINSO₃**@$Pd_2$(**CV-P**)$_4$($NO_3$)]$^{5+}$.

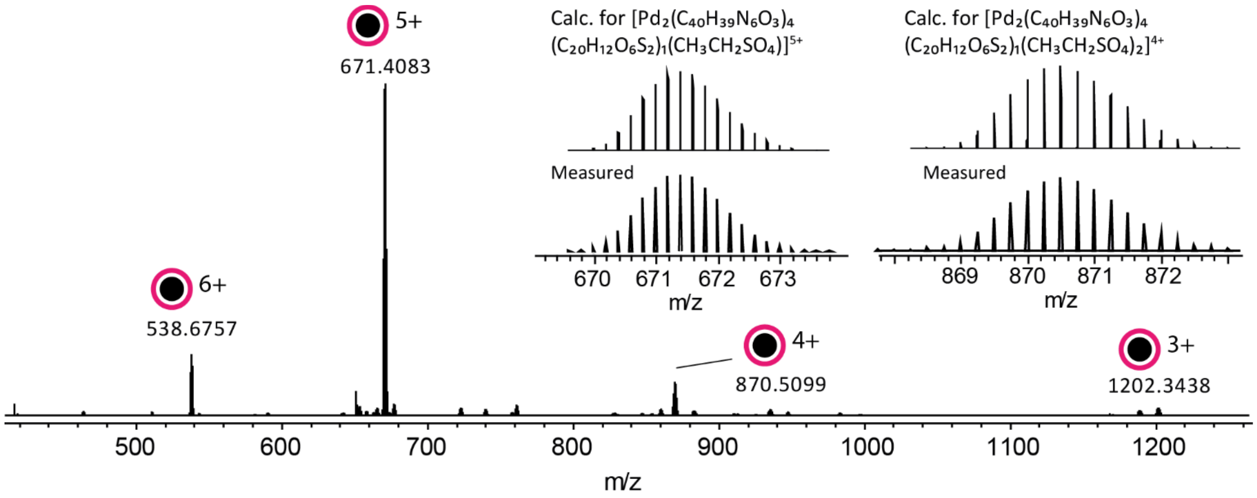


**Figure S143:** ESI-MS spectrum of cage [$Pd_2$(**RE-P**)$_4$] (pink circle) with guest (*R*)-**BINSO₃** (black dot) [(*R*)-**$BINSO_3$**@$Pd_2$(**RE-P**)$_4$+n$EtSO_4$]$^{6-n\ +}$, n=0-3 with isotopic pattern simulation of [(*R*)-**$BINSO_3$**@$Pd_2$(**RE-P**)$_4$($C_2H_5O_4S$)]$^{5+}$.

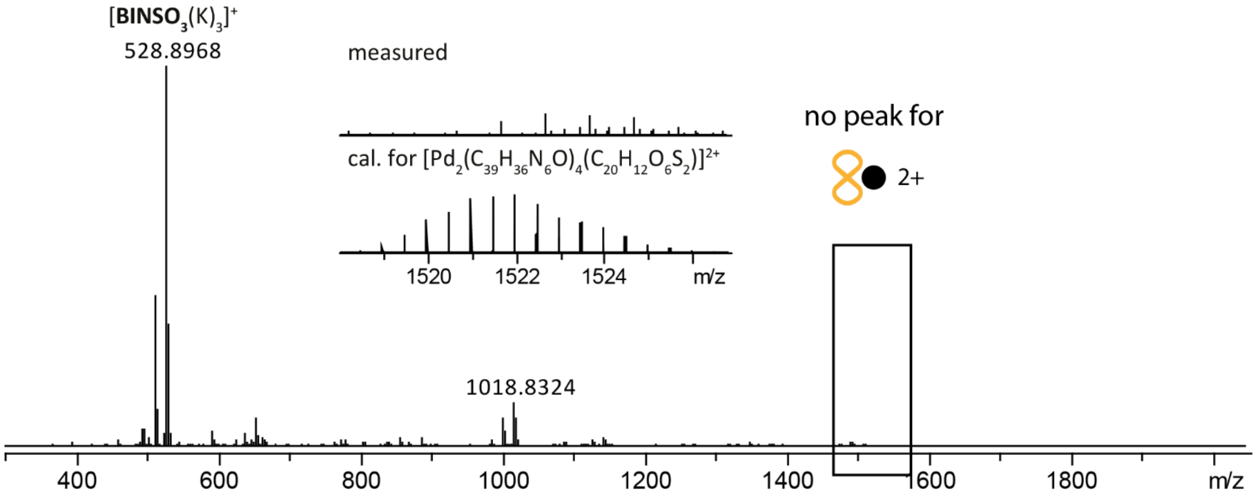


**Figure S144:** ESI-MS spectrum of helicate [$Pd_2$(**RB-Q**)$_4$] (twisted black circle) with guest (*R*)-**$BINSO_3$** (black dot), no signals for the host-guest complex nor the free helicate are found.

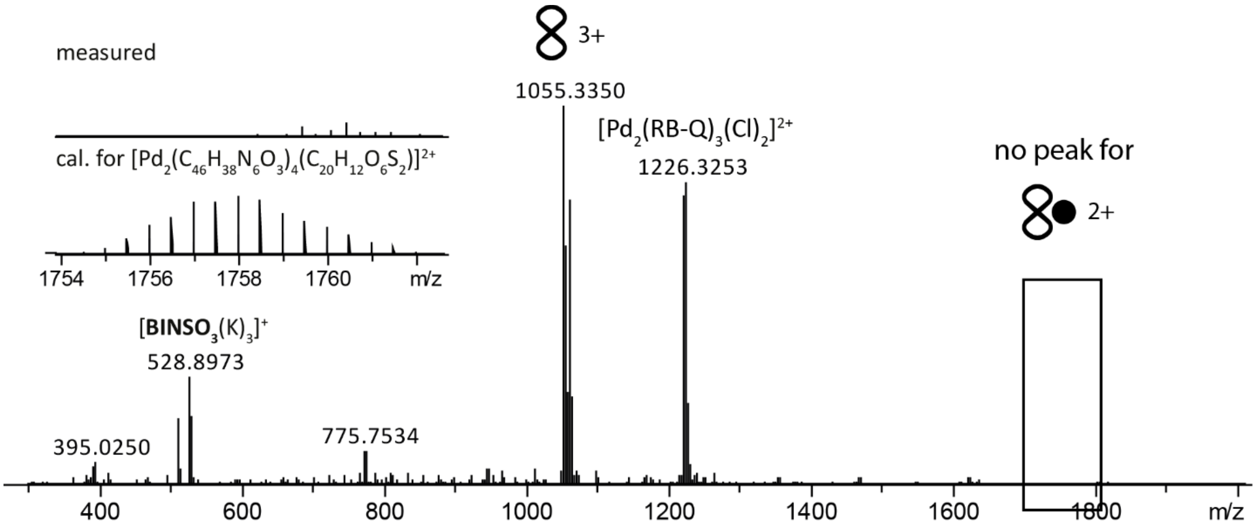


**Figure S145:** ESI-MS spectrum of helicate [Pd$_2$(**RB-Q**)$_4$] (twisted black circle) with guest (*R*)-**BINSO$_3$** (black dot), no signals for the host-guest complex are found but a signal for the free [Pd$_2$(**RB-Q**)$_4$].

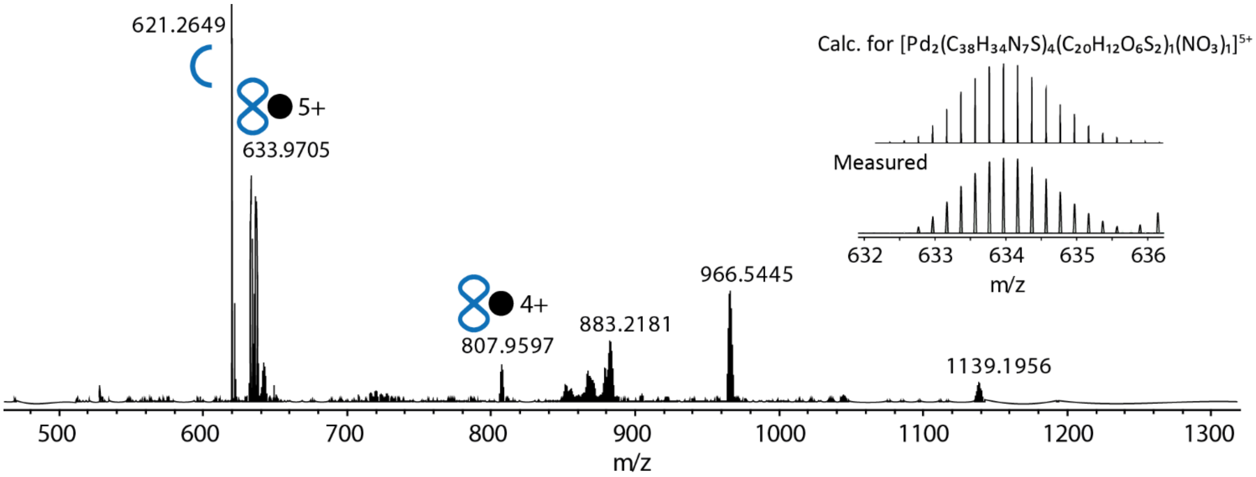


**Figure S146:** ESI-MS spectrum of helicate [Pd$_2$(**MB-Q**)$_4$] (twisted blue circle) with guest (*R*)-**BINSO$_3$** (black dot) [(*R*)-**BINSO$_3$**+Pd$_2$(**MB-Q**)$_4$+nNO$_3$]$^{6-n+}$, n=1,2 with isotopic pattern simulation of [(*R*)-**BINSO$_3$**+Pd$_2$(**MB-Q**)$_4$(NO$_3$)]$^{5+}$.

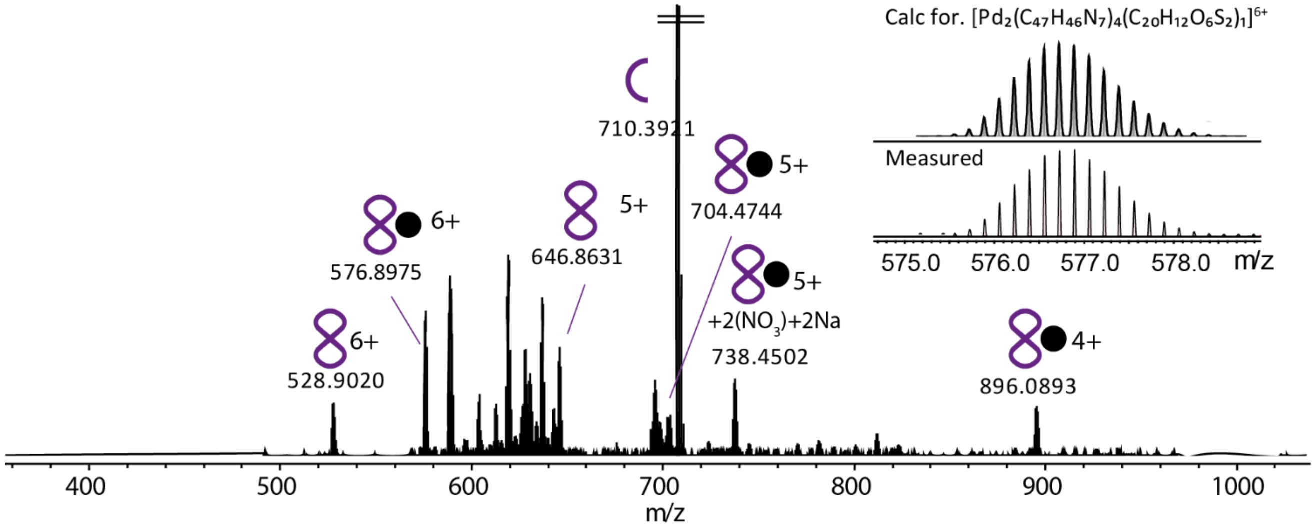


**Figure S147:** ESI-MS spectrum of helicate [$Pd_2$(**CV-Q**)$_4$] (twisted violet circle) with guest (*R*)-**BINSO₃** (black dot) [(*R*)-**BINSO₃**+$Pd_2$(**CV-Q**)$_4$+n$NO_3$]$^{6\text{-}n+}$, n=0-2 with isotopic pattern simulation of [(*R*)-**BINSO₃**+$Pd_2$(**CV-Q**)$_4$]$^{6+}$.

### 7.2.4 Competition experiments with [$Pd_2$(MB-Q)$_4$] and [$Pd_2$(RE-P)$_4$] on (*R*)-BINSO₃

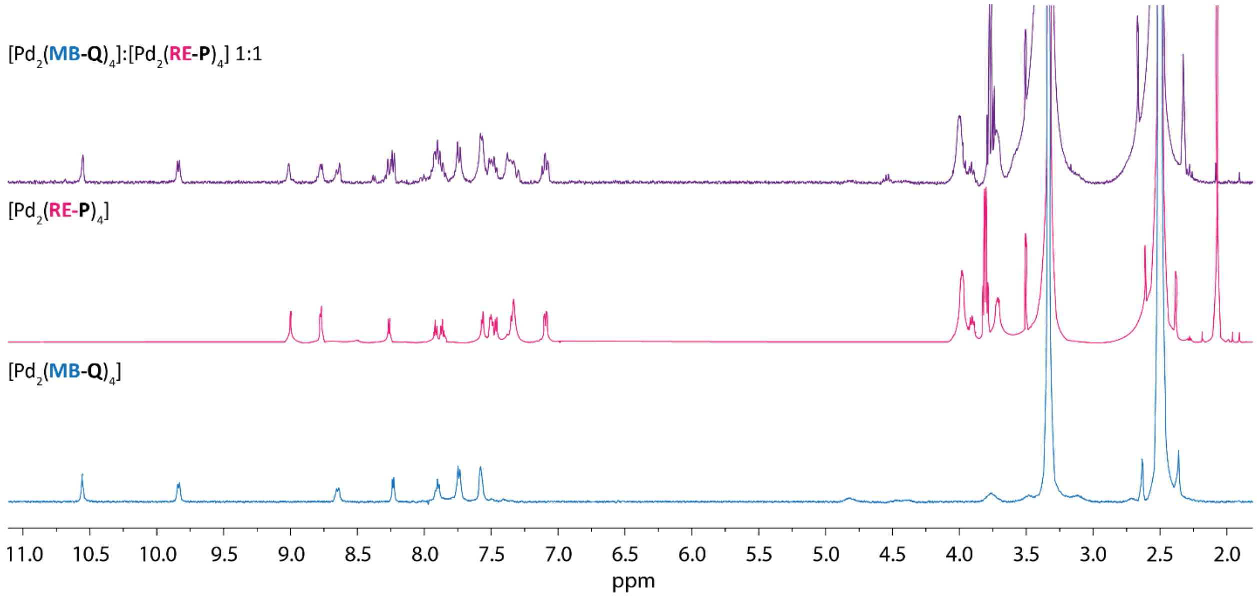


**Figure S148:** Stack of $^1$H NMR spectra at 25 °C in DMSO-$d_6$. From the bottom: helicate [$Pd_2$(**MB-Q**)$_4$] in blue, cage [$Pd_2$(**RE-P**)$_4$] in pink, kinetically-trapped, narcissistic mixture of [$Pd_2$(**MB-Q**)$_4$] and [$Pd_2$(**RE-P**)$_4$] in violet.

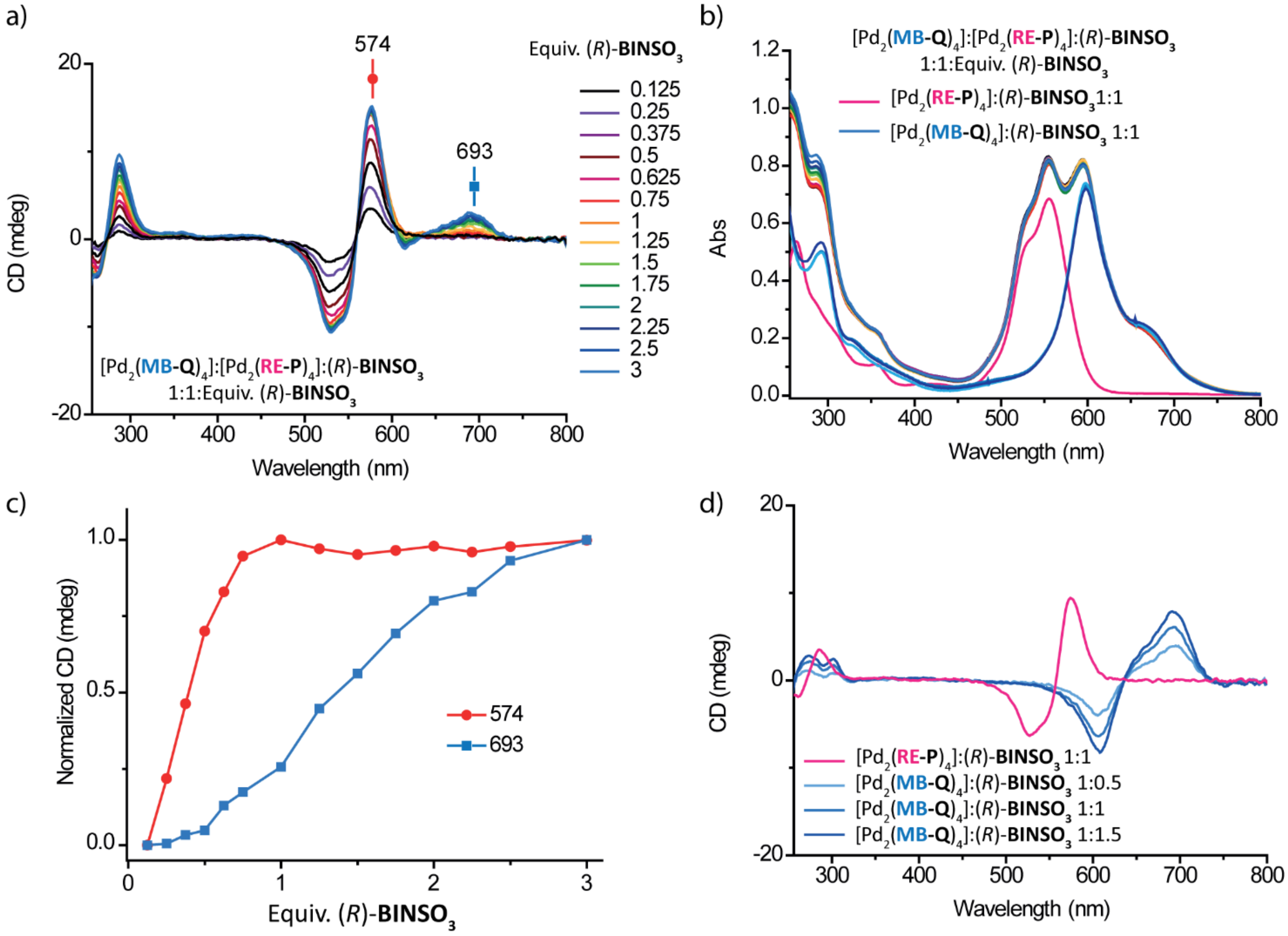


**Figure S149:** a) CD and b) UV-Vis absorption spectra of equimolar 18.75 µM DMSO solution of helicate [$Pd_2$(**MB-Q**)$_4$] and cage [$Pd_2$(**RE-P**)$_4$] with guest (*R*)-**BINSO$_3$** in 1:1:Equiv ratio. (*R*)-**BINSO$_3$** (indicated in the Figure), b) CD intensities at 574 nm (selective for [$Pd_2$(**RE-P**)$_4$]), 287 nm (not selective) and 574 nm (selective for [$Pd_2$(**MB-Q**)$_4$]), d) CD spectra of DMSO 18.75 µM solutions of [$Pd_2$(**RE-P**)$_4$] and guest (*R*)-**BINSO$_3$** in a 1:1 ratio in pink, [$Pd_2$(**MB-Q**)$_4$] and guest (*R*)-**BINSO$_3$** in 1:0.5, 1:1 and 1:1.5 ratios in different shades of blue.

### 7.2.5 Ion Mobility Measurements

Ion mobility measurements were performed on a Bruker timsTOF instrument combining a trapped ion mobility (TIMS) with a time-of-flight (TOF) mass spectrometer in one instrument.

In contrast to the conventional drift tube method to determine mobility data, where ions are carried by an electric field through a stationary drift gas, the TIMS method is based on an electric field ramp to hold ions in place against a carrier gas pushing them in the direction of the analyzer. Consequently, larger sized ions that experience more carrier gas impacts leave the TIMS units first and smaller ions elute later. This method offers a much higher mobility resolution despite a smaller device size.

Briefly, in trapped ion mobility spectrometry (TIMS) the ions are held by an electric field gradient (EFG) while exposed to a constant $N_2$ flow. The source of the flow is a pressure difference (Δp) between the entrance and the exit of the TIMS-tunnel. Lowering the EFG results in the elution of the analyte according to its inverse mobility ($1/K_0$). Depending on the difference between start-EFG and end-EFG (ΔEFG) as well as the duration of this reduction (ramp time = $t_r$), rather high ion mobility resolutions can be achieved. Resolutions of 200 and higher was targeted in all the experiments using custom mode of measurement. In front of the trapping

part of the TIMS-tunnel, a second ramp accumulates the ions for a given period of time (accumulation time = $t_a$) in order to enhance the overall duty cycle.

Since TIMS doesn't operate on first principles, a calibration is necessary. For calibration, Agilent Tune-Mix™ was used as purchased and the reported drift tube $^{DT}CCS_{N2}$ values published by Stow et al.[22] have been used. TIMS determines (calibrated) inverse mobilities which were converted to the tabulated $^{TIMS}CCS_{N2}$ values following Mason-Schamp equation:

$$\text{CCS} = \left(\frac{3}{16N}\right)\left(\frac{2\pi}{kT}\right)^{0.5}\frac{q}{\sqrt{\mu}}\frac{1}{K_0}$$

where, q is the charge of the ion, N the number density of the collision gas, µ is the reduced mass of the ion and the $N_2$ collision gas, k the Boltzmann's constant, T the temperature in Kelvin. Both the analyte solution and the calibrants have been electrosprayed in cationic mode with the same experimental conditions. Therefore, the first part of the equation is constant for every ions measured in the same condition. The constant was found by calculating the reduced mobility of the calibrant ions and plotting them against their reported CCS. The plot was fitted using linear fit with fixed intercept at 0. The slope is the constant for the specific experiment. The $1/K_0$ found from the experiment was then multiplied as per the equation.

Optimized parameters: After the generation of ions by electrospray ionisation (ESI, analyte concentration: 0.1 mg/mL solvent: Acetonitrile, capillary voltage: 2000V, end plate offset voltage: 150V, nebulizer gas pressure: 0.3 bar, dry gas flow rate: 3.0 l/min, dry temperature: 75 °C) the desired ions were orthogonally deflected into the TIMS cell consisting of an entrance funnel, the TIMS analyser (carrier gas: $N_2$, temperature: 305 K, entrance pressure: 2.72 mbar, exit pressure: 0.96 mbar, Δp = 1.77 mbar, $t_r$ = 450 ms, $t_a$ = 5 ms, a stepwise reduction of the electric field strength leads to a release of ion packages separated by their mobility. After a subsequent focussing, the separated ions are transferred to the TOF-analyser.[23-25]

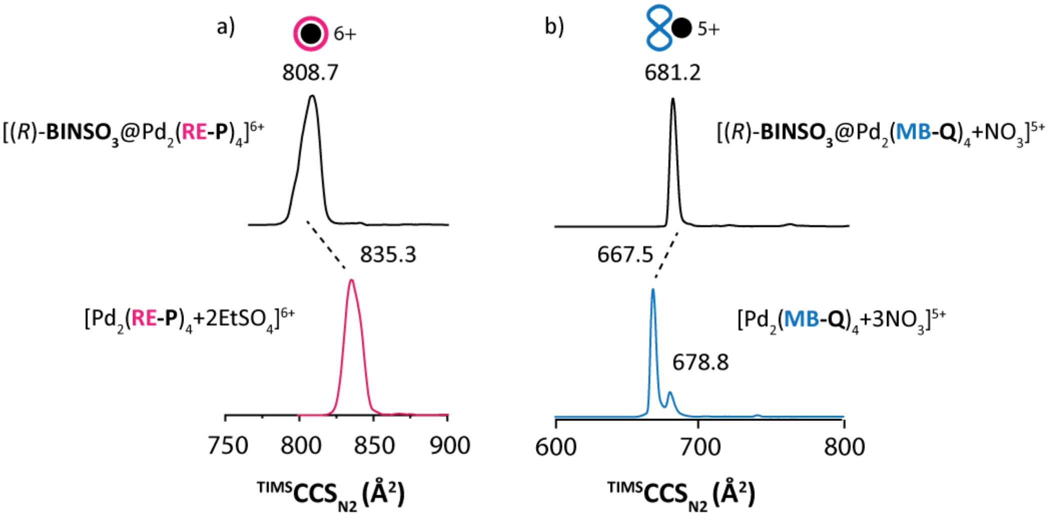


**Figure S150:** Collision cross sections obtained from high resolution trapped ion mobility spectrometry of a) $[Pd_2($**RE-P**$)_4+2EtSO_4]^{6+}$ and $[(R)$-**BINSO$_3$**@$Pd_2($**RE-P**$)_4]^{6+}$; b) $[Pd_2($**MB-Q**$)_4+3NO_3]^{5+}$ and $[(R)$-**BINSO$_3$**@$Pd_2($**MB-Q**$)_4+NO_3]^{5+}$. While the host-guest complex (in black) with cage $[Pd_2($**RE-P**$)_4]$ is showing a smaller CCS value compared to the free cage (in pink), indication that the guest is inside the cage cavity, the host-guest complex (in black) with helicate $[Pd_2($**MB-Q**$)_4]$ shows a larger CCS value compared to the free helicate (in blue). This supports the *outside* binding of the guest molecule.

References:


[1] Spartan`18 Parallel Suite, Wavefunction, Inc., Irvine.

[2] M. J. Frisch et al., Gaussian 16, Revision C. Gaussian, Inc., Wallingford CT, **2016**.

[3] a) G. H. Clever, W. Kawamura, M. Shionoya, *Inorg. Chem.* **2011**, *50*, 4689. b) G. H. Clever, S. Tashiro, M. Shionoya, *Angew. Chem. Int. Ed.* **2009**, *48*, 7010. b) G. H. Clever, S. Tashiro, M. Shionoya, *J. Am. Chem. Soc.* **2010**, *132*, 9973; c) S. Löffler, J. Lübben, A. Wuttke, R. A. Mata, M. John, B. Dittrich, G. H. Clever, *Chem. Sci.* **2016**, *7*, 4676.

[4] O. Plietzsch, A. Schade, A. Hafner, J. Huuskonen, K. Rissanen, M. Nieger, T. Muller, S. Bräse, *Eur. J. Org. Chem.* **2012**, *2013*, 283–299.

[5] J. B. Grimm, L. D. Lavis, *Org. Lett.* **2011**, *13*, 6354–7.

[6] T. Gruene, J. T. C. Wennmacher, C. Zaubitzer, J. J. Holstein, J. Heidler, A. Fecteau-Lefebvre, S. D. Carlo, E. Müller, K. N. Goldie, I. Regeni, T. Li, G. Santiso-Quinones, G. Steinfeld, S. Handschin, E. van Genderen, J. A. van Bokhoven, G. H. Clever, R. Pantelic, *Angew. Chem. Int. Ed.* **2018**, *57*, 16313–16317.

[7] A. Jerschow, N. Müller, *J. Magn. Reson.* **1996**, *123*, 222–225.

[8] A. Jerschow, N. Müller, *J. Magn. Reson.* **1998**, *132*, 13–18.

[9] E. O. Stejskal, J. E. Tanner, *J. Chem. Phys.* **1965**, *42*, 288–292.

[10] A. Macchioni, G. Ciancaleoni, C. Zuccaccia, D. Zuccaccia, *Chem. Soc. Rev.* **2007**, *37*, 479–489.

[11] L. Avram, Y. Cohen, *Chem. Soc. Rev.* **2014**, *44*, 586–602.

[12] A. Burkhardt, T. Pakendorf, B. Reime, J. Meyer, P. Fischer, N. Stube, S. Panneerselvam, O. Lorbeer, K. Stachnik, M. Warmer, P. Rodig, D. Gories, A. Meents, *Eur. Phys. J. Plus* **2016**, *131*, 1-9.

[13] W. Kabsch, *Acta Crystallogr. Sect. D* **2010**, *66*, 125-132.

[14] G. Sheldrick, *Acta Crystallogr. Sect. A* **2015**, *71*, 3-8.

[15] G. Sheldrick, *Acta Crystallogr. Sect. C* **2015**, *71*, 3-8.

[16] C. B. Hubschle, G. M. Sheldrick, B. Dittrich, *J. Appl. Crystallogr.* **2011**, *44*, 1281-1284.

[17] D. Kratzert, J. J. Holstein, I. Krossing, *J. Appl. Crystallogr*. **2015**, *48*, 933-938.

[18] A. Thorn, B. Dittrich, G. M. Sheldrick, *Acta Crystallogr. Sect. A* **2012**, *68*, 448-451.

[19] A. Spek*, Acta Crystallogr. Sect. C* **2015**, *71*, 9-18.

[20] A. Spek, *Acta Crystallogr. Sect. D* **2009**, *65*, 148-155.

[21] Bruker-Nonius, APEX, SAINT, SADABS and XPREP, Bruker AXS Inc., Madison (USA), **2013**.

[22] S. M. Stow, T. J. Causon, X. Zheng, R. T. Kurulugama, T. Mairinger, J. C. May, E. E. Rennie, E. S. Baker, R. D. Smith, J. A. McLean, S. Hann, J. C. Fjeldsted, *Anal. Chem.* **2017**, *89*, 9048-9055

[23] F. A. Fernandez-Lima, D. A. Kaplan, M. A. Park, *Rev. Sci. Instrum.* **2011**, *82*, 126106

[24] D. R. Hernandez, J. D. DeBord, M. E. Ridgeway, D. A. Kaplan, M. A. Park, F. Fernandez-Lima, *Analyst* **2014**, *139*, 1913–1921.

[25] J.-F. Greisch, J. Chmela, M. E. Harding, D. Wunderlich, B. Schäfer, M. Ruben, W. Klopper, D. Schooss, M. M. Kappes, *Phys. Chem. Chem. Phys.* **2017**, *19*, 6105–6112.
